# Regioselective Synthesis of 1,2,3-Trisubstituted Pyrroles via Addition–Cyclization of Crotonate-Derived Sulfonium Salts with a Carboxylic Acid and an Amine

**DOI:** 10.1021/acs.joc.5c03146

**Published:** 2026-03-02

**Authors:** Lahu N. Chavan, Gouthami Pashikanti, Mark M. Goodman, Lanny S. Liebeskind

**Affiliations:** † Department of Chemistry, Emory University, 1515 Dickey Drive, Atlanta, Georgia 30322, United States; ‡ 1371 Department of Radiology and Imaging Sciences, 101 Woodruff Circle, Atlanta, Georgia 30322, United States

## Abstract

A sequential addition-cyclization reaction between carboxylic acids, crotonate sulfonium salts, and amines has been developed for the construction of 1,2,3-trisubstituted pyrroles. The reaction involves regioselective addition of crotonate sulfonium salts directly to *in situ*-activated carboxylic acids to construct the first C–C bond, followed by cyclization with a primary amine to create the two C–N bonds in one pot. The new transformation appears to have a general substrate scope, uses readily accessible starting materials, and proceeds rapidly at room temperature. The reaction is versatile and scalable, making it suitable for applications in process and medicinal chemistry.

## Graphical abstract

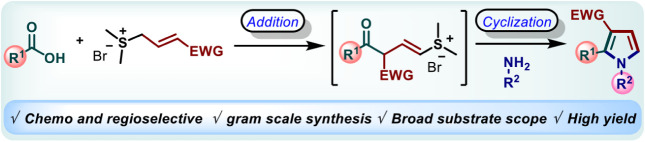

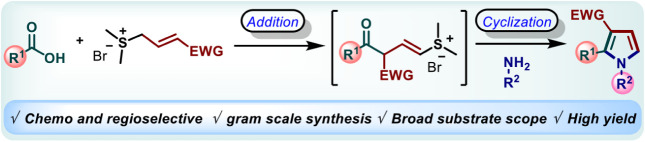

## Introduction

Functionalized heterocycles are privileged structural motifs frequently encountered in pharmaceuticals and biologically active molecules.^1,2^ Among them, pyrroles represent a fundamental class of five-membered, nitrogen-containing heterocycles found in active pharmaceutical ingredients and in top-selling drugs such as lipitor, and widely marketed dietary supplements such as methoxatin (Scheme 1).^3,4^ In addition to their medicinal importance, pyrrole derivatives serve as versatile building blocks in the synthesis of agrochemicals, dyes, flavors, and optoelectronic materials.^5^ Substituted pyrroles bearing aryl or alkyl groups at the 1-, 2-, and 3-positions are particularly valuable scaffolds.^6^ Despite established classical methods like the Knorr, Paal–Knorr, and Hantzsch syntheses, the regioselective construction of highly substituted pyrroles remains challenging.^7^ These approaches are often limited by poor regiocontrol and the chemical instability of pyrrole derivatives under harsh conditions. Consequently, the facile synthesis of polysubstituted pyrroles in a single operation directly from simple feedstocks is a useful goal in modern organic synthesis.

**Scheme 1:**
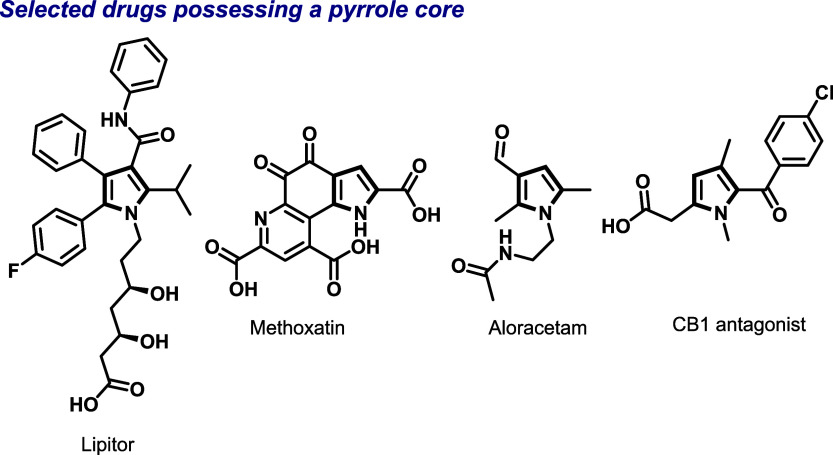
Selected Drugs and Bioactive Molecules

On the other hand, crotonate-derived sulfur ylides have attracted attention due to their diverse reactivity profiles, functioning as C_1_, C_2_, or C_3_ synthons in cascade annulations to access complex carbo- and heterocyclic frameworks (Scheme 2a).^8–10^ They have shown great potential as synthons for constructing complex carbo- and heterocycles; however, their synthetic potential remains underutilized. Notably, the corresponding allyl sulfonium salts are readily accessible from commercially available γ-bromocrotonates. These building blocks exhibit dual reactivity, combining an *α, β*-unsaturated ester moiety with an allylic sulfur ylide motif, which, upon deprotonation, generates resonance-stabilized zwitterionic intermediates capable of engaging in diverse transformations.^10c^ Inspired by our previous work on the direct synthesis of 4,5-disubstituted oxazoles from carboxylic acids,^11a^ we envisioned that *in situ* activation of carboxylic acids with triflylpyridinium reagents (a stable and easily accessible reagent for the *in situ* activation of carboxylic acids)^11b^ would engage in base-catalyzed nucleophilic addition by crotonate-derived sulfonium salts, followed by condensation with amines to furnish 1,2,3-trisubstituted pyrroles (Scheme 2b). Herein, we report the use of crotonate-derived sulfur ylides as a C_3_ synthon, reacting with *in situ* activated carboxylic acids and primary amines to synthesize polysubstituted pyrroles. The protocol offers a straightforward route to pyrroles using stable, low-cost, and readily available starting materials under mild conditions.

**Scheme 2:**
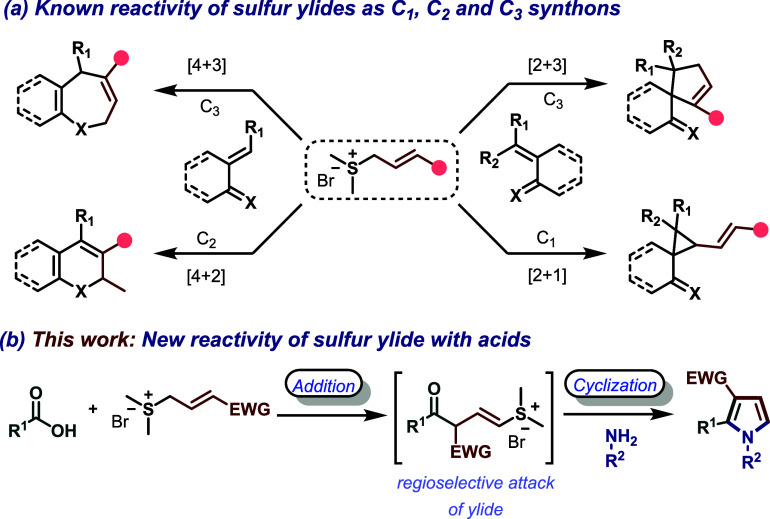
Reaction Profiles of Sulfur Ylides with Oxa/Aza-Dienes and Exploration of their New Reactivity

## Results and Discussion

To validate our proposed reaction, we conducted a series of experiments. Initially, we investigated the feasibility of the reaction using 3-fluorobenzoic acid **1a** as a model substrate. Various reaction parameters were systematically investigated to identify the optimal conditions. Initially, four different triflate-based pyridinium activators were evaluated (Table 1), as such reagents are known to promote *in situ* activation of carboxylic acids.^11b^ Among them, DMAP–Tf proved to be the most effective, affording the desired product **4aa** in 65% yield (Table 1, entry 1). In contrast, Tf–DPPD, Tf–MPLP, and Tf–DPAP provided the product in 40%, 24%, and trace yields, respectively. Further optimization of the base revealed that replacing DMAP with NEt_3_, Cs_2_CO_3_, or NaH completely suppressed product formation, whereas DABCO afforded a moderate yield (35%). Increasing the amount of DMAP to 1.5 equiv in the presence of DMAP–Tf enhanced the yield to 95% (Table 1, entry 9). Solvent screening indicated that toluene was optimal, giving the product in the highest yield (95%), while the use of DCM, MeCN, dioxane, and THF afforded slightly lower yields of 83%, 80%, 55%, and 54%, respectively (Table 1, entries 10–13). Notably, the reaction did not proceed in DMF.

**Table 1:**
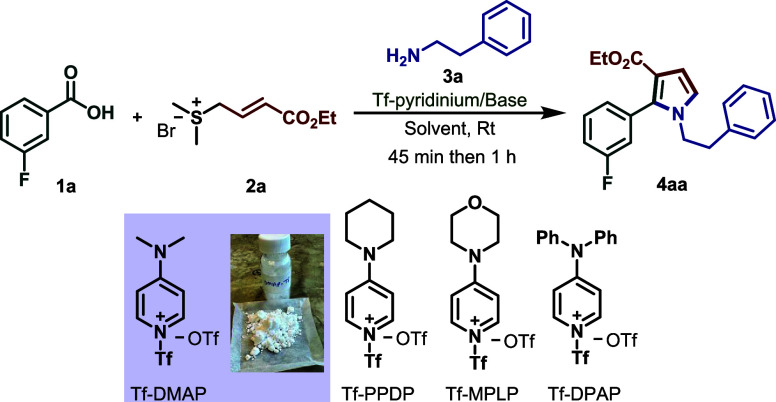
Optimization Table^a^,^b^

Entry	Tf-Pyri. (equiv)	Base (equiv)	Solvents	Yield (%)^b^
1	Tf-DMAP (1.2)	DMAP (1.3)	Toluene	65
2	Tf-PPDP (1.2)	PPDP (1.3)	Toluene	40
3	Tf-MPLP (1.2)	MPLP (1.3)	Toluene	24
4	Tf-DPAP (1.2)	DPAP (1.3)	Toluene	trace
5	Tf-DMAP (1.2)	Et_3_N (1.3)	Toluene	ND
6	Tf-DMAP (1.2)	CsCO3 (1.3)	Toluene	ND
7	Tf-DMAP (1.2)	DABCO (1.3)	Toluene	35
8	Tf-DMAP (1.2)	NaH (1.3)	Toluene	ND
**8**	**Tf-DMAP (1.3)**	**DMAP (1.5)**	**Toluene**	**95**
10	Tf-DMAP (1.3)	DMAP (1.5)	DCM	83
11	Tf-DMAP (1.3)	DMAP (1.5)	MeCN	80
12	Tf-DMAP (1.3)	DMAP (1.5)	Dioxane	55
13	Tf-DMAP (1.3)	DMAP (1.5)	THF	54
14	Tf-DMAP (1.3)	DMAP (1.5)	DMF	ND

a Standard reaction conditions: **1a** (1.0 equiv), **2a** (1.2 equiv), Tf-pyridinium (1.3 equiv), base (1.5 equiv), and amine **3a** (1.2 equiv) in toluene (0.1 M).

b Yield of the isolated product. ND: not detected.

Next, the reaction time was optimized to eliminate the undesired product **4aa′**. A mixture of acid **1a** and sulfonium salt **2a** was treated with the Tf–DMAP/DMAP reagent in toluene at room temperature and stirred for different time intervals before the addition of amine **3a**. When the amine was added after 5 min, the reaction afforded the desired product **4aa** and the undesired amide **4aa′** in a 40:60 ratio, as indicated by NMR analysis. Increasing the prestirring time to 15 min improved the ratio to 66:34, while 25 min of prestirring gave a 71:29 ratio of **4aa** to **4aa′**. Extending the reaction time to 35 min further enhanced the product selectivity to 84:16. Finally, when the amine was added after 45 min of prestirring, only the desired product **4aa** was detected by ^1^H NMR (Scheme 3).

**Scheme 3:**
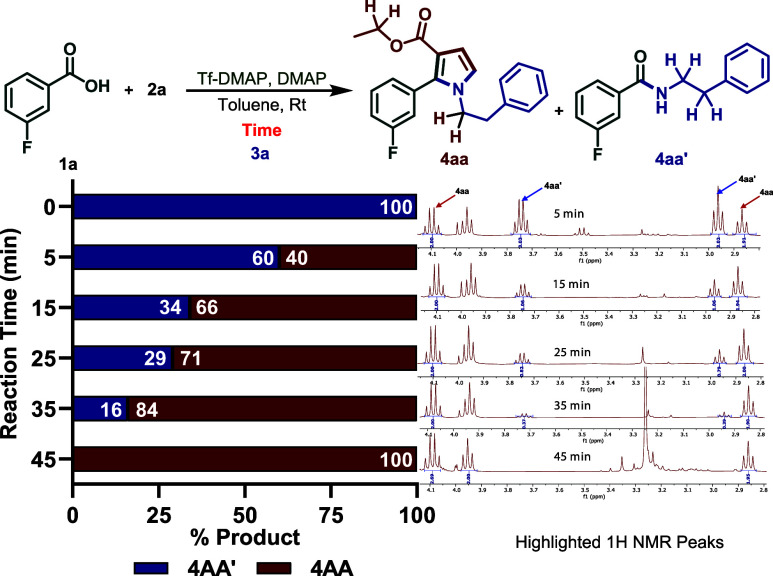
Time Optimization Study

With the optimized conditions in hand, we next evaluated the substrate scope with respect to amines (Scheme 4). A wide range of aliphatic amines reacted smoothly with **1a** and **2a**, affording the desired pyrroles in excellent yields (80–95%). A diverse set of benzylic amines bearing either electron-donating or electron-withdrawing substituents (**4ai, 4ah**) as well as heteroaromatic groups (**4ae, 4ad**) also underwent the reaction efficiently. Notably, both allylic and propargylamines were well tolerated, furnishing excellent yields of the corresponding products at 88% and 87% yield, respectively.

**Scheme 4:**
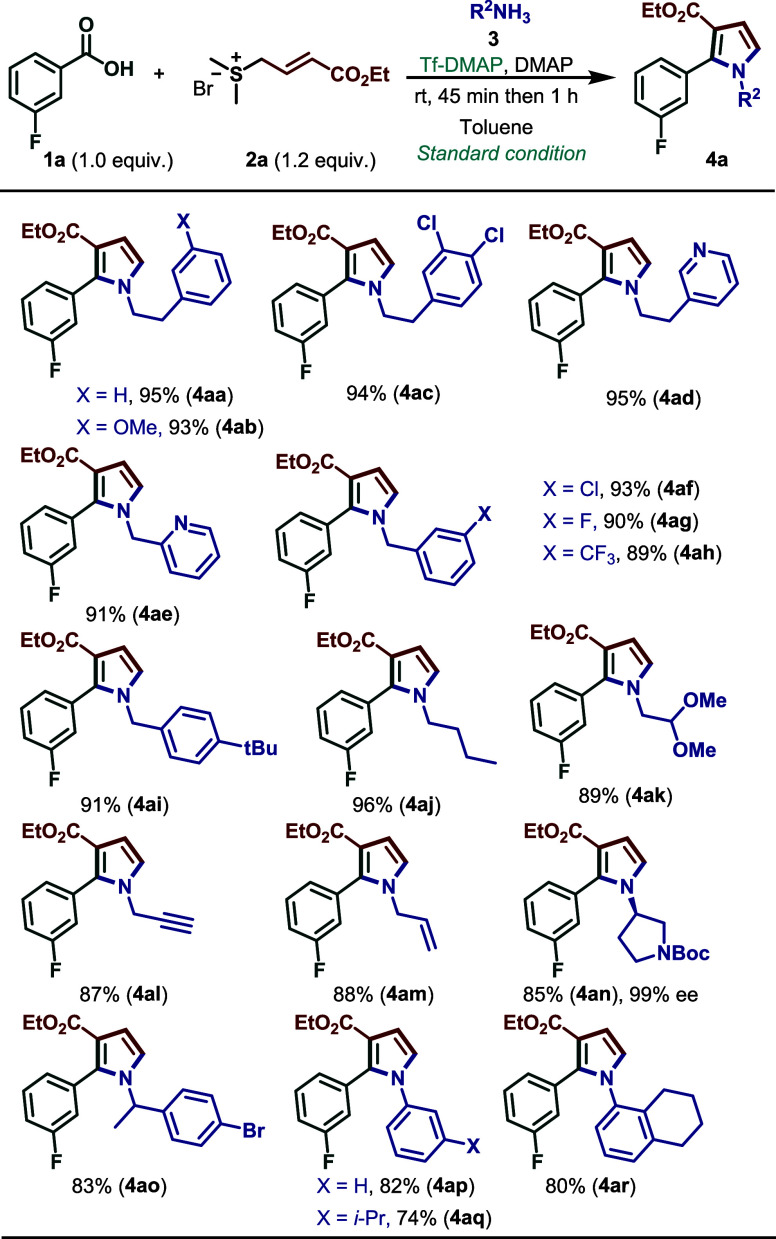
Substrate Scope for Amine^a^ ^b^

a Standard reaction conditions: **1** (1.0 equiv), **2** (1.2 equiv), DMAP-Tf (1.3 equiv), base (1.5 equiv), and **3a** (1.2 equiv) in toluene (0.1 M).
b Yield of the isolated product.

Furthermore, the transformation showed good compatibility with valuable functional groups, including chloro (**4af**), bromo (**4ao**), fluoro (**4ag**), methoxy (**4ab**), trifluoromethyl (**4ah**) and dimethoxy acetal (**4ak**) substituents. In addition, aromatic amines delivered the corresponding pyrroles (**4ap**–**4ar**) in consistently good yields (80–84%). It is important to mention that the chiral amine (*R*)-3-amino-1-Boc-pyrrolidine delivered product (**4an**) in 85% yield, without racemization, as confirmed by chiral HPLC analysis (see Supporting Information for more details).

We subsequently investigated the scope and reactivity pattern of carboxylic acids (Scheme 5). As shown, a wide range of aromatic and heteroaromatic acids underwent the transformation smoothly, affording pyrroles in good to excellent yields (67–94%). Notably, substrates bearing electron-withdrawing (**4ca**–**4ea**) and electron-neutral (**4ba**) substituents on the aromatic ring were well tolerated, whereas those containing electron-donating groups (**4va**, **4wa**) did not furnish the corresponding pyrroles but instead diverted to the corresponding amide side products, like 4aa’ above. Furthermore, a substituent at the *ortho* position on the aromatic ring was also compatible, affording the corresponding pyrrole **4xa** in excellent yield. Gratifyingly, heteroaromatic acids such as pyridine, quinoxaline, furan, thiophene, oxazole, and thiazole derivatives also reacted efficiently, providing the expected products (**4ga**–**4na**) in yields of up to 92%. Encouragingly, the methodology was further extended to aliphatic carboxylic acids, where both primary and secondary substrates reacted efficiently, affording the desired pyrroles (**4oa–4ua**) in practical yields. Moreover, 4,4-difluorocyclohexanoic acid delivered the corresponding products (**4oa** and **4ua**) in 67% and 70% yield, respectively. Importantly, variation of the sulfonium salt substituent from ethyl to methyl had no appreciable impact on the reaction outcome, as comparable yields were obtained. Finally, the chiral acid (**4sa**) delivered the desired pyrroles in 71% yield. However, chiral HPLC analysis revealed that racemization of the stereocenter occurred under the reaction conditions (see Supporting Information for more details).

**Scheme 5:**
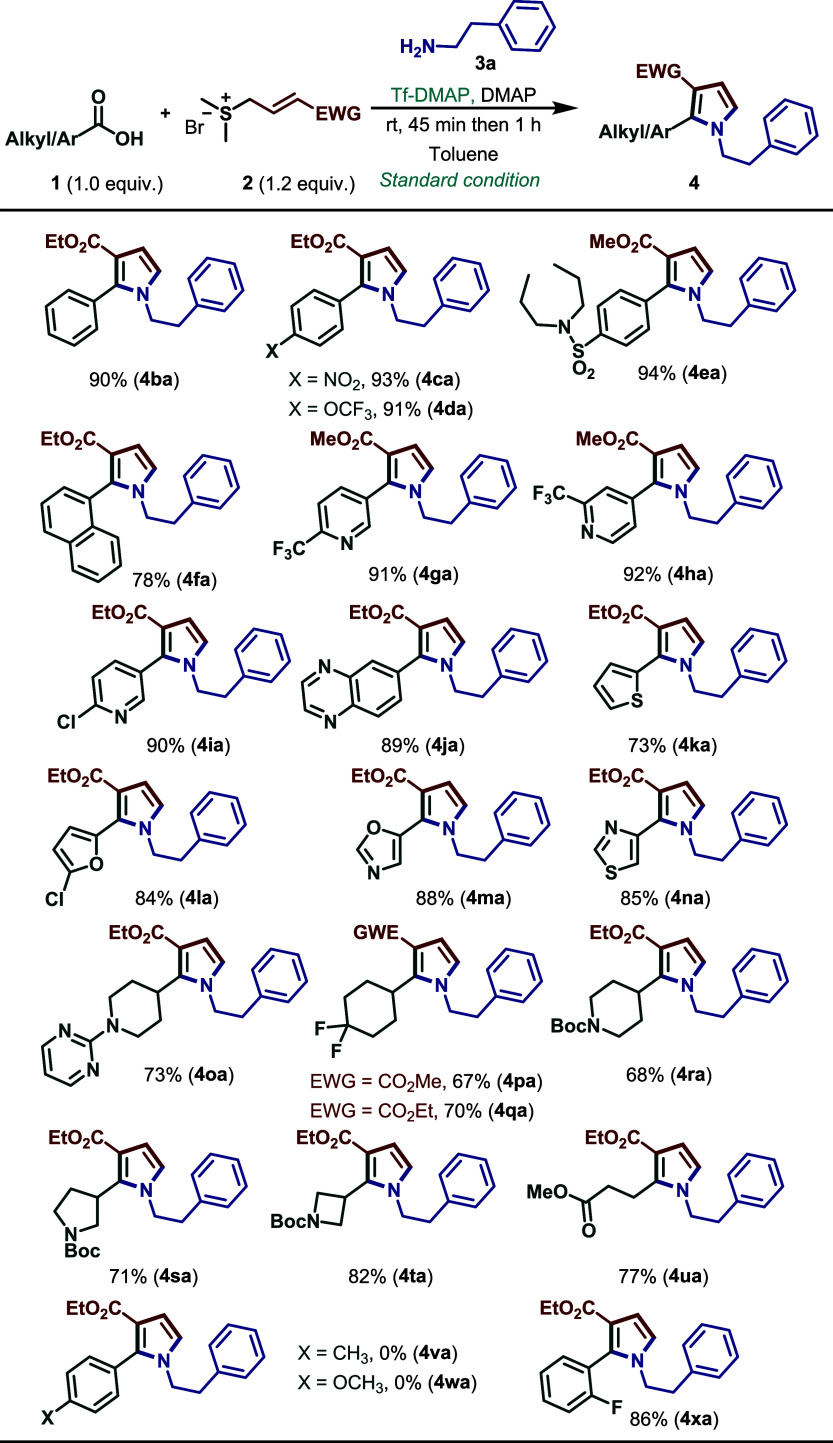
Substrate Scope for Carboxylic Acids^a^ ^b^

a Standard reaction conditions: **1** (1.0 equiv), **2** (1.2 equiv), DMAP-Tf (1.3 equiv), base (1.5 equiv), and **3a** (1.2 equiv) in toluene (0.1 M).
b Yield of the isolated product.

Next, several control experiments were conducted to elucidate the importance of preformation of the sulfonium salt and other reacting components (Scheme 6a). At first, treatment of the acid with bromocrotonate in the presence of dimethyl sulfide or thiophane (THT) and Tf-DMAP/DMAP gave only amide **4aa′** (80%), with no desired product. This observation suggests that the presynthesized sulfonium salt is crucial for the reaction. Similarly, combining acid and sulfonium salt **2a** with premixed Tf_2_O/DMAP, followed by treatment with amine **3a**, furnished product **4aa** in 32% yield and amide **4aa′** 60% yield suggesting that *in situ* DMAP triflate formation diminished the yield of the reaction. Use of an acid chloride under optimized conditions did afford the product **4aa** in 52% yield along with 15% of amide **4aa′**, but this approach is limited by the instability and poor availability of many acid chlorides. We confirmed that amide **4aa’** is not an intermediate in the pyrrole-forming pathway—it remained unreacted under the standard conditions.

**Scheme 6:**
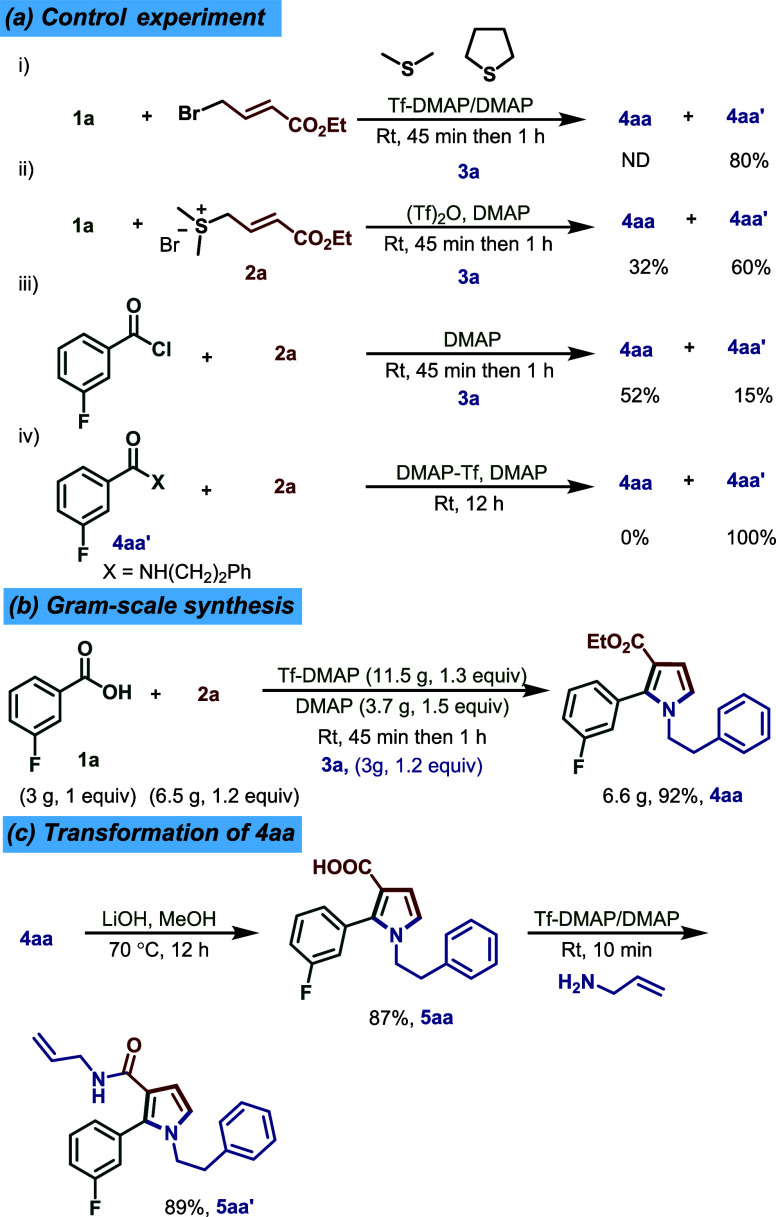
Controlled Experiments and Gram Scale Synthesis

Finally, the scalability of the method was demonstrated by performing a gram-scale synthesis of **4aa**, which proceeded smoothly under the optimized conditions to deliver the product in 92% yield (Scheme 6b). To further highlight the versatility of this chemistry, the ethyl ester moiety in **4aa** was hydrolyzed^12^ with LiOH to furnish the corresponding acid **5aa**, which was then coupled^11^ with allylamine to offer **5aa′**. This late-stage modification produced **5aa′** in 89% yield, underscoring the protocol’s utility for structural diversification (Scheme 6c).

Based on the experimental outcome and previous reports,^11^ a plausible reaction mechanism is outlined in Scheme 7. The process likely begins with the *in situ* activation of carboxylic acid substrate **1**, generating acylpyridinium salt **C**. Concurrently, the allyl sulfonium salts **2** were deprotonated with the help of base DMAP to form allyl sulfonium ylides **A** and the resonance-stabilized zwitterionic intermediate **B**. Subsequently, activated intermediate **C** undergoes nucleophilic attack by zwitterionic intermediate **B**, generating the corresponding intermediate **D**. Following a subsequent double bond shift to intermediate **E** and condensation of the keto function with amine **3** furnishes intermediate **F**. Finally, intermediate **F** undergoes C–N bond formation and aromatization to yield the desired 1,2,3-trisubstituted pyrrole derivatives **4**.

**Scheme 7:**
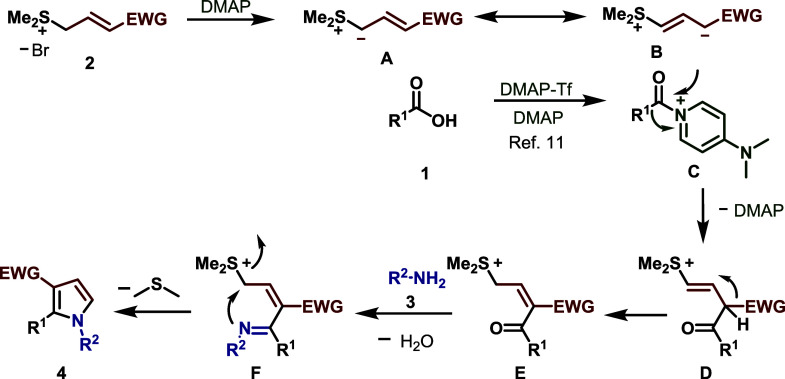
Plausible Mechanism

## Conclusion

In summary, a new reaction among carboxylic acids, crotonate-derived sulfonium salts, and amines has been developed for the efficient synthesis of 1,2,3-trisubstituted pyrroles. Control and time-optimization experiments revealed that the preformation of the sulfonium salt and a 45-min activation period are crucial for achieving complete conversion and selectivity. The transformation proceeds under mild conditions, tolerates a range of functional groups, and is scalable, demonstrating potential for applications in synthetic and medicinal chemistry.

## Experimental Section

### General Information

All solvents were purchased from Fisher Scientific or Sigma-Aldrich and dried over 4 Å molecular sieves (8–12 mesh, Sigma-Aldrich). Unless otherwise noted, all commercially available reagents and substrates were used directly as received. Thin-layer chromatography was performed on Merck silica gel plates and visualized by UV light or potassium permanganate. ^1^H, ^13^C, and ^19^F NMR spectra were recorded on Bruker 300, Varian INOVA 600, INOVA 500, and INOVA 400 spectrometers. Residual solvent resonances were treated as internal reference signals. ^19^F spectra were referenced to either trifluoroacetic acid (−76.55 ppm) or fluorobenzene (−113.15 ppm). Chemical shifts (δ) are reported in parts per million using the residual solvent peak in CDCl_3_ (H δ = 7.26 and C δ = 77.16 ppm) as an internal standard, and coupling constants (J) are given in Hz. HRMS were recorded using ESI-TOF techniques at Emory University. IR spectra were recorded on a Nicolet iS10 FT-IR spectrometer, and the absorption peaks were reported in cm^–1^. The purification of the products was performed via flash chromatography unless otherwise noted. High-resolution mass spectra were obtained from the Emory University Mass Spec Facility Inc. All solvents were dried before use by following standard procedures. Reactions were monitored using thin-layer chromatography (SiO_2_). TLC plates were visualized with UV light (254 nm), iodine treatment, or ninhydrin stain. Column chromatography was carried out using silica gel (60–120 mesh and 100–200 mesh) packed in glass columns.

## Experimental Procedures

### General Procedure for the Syntheses of 1,2,3-Trisubstituted Pyrroles from Amines (**4aa**-**4ar**)


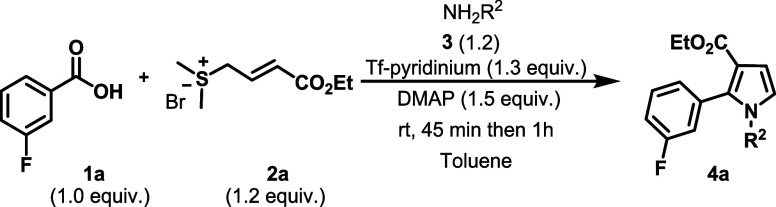


A screw-capped vial with a Teflon magnetic stirring bar was charged with carboxylic acid **1a** (0.21 mmol, 1.0 equiv), sulfonium salt **2a** (0.25 mmol, 1.2 equiv), DMAP-Tf (0.27 mmol, 1.3 equiv), DMAP (0.32 mmol, 1.5 equiv), and toluene (0.5 mL) under a dry nitrogen atmosphere. The reaction mixture was stirred for 45 min at room temperature, then amine **3** (0.25 mmol, 1.2 equiv) was added into the reaction mixture, and the reaction continued for another 60 min. After completion of the reaction, the mixture was eluted with ethyl acetate through a short silica gel column. The filtrate was concentrated under reduced pressure, and the residue was purified by column chromatography on silica gel (*n*-hexane/EtOAc) to give the desired pyrrole derivatives **4a**.

### General Procedure for the Syntheses of 1,2,3-Disubstituted Pyrroles from Carboxylic Acids (**4ba**-**3ua**)


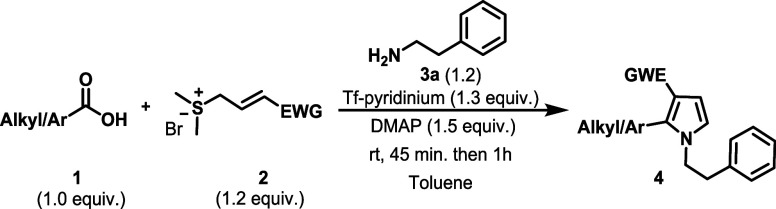


A screw-capped vial with a teflon magnetic stirrer was charged with carboxylic acid **1** (0.21 mmol, 1.0 equiv), sulfonium salt **2** (0.25 mmol, 1.2 equiv), DMAP-Tf (0.27 mmol, 1.3 equiv), DMAP (0.32 mmol, 1.5 equiv), and solvent toluene (0.5 mL) under a dry nitrogen atmosphere. The reaction mixture was stirred for 45 min at room temperature, then amine **3a** (0.25 mmol, 1.2 equiv) was added into the reaction mixture, and the reaction continued for another 60 min. After completion of the reaction, the mixture was eluted with ethyl acetate through a short silica gel column. The filtrate was then concentrated under reduced pressure, and the residue was purified by column chromatography on silica gel (*n*-hexane/EtOAc) to give the desired pyrrole derivatives **4**.

### Experimental Procedure of the Time Optimization Study

A screw-capped vial with Teflon magnetic stirring was charged with carboxylic acid **1a** (0.21 mmol, 1.0 equiv), sulfonium salt **2a** (0.27 mmol, 1.2 equiv), DMAP-Tf (0.25 mmol, 1.3 equiv), DMAP (0.32 mmol, 1.5 equiv), and solvent toluene (0.5 mL) under a dry nitrogen atmosphere. Then, amine **3a** (0.25 mmol, 1.2 equiv) was added into the reaction mixture in the time frame of 5, 15, 25, 35, and 45 min, and the reaction continued for another 60 min. After completion of the reaction (monitored by TLC), the mixture was filtered through a pad of silica gel. The combined organic layers were washed with 1 N HCl (30 mL) and dried over anhydrous sodium sulfate. The solvent was removed under reduced pressure, and the extent of formation of products **4aa** and **4aa’** was measured by ^1^H NMR analysis of the crude mixture.

### Gram-Scale Synthesis

To a screw-capped, sealable round-bottomed flask with a teflon magnetic stirrer were added 3-fluorobenzoic acid **1a** (3 g, 21 mmol, 1.0 equiv), sulfonium salt **2a** (6.5 g, 26 mmol, 1.2 equiv), DMAP-Tf (11.5 g, 27 mmol, 1.3 equiv), DMAP (3.7 g, 31 mmol, 1.5 equiv), and toluene (∼30 mL) under a dry nitrogen atmosphere. The reaction mixture was stirred for 45 min at room temperature, and then amine **3a** (3 g, 0.25 mmol, 1.2 equiv) was added into the reaction mixture, and the reaction continued for another 60 min. After completion of the reaction, the mixture was eluted with ethyl acetate through a short silica gel column and. The filtrate was concentrated under reduced pressure, and the residue was purified by column chromatography on silica gel (*n*-hexane/EtOAc) to give the desired product **4aa** in 92% (6.6 g) yield.

### General Procedure for the Synthetic Transformations General Procedure for the Synthetic Transformations:^11,12^

i) To an oven-dried 100 mL round-bottom flask, **4aa** (500 mg, 1.3 mmol, 1 equiv) was dissolved in 5 mL of anhydrous methanol, and LiOH·H_2_O (67 mg, 1.6 mmol, 1.2 equiv) was added. The solution was stirred for 11 h at 60 °C. The solvent was removed under reduced pressure, and the suspension was neutralized by adding 2 N HCl and extracted with EtOAc (2 × 50 mL). The combined organic layers were washed with brine (2 × 50 mL), dried over anhydrous sodium sulfate, and concentrated. The residue was purified by column chromatography on silica gel to afford the product **5aa** in 87% (398 mg) yield.
ii) To a screw-capped vial with a spinvane triangular-shaped teflon stirbar were charged with carboxylic acid **5aa** (100 mg, 0.32 mmol, 1.0 equiv), DMAP-Tf (156 mg, 0.38 mmol, 1.2 equiv), DMAP (51 mg, 0.42 mmol, 1.3 equiv), and solvent DCM (0.5 mL) under a dry nitrogen atmosphere. The reaction mixture was stirred for 5 min at room temperature, then allyl amine (20 mg, 0.35 mmol, 1.1 equiv) was added to the reaction mixture, and the reaction continued for another 10 min. After completion of the reaction, the mixture was filtered through a pad of silica. The filtrate was then concentrated under reduced pressure, and the residue was purified by column chromatography on silica gel (*n*-hexane/EtOAc) to give the desired product **5aa’** in 89% (100 mg) yield.

## Spectroscopic Data of Pyrrole Derivatives Obtained in This Study

### Ethyl 2-(3-Fluorophenyl)-1-phenethyl-1*H*-pyrrole-3-carboxylate (**4aa**)

Following the general procedure a: colorless liquid (57 mg, 95% yield). *R*_f_ = 0.6 (EtOAc/hexane, 2:8); ^**1**^ **H NMR** (400 MHz, CDCl_3_) δ (400 MHz, CDCl_3_) δ 7.35 (td, *J* = 8.0, 6.0 Hz, 1H), 7.27–7.21 (m, 3H), 7.10 (tdd, *J* = 8.5, 2.7, 1.0 Hz, 1H), 6.94–6.86 (m, 3H), 6.77 (m, 1H), 6.71 (d, *J* = 3.1 Hz, 1H), 6.68 (d, *J* = 3.0 Hz, 1H), 4.11 (q, *J* = 7.1 Hz, 2H), 3.96 (t, *J* = 7.2 Hz, 2H), 2.87 (t, *J* = 7.2 Hz, 2H), 1.13 (t, *J* = 7.1 Hz, 3H). ^**13**^ **C {** ^**1**^ **H} NMR** (101 MHz, CDCl_3_) δ 164.6, 162.2 (d, *J* = 246.2 Hz), 137.5, 136.8 (d, *J* = 2.2 Hz), 133.9 (d, *J* = 8.4 Hz), 129.3 (d, *J* = 8.4 Hz), 128.7, 126.9, 126.4 (d, *J* = 3.0 Hz), 120.8, 117.9 (d, *J* = 21.8 Hz), 115.2 (d, *J* = 20.9 Hz), 113.9, 110.4, 59.4, 48.6, 37.8, 14.1. ^**19**^ **F NMR** (376 MHz, CDCl_3_) δ −113.5 (td, *J* = 8.9, 5.6 Hz). **HRMS** (ESI) calcd for C_21_H_21_O_2_NF [M + H]^+^: 338.1550; found: 338.1551.

### Ethyl 2-(3-Fluorophenyl)-1-(3-methoxyphenethyl)-1*H*-pyrrole-3-carboxylate (**4ab**)

Following the general procedure a: colorless liquid (60 mg, 93% yield). *R*_f_ = 0.4 (EtOAc/hexane, 2:8); ^**1**^ **H NMR** (600 MHz, CDCl_3_) δ 7.35 (ddd, *J* = 8.5, 7.5, 5.9 Hz, 1H), 7.16 (dd, *J* = 8.3, 7.5 Hz, 1H), 7.10 (tdd, *J* = 8.5, 2.6, 1.0 Hz, 1H), 6.91 (ddd, *J* = 7.5, 1.5, 1.0 Hz, 1H), 6.79 (ddd, *J* = 8.3, 2.6, 0.9 Hz, 1H), 6.76 (ddd, *J* = 9.5, 2.6, 1.5 Hz, 1H), 6.71 (d, *J* = 3.0 Hz, 1H), 6.69 (d, *J* = 3.0 Hz, 1H), 6.48 (ddd, *J* = 7.5, 1.6, 1.0 Hz, 1H), 6.38 (dd, *J* = 2.6, 1.6 Hz, 1H), 4.10 (q, *J* = 7.1 Hz, 2H), 3.96 (t, *J* = 7.1 Hz, 2H), 3.74 (s, 3H), 2.84 (t, *J* = 7.1 Hz, 2H), 1.13 (t, *J* = 7.1 Hz, 3H). ^**13**^ **C {** ^**1**^ **H} NMR** (151 MHz, CDCl_3_) δ 164.6, 162.1 (d, *J* = 246.1 Hz), 159.7, 139.0, 136.9 (d, *J* = 2.2 Hz), 133.9 (d, *J* = 8.4 Hz), 129.7, 129.3 (d, *J* = 8.6 Hz), 126.4 (d, *J* = 3.1 Hz), 121.0, 120.8, 117.8 (d, *J* = 21.6 Hz), 115.2 (d, *J* = 21.0 Hz), 114.1, 113.8, 112.3, 110.4, 59.4, 55.1, 48.6, 37.8, 14.1. ^**19**^ **F NMR** (565 MHz, CDCl_3_) δ −113.54 to −113.60 (m). HRMS (ESI) calcd for C_22_H_23_O_3_NF [M + H]^+^: 368.1656; found: 368.1659.

### Ethyl 1-(3,4-Dichlorophenethyl)-2-(3-fluorophenyl)-1*H*-pyrrole-3-carboxylate (**4ac**)

Following the general procedure a: colorless liquid (67 mg, 94% yield). *R*_f_ = 0.6 (EtOAc/hexane, 8:2); ^**1**^ **H NMR** (600 MHz, CDCl_3_) δ 7.39–7.35 (m, 1H), 7.29 (d, *J* = 8.2 Hz, 1H), 7.12 (tdd, *J* = 8.5, 2.7, 0.9 Hz, 1H), 6.94 (d, *J* = 2.1 Hz, 1H), 6.88 (dt, *J* = 7.7, 1.2 Hz, 1H), 6.83–6.80 (m, 1H), 6.71 (d, *J* = 3.0 Hz, 1H), 6.64 (d, *J* = 3.0 Hz, 1H), 6.65–6.62 (m, 1H), 4.11 (q, *J* = 7.1 Hz, 2H), 3.98 (t, *J* = 6.9 Hz, 2H), 2.80 (t, *J* = 6.9 Hz, 2H), 1.13 (t, *J* = 7.1 Hz, 3H). ^**13**^ **C {** ^**1**^ **H} NMR** (151 MHz, CDCl_3_) δ 164.5, 162.2 (d, *J* = 246.6 Hz), 137.7, 136.7 (d, *J* = 2.2 Hz), 133.6 (d, *J* = 8.3 Hz), 132.6, 131.0, 130.6, 130.5, 129.4, 128.1, 126.4 (d, *J* = 2.9 Hz), 120.8, 117.7 (d, *J* = 21.9 Hz), 115.4 (d, *J* = 20.8 Hz), 114.1, 110.7, 59.5, 48.1, 36.8, 14.1. ^**19**^ **F NMR** (565 MHz, CDCl_3_) δ −113.09 (td, *J* = 9.0, 5.9 Hz). **HRMS** (ESI) calcd for C_21_H_19_O_2_N^35^ Cl_2_F [M + H]^+^: 406.0771; found: 406.0772.

### Ethyl 2-(3-Fluorophenyl)-1-(2-(pyridin-3-yl)­ethyl)-1*H*-pyrrole-3-carboxylate (**4ad**)

Following the general procedure a: colorless liquid (57 mg, 95% yield). *R*_f_ = 0.4 (EtOAc/hexane, 3:7); ^**1**^ **H NMR** (600 MHz, CDCl_3_) δ 8.49 (dd, *J* = 4.8, 1.7 Hz, 1H), 8.15 (d, *J* = 2.3 Hz, 1H), 7.35 (td, *J* = 8.0, 6.0 Hz, 1H), 7.17–7.14 (m, 1H), 7.13–7.08 (m, 2H), 6.90–6.87 (m, 1H), 6.82–6.79 (m, 1H), 6.71 (d, *J* = 3.1 Hz, 1H), 6.64 (d, *J* = 3.0 Hz, 1H), 4.10 (q, *J* = 7.1 Hz, 2H), 4.00 (t, *J* = 7.0 Hz, 2H), 2.85 (t, *J* = 7.0 Hz, 2H), 1.12 (t, *J* = 7.1 Hz, 3H). ^**13**^ **C {** ^**1**^ **H} NMR** (151 MHz, CDCl_3_) δ 164.4, 162.2 (d, *J* = 246.6 Hz), 149.9, 148.4, 136.6 (d, *J* = 2.2 Hz), 136.1, 133.7 (d, *J* = 8.3 Hz), 133.0, 129.5 (d, *J* = 8.4 Hz), 126.3 (d, *J* = 2.8 Hz), 123.5, 120.8, 117.8 (d, *J* = 21.8 Hz), 115.4 (d, *J* = 21.0 Hz), 114.3, 110.7, 59.4, 48.2, 34.8, 14.1. ^**19**^ **F NMR** (565 MHz, CDCl_3_) δ −113.20 (td, *J* = 9.3, 6.2 Hz). **HRMS** (ESI) calcd for C_20_H_20_O_2_N_2_F [M + H]^+^: 339.1503; found: 339.1509.

### Ethyl 2-(3-Fluorophenyl)-1-(pyridin-2-ylmethyl)-1*H*-pyrrole-3-carboxylate (**4ae**)

Following the general procedure a: colorless liquid (52 mg, 91% yield). *R*_f_ = 0.4 (EtOAc/hexane, 3:7); ^**1**^ **H NMR** (600 MHz, CDCl_3_) δ 8.54–8.51 (m, 1H), 7.62 (td, *J* = 7.7, 1.8 Hz, 1H), 7.33 (td, *J* = 7.9, 5.9 Hz, 1H), 7.20–7.17 (m, 1H), 7.10–7.04 (m, 2H), 7.02–6.99 (m, 1H), 6.80 (d, *J* = 3.1 Hz, 1H), 6.79 (d, *J* = 3.1 Hz, 1H), 6.72 (d, *J* = 7.5 Hz, 1H), 5.07 (s, 2H), 4.14 (q, *J* = 7.1 Hz, 2H), 1.15 (t, *J* = 7.1 Hz, 3H). ^**13**^ **C {** ^**1**^ **H} NMR** (151 MHz, CDCl_3_) δ 164.5, 162.2 (d, *J* = 246.5 Hz), 157.1, 149.5, 137.1 (d, *J* = 2.3 Hz), 137.0, 133.5 (d, *J* = 8.6 Hz), 129.4 (d, *J* = 8.4 Hz), 126.5 (d, *J* = 3.1 Hz), 122.6, 122.1, 120.8, 117.9 (d, *J* = 21.8 Hz), 115.5 (d, *J* = 21.0 Hz), 114.6, 111.0, 59.5, 52.6, 14.1. ^**19**^ **F NMR** (565 MHz, CDCl_3_) δ −113.40 (td, *J* = 9.1, 6.3 Hz). **HRMS** (ESI) calcd for C_19_H_18_O_2_N_2_F [M + H]^+^: 325.1346; found: 325.1349.

### Ethyl 1-(3-Chlorobenzyl)-2-(3-fluorophenyl)-1*H*-pyrrole-3-carboxylate (**4af**)

Following the general procedure a: colorless liquid (63 mg, 93% yield). *R*_f_ = 0.6 (EtOAc/hexane, 2:8); ^**1**^ **H NMR** (600 MHz, CDCl_3_) δ 7.35 (ddd, *J* = 8.4, 7.6, 5.9 Hz, 1H), 7.27–7.21 (m, 2H), 7.11 (tdd, *J* = 8.5, 2.6, 1.0 Hz, 1H), 7.04 (ddd, *J* = 7.6, 1.5, 1.0 Hz, 1H), 6.99 (ddd, *J* = 9.5, 2.6, 1.5 Hz, 1H), 6.92–6.90 (m, 1H), 6.80–6.78 (m, 1H), 6.78 (d, *J* = 3.0 Hz, 1H), 6.71 (d, *J* = 3.0 Hz, 1H), 4.93 (s, 2H), 4.14 (q, *J* = 7.1 Hz, 2H), 1.16 (t, *J* = 7.1 Hz, 3H). ^**13**^ **C {** ^**1**^ **H} NMR** (151 MHz, CDCl_3_) δ 164.5, 162.2 (d, *J* = 246.6 Hz), 139.3, 137.0 (d, *J* = 2.5 Hz), 134.7, 133.5 (d, *J* = 8.5 Hz), 130.1, 129.5 (d, *J* = 8.4 Hz), 128.0, 126.8, 126.5 (d, *J* = 3.0 Hz), 124.7, 121.8, 117.9 (d, *J* = 21.9 Hz), 115.6 (d, *J* = 20.9 Hz), 114.6, 111.0, 59.6, 50.3, 14.1. ^**19**^ **F NMR** (565 MHz, CDCl_3_) δ −113.21 (td, *J* = 9.2, 6.1 Hz). **HRMS** (ESI) calcd for C_20_H_18_O_2_N^35^ ClF [M + H]^+^: 358.1004; found: 358.1001.

### Ethyl 1-(3-Fluorobenzyl)-2-(3-fluorophenyl)-1*H*-pyrrole-3-carboxylate (**4ag**)

Following the general procedure a: colorless liquid (54 mg, 90% yield). *R*_f_ = 0.7 (EtOAc/hexane, 2:8); ^**1**^ **H NMR** (600 MHz, CDCl_3_) δ 7.35 (ddd, *J* = 8.4, 7.6, 5.9 Hz, 1H), 7.26 (td, *J* = 8.0, 5.8 Hz, 1H), 7.10 (tdd, *J* = 8.5, 2.6, 1.0 Hz, 1H), 7.05 (ddd, *J* = 7.6, 1.5, 1.0 Hz, 1H), 7.01–6.94 (m, 2H), 6.78 (d, *J* = 3.0 Hz, 1H), 6.72 (d, *J* = 3.0 Hz, 1H), 6.72 – 6.69 (m, 1H), 6.62 (ddd, *J* = 9.6, 2.6, 1.6 Hz, 1H), 4.95 (s, 2H), 4.14 (q, *J* = 7.1 Hz, 2H), 1.16 (t, *J* = 7.1 Hz, 3H). ^**13**^ **C {** ^**1**^ **H} NMR** (151 MHz, CDCl_3_) δ 164.5, 163.4 (d, *J* = 123.9 Hz), 161.8 (d, *J* = 123.3 Hz), 139.9 (d, *J* = 6.9 Hz), 137.0 (d, *J* = 2.2 Hz), 133.5 (d, *J* = 8.4 Hz), 130.4 (d, *J* = 8.3 Hz), 129.5 (d, *J* = 8.6 Hz), 126.5 (d, *J* = 3.1 Hz), 122.1 (d, *J* = 2.8 Hz), 121.8, 117.9 (d, *J* = 21.7 Hz), 115.6 (d, *J* = 20.9 Hz), 114.8 (d, *J* = 21.0 Hz), 114.5, 113.6 (d, *J* = 22.2 Hz), 110.9, 59.6, 50.3 (d, *J* = 2.0 Hz), 14.1. ^**19**^ **F NMR** (565 MHz, CDCl_3_) δ −112.22 (td, *J* = 9.2, 6.1 Hz), −113.25 to −113.30 (m). **HRMS** (ESI) calcd for C_20_H_18_O_2_NF_2_ [M + H]^+^: 342.1300; found: 342.1307.

### Ethyl 2-(3-Fluorophenyl)-1-(3-(trifluoromethyl)­benzyl)-1*H*-pyrrole-3-carboxylate (**4ah**)

Following the general procedure a: colorless liquid (61 mg, 89% yield). *R*_f_ = 0.6 (EtOAc/hexane, 2:8); ^**1**^ **H NMR** (600 MHz, CDCl_3_) δ 7.56–7.51 (m, 1H), 7.42 (t, *J* = 7.6 Hz, 1H), 7.34 (ddd, *J* = 8.4, 7.6, 5.9 Hz, 1H), 7.15 (s, 1H), 7.12 – 7.06 (m, 2H), 7.02 (ddd, *J* = 7.6, 1.5, 1.0 Hz, 1H), 6.97 (ddd, *J* = 9.4, 2.6, 1.5 Hz, 1H), 6.79 (d, *J* = 3.0 Hz, 1H), 6.74 (d, *J* = 3.0 Hz, 1H), 5.01 (s, 2H), 4.14 (q, *J* = 7.1 Hz, 2H), 1.15 (t, *J* = 7.1 Hz, 3H). ^**13**^ **C {** ^**1**^ **H} NMR** (151 MHz, CDCl_3_) δ 164.4, 162.2 (d, *J* = 246.6 Hz), 138.2, 137.0 (d, *J* = 2.2 Hz), 133.5 (d, *J* = 8.3 Hz), 131.1 (q, *J* = 32.5 Hz), 130.0, 129.6 (d, *J* = 8.6 Hz), 129.4, 126.4 (d, *J* = 3.1 Hz), 124.7 (q, *J* = 3.6 Hz), 123.5 (q, *J* = 3.8 Hz), 123.2 (q, *J* = 272.2 Hz), 121.8, 117.8 (d, *J* = 21.7 Hz), 115.6 (d, *J* = 20.9 Hz), 114.8, 111.0, 59.6, 50.5, 14.1. ^**19**^ **F NMR** (565 MHz, CDCl_3_) δ −62.71, −113.18 (td, *J* = 9.2, 6.1 Hz). **HRMS** (ESI) calcd for C_21_H_18_O_2_NF_4_ [M + H]^+^: 392.1268; found: 392.1276.

### Ethyl 1-(4-(*tert*-Butyl)­benzyl)-2-(3-fluorophenyl)-1*H*-pyrrole-3-carboxylate (**4ai**)

Following the general procedure a: colorless liquid (61 mg, 91% yield). *R*_f_ = 0.7 (EtOAc/hexane, 2:8); ^**1**^ **H NMR** (600 MHz, CDCl_3_) δ 7.38–7.34 (m, 1H), 7.32 (d, *J* = 8.5 Hz, 2H), 7.12–7.08 (m, 2H), 7.03–7.00 (m, 1H), 6.89 (d, *J* = 8.0 Hz, 2H), 6.75 (d, *J* = 3.1 Hz, 1H), 6.71 (d, *J* = 3.2 Hz, 1H), 4.91 (s, 2H), 4.14 (q, *J* = 7.1 Hz, 2H), 1.31 (s, 9H), 1.16 (t, *J* = 7.1 Hz, 3H). ^**13**^ **C {** ^**1**^ **H} NMR** (151 MHz, CDCl_3_) δ 164.6, 162.2 (d, *J* = 246.1 Hz), 150.8, 137.1 (d, *J* = 2.2 Hz), 134.2, 133.8 (d, *J* = 8.5 Hz), 129.4 (d, *J* = 8.6 Hz), 126.6 (d, *J* = 3.1 Hz), 126.5, 125.7, 121.7, 118.0 (d, *J* = 21.7 Hz), 115.4 (d, *J* = 20.9 Hz), 114.1, 110.6, 59.5, 50.5, 34.6, 31.3, 14.2. ^**19**^ **F NMR** (565 MHz, CDCl_3_) δ −113.52 (td, *J* = 9.2, 6.1 Hz). **HRMS** (ESI) calcd for C_24_H_27_O_2_NF [M + H]^+^: 380.2020; found: 380.2024.

### Ethyl 1-Butyl-2-(3-fluorophenyl)-1*H*-pyrrole-3-carboxylate (**4aj**)

Following the general procedure a: Colorless liquid (49 mg, 96% yield). *R*_f_ = 0.7 (EtOAc/hexane, 2:8); ^**1**^ **H NMR** (600 MHz, CDCl_3_) δ 7.43–7.39 (m, 1H), 7.17–7.10 (m, 2H), 7.09–7.05 (m, 1H), 6.71 (s, 2H), 4.12 (q, *J* = 7.1 Hz, 2H), 3.74 (t, *J* = 7.3 Hz, 2H), 1.62–1.54 (m, 2H), 1.20 (dt, *J* = 15.0, 7.5 Hz, 2H), 1.14 (t, *J* = 7.1 Hz, 3H), 0.82 (t, *J* = 7.4 Hz, 3H). ^**13**^ **C {** ^**1**^ **H} NMR** (151 MHz, CDCl_3_) δ 164.6, 162.2 (d, *J* = 246.0 Hz), 136.6 (d, *J* = 2.2 Hz), 134.1 (d, *J* = 8.5 Hz), 129.4 (d, *J* = 8.3 Hz), 126.6 (d, *J* = 2.9 Hz), 120.9, 117.9 (d, *J* = 21.6 Hz), 115.3 (d, *J* = 21.0 Hz), 113.8, 110.2, 59.4, 46.9, 33.2, 19.7, 14.1, 13.6. ^**19**^ **F NMR** (565 MHz, CDCl_3_) δ −113.54 (td, *J* = 9.2, 6.0 Hz). **HRMS** (ESI) calcd for C_17_H_21_O_2_NF [M + H]^+^: 290.1550; found: 290.1558.

### Ethyl 1-(2,2-Dimethoxyethyl)-2-(3-fluorophenyl)-1*H*-pyrrole-3-carboxylate (**4ak**)

Following the general procedure a: colorless liquid (51 mg, 89% yield). *R*_f_ = 0.4 (EtOAc/hexane, 2:8); ^**1**^ **H NMR** δ (600 MHz, CDCl_3_) δ 7.44–7.40 (m, 1H), 7.17–7.12 (m, 2H), 7.12–7.09 (m, 1H), 6.80 (d, *J* = 3.1 Hz, 1H), 6.71 (d, *J* = 3.0 Hz, 1H), 4.29 (t, *J* = 5.2 Hz, 1H), 4.12 (q, *J* = 7.1 Hz, 2H), 3.85 (d, *J* = 5.2 Hz, 2H), 3.26 (s, 6H), 1.14 (t, *J* = 7.1 Hz, 3H). ^**13**^ **C {** ^**1**^ **H} NMR** (151 MHz, CDCl_3_) δ 164.6, 163.1, 161.4, 136.9 (d, *J* = 2.3 Hz), 133.8 (d, *J* = 8.3 Hz), 129.6 (d, *J* = 8.4 Hz), 126.8 (d, *J* = 2.9 Hz), 122.3, 118.1 (d, *J* = 21.7 Hz), 115.5 (d, *J* = 20.8 Hz), 114.2, 110.3, 103.8, 59.5, 55.0, 49.1, 14.1. ^**19**^ **F NMR** (565 MHz, CDCl_3_) δ −113.27 (td, *J* = 8.9, 5.9 Hz). **HRMS** (ESI) calcd for C_17_H_21_O_4_NF [M + H]^+^: 322.1449; found: 322.1458.

### Ethyl 2-(3-Fluorophenyl)-1-(prop-2-yn-1-yl)-1*H*-pyrrole-3-carboxylate (**4al**)

Following the general procedure a: colorless liquid (42 mg, 87% yield). *R*_f_ = 0.7 (EtOAc/hexane, 2:8); ^**1**^ **H NMR** (600 MHz, CDCl_3_) δ 7.46–7.41 (m, 1H), 7.22–7.19 (m, 1H), 7.18–7.13 (m, 2H), 6.91 (d, *J* = 3.1 Hz, 1H), 6.76 (d, *J* = 3.1 Hz, 1H), 4.48 (d, *J* = 2.6 Hz, 2H), 4.14 (q, *J* = 7.1 Hz, 2H), 2.43 (t, *J* = 2.6 Hz, 1H), 1.16 (t, *J* = 7.1 Hz, 3H). ^**13**^ **C {** ^**1**^ **H} NMR** (151 MHz, CDCl_3_) δ 164.3, 162.3 (d, *J* = 246.6 Hz), 136.4 (d, *J* = 2.2 Hz), 133.1 (d, *J* = 8.4 Hz), 129.6 (d, *J* = 8.3 Hz), 126.6 (d, *J* = 3.1 Hz), 121.0, 118.0 (d, *J* = 22.0 Hz), 115.7 (d, *J* = 20.9 Hz), 114.6, 111.0, 77.8, 74.0, 59.6, 36.8, 14.1. ^**19**^ **F NMR** (565 MHz, CDCl_3_) δ −113.20 (td, *J* = 9.2, 6.1 Hz). HRMS (ESI) calcd for C_16_H_15_O_2_NF [M + H]^+^: 272.1081; found: 272.1087.

### Ethyl 1-Allyl-2-(3-fluorophenyl)-1*H*-pyrrole-3-carboxylate (**4am**)

Following the general procedure a: colorless liquid (42 mg, 88% yield). *R*_f_ = 0.7 (EtOAc/hexane, 2:8); ^**1**^ **H NMR** (600 MHz, CDCl_3_) δ 7.39 (td, *J* = 8.0, 5.9 Hz, 1H), 7.15–7.10 (m, 2H), 7.09–7.06 (m, 1H), 6.74 (d, *J* = 3.0 Hz, 1H), 6.70 (d, *J* = 3.0 Hz, 1H), 5.85 (ddt, *J* = 17.1, 10.3, 5.2 Hz, 1H), 5.20 (dd, *J* = 10.3, 1.4 Hz, 1H), 4.98 (dd, *J* = 17.0, 1.0 Hz, 1H), 4.34 (dt, *J* = 5.3, 1.7 Hz, 2H), 4.13 (q, *J* = 7.1 Hz, 2H), 1.15 (t, *J* = 7.1 Hz, 3H). ^**13**^ **C {** ^**1**^ **H} NMR** (151 MHz, CDCl_3_) δ 164.6, 162.2 (d, *J* = 246.2 Hz), 136.7 (d, *J* = 2.3 Hz), 133.8 (d, *J* = 8.6 Hz), 133.7, 129.3 (d, *J* = 8.4 Hz), 126.5 (d, *J* = 3.1 Hz), 121.3, 117.8 (d, *J* = 21.9 Hz), 117.5, 115.4 (d, *J* = 21.0 Hz), 114.0, 110.5, 59.4, 49.5, 14.1. ^**19**^ **F NMR** (565 MHz, CDCl_3_) δ −113.59 (td, *J* = 9.1, 5.6 Hz). **HRMS** (ESI) calcd for C_16_H_17_O_2_NF [M + H]^+^: 274.1237; found: 274.1233.

### Ethyl (*R*)-1-(1-(*tert*-Butoxycarbonyl)­pyrrolidin-3-yl)-2-(3-fluorophenyl)-1*H*-pyrrole-3-carboxylate (**4an**)

Following the general procedure a: colorless liquid (61 mg, 85% yield). *R*_f_ = 0.4 (EtOAc/hexane, 3:7); ^**1**^ **H NMR** (600 MHz, CDCl_3_) δ 7.47–7.40 (m, 1H), 7.20–7.14 (m, 1H), 7.13–7.10 (m, 1H), 7.09–7.04 (m, 1H), 6.79–6.72 (m, 1H), 4.49 (h, *J* = 6.9 Hz, 1H), 4.11 (q, *J* = 7.2 Hz, 2H), 3.73–3.34 (m, 4H), 2.24–2.09 (m, 2H), 1.48 (s, 9H), 1.13 (t, *J* = 7.2 Hz, 3H). ^**13**^ **C {** ^**1**^ **H} NMR** (151 MHz, CDCl_3_) δ 164.4, 162.4 (d, *J* = 246.8 Hz), 154.2 (d, *J* = 4.8 Hz), 136.8, 133.8 (d, *J* = 8.3 Hz), 129.8 (d, *J* = 8.6 Hz), 126.5 (d, *J* = 3.0 Hz), 117.9 (d, *J* = 21.6 Hz), 117.2, 115.7 (d, *J* = 21.1 Hz), 114.2 (d, *J* = 13.8 Hz), 111.2 (d, *J* = 15.2 Hz), 80.1 (d, *J* = 10.8 Hz), 59.6, 54.7 (d, *J* = 112.3 Hz), 51.5 (d, *J* = 84.4 Hz), 44.2 (d, *J* = 45.9 Hz), 32.7 (d, *J* = 113.4 Hz), 28.6, 14.1. ^**19**^ **F NMR** (565 MHz, CDCl_3_) δ −112.86. **HRMS** (ESI) calcd for C_22_H_27_O_4_N_2_F [M + H]^+^: 402.1962; found: 402.1957.

### Ethyl 1-(1-(4-Bromophenyl)­ethyl)-2-(3-fluorophenyl)-1*H*-pyrrole-3-carboxylate (**4ao**)

Following the general procedure a: pale yellow liquid (61 mg, 83% yield). *R*_f_ = 0.4 (EtOAc/hexane, 2:8); ^**1**^ **H NMR** (600 MHz, CDCl_3_) δ 7.43–7.40 (m, 2H), 7.39–7.32 (m, 1*H*), 7.14–7.09 (m, 1H), 7.07–6.87 (m, 2H), 6.84–6.82 (m, 2H), 6.79 (d, *J* = 3.3 Hz, 1H), 6.78 (d, *J* = 3.2 Hz, 1H), 5.13 (q, *J* = 7.1 Hz, 1H), 4.12 (q, *J* = 7.1 Hz, 2H), 1.76 (d, *J* = 7.1 Hz, 3H), 1.13 (t, *J* = 7.1 Hz, 3H). ^**13**^ **C {** ^**1**^ **H} NMR** (151 MHz, CDCl_3_) δ 164.6, 162.2 (d, *J* = 246.6 Hz), 141.3, 136.9 (d, *J* = 2.2 Hz), 133.9 (d, *J* = 8.3 Hz), 131.8, 129.6 (d, *J* = 8.4 Hz), 127.6, 126.5, 121.5, 118.1, 117.8 (d, *J* = 21.4 Hz), 115.5 (d, *J* = 20.8 Hz), 114.2, 110.7, 59.5, 54.6, 21.9, 14.1. ^**19**^ **F NMR** (565 MHz, CDCl_3_) δ −113.19. **HRMS** (ESI) calcd for C_21_H_20_O_2_N^79^ BrF [M + H]^+^: 416.0656; found: 416.0656.

### Ethyl 2-(3-Fluorophenyl)-1-phenyl-1*H*-pyrrole-3-carboxylate (**4ap**)

Following the general procedure a: colorless liquid (45 mg, 82% yield). *R*_f_ = 0.6 (EtOAc/hexane, 2:8); ^**1**^ **H NMR** (600 MHz, CDCl_3_) δ 7.32–7.26 (m, 3H), 7.24–7.18 (m, 1H), 7.10–7.06 (m, 2H), 7.01–6.95 (m, 3H), 6.91 (d, *J* = 3.0 Hz, 1H), 6.87 (d, *J* = 3.0 Hz, 1H), 4.21 (q, *J* = 7.1 Hz, 2H), 1.22 (t, *J* = 7.1 Hz, 3H). ^**13**^ **C NMR** (151 MHz, CDCl_3_) δ 164.6, 161.9 (d, *J* = 245.4 Hz), 139.2, 136.1 (d, *J* = 2.4 Hz), 133.4 (d, *J* = 8.7 Hz), 129.1, 128.9 (d, *J* = 8.4 Hz), 127.5, 127.1 (d, *J* = 3.0 Hz), 126.0, 123.1, 118.3 (d, *J* = 22.1 Hz), 115.3, 114.8 (d, *J* = 21.0 Hz), 111.3, 59.7, 14.2. ^**19**^ **F NMR** (565 MHz, CDCl_3_) δ −114.07 (q, *J* = 9.1 Hz). **HRMS** (ESI) calcd for C_21_H_22_O_2_N [M + H]^+^: 320.1645; found: 320.1651.

### Ethyl 2-(3-Fluorophenyl)-1-(3-isopropylphenyl)-1*H*-pyrrole-3-carboxylate (**4aq**)

Following the general procedure a: colorless liquid (46 mg, 74% yield). *R*_f_ = 0.6 (EtOAc/hexane, 2:8); ^**1**^ **H NMR** (400 MHz, CDCl_3_) δ 7.17–7.08 (m, 2H), 7.04–6.98 (m, 1H), 6.94–6.85 (m, 4H), 6.84 (d, *J* = 3.1 Hz, 1H), 6.76 (d, *J* = 3.1 Hz, 1H), 6.72–6.67 (m, 1H), 4.11 (q, *J* = 7.1 Hz, 2H), 2.69 (p, *J* = 6.9 Hz, 1H), 1.13 (t, *J* = 7.1 Hz, 3H), 0.99 (d, *J* = 7.0 Hz, 6H). ^**13**^ **C NMR** (101 MHz, CDCl_3_) δ 164.6, 162.0 (d, *J* = 245.3 Hz), 150.0, 139.1, 136.1, 133.7 (d, *J* = 8.5 Hz), 129.0, 128.8 (d, *J* = 8.4 Hz), 127.1 (d, *J* = 3.0 Hz), 125.7, 124.2, 123.0, 122.9, 118.3 (d, *J* = 22.2 Hz), 115.2, 114.7 (d, *J* = 21.0 Hz), 111.1, 59.7, 33.7, 23.6, 14.2. ^**19**^ **F NMR** (376 MHz, CDCl_3_) δ −114.29 to −114.39 (m). **HRMS** (ESI) calcd for C_22_H_23_O_2_NF [M + H]^+^: 352.1707; found: 352.1701.

### Ethyl 2-(3-Fluorophenyl)-1-(5,6,7,8-tetrahydronaphthalen-1-yl)-1*H*-pyrrole-3-carboxylate (**4ar**)

Following the general procedure a: colorless liquid (51 mg, 80% yield). *R*_f_ = 0.6 (EtOAc/hexane, 2:8); ^**1**^ **H NMR** (600 MHz, CDCl_3_) δ 7.16 (ddd, *J* = 8.2, 7.6, 6.0 Hz, 1H), 7.09 – 7.05 (m, 2H), 7.0–6.96 (m, 2H), 6.96–6.89 (m, 2H), 6.85 (d, *J* = 3.0 Hz, 1H), 6.70 (d, *J* = 3.0 Hz, 1H), 4.26–4.16 (m, 2H), 2.80–2.70 (m, 2H), 2.37–2.29 (m, 1H), 2.18–2.10 (m, 1H), 1.79–1.59 (m, 4H), 1.23 (t, *J* = 7.1 Hz, 3H). ^**13**^ **C {** ^**1**^ **H} NMR** (151 MHz, CDCl_3_) δ 164.7, 161.7 (d, *J* = 244.6 Hz), 138.8, 138.0, 137.0 (d, *J* = 2.2 Hz), 134.7, 133.3 (d, *J* = 8.4 Hz), 129.7, 128.6 (d, *J* = 8.3 Hz), 126.6 (d, *J* = 3.1 Hz), 125.7, 125.5, 123.1, 117.9 (d, *J* = 22.3 Hz), 114.7 (d, *J* = 21.0 Hz), 114.2, 110.9, 59.7, 29.3, 24.8, 22.6, 22.4, 14.2. ^**19**^ **F NMR** (565 MHz, CDCl_3_) δ −114.36 (td, *J* = 9.5, 5.9 Hz). **HRMS** (ESI) calcd for C_23_H_23_O_2_NF [M + H]^+^: 364.1707; found: 364.1703.

### Ethyl 1-Phenethyl-2-phenyl-1*H*-pyrrole-3-carboxylate (**4ba**)

Following the general procedure b: colorless liquid (58 mg, 90% yield). *R*_f_ = 0.6 (EtOAc/hexane, 2:8); ^**1**^ **H NMR** (600 MHz, CDCl_3_) δ 7.43–7.38 (m, 3H), 7.26–7.20 (m, 3H), 7.20–7.16 (m, 2H), 6.92–6.88 (m, 2H), 6.70 (d, *J* = 3.0 Hz, 1H), 6.64 (d, *J* = 3.0 Hz, 1H), 4.10 (q, *J* = 7.1 Hz, 2H), 3.96 (t, *J* = 7.4 Hz, 2H), 2.86 (t, *J* = 7.4 Hz, 2H), 1.12 (t, *J* = 7.1 Hz, 3H). ^**13**^ **C {** ^**1**^ **H} NMR** (151 MHz, CDCl_3_) δ 164.8, 138.4, 137.7, 131.9, 130.6, 128.7, 128.6, 128.2, 127.9, 126.7, 120.6, 113.7, 110.2, 59.3, 48.6, 37.7, 14.1. HRMS (ESI) calcd for C_21_H_22_O_2_N [M + H]^+^: 320.1645; found: 320.1651.

### Ethyl 2-(4-Nitrophenyl)-1-phenethyl-1*H*-pyrrole-3-carboxylate (**4ca**)

Following the general procedure b: pale yellow liquid (50 mg, 93% yield). *R*_f_ = 0.4 (EtOAc/hexane, 2:8); ^**1**^ **H NMR** (400 MHz, CDCl_3_) δ 8.19–8.14 (m, 2H), 7.24–7.18 (m, 3H), 7.16–7.11 (m, 2H), 6.84–6.79 (m, 2H), 6.74 (d, *J* = 3.1 Hz, 1H), 6.73 (d, *J* = 3.0 Hz, 1H), 4.08 (q, *J* = 7.1 Hz, 2H), 3.95 (t, *J* = 6.9 Hz, 2H), 2.86 (t, *J* = 6.8 Hz, 2H), 1.12 (t, *J* = 7.1 Hz, 3H). ^**13**^ **C {** ^**1**^ **H} NMR** (101 MHz, CDCl_3_) δ 164.3, 147.5, 138.6, 137.3, 135.8, 131.8, 128.8, 128.6, 127.0, 122.9, 121.5, 114.4, 111.0, 59.6, 48.7, 37.7, 14.2. **HRMS** (ESI) calcd for C_21_H_21_O_4_N_2_ [M + H]^+^: 365.1495; found: 365.1496.

### Ethyl 1-Phenethyl-2-(4-(trifluoromethoxy)­phenyl)-1*H*-pyrrole-3-carboxylate (**4da**)

Following the general procedure b: colorless liquid (44 mg, 91% yield). *R*_f_ = 0.5 (EtOAc/hexane, 2:8); ^**1**^ **H NMR** (600 MHz, CDCl_3_) δ 7.26–7.19 (m, 5H), 7.13–7.07 (m, 2H), 6.89–6.83 (m, 2H), 6.72 (d, *J* = 3.0 Hz, 1H), 6.69 (d, *J* = 3.0 Hz, 1H), 4.10 (q, *J* = 7.1 Hz, 2H), 3.96 (t, *J* = 7.1 Hz, 2H), 2.87 (t, *J* = 7.1 Hz, 2H), 1.11 (t, *J* = 7.1 Hz, 3H)^.**13**^ **C {** ^**1**^ **H} NMR** (151 MHz, CDCl_3_) δ 164.6, 149.1, 137.5, 136.8, 132.2, 130.6, 128.7, 128.7, 126.8, 122.2 (q, *J* = 257.1 Hz), 120.8, 120.3, 114.0, 110.5, 59.4, 48.6, 37.8, 14.0. ^**19**^ **F NMR** (565 MHz, CDCl_3_) δ −57.75. **HRMS** (ESI) calcd for C_22_H_21_O_3_NF_3_ [M + H]^+^: 404.1468; found: 404.1471.

### Ethyl 2-(4-(*N*,*N*-Dipropylsulfamoyl)­phenyl)-1-phenethyl-1*H*-pyrrole-3-carboxylate (**4ea**)

Following the general procedure b: colorless liquid (39 mg, 94% yield). *R*_f_ = 0.3 (EtOAc/hexane, 6:4); (0.510 g, 85% yield). ^**1**^ **H NMR** (600 MHz, CDCl_3_) δ 7.81–7.76 (m, 2H), 7.27–7.20 (m, 3H), 7.19–7.14 (m, 2H), 6.86–6.81 (m, 2H), 6.74 (d, *J* = 3.0 Hz, 1H), 6.72 (d, *J* = 3.0 Hz, 1H), 4.09 (q, *J* = 7.1 Hz, 2H), 3.93 (t, *J* = 7.0 Hz, 2H), 3.15–3.10 (m, 4H), 2.85 (t, *J* = 7.0 Hz, 2H), 1.66–1.56 (m, 4H), 1.10 (t, *J* = 7.1 Hz, 3H), 0.92 (t, *J* = 7.4 Hz, 6H). ^**13**^ **C {** ^**1**^ **H} NMR** (151 MHz, CDCl_3_) δ 164.5, 139.7, 137.4, 136.3, 136.1, 131.4, 128.7, 128.6, 126.9, 126.5, 121.2, 114.2, 110.9, 59.4, 50.2, 48.6, 37.7, 22.2, 14.1, 11.2. **HRMS** (ESI) calcd for C_27_H_35_O_4_N_2_^32^ S [M + H]^+^: 483.2325; found: 483.2317.

### Ethyl 2-(Naphthalen-1-yl)-1-phenethyl-1*H*-pyrrole-3-carboxylate (**4fa**)

Following the general procedure b: colorless liquid (41 mg, 78% yield). *R*_f_ = 0.7 (EtOAc/hexane, 2:8); ^**1**^ **H NMR** (400 MHz, CDCl_3_) δ 7.92–7.83 (m, 2H), 7.83–7.74 (m, 1H), 7.59–7.47 (m, 3H), 7.31–7.27 (m, 2H), 7.25–7.19 (m, 3H), 6.92–6.83 (m, 2H), 6.76 (d, *J* = 3.1 Hz, 1H), 6.71 (d, *J* = 3.1 Hz, 1H), 4.08 (q, *J* = 7.1 Hz, 2H), 4.01 (t, *J* = 7.1 Hz, 2H), 2.90 (t, *J* = 7.1 Hz, 2H), 1.05 (t, *J* = 7.1 Hz, 3H). ^**13**^ **C {** ^**1**^ **H} NMR** (101 MHz, CDCl_3_) δ 164.8, 138.4, 137.7, 133.0, 132.8, 129.8, 129.3, 128.7, 128.6, 128.5, 128.2, 127.7, 127.3, 126.7, 126.4, 126.1, 120.6, 113.9, 110.4, 59.3, 48.6, 37.7, 14.1. **HRMS** (ESI) calcd for C_25_H_24_O_2_N [M + H]^+^: 370.1801; found: 370.1796.

### Methyl 1-Phenethyl-2-(6-(trifluoromethyl)­pyridin-3-yl)-1*H*-pyrrole-3-carboxylate (**4ga**)

Following the general procedure b: colorless liquid (43 mg, 91% yield). *R*_f_ = 0.5 (EtOAc/hexane, 4:6); ^**1**^ **H NMR** (600 MHz, CDCl_3_) δ 8.42–8.40 (m, 1H), 7.61 (dd, *J* = 8.0, 0.9 Hz, 1H), 7.33–7.36 (m, 1H), 7.27–7.20 (m, 3H), 6.83–6.81 (m, 1H), 6.80 (d, *J* = 3.1 Hz, 1H), 6.77 (d, *J* = 3.0 Hz, 1H), 4.02 (t, *J* = 6.7 Hz, 2H), 3.66 (s, 3H), 2.90 (t, *J* = 6.7 Hz, 2H). ^**13**^ **C {** ^**1**^ **H} NMR** (151 MHz, CDCl_3_) δ 164.6, 151.0, 147.3 (q, *J* = 34.9 Hz), 139.8, 137.1, 133.2, 130.6, 128.8, 128.6, 127.1, 123.3 (q, *J* = 274.2 Hz), 121.9, 119.4 (q, *J* = 2.7 Hz), 114.8, 111.2, 51.0, 48.6, 37.8. ^**19**^ **F NMR** (565 MHz, CDCl_3_) δ −67.85. **HRMS** (ESI) calcd for C_20_H_18_O_2_N_2_F_3_ [M + H]^+^: 375.1314; found: 375.1320.

### Methyl 1-Phenethyl-2-(2-(trifluoromethyl)­pyridin-4-yl)-1*H*-pyrrole-3-carboxylate (**4ha**)

Following the general procedure b: colorless liquid (44 mg, 92% yield). *R*_f_ = 0.5 (EtOAc/hexane, 4:6); ^**1**^ **H NMR** (600 MHz, CDCl_3_) δ 8.67 (d, *J* = 4.9 Hz, 1H), 7.28–7.24 (m, 1H), 7.23–7.18 (m, 3H), 7.04 (dd, *J* = 4.9, 1.5 Hz, 1H), 6.84 (d, *J* = 3.1 Hz, 1H), 6.78 (d, *J* = 3.1 Hz, 2H), 6.77–6.75 (m, 1H), 4.01 (t, *J* = 6.6 Hz, 2H), 3.65 (s, 3H), 2.90 (t, *J* = 6.6 Hz, 2H). ^**13**^ **C {** ^**1**^ **H} NMR** (151 MHz, CDCl_3_) δ 164.5, 149.5, 147.7 (q, *J* = 34.7 Hz), 141.4, 136.9, 134.0, 128.8, 128.5, 128.1, 127.2, 122.3 (q, *J* = 2.9 Hz), 122.1, 119.6 (d, *J* = 274.3 Hz), 114.3, 111.5, 51.1, 48.7, 37.8. ^**19**^ **F NMR** (565 MHz, CDCl_3_) δ −67.78. **HRMS** (ESI) calcd for C_20_H_18_O_2_N_2_F_3_ [M + H]^+^: 375.1314; found: 375.1318.

### Ethyl 2-(6-Chloropyridin-3-yl)-1-phenethyl-1*H*-pyrrole-3-carboxylate (**4ia**)

Following the general procedure b: colorless liquid (50 mg, 90% yield). *R*_f_ = 0.4 (EtOAc/hexane, 4:6); ^**1**^ **H NMR** (400 MHz, CDCl_3_) δ 7.96–7.93 (m, 1H), 7.21–7.12 (m, 3H), 7.07 (dd, *J* = 8.2, 2.4 Hz, 1H), 6.80–6.71 (m, 2H), 6.67 (d, *J* = 3.1 Hz, 1H), 6.66 (d, *J* = 3.1 Hz, 1H), 4.01 (q, *J* = 7.1 Hz, 2H), 3.89 (t, *J* = 6.8 Hz, 3H), 2.80 (t, *J* = 6.8 Hz, 3H), 1.05 (t, *J* = 7.1 Hz, 3H). ^**13**^ **C {** ^**1**^ **H} NMR** (101 MHz, CDCl_3_) δ 164.3, 151.1, 150.7, 141.1, 137.2, 133.2, 128.8, 128.6, 127.0, 126.8, 123.3, 121.5, 115.0, 111.1, 59.6, 48.5, 37.8, 14.1. **HRMS** (ESI) calcd for C_20_H_20_O_2_N_2_^35^ Cl [M + H]^+^: 355.1207; found: 355.1215.

### Ethyl 1-Phenethyl-2-(quinoxalin-6-yl)-1*H*-pyrrole-3-carboxylate (**4ja**)

Following the general procedure b: colorless liquid (47 mg, 89% yield). *R*_f_ = 0.5 (EtOAc/hexane, 3:7); ^**1**^ **H NMR** (600 MHz, CDCl_3_) δ 8.91 (s, 2H), 8.09 (dd, *J* = 8.6, 0.6 Hz, 1H), 7.95 (dd, *J* = 1.9, 0.6 Hz, 1H), 7.51 (dd, *J* = 8.6, 1.9 Hz, 1H), 7.24 – 7.17 (m, 3H), 6.86 – 6.83 (m, 2H), 6.77 (d, *J* = 3.0 Hz, 1H), 6.74 (d, *J* = 3.1 Hz, 1H), 4.10 (q, *J* = 7.1 Hz, 2H). 4.07 (t, *J* = 7.1 Hz, 2H), 2.89 (t, *J* = 7.1 Hz, 2H), 1.08 (t, *J* = 7.1 Hz, 3H). ^**13**^ **C {** ^**1**^ **H} NMR** (151 MHz, CDCl_3_) δ: 162.0, 155.6, 149.0, 130.5, 128.5, 128.4, 126.7, 126.6, 61.4, 14.2. **HRMS** (ESI) calcd for C_23_H_22_O_2_N_3_ [M + H]^+^: 372.1706; found: 372.1711.

### Ethyl 1-Phenethyl-2-(thiophen-2-yl)-1*H*-pyrrole-3-carboxylate (**4ka**)

Following the general procedure b: colorless liquid (46 mg, 73% yield). *R*_f_ = 0.6 (EtOAc/hexane, 2:8); ^**1**^ **H NMR** (600 MHz, CDCl_3_) δ 7.48 (dd, *J* = 5.2, 1.2 Hz, 1H), 7.28–7.21 (m, 3H), 7.11 (dd, *J* = 5.2, 3.5 Hz, 1H), 7.00–6.97 (m, 2H), 6.90 (dd, *J* = 3.5, 1.2 Hz, 1H), 6.69 (d, *J* = 3.0 Hz, 1H), 6.65 (d, *J* = 3.1 Hz, 1H), 4.15 (q, *J* = 7.1 Hz, 2H), 4.03 (t, *J* = 7.5 Hz, 2H), 2.94 (t, *J* = 7.5 Hz, 2H), 1.17 (t, *J* = 7.1 Hz, 3H). ^**13**^ **C {** ^**1**^ **H} NMR** (151 MHz, CDCl_3_) δ 164.4, 137.8, 131.5, 129.9, 129.5, 128.7, 128.6, 127.5, 126.8, 126.7, 121.7, 116.2, 110.5, 59.4, 48.8, 38.0, 14.1. **HRMS** (ESI) calcd for C_19_H_20_O_2_N^32^ S [M + H]^+^: 326.1209; found: 326.1213.

### Ethyl 2-(5-Chlorofuran-2-yl)-1-phenethyl-1*H*-pyrrole-3-carboxylate (**4la**)

Following the general procedure b: colorless liquid (49 mg, 84% yield). *R*_f_ = 0.5 (EtOAc/hexane, 2:8); ^**1**^ **H NMR** (600 MHz, CDCl_3_) δ 7.31–7.29 (m, 3H), 7.27–7.23 (m, 1H), 7.10–7.05 (m, 2H), 6.66 (d, *J* = 3.0 Hz, 1H), 6.60 (d, *J* = 3.0 Hz, 1H), 6.59 (d, *J* = 3.3 Hz, 1H), 6.30 (d, *J* = 3.4 Hz, 1H), 4.22 (q, *J* = 7.1 Hz, 2H), 4.14 (t, *J* = 7.4 Hz, 2H), 3.00–2.97 (t, *J* = 7.4 Hz, 2H), 1.27 (t, *J* = 7.1 Hz, 3H). ^**13**^ **C {** ^**1**^ **H} NMR** (151 MHz, CDCl_3_) δ 164.1, 143.7, 137.7, 136.4, 128.7, 128.7, 126.8, 125.5, 122.8, 116.3, 115.1, 110.9, 107.7, 59.7, 50.1, 37.9, 14.2. **HRMS** (ESI) calcd for C_19_H_19_O_3_N^35^ Cl [M + H]^+^: 344.1048; found: 344.1051.

### Ethyl 2-(Oxazol-5-yl)-1-phenethyl-1*H*-pyrrole-3-carboxylate (**4ma**)

Following the general procedure b: white solid (60 mg, 88% yield). *R*_f_ = 0.4 (EtOAc/hexane, 4:6); ^**1**^ **H NMR** (600 MHz, CDCl_3_) δ 8.01 (s, 1H), 7.30–7.23 (m, 3H), 7.00–6.95 (m, 2H), 6.70 (d, *J* = 3.0 Hz, 1H), 6.66 (d, *J* = 3.0 Hz, 1H), 4.21 (q, *J* = 7.1 Hz, 2H), 4.12 (t, *J* = 7.2 Hz, 2H), 2.96 (t, *J* = 7.2 Hz, 2H), 1.25 (t, *J* = 7.1 Hz, 3H). ^**13**^ **C {** ^**1**^ **H} NMR** (151 MHz, CDCl_3_) δ 163.9, 151.1, 141.7, 137.4, 128.7, 128.6, 128.4, 127.0, 123.4, 122.6, 117.4, 111.3, 59.9, 49.8, 37.9, 14.2. **HRMS** (ESI) calcd for C_18_H_19_O_3_N_2_ [M + H]^+^: 311.1390; found: 311.1390.

### Ethyl 1-Phenethyl-2-(thiazol-4-yl)-1*H*-pyrrole-3-carboxylate (**4na**)

Following the general procedure b: colorless liquid (53 mg, 85% yield). *R*_f_ = 0.5 (EtOAc/hexane, 3:7); ^**1**^ **H NMR** (600 MHz, CDCl_3_) δ 8.90 (d, *J* = 2.1 Hz, 1H), 7.48 (d, *J* = 2.1 Hz, 1H), 7.26–7.20 (m, 3H), 6.98–6.94 (m, 2H), 6.69 (d, *J* = 3.0 Hz, 1H), 6.64 (d, *J* = 3.0 Hz, 1H), 4.21 (t, *J* = 7.4 Hz, 2H), 4.18 (q, *J* = 7.1 Hz, 2H), 2.92 (t, *J* = 7.4 Hz, 2H), 1.24 (t, *J* = 7.1 Hz, 3H). ^**13**^ **C {** ^**1**^ **H} NMR** (151 MHz, CDCl_3_) δ 164.7, 151.3, 146.0, 138.1, 130.4, 128.7, 128.6, 126.6, 122.0, 121.1, 115.0, 110.7, 59.6, 49.7, 37.8, 14.3. **HRMS** (ESI) calcd for C_18_H_19_O_2_N_2_^32^ S [M + H]^+^: 327.1161; found: 327.1164.

### Ethyl 1-Phenethyl-2-(1-(pyrimidin-2-yl)­piperidin-4-yl)-1*H*-pyrrole-3-carboxylate (**4oa**)

Following the general procedure b: colorless liquid (35 mg, 73% yield). *R*_f_ = 0.4 (EtOAc/hexane, 4:6); ^**1**^ **H NMR** (600 MHz, CDCl_3_) δ 8.32 (d, *J* = 4.7 Hz, 2H), 7.32–7.29 (m, 2H), 7.27–7.23 (m, 1H), 7.09–7.06 (m, 2H), 6.60 (d, *J* = 3.1 Hz, 1H), 6.47 (t, *J* = 4.8 Hz, 1H), 6.45 (d, *J* = 3.1 Hz, 1H), 4.89 (dp, *J* = 13.3, 1.8 Hz, 2H), 4.18 (p, *J* = 7.3 Hz, 4H), 3.40–3.24 (m, 1H), 3.03 (t, *J* = 7.3 Hz, 2H), 2.86 (td, *J* = 12.9, 2.8 Hz, 2H), 2.40–2.28 (m, 2H), 1.54–1.48 (m, 2H), 1.26 (t, *J* = 7.1 Hz, 3H). ^**13**^ **C {** ^**1**^ **H} NMR** (151 MHz, CDCl_3_) δ 165.1, 161.6, 157.7, 141.1, 137.6, 128.9, 128.7, 126.9, 120.1, 112.2, 111.0, 109.3, 59.5, 49.1, 44.8, 38.4, 35.2, 29.0, 14.3. **HRMS** (ESI) calcd for C_24_H_29_O_2_N_4_ [M + H]^+^: 405.2285; found: 405.2293.

### Methyl 2-(4,4-Difluorocyclohexyl)-1-phenethyl-1*H*-pyrrole-3-carboxylate (**4pa**)

Following the general procedure b: colorless liquid (35 mg, 67% yield). *R*_f_ = 0.5 (EtOAc/hexane, 2:8); ^**1**^ **H NMR** (600 MHz, CDCl_3_) δ 7.34–7.30 (m, 2H), 7.28–7.25 (m, 1H), 7.10–7.06 (m, 2H), 6.58 (d, *J* = 3.1 Hz, 1H), 6.45 (d, *J* = 3.1 Hz, 1H), 4.16 (t, *J* = 7.2 Hz, 2H), 3.80 (s, 3H), 3.24–3.06 (m, 1H), 3.03 (t, *J* = 7.2 Hz, 2H), 2.48–2.35 (m, 2H), 2.20–2.13 (m, 2H), 1.81–1.67 (m, 2H), 1.59 (s, 3H), 1.54–1.46 (m, 2H). ^**13**^ **C {** ^**1**^ **H} NMR** (151 MHz, CDCl_3_) δ 165.4, 140.8 (d, *J* = 2.7 Hz), 137.5, 128.9, 128.7, 127.0, 124.6 (d, *J* = 241.5 Hz), 122.2 (d, *J* = 241.5 Hz), 120.3, 111.7, 111.0, 50.9, 49.1, 38.4, 34.6–33.9 (m), 26.0 (d, *J* = 10.0 Hz). ^**19**^ **F NMR** (565 MHz, CDCl_3_) δ −91.11 (d, *J* = 235.4 Hz), −102.32 (d, *J* = 236.3 Hz). **HRMS** (ESI) calcd for C_20_H_24_O_2_NF_2_ [M + H]^+^: 348.1778; found: 348.1769.

### Ethyl 2-(4,4-Difluorocyclohexyl)-1-phenethyl-1*H*-pyrrole-3-carboxylate (**4qa**)

Following the general procedure b: colorless liquid (45 mg, 70% yield). *R*_f_ = 0.5 (EtOAc/hexane, 2:8); ^**1**^ **H NMR** (600 MHz, CDCl_3_) δ 7.34–7.30 (m, 2H), 7.29–7.22 (m, 2H), 7.11–7.06 (m, 2H), 6.60 (d, *J* = 3.0 Hz, 1H), 6.46 (d, *J* = 3.1 Hz, 1H), 4.27 (q, *J* = 7.1 Hz, 2H), 4.16 (t, *J* = 7.3 Hz, 2H), 3.21–3.07 (m,1H), 3.03 (t, *J* = 7.2 Hz, 2H), 2.49–2.34 (m, 2H), 2.21–2.09 (m, 2H), 1.82–1.67 (m, 2H), 1.56–1.47 (m, 2H), 1.36 (t, *J* = 7.1 Hz, 3H). ^**13**^ **C {** ^**1**^ **H} NMR** (151 MHz, CDCl_3_) δ 165.1, 140.4 (d, *J* = 2.6 Hz), 137.6, 129.6 (d, *J* = 149.7 Hz), 128.8 (d, *J* = 23.7 Hz), 128.3–127.2 (m), 127.0, 124.7–121.1 (m), 120.3, 112.2, 111.1, 59.6, 49.2, 38.4, 34.3 (dd, *J* = 25.6, 22.5 Hz), 26.1 (d, *J* = 10.3 Hz), 14.4. ^**19**^ **F NMR** (565 MHz, CDCl_3_) δ −77.41, −91.06 (d, *J* = 235.7 Hz), −101.98 (d, *J* = 233.8 Hz). **HRMS** (ESI) calcd for C_21_H_26_O_2_NF_2_ [M + H]^+^: 362.1926; found: 362.1927.

### *tert*-Butyl 4-(3-(ethoxycarbonyl)-1-phenethyl-1*H*-pyrrol-2-yl)­piperidine-1-carboxylate (**4ra**)

Following the general procedure b: colorless liquid (31 mg, 68% yield). *R*_f_ = 0.4 (EtOAc/hexane, 3:7); ^**1**^ **H NMR** (600 MHz, CDCl_3_) δ 7.33–7.29 (m, 2H), 7.28–7.24 (m, 1H), 7.10–7.07 (m, 2H), 6.59 (d, *J* = 3.0 Hz, 1H), 6.44 (d, *J* = 3.0 Hz, 1H), 4.25 (q, *J* = 7.1 Hz, 2H), 4.21–4.18 (m, 2H), 4.15 (t, *J* = 7.3 Hz, 2H), 3.29–3.08 (m, 1H), 3.02 (t, *J* = 7.3 Hz, 2H), 2.75–2.61 (m, 2H), 2.32–2.20 (m, 2H), 1.49 (s, 9H), 1.44–1.37 (m, 2H), 1.35 (t, *J* = 7.1 Hz, 3H). ^**13**^ **C {** ^**1**^ **H} NMR** (151 MHz, CDCl_3_) δ 165.0, 154.8, 141.0, 137.6, 128.8, 128.7, 127.0, 120.2, 112.2, 111.0, 79.4, 59.5, 49.2, 38.4, 34.9, 29.1, 28.5, 14.5. **HRMS** (ESI) calcd for C_25_H_35_O_4_N_2_ [M + H]^+^: 427.2591; found: 427.2596.

### Ethyl (*R*)-2-(1-(*tert*-Butoxycarbonyl)­pyrrolidin-3-yl)-1-phenethyl-1*H*-pyrrole-3-carboxylate (**4sa**)

Following the general procedure b: colorless liquid (34 mg, 71% yield). *R*_f_ = 0.4 (EtOAc/hexane, 3:7); ^**1**^ **H NMR** (600 MHz, CDCl_3_) δ 7.33–7.28 (m, 2H), 7.28–7.24 (m, 1H), 7.07–7.02 (m, 2H), 6.62 (d, *J* = 3.0 Hz, 1H), 6.46 (d, *J* = 3.0 Hz, 1H), 4.25 (q, *J* = 7.1 Hz, 2H), 4.16 (t, *J* = 7.1 Hz, 2H), 3.72–3.64 (m, 2H), 3.56–3.48 (m, 1H), 3.30 (td, *J* = 10.7, 7.1 Hz, 1H), 3.02 (td, *J* = 7.0, 3.4 Hz, 2H), 2.66–2.57 (m, 1H), 1.75–1.69 (m, 1H), 1.48 (s, 9H), 1.34 (t, *J* = 7.1 Hz, 3H). ^**13**^ **C {** ^**1**^ **H} NMR** (151 MHz, CDCl_3_) δ 164.8, 154.4, 137.4, 136.7, 128.8, 128.7, 127.0, 120.7, 112.9, 111.3, 79.0, 59.6, 49.2, 49.0, 45.8, 38.3, 35.6, 29.6, 28.6, 14.4. **HRMS** (ESI) calcd for C_24_H_33_O_4_N_2_ [M + H]^+^: 413.2434; found: 413.2439.

### Ethyl 2-(1-(*tert*-Butoxycarbonyl)­azetidin-3-yl)-1-phenethyl-1*H*-pyrrole-3-carboxylate (**4ta**)

Following the general procedure b: colorless liquid (40 mg, 82% yield). *R*_f_ = 0.5 (EtOAc/Hexane, 3:7); ^**1**^ **H NMR** (400 MHz, CDCl_3_) δ 7.38–7.24 (m, 3H), 7.09–7.01 (m, 2H), 6.67 (d, *J* = 3.1 Hz, 1H), 6.58 (d, *J* = 3.1 Hz, 1H), 4.32 (q, *J* = 7.1 Hz, 2H), 4.21 (d, *J* = 7.8 Hz, 2H), 4.18 (d, *J* = 7.8 Hz, 2H), 4.15–4.06 (m, 1H), 4.05– 4.97 (m, 2H), 3.05 (t, *J* = 7.1 Hz, 2H), 1.52 (s, 9H), 1.39 (t, *J* = 7.1 Hz, 3H). ^**13**^ **C {** ^**1**^ **H} NMR** (101 MHz, CDCl_3_) δ 164.8, 156.8, 137.3, 136.6, 128.9, 128.7, 127.1, 120.8, 113.8, 111.3, 79.4, 59.6, 48.8, 38.4, 28.4, 24.7, 14.5. **HRMS** (ESI) calcd for C_23_H_31_O_4_N_2_ [M + H]^+^: 399.2278; found: 399.2274.

### Ethyl 2-(3-Methoxy-3-oxopropyl)-1-phenethyl-1*H*-pyrrole-3-carboxylate (**4ua**)

Following the general procedure b: colorless liquid (47 mg, 77% yield). *R*_f_ = 0.4 (EtOAc/hexane, 3:7); ^**1**^ **H NMR** (600 MHz, CDCl_3_) δ 7.34–7.28 (m, 2H), 7.28–7.23 (m, 1H), 7.10–7.06 (m, 2H), 6.56 (d, *J* = 3.1 Hz, 1H), 6.47 (d, *J* = 3.1 Hz, 1H), 4.27 (q, *J* = 7.1 Hz, 2H), 4.16 (t, *J* = 7.3 Hz, 2H), 3.67 (s, 3H), 3.09–3.05 (m, 2H), 3.01 (t, *J* = 7.3 Hz, 2H), 2.54–2.50 (m, 2H), 1.35 (t, *J* = 7.1 Hz, 3H). ^**13**^ **C {** ^**1**^ **H} NMR** (151 MHz, CDCl_3_) δ: 173.4, 165.0, 137.8, 137.7, 128.8, 128.7, 126.9, 120.1, 112.2, 110.0, 59.4, 51.6, 48.2, 38.1, 33.5, 20.3, 14.5. **HRMS** (ESI) calcd for C_19_H_24_O_4_N [M + H]^+^: 330.1699; found: 330.1706.

### Ethyl 2-(2-Fluorophenyl)-1-phenethyl-1*H*-pyrrole-3-carboxylate (**4xa**)

Following the general procedure b: colorless liquid (47 mg, 86% yield). *R*_f_ = 0.4 (EtOAc/hexane, 3:7); ^**1**^ **H NMR** (400 MHz, CDCl_3_) δ 7.38–7.28 (m, 1H), 7.17–7.11 (m, 3H), 7.11–7.04 (m, 2H), 7.00 (td, *J* = 7.5, 2.0 Hz, 1H), 6.86–6.79 (m, 2H), 6.62 (d, *J* = 3.0 Hz, 1H), 6.56 (d, *J* = 3.1 Hz, 1H), 4.01 (q, *J* = 7.1 Hz, 2H), 3.92 (dt, *J* = 14.0, 7.0 Hz, 1H), 3.79 (dt, *J* = 14.0, 7.8 Hz, 1H), 2.77 (t, *J* = 7.4 Hz, 2H), 1.02 (t, *J* = 7.1 Hz, 3H). ^**13**^ **C {** ^**1**^ **H} NMR** (101 MHz, CDCl_3_) δ 164.5, 160.5 (d, *J* = 247.0 Hz), 137.7, 133.0 (d, *J* = 2.4 Hz), 131.2, 130.6 (d, *J* = 8.2 Hz), 128.6, 128.6, 126.7, 123.7 (d, *J* = 3.7 Hz), 121.4, 119.9 (d, *J* = 16.0 Hz), 115.5 (d, *J* = 22.1 Hz), 115.0, 110.4, 59.3, 48.9, 37.6, 14.1. ^**19**^ **F NMR** (376 MHz, CDCl_3_) δ −112.69 (ddd, *J* = 9.7, 7.1, and 5.2 Hz). **HRMS** (ESI) calcd for C_21_H_21_O_2_NF [M + H]^+^: 338.1550; found: 338.1551.

### 2-(3-Fluorophenyl)-1-phenethyl-1*H*-pyrrole-3-carboxylic acid (**5aa**)

Following the general procedure e­(i): white solid (398 mg, 87% yield). *R*_f_ = 0.4 (EtOAc/hexane, 6:4); ^**1**^ **H NMR** (400 MHz, CDCl_3_) δ 7.22 (td, *J* = 8.0, 5.9 Hz, 1H), 7.16–7.10 (m, 3H), 6.99 (tdd, *J* = 8.5, 2.6, 1.0 Hz, 1H), 6.80–6.72 (m, 3H), 6.66–6.62 (m, 1H), 6.61 (d, *J* = 3.1 Hz, 1H), 6.56 (d, *J* = 3.1 Hz, 1H), 3.83 (t, *J* = 7.1 Hz, 2H), 2.75 (t, *J* = 7.1 Hz, 2H). ^**13**^ **C {** ^**1**^ **H} NMR** (101 MHz, CDCl_3_) δ 169.7, 162.2 (d, *J* = 246.4 Hz), 137.8 (d, *J* = 2.2 Hz), 137.4, 133.4 (d, *J* = 8.4 Hz), 129.4 (d, *J* = 8.4 Hz), 128.7, 128.6, 126.9, 126.4 (d, *J* = 2.9 Hz), 121.1, 117.8 (d, *J* = 21.8 Hz), 115.4 (d, *J* = 20.9 Hz), 112.9, 111.2, 48.7, 37.7. ^**19**^ **F NMR** (376 MHz, CDCl_3_) δ −113.27 (td, *J* = 9.1, 5.9 Hz). **HRMS** (ESI) calcd for C_19_H_17_O_2_NF [M + H]^+^: 310.1237; found: 310.1243.

### *N*-Allyl-2-(3-fluorophenyl)-1-phenethyl-1*H*-pyrrole-3-carboxamide (**5aa’**)

Following the general procedure e­(ii): white solid (100 mg, 89% yield). *R*_f_ = 0.6 (EtOAc/hexane, 6:4); ^**1**^ **H NMR** (400 MHz, CDCl_3_) δ 7.30 (ddd, *J* = 8.4, 7.6, 5.9 Hz, 1H), 7.17–7.09 (m, 3H), 7.04 (tdd, *J* = 8.5, 2.6, 1.0 Hz, 1H), 6.87–6.83 (m, 1H), 6.80–6.74 (m, 2H), 6.67 (ddd, *J* = 9.3, 2.6, 1.5 Hz, 1H), 6.61 (d, *J* = 3.0 Hz, 1H), 6.57 (d, *J* = 3.0 Hz, 1H), 5.62 (ddt, *J* = 17.1, 10.3, 5.5 Hz, 1H), 5.22–5.11 (m, 1H), 4.90 (dq, *J* = 10.4, 1.5 Hz, 1H), 4.83 (dq, *J* = 17.2, 1.7 Hz, 1H), 3.83 (t, *J* = 7.1 Hz, 2H), 3.75 (tt, *J* = 5.6, 1.6 Hz, 2H), 2.77 (t, *J* = 7.1 Hz, 2H). ^**13**^ **C {** ^**1**^ **H} NMR** (101 MHz, CDCl_3_) δ 164.3, 162.5 (d, *J* = 248.4 Hz), 137.6, 134.5, 133.7 (d, *J* = 8.1 Hz), 132.5 (d, *J* = 2.2 Hz), 130.3 (d, *J* = 8.5 Hz), 128.7, 128.7, 126.8, 126.7 (d, *J* = 3.1 Hz), 120.8, 118.2, 117.9 (d, *J* = 15.0 Hz), 116.0 (d, *J* = 20.9 Hz), 115.5, 109.3, 48.6, 41.6, 37.8. ^**19**^ **F NMR** (376 MHz, CDCl_3_) δ −111.74 (td, *J* = 8.9, 5.9 Hz). **HRMS** (ESI) calcd for C_22_H_22_ON_2_F [M + H]^+^: 349.1710; found: 349.1713.

## Supplementary Material



## Data Availability

The data underlying this study are available in the published article and its Supporting Information.

## Notes

Experimental procedure, mechanistic studies, and characterization of new compounds (^1^H, ^13^C NMR spectra) (PDF)
