# Supplementary material for: Regioselective Synthesis of 1,2,3-Trisubstituted Pyrroles via Addition–Cyclization of Crotonate-Derived Sulfonium Salts with a Carboxylic Acid and an Amine
Source: J Org Chem. 2026 Mar 2;91(10):3776–88. doi: 10.1021/acs.joc.5c03146 (PMC12993846; doi:10.1021/acs.joc.5c03146)
Supplement: Supplementary file 1 [file jo5c03146_si_001.pdf]

Supporting Information

Regioselective Synthesis of 1,2,3,-Trisubstituted Pyrroles via Addition−Cyclization of Crotonate-Derived Sulfonium Salts with a Carboxylic Acid and an Amine

Lahu N. Chavan,[1*] Gouthami Pashikanti,[1*] Mark M. Goodman[2] and Lanny S. Liebeskind[1]

[1]Department of Chemistry, Emory University, 1515 Dickey Drive, Atlanta, Georgia 30322.
[2]Department of Radiology and Imaging Sciences, 101 Woodruff Circle, Atlanta, GA, 30322.
*E-mail: lchavan@emory.edu

Pages

1. Experimental Procedures S-03 to S-09

2. Chiral HPLC spectral data S-10 to S-11

3. ^{1}H NMR, ^{13}C NMR, ^{19}F NMR spectra S-12 to S-78

References S-79

Experimental Section

General information: All solvents were purchased from Fisher Scientific or Sigma-Aldrich and dried over 4 Å molecular sieves (8−12 mesh, Sigma-Aldrich). Unless otherwise noted, all commercially available reagents and substrates were used directly as received. Thin layer chromatography was performed on Merck silica gel plates and visualized by UV light and/or potassium permanganate. ^{1}H, ^{13}C, and ^{19}F NMR spectra were recorded on Bruker 300, Varian INOVA 600, INOVA 500 and INOVA 400 spectrometers. Residual solvent resonances were treated as internal reference signals. ^{19}F spectra were referenced to either trifluoroacetic acid (−76.55 ppm) or fluorobenzene (−113.15 ppm). Chemical shifts (δ) are reported in ppm, using the residual solvent peak in $CDCl_3$ (H δ = 7.26 and C δ = 77.16 ppm) as an internal standard, and coupling constants (J) are given in Hz. HRMS were recorded using ESI-TOF techniques at Emory University. IR spectra were recorded on a Nicolet iS10 FT-IR spectrometer, and the absorption peaks were reported in cm^{-1}. The purification of products was performed via flash chromatography unless otherwise noted. High resolution mass spectra were obtained from the Emory University Mass Spec Facility Inc. All solvents were dried before use following the standard procedures. Reactions were monitored using thin-layer chromatography (SiO_2). TLC plates were visualized with UV light (254 nm), iodine treatment, or using ninhydrin stain. Column chromatography was carried out using silica gel (60−120 mesh and 100−200 mesh) packed in glass columns.

1. Experimental Procedures:

a) General Procedure for the synthesis of Tf-Pyridinium Reagents[1]

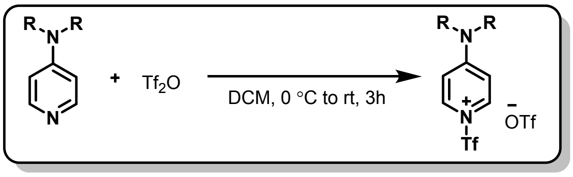

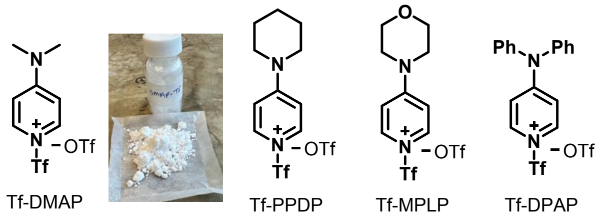

Tf-DMAP Tf-PPDP Tf-MPLP Tf-DPAP

Synthesis of Tf-DMAP, Tf-DPAP and Tf-MPLP carried out according to literature precedent.[1] Into a solution of DMAP (6.1 g, 50 mmol) in DCM (80 mL) was added $(CF_3SO_2)_2O$ (10 mL, 60 mmol, 1.2 equiv) dropwise at 0 °C. After addition, the mixture was warmed to room temperature and stirred for 3 h. The crude product was precipitated from the solution. After filtration, the solid was washed with DCM (40 mL × 4), then dried under reduced pressure to

give the pure product as a white powder (19 g, 94%). The synthesis of Tf-DPAP and Tf-MPLP were similar to that of Tf-DMAP.

b) Time optimization study:

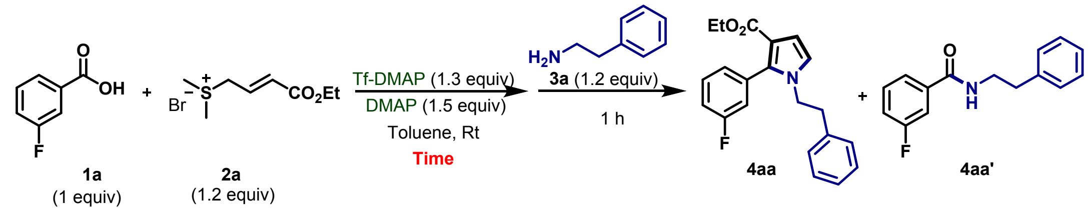

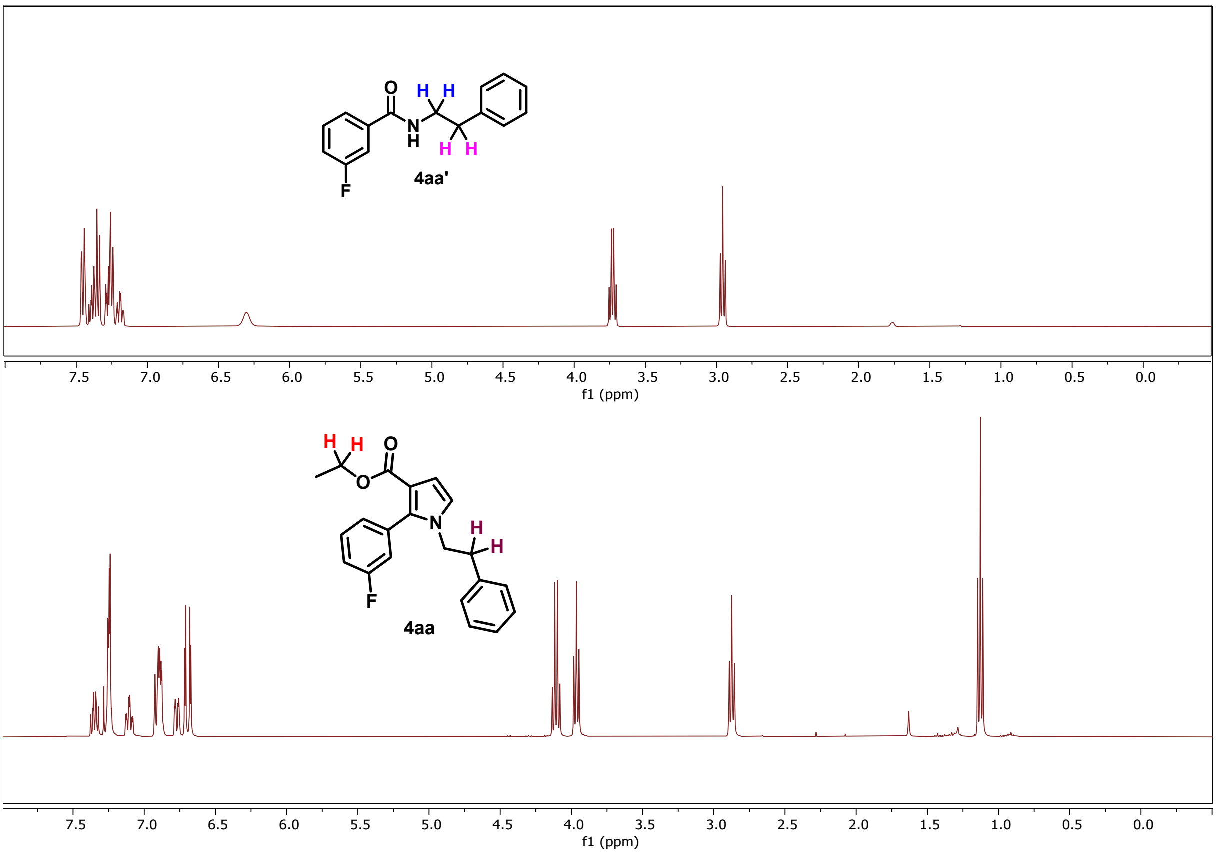

Figure S1. ^{1}H NMR comparison of **4aa** with **4aa'**

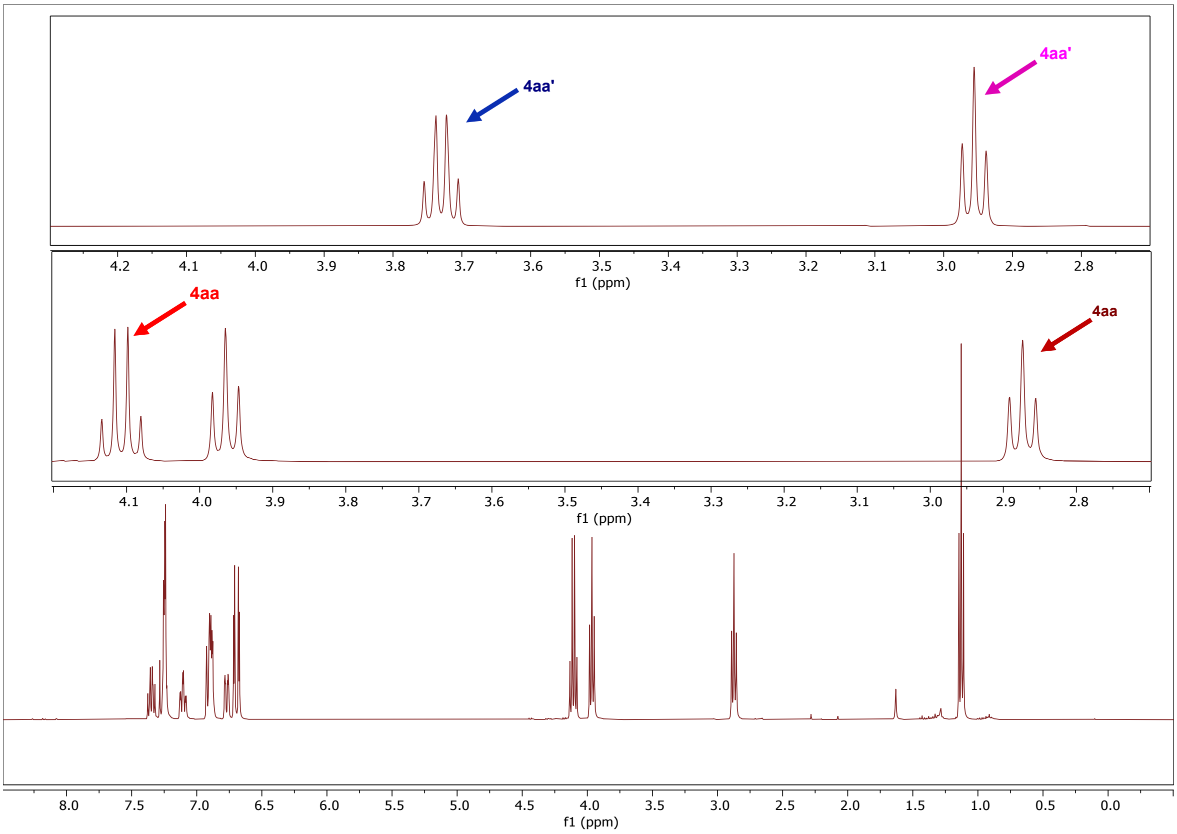

Figure S2. Highlighted ^{1}H NMR peaks of **4aa** with **4aa’**

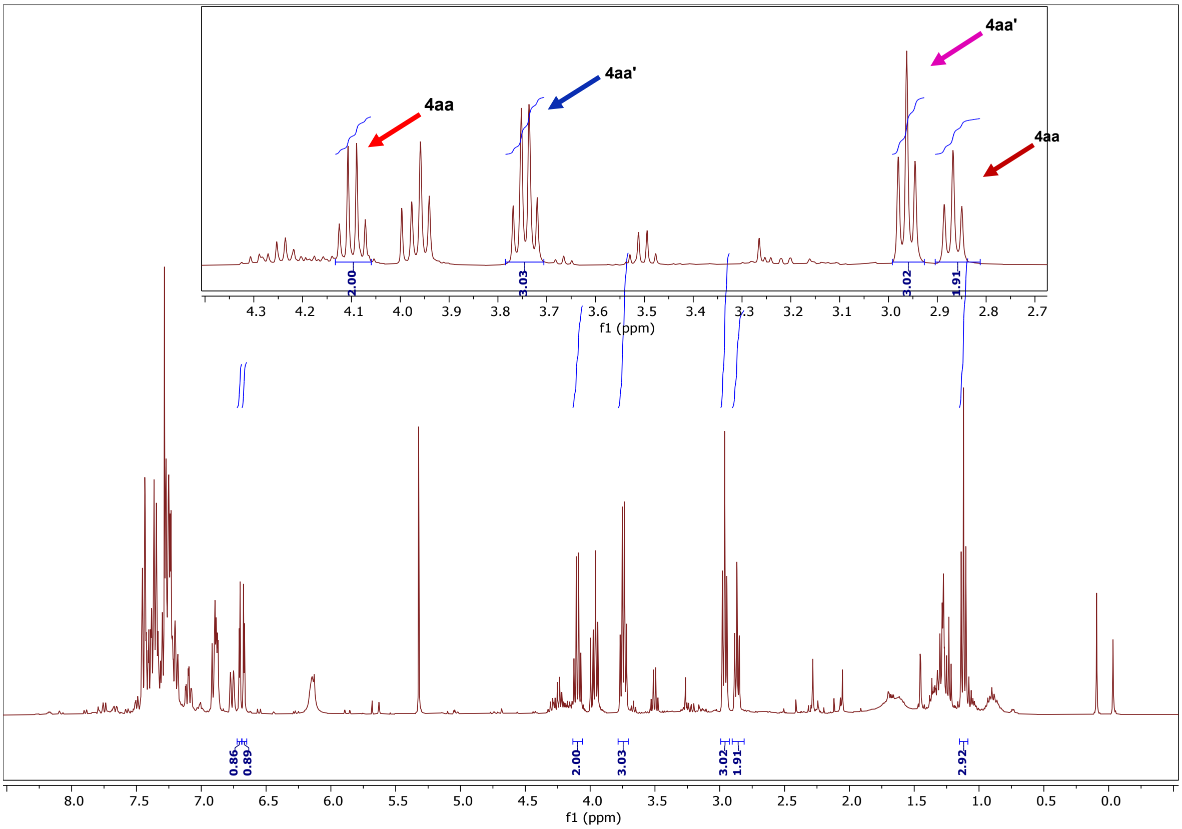

Figure S3. Crude ^{1}H NMR peaks after 5 minutes of **4aa** with **4aa’** shown 40:60%

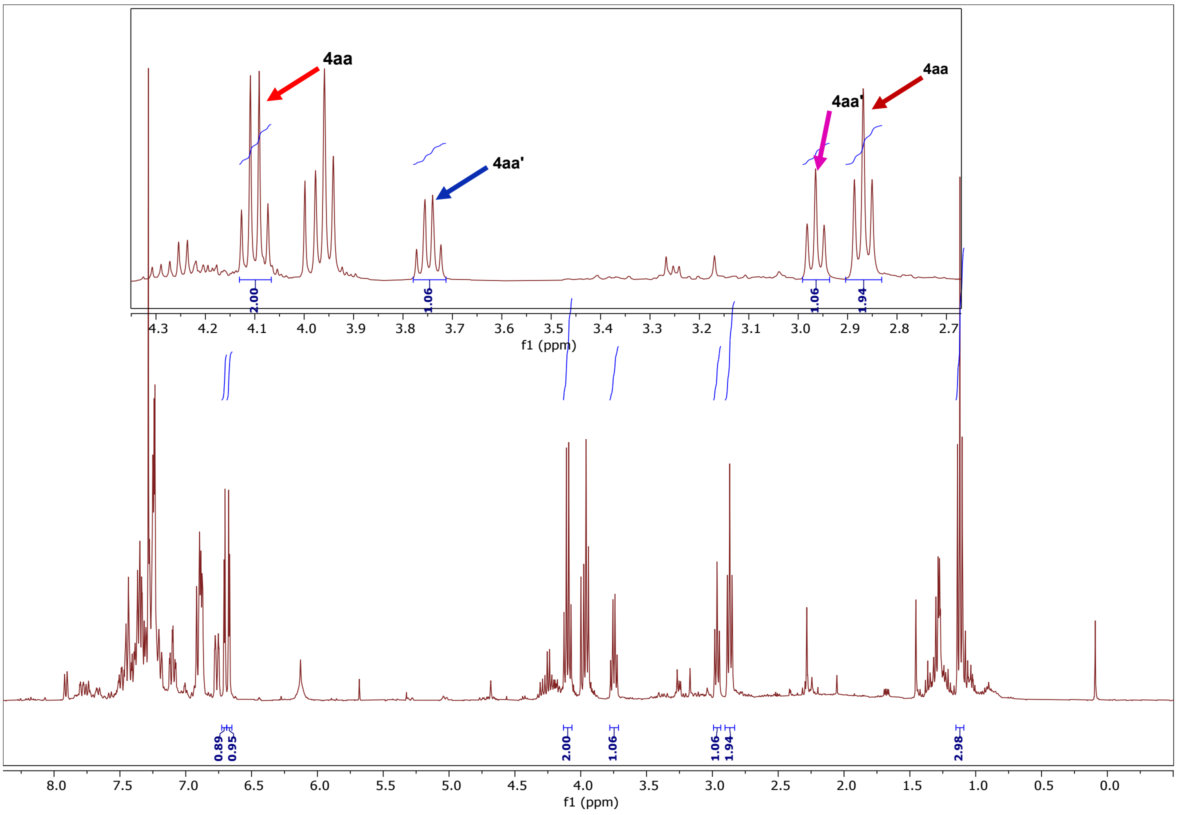

Figure S4. Crude ^{1}H NMR peaks after 15 minutes of **4aa** with **4aa’** shown 66:34%

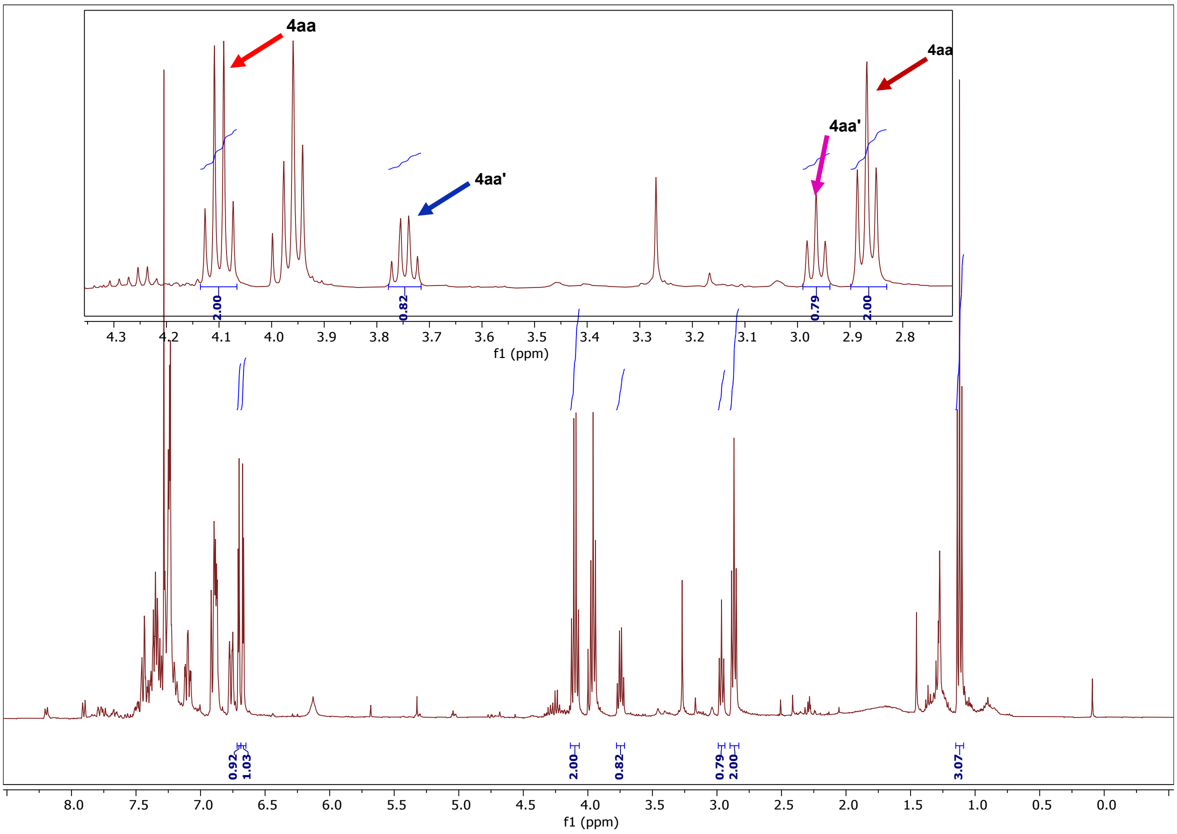

Figure S5. Crude ^{1}H NMR peaks after 25 minutes of **4aa** with **4aa’** shown 71:29%

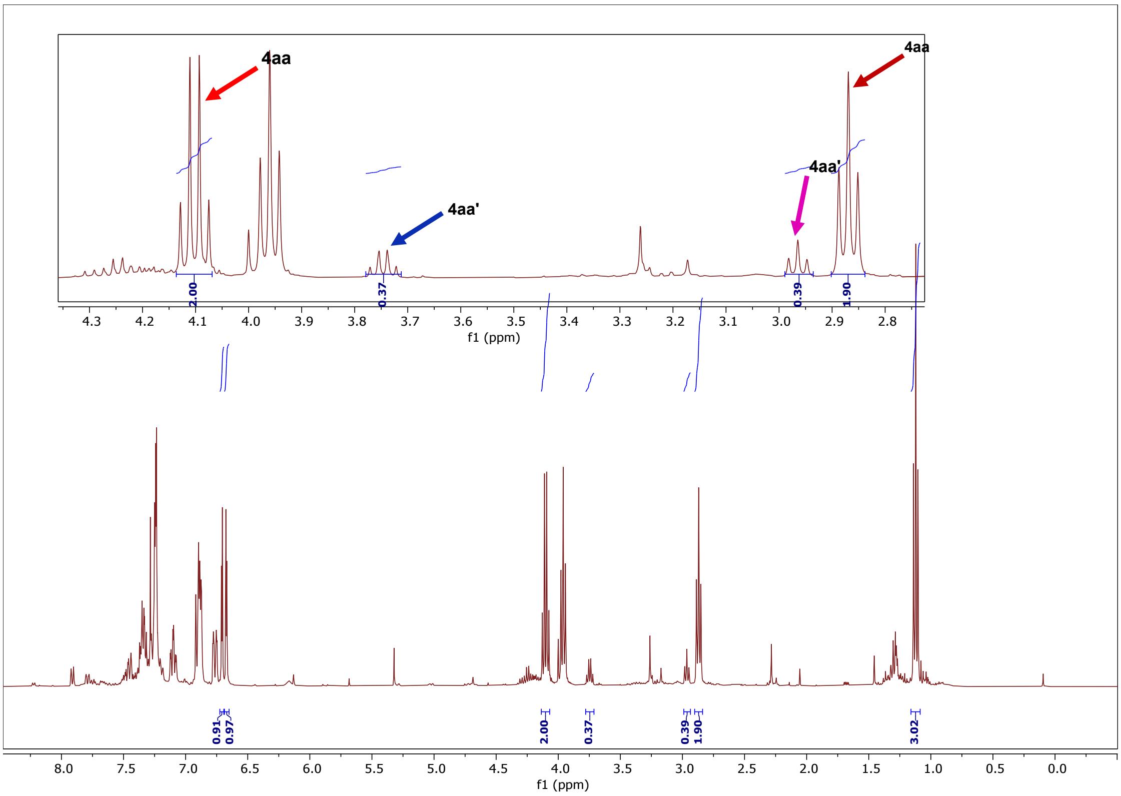

Figure S6. Crude ^{1}H NMR peaks after 35 minutes of **4aa** with **4aa'** shown 84:16%

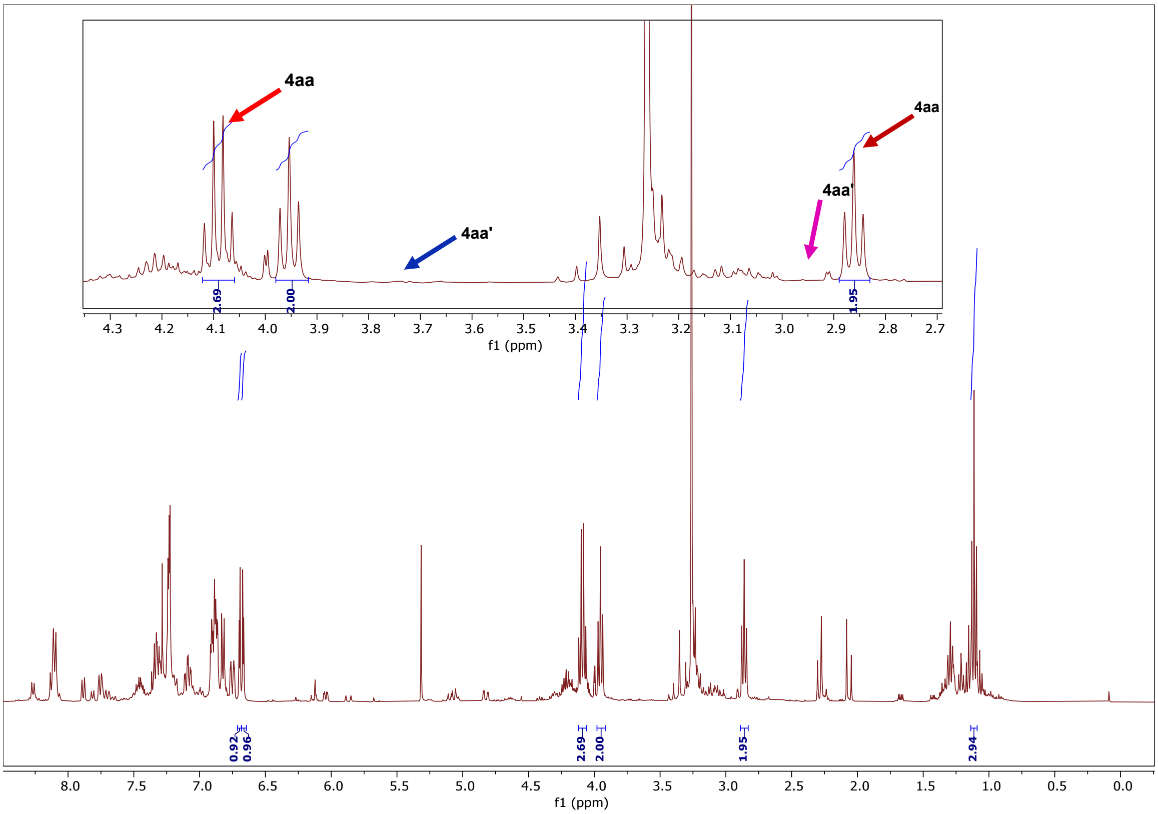

Figure S6. Crude ^{1}H NMR peaks after 45 minutes of **4aa** with **4aa'** shown 100%:00

c) Controle experiments:

i)

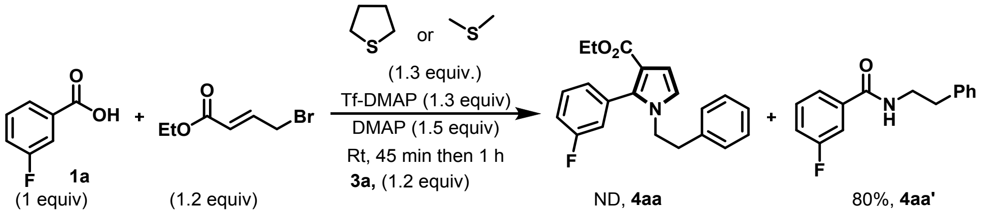

To a screw-capped seal round-bottomed flask with a Teflon magnetic stirring were added 3-fluorobenzoic acid **1a** (30 mg, 0.21 mmol, 1.0 equiv), ethyl (*E*)-4-bromobut-2-enoate (50 mg, 0.26 mmol, 1.2 equiv), tetrahydrothiophene or dimethylsulfide (0.27 mmol, 1.3 equiv), DMAP-Tf (115 mg, 0.27 mmol, 1.3 equiv), DMAP (37 mg, 0.31 mmol, 1.5 equiv) and toluene (~ 2 mL) under a dry nitrogen atmosphere. The reaction mixture was stirred for 45 min at room temperature then amine **3a** (30 mg, 0.25 mmol, 1.2 equiv) was added into the reaction mixture and the reaction continued for another 60 min. The filtrate was concentrated under reduced pressure, and the residue was purified by column chromatography on silica gel (*n*-hexane/EtOAc) to give the desired product (**4aa'**, 80%) yield.

ii)

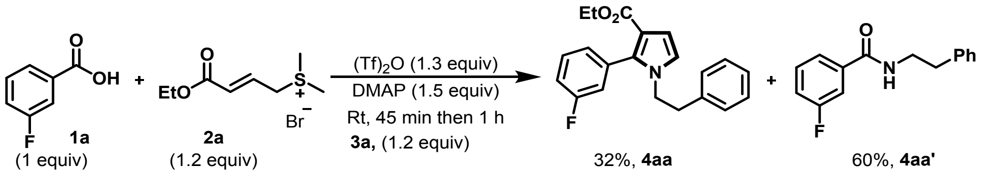

To a screw-capped seal round-bottomed flask with a Teflon magnetic stirring were added $(Tf)_2O$ (0.27 mmol, 1.3 equiv) slowly under a dry nitrogen atmosphere at 0 °C containing DMAP (37 mg, 0.31 mmol, 1.5 equiv) in toluene (~ 2 mL). The reaction mixture was stirred for 5 min at room temperature then 3-fluorobenzoic acid **1a** (30 mg, 0.21 mmol, 1.0 equiv), **2a** (50 mg, 0.26 mmol, 1.2 equiv). The reaction mixture was stirred for 45 min at room temperature then amine **3a** (30 mg, 0.25 mmol, 1.2 equiv) was added into the reaction mixture and the reaction continued for another 60 min. The filtrate was concentrated under reduced pressure, and the residue was purified by column chromatography on silica gel (*n*-hexane/EtOAc) to give the desired product (**4aa**, 32% **and 4aa'**, 60%) yield.

iii)

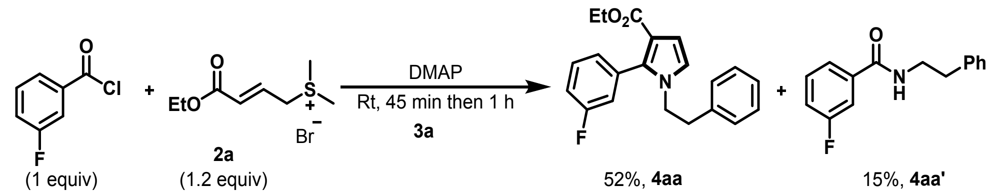

To a screw-capped seal round-bottomed flask with a Teflon magnetic stirring were added 3-fluorobenzoyl chloride (30 mg, 0.21 mmol, 1.0 equiv), sulfonium salt **2a** (0.27 mmol, 1.2 equiv), DMAP (0.32 mmol, 1.5 equiv), and solvent toluene (0.5 mL) under a dry nitrogen atmosphere. The reaction mixture was stirred for 45 min at room temperature then amine **3a** (30 mg, 0.25 mmol, 1.2 equiv) was added into the reaction mixture and the reaction continued for another 60 min. The filtrate was concentrated under reduced pressure, and the residue was purified by column chromatography on silica gel (*n*-hexane/EtOAc) to give the desired product (**4aa**, 52% and **4aa'**, 15%) yield.

iv)

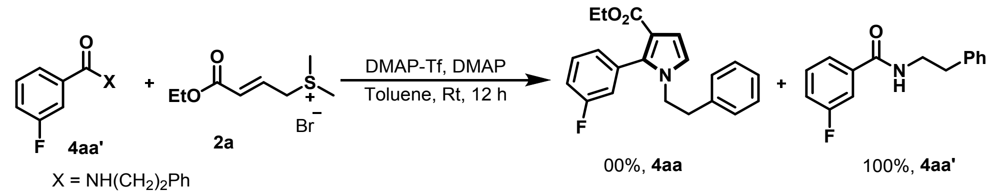

To a screw-capped seal round-bottomed flask with a Teflon magnetic stirring were added Amide **4aa'** (30 mg, 0.21 mmol, 1.0 equiv), sulfonium salt **2a** (0.27 mmol, 1.2 equiv), DMAP-Tf (115 mg, 0.27 mmol, 1.3 equiv), DMAP (0.32 mmol, 1.5 equiv), and solvent toluene (1 mL) under a dry nitrogen atmosphere. The reaction mixture was stirred overnight at room temperature. The filtrate was concentrated under reduced pressure, and the residue was purified by column chromatography on silica gel (*n*-hexane/EtOAc) to give the desired product (**4aa'**, 100%) yield.

2. Chiral HPLC Analysis:

Chiral HPLC method: C:\Users\Public\Documents\ChemStation\1\Data\IA_NP_Data\2025-09-04 17-05-20 5% IPA_IA_30min\5_IPA_IA_FIXED_1.0mL-min_30min.M (Sequence Method)

Wavelengths: 210nm, 230nm, 254nm, 280nm

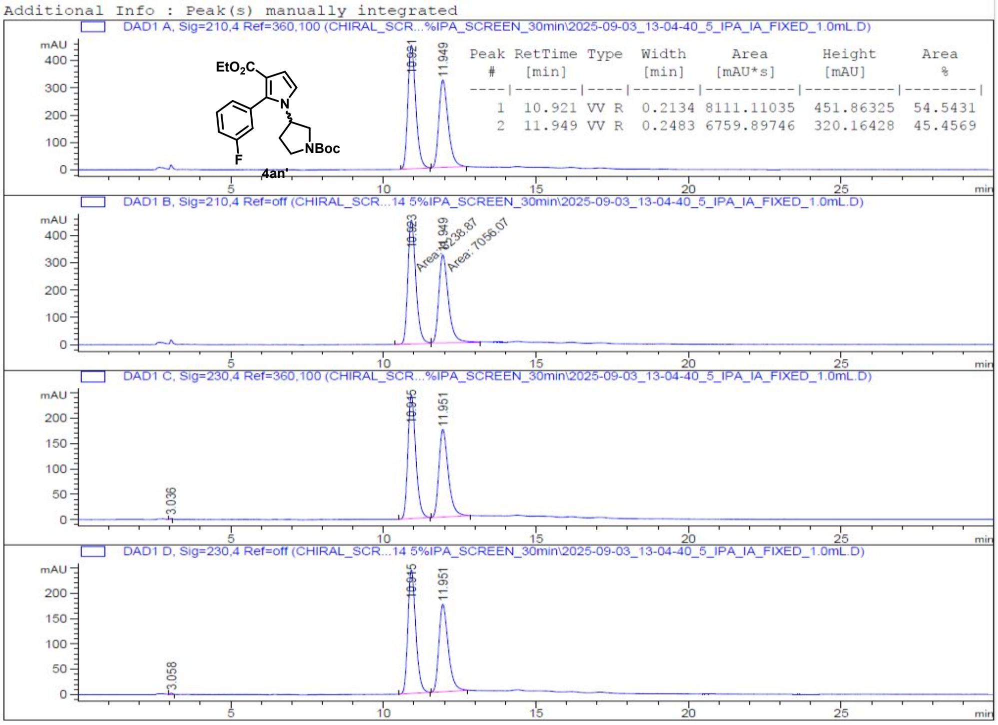

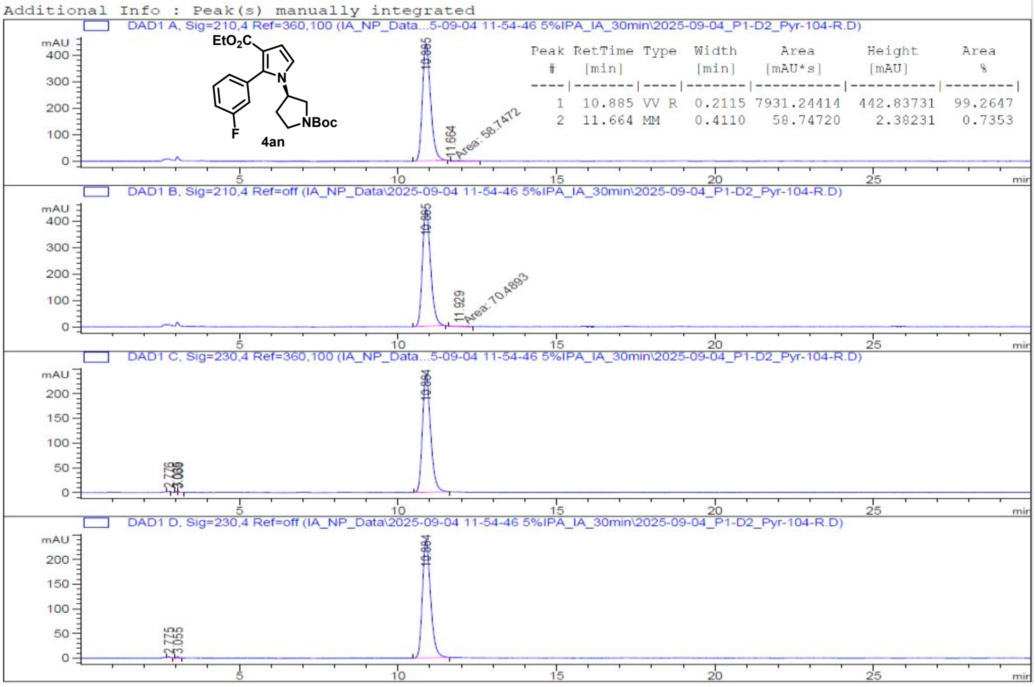

Figure S7. Chiral HPLC for racemic **4an'** and enantiomer **4an**

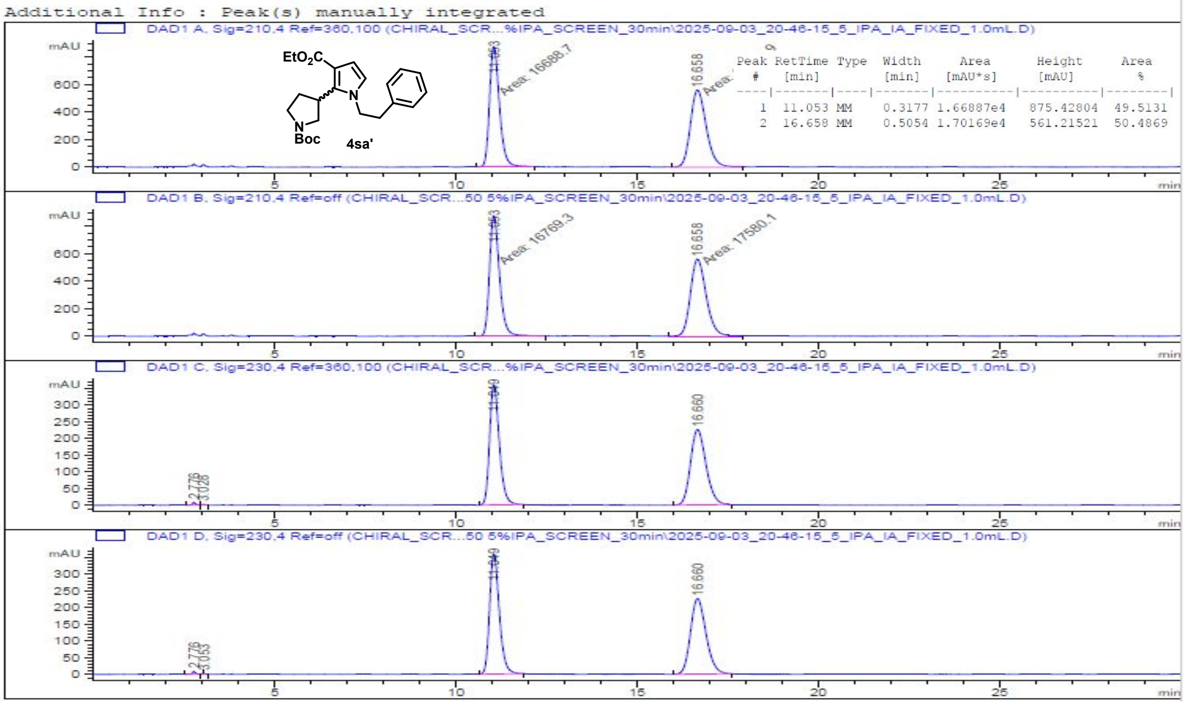

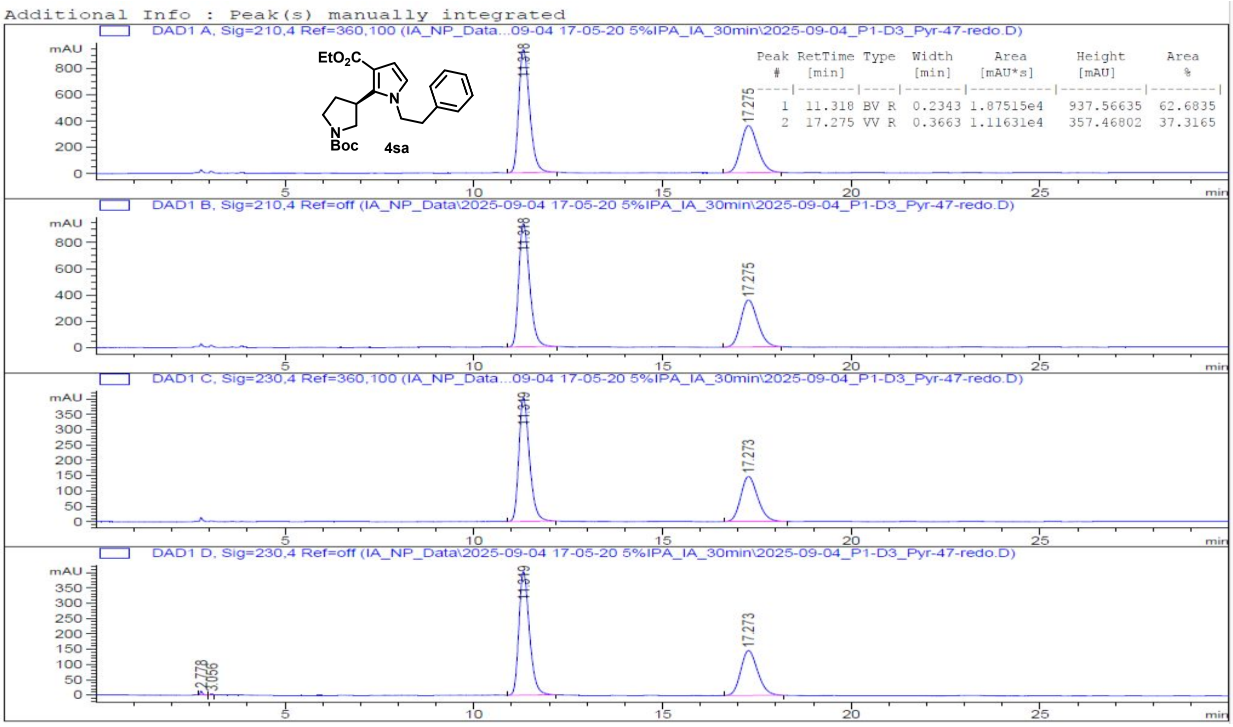

Figure S7. Chiral HPLC for racemic **4sa’** and enantiomer **4sa**

3. ^{1}H NMR, ^{13}C NMR spectra:

Ethyl 2-(3-fluorophenyl)-1-phenethyl-1H-pyrrole-3-carboxylate (4aa):

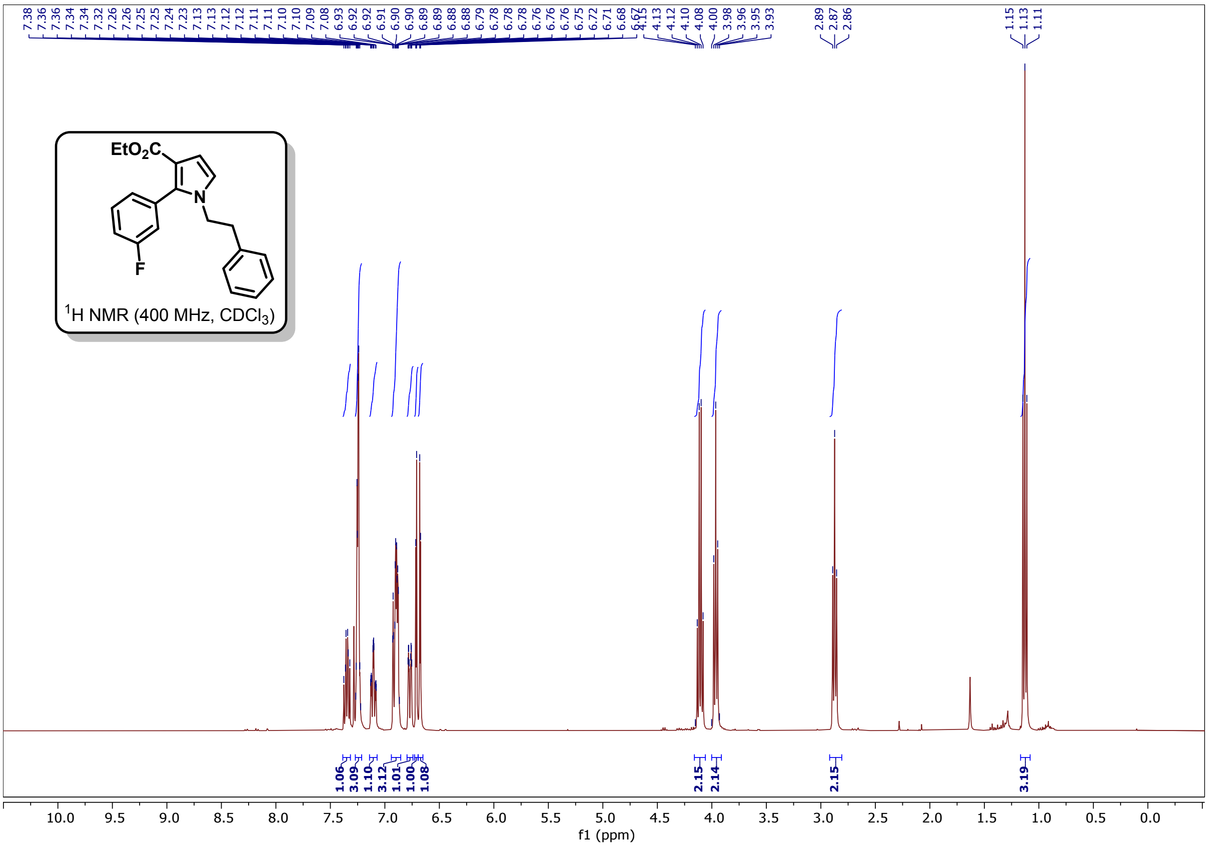

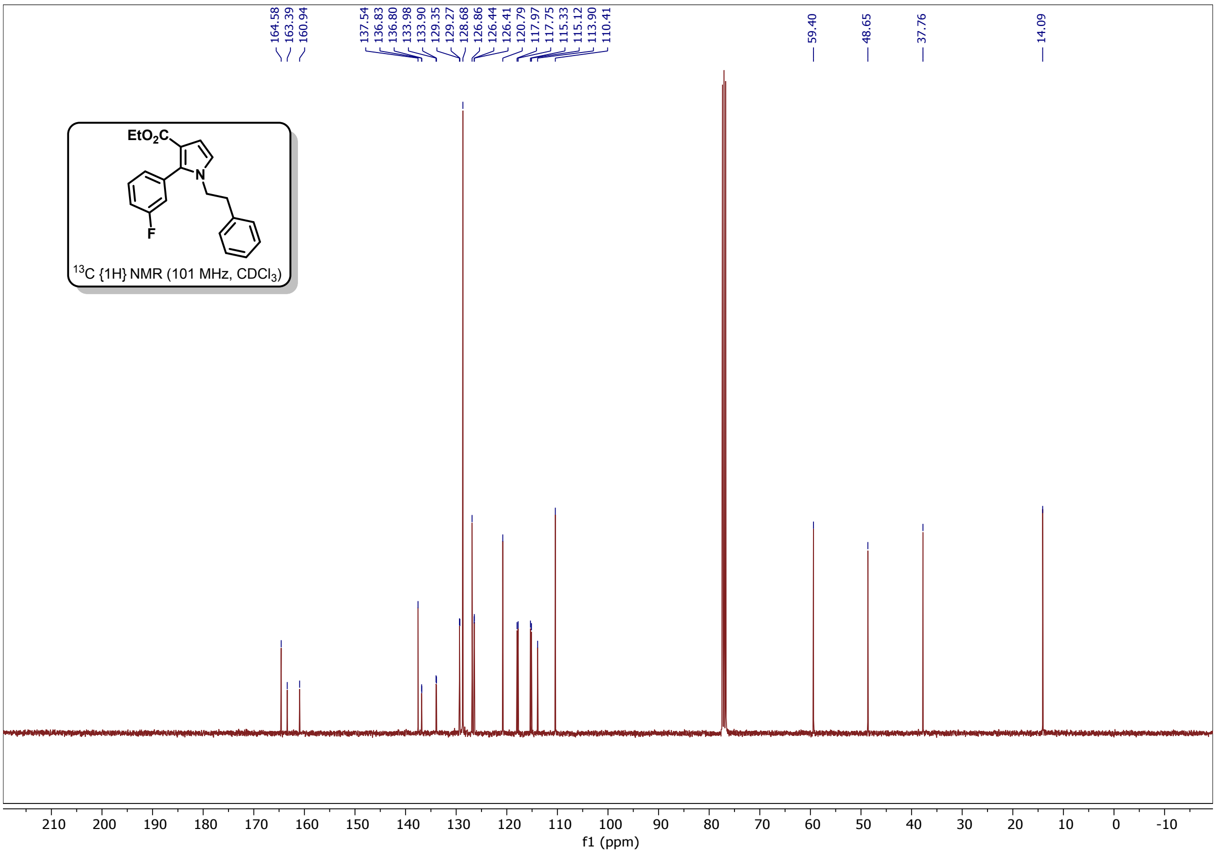

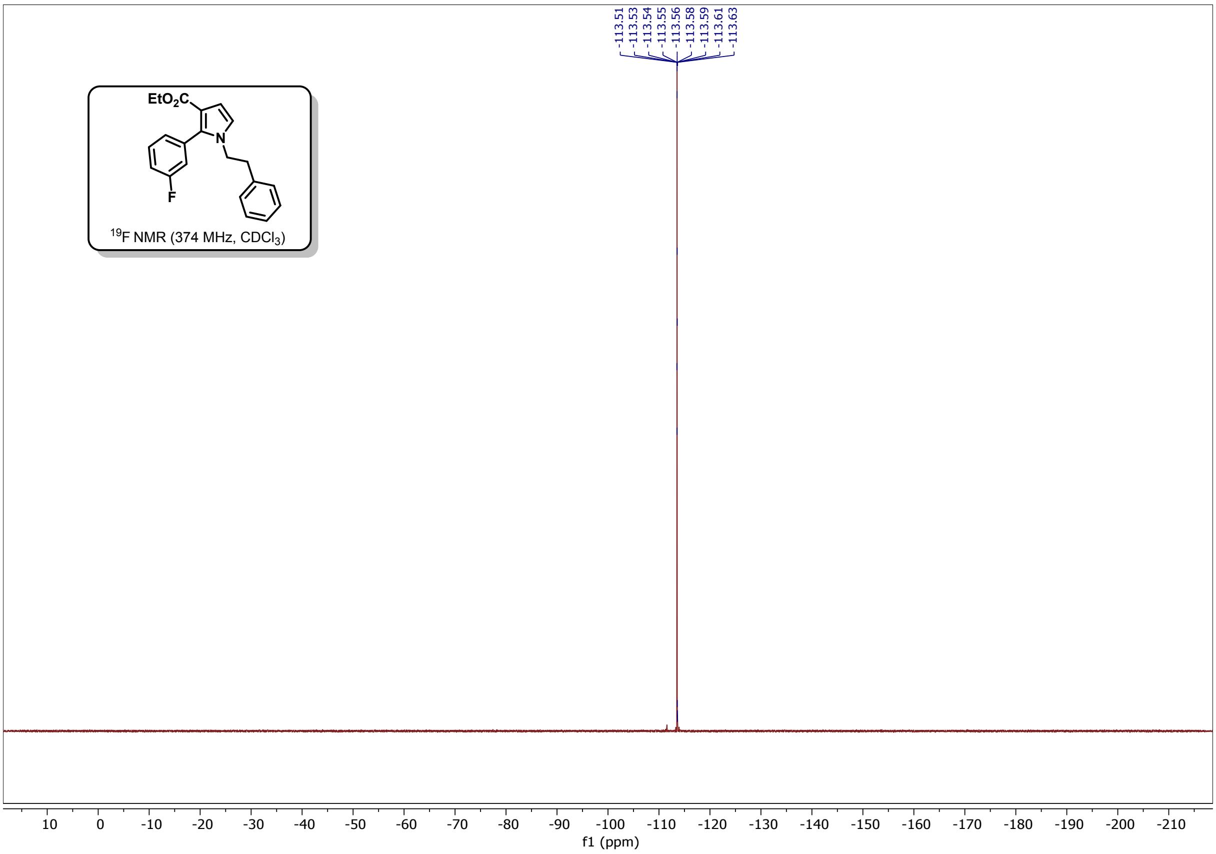
-113.51
-113.53
-113.54
-113.55
-113.56
-113.58
-113.59
-113.61
-113.63
EtO2C
N
F
19F NMR (374 MHz, CDCl3)
10 0 -10 -20 -30 -40 -50 -60 -70 -80 -90 -100 -110 -120 -130 -140 -150 -160 -170 -180 -190 -200 -210
f1 (ppm)

Ethyl 2-(3-fluorophenyl)-1-(3-methoxyphenethyl)-1H-pyrrole-3-carboxylate (4ab):

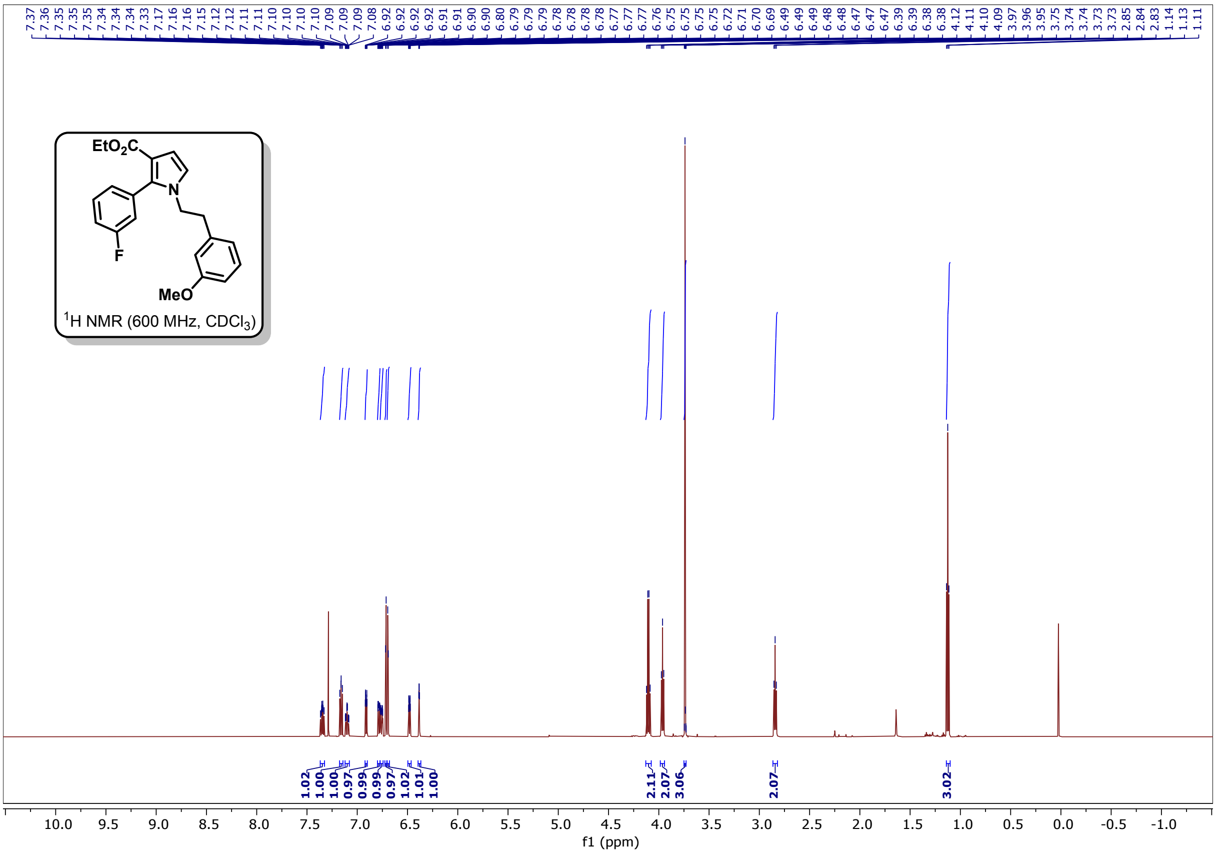

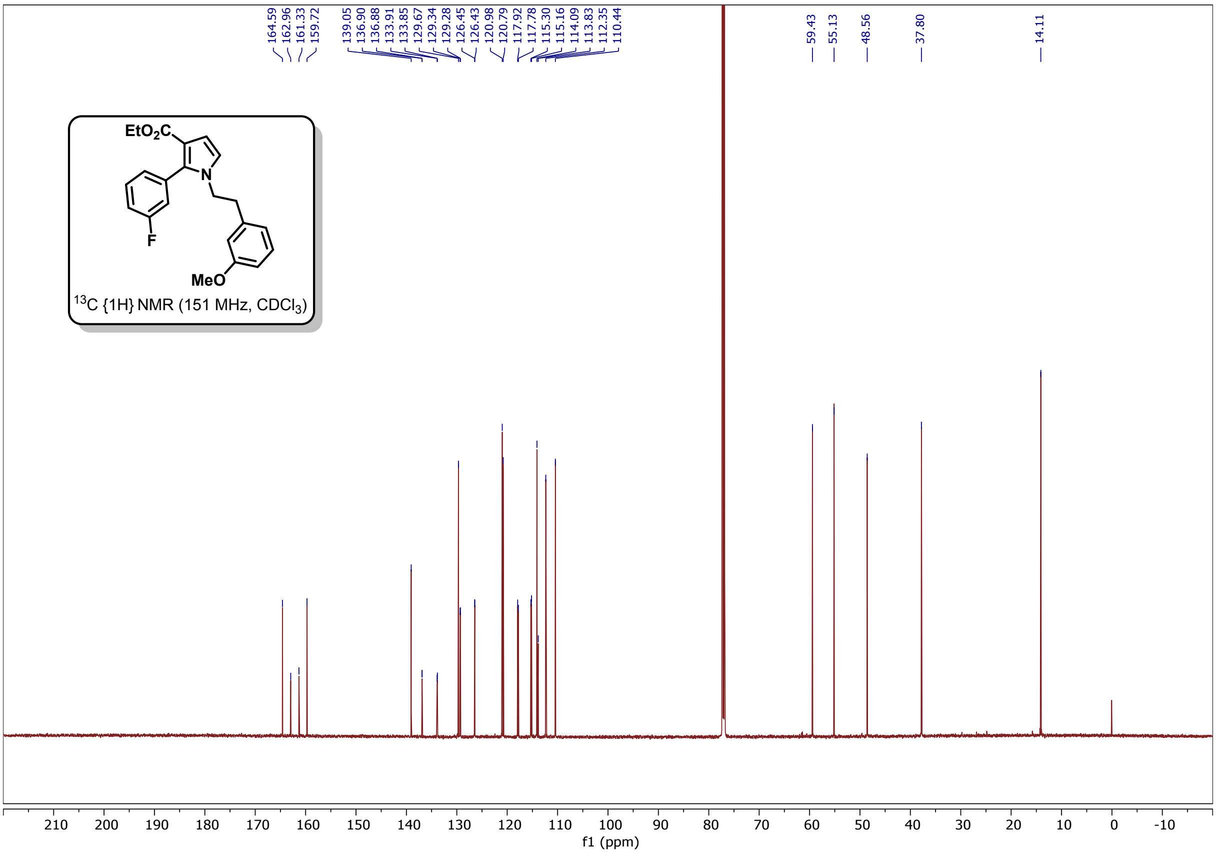

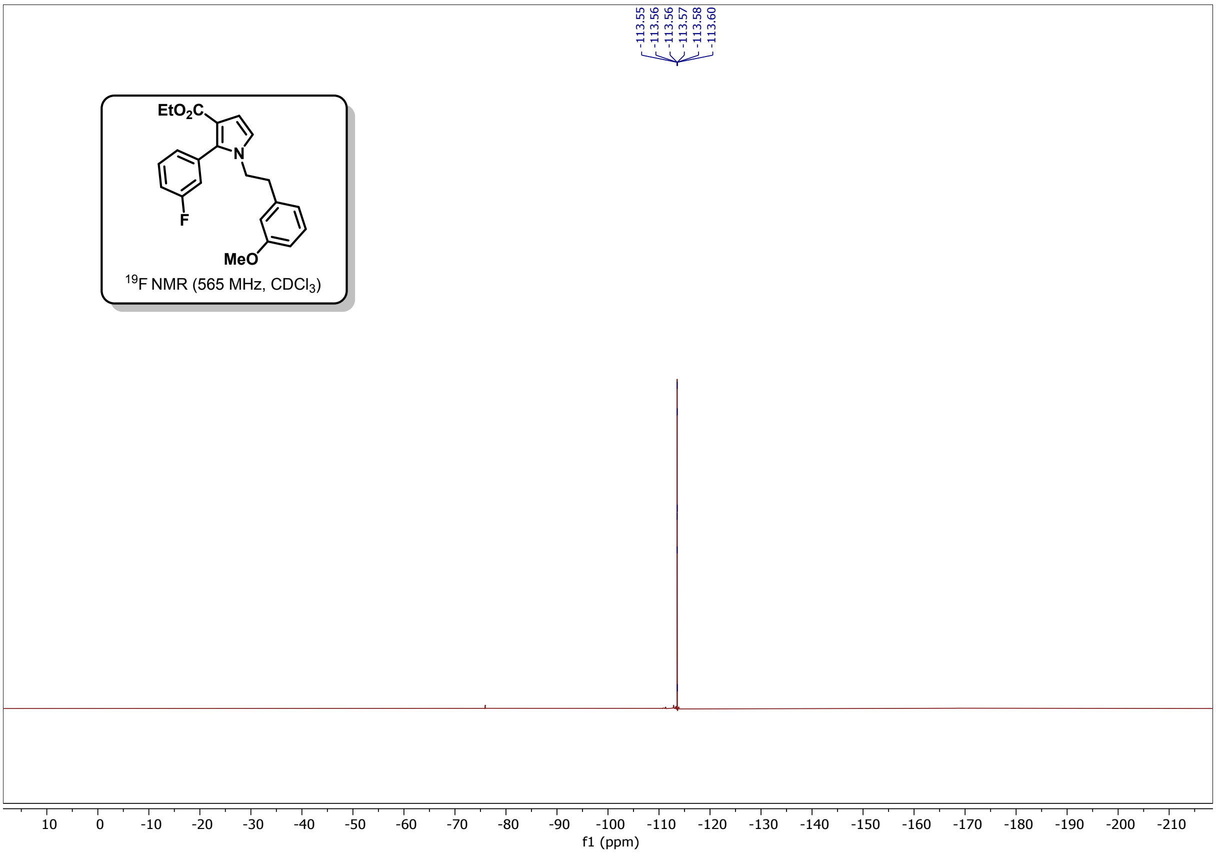
-113.55
-113.56
-113.56
-113.57
-113.58
-113.60
EtO2C
N
F
MeO
19F NMR (565 MHz, CDCl3)
10 0 -10 -20 -30 -40 -50 -60 -70 -80 -90 -100 -110 -120 -130 -140 -150 -160 -170 -180 -190 -200 -210
f1 (ppm)

Ethyl 1-(3,4-dichlorophenethyl)-2-(3-fluorophenyl)-1H-pyrrole-3-carboxylate (4ac):

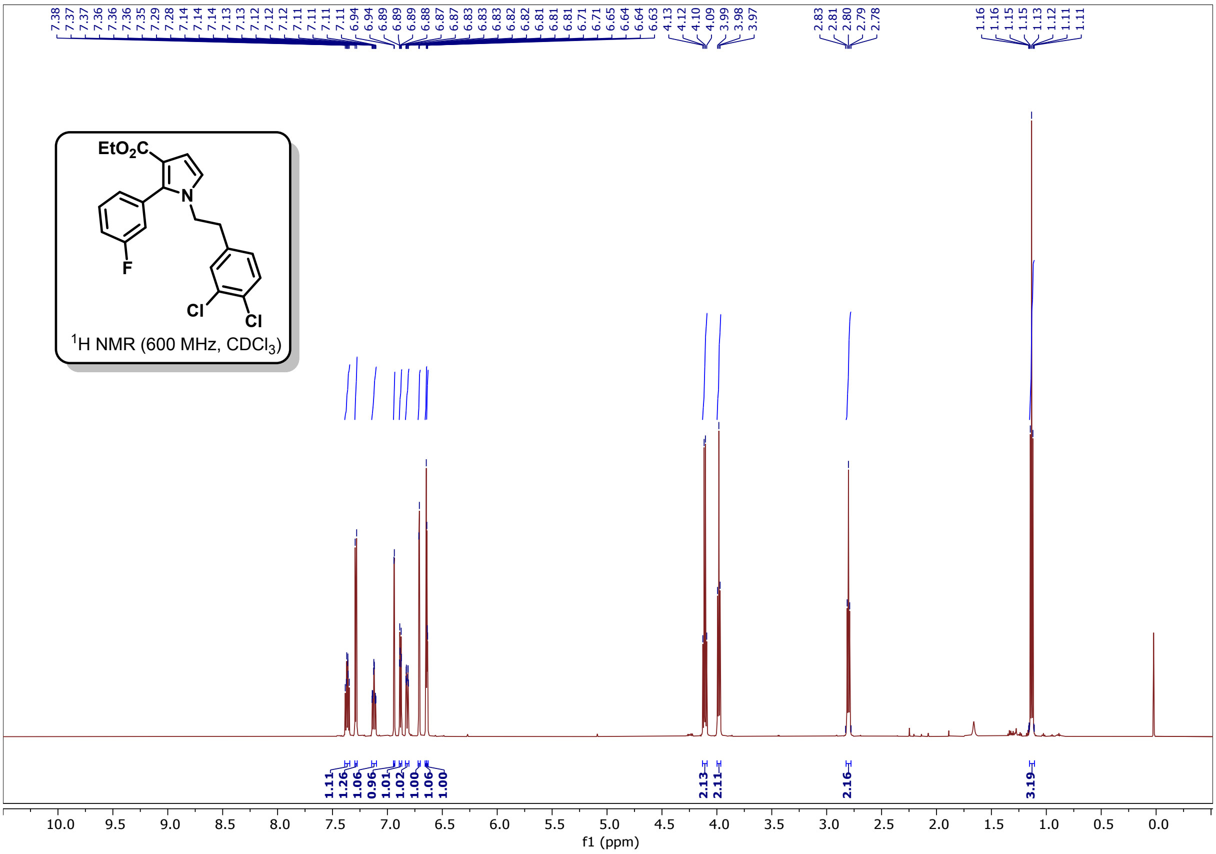

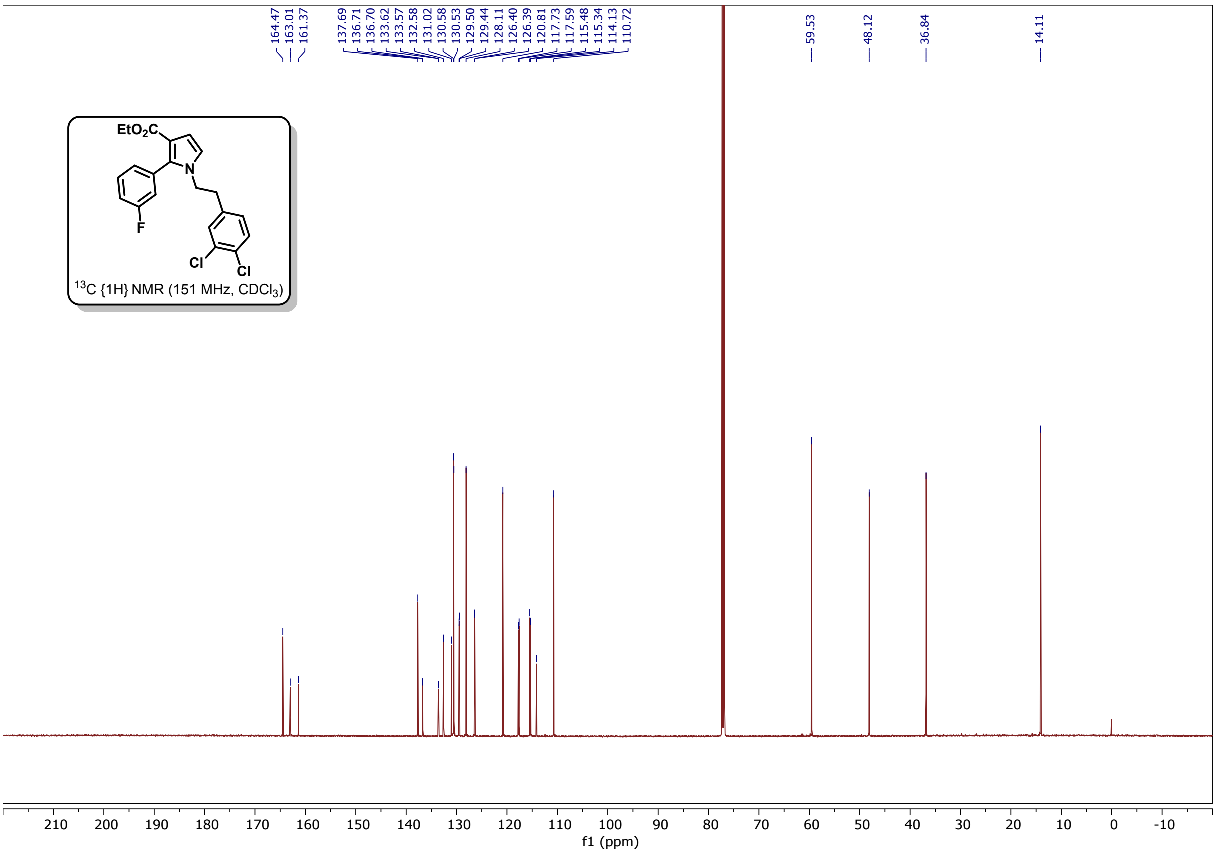

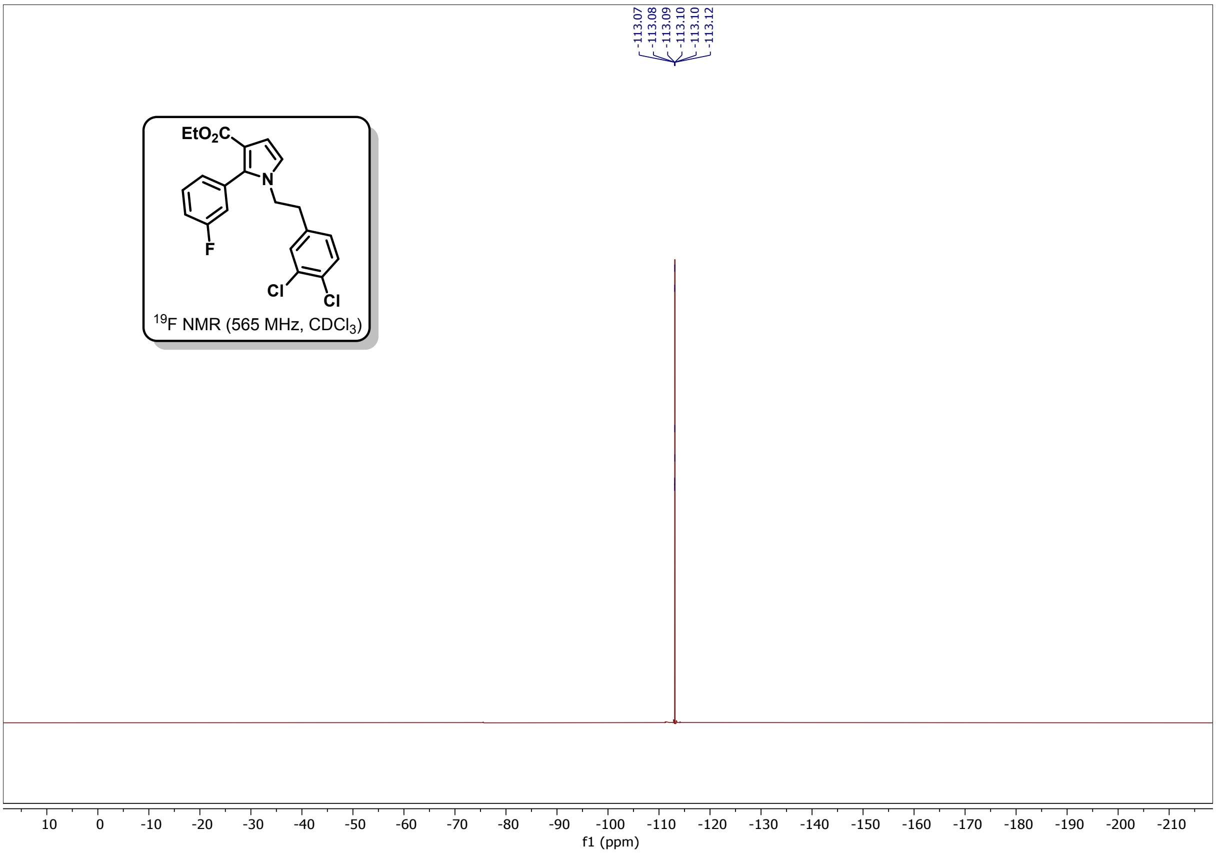

-113.07
-113.08
-113.09
-113.10
-113.10
-113.12
EtO2C
N
F
Cl
Cl
19F NMR (565 MHz, CDCl3)
10 0 -10 -20 -30 -40 -50 -60 -70 -80 -90 -100 -110 -120 -130 -140 -150 -160 -170 -180 -190 -200 -210
f1 (ppm)

Ethyl 2-(3-fluorophenyl)-1-(2-(pyridin-3-yl)ethyl)-1H-pyrrole-3-carboxylate (4ad):

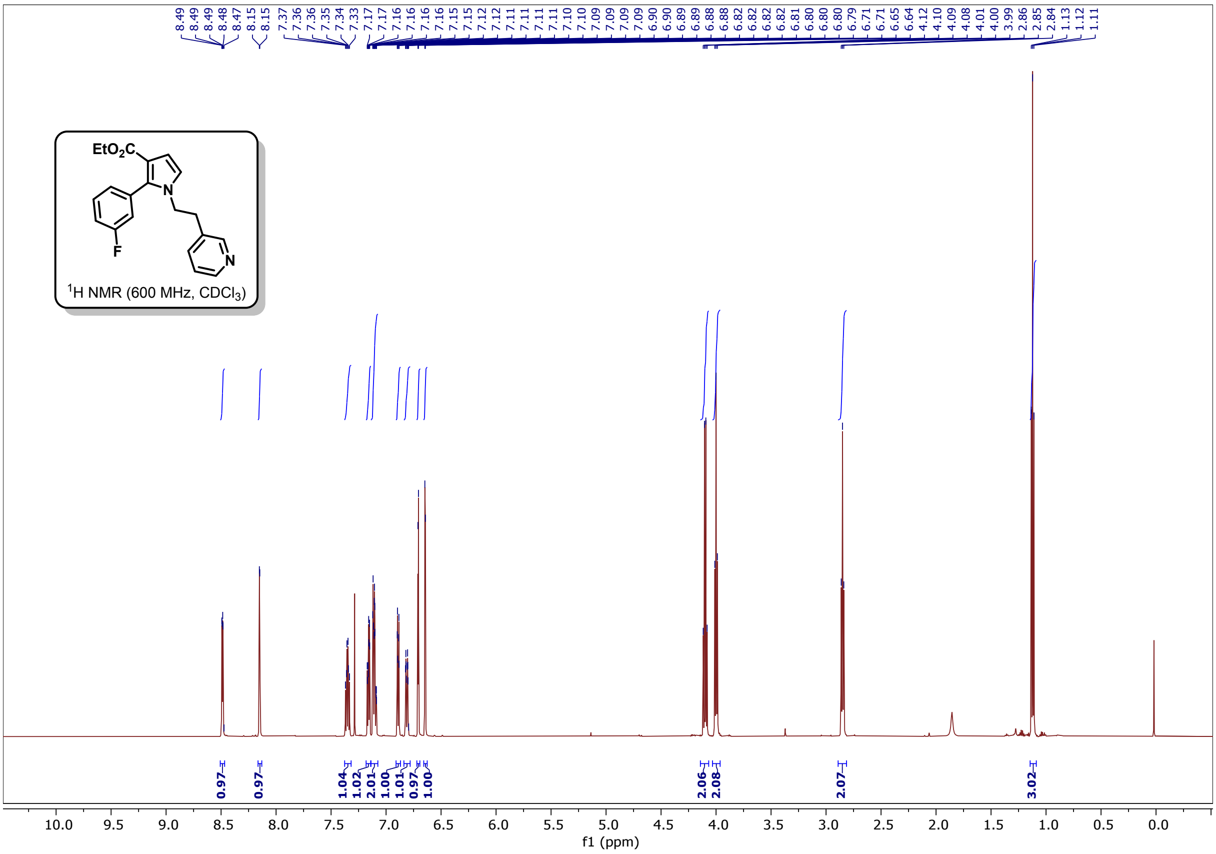

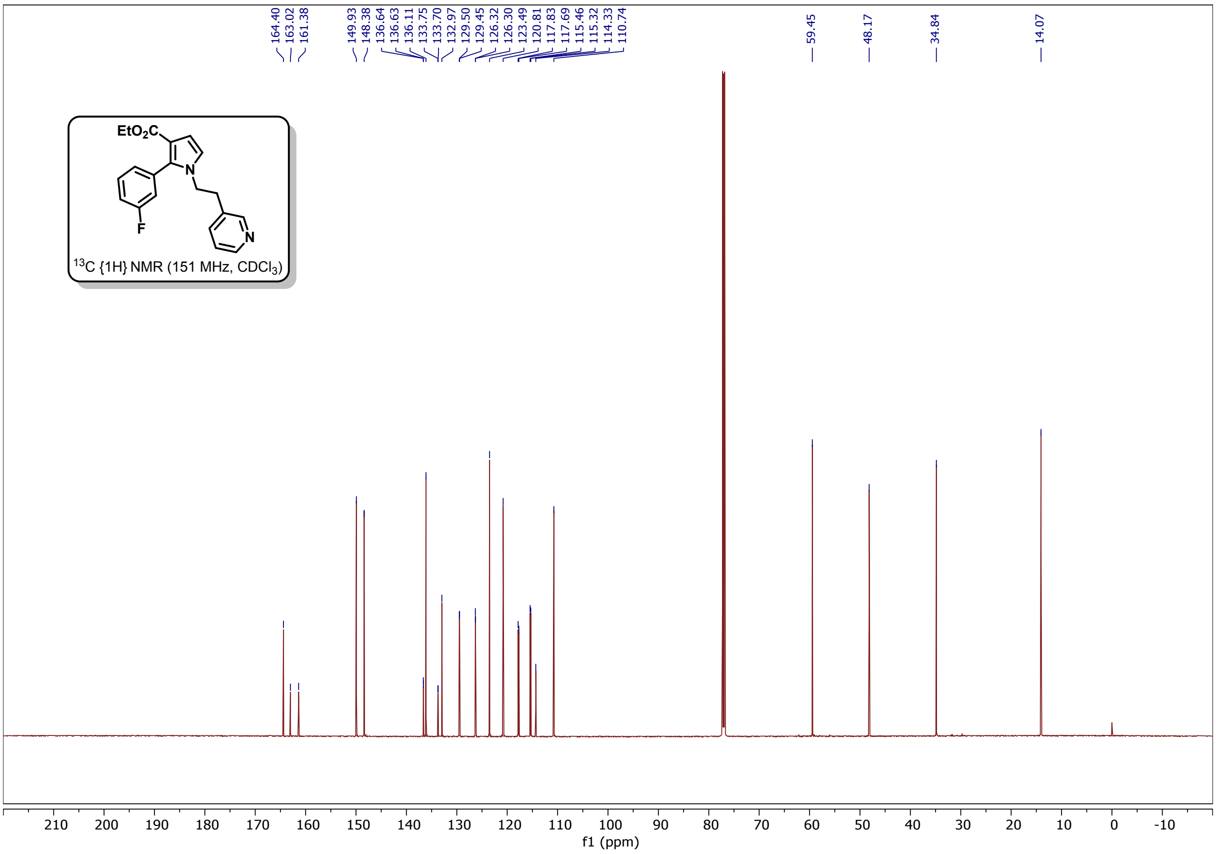

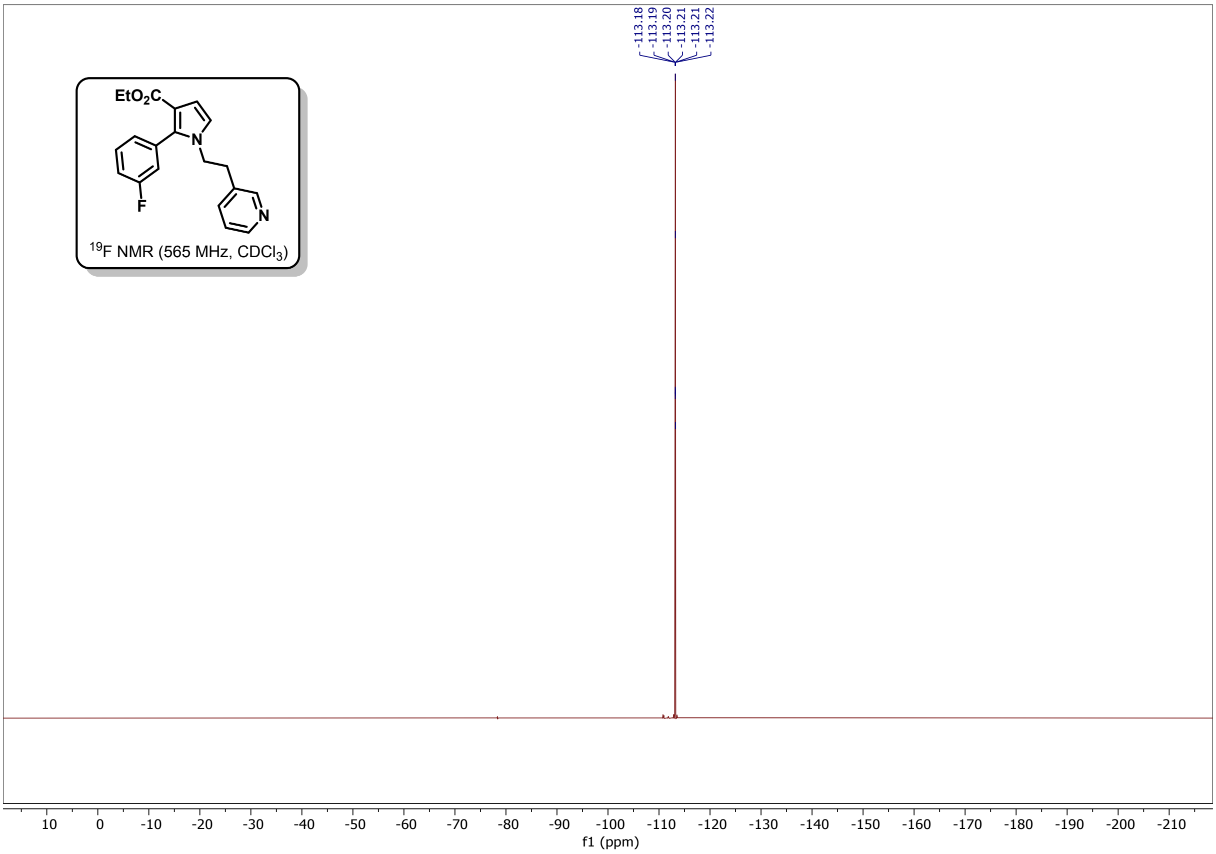
-113.18
-113.19
-113.20
-113.21
-113.21
-113.22
EtO2C
N
F
N
19F NMR (565 MHz, CDCl3)
10 0 -10 -20 -30 -40 -50 -60 -70 -80 -90 -100 -110 -120 -130 -140 -150 -160 -170 -180 -190 -200 -210
f1 (ppm)

Ethyl 2-(3-fluorophenyl)-1-(pyridin-2-ylmethyl)-1H-pyrrole-3-carboxylate (4ae):

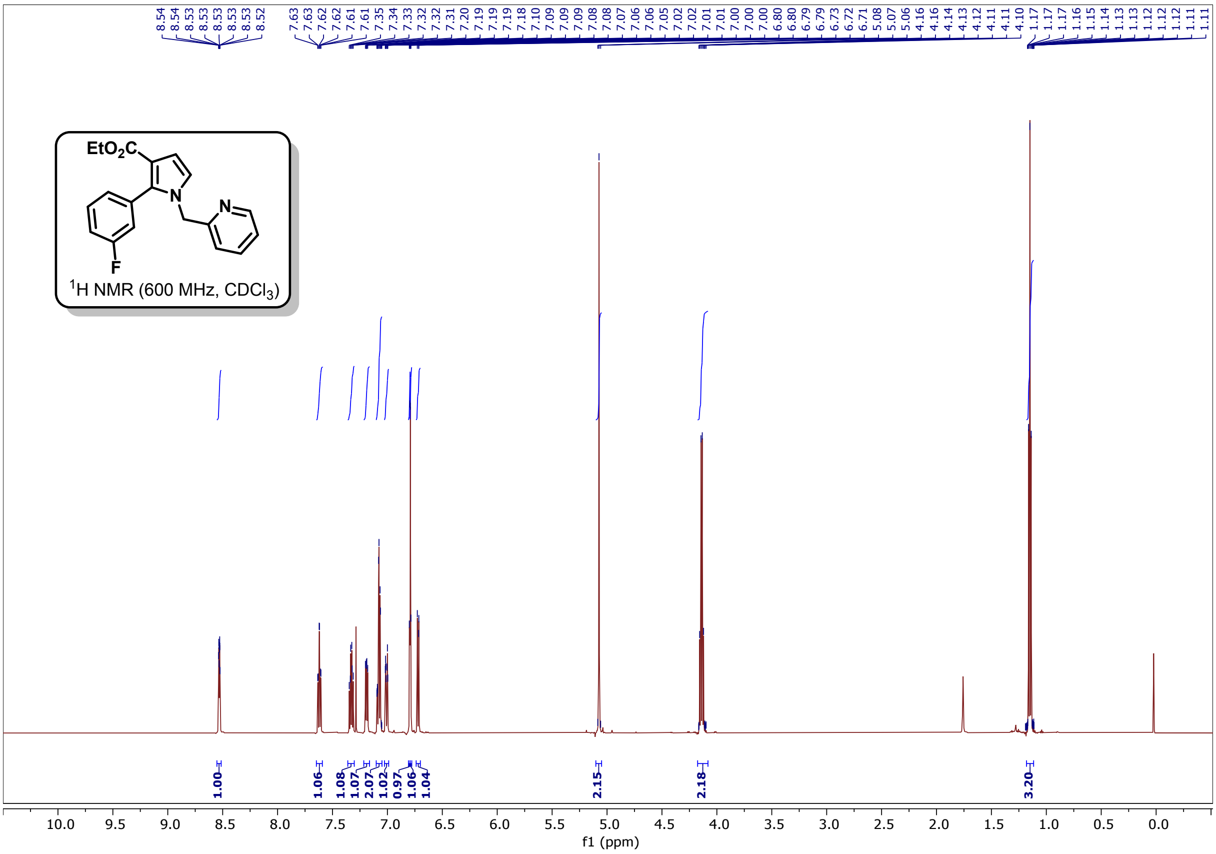

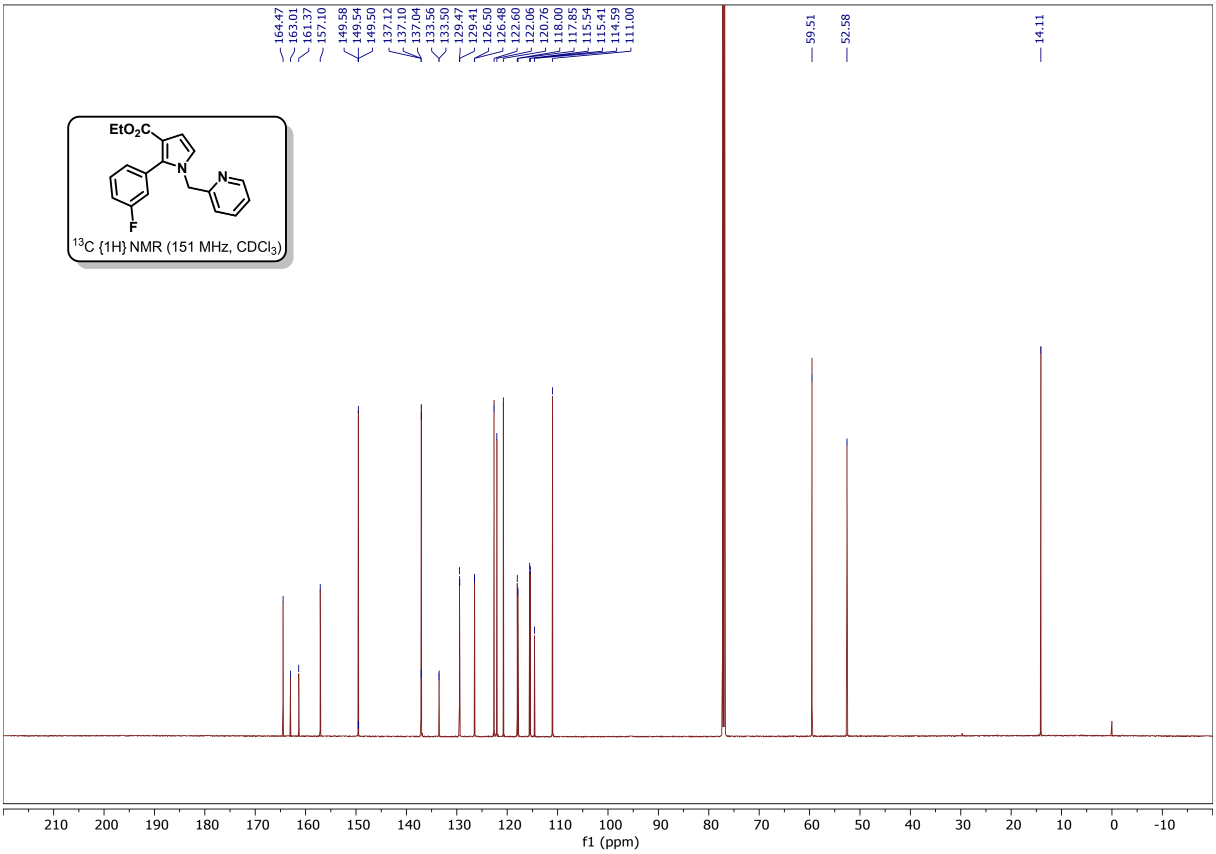

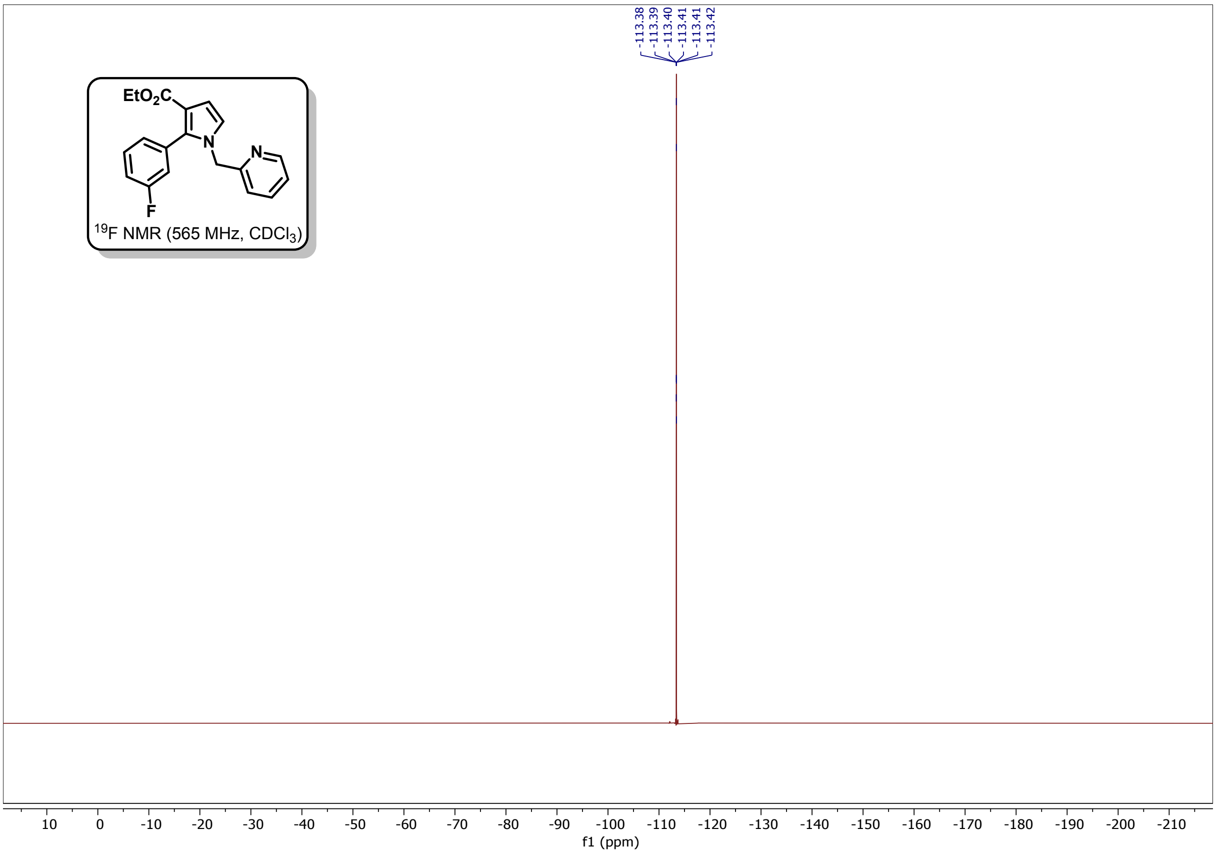
-113.38
-113.39
-113.40
-113.41
-113.41
-113.42
EtO2C
N
N
F
19F NMR (565 MHz, CDCl3)
10 0 -10 -20 -30 -40 -50 -60 -70 -80 -90 -100 -110 -120 -130 -140 -150 -160 -170 -180 -190 -200 -210
f1 (ppm)

Ethyl 1-(3-chlorobenzyl)-2-(3-fluorophenyl)-1H-pyrrole-3-carboxylate (4af):

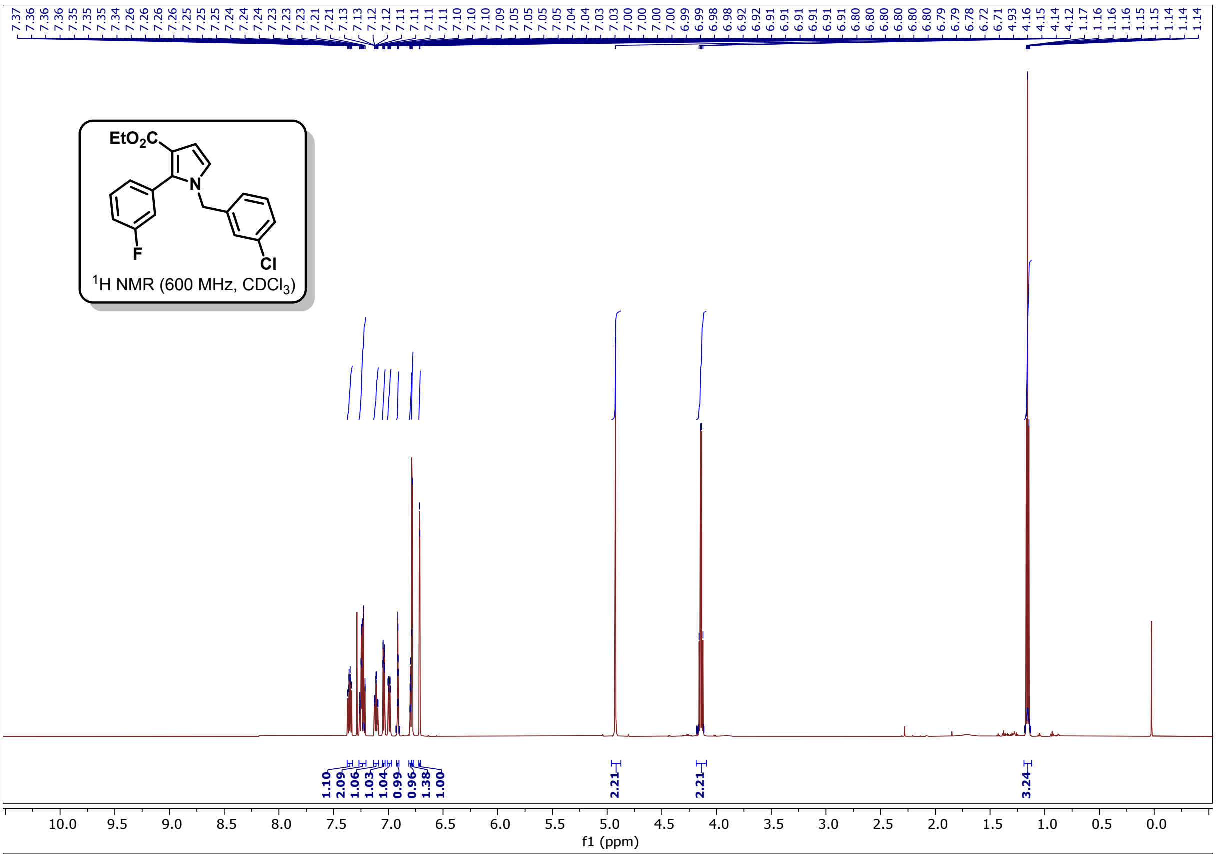

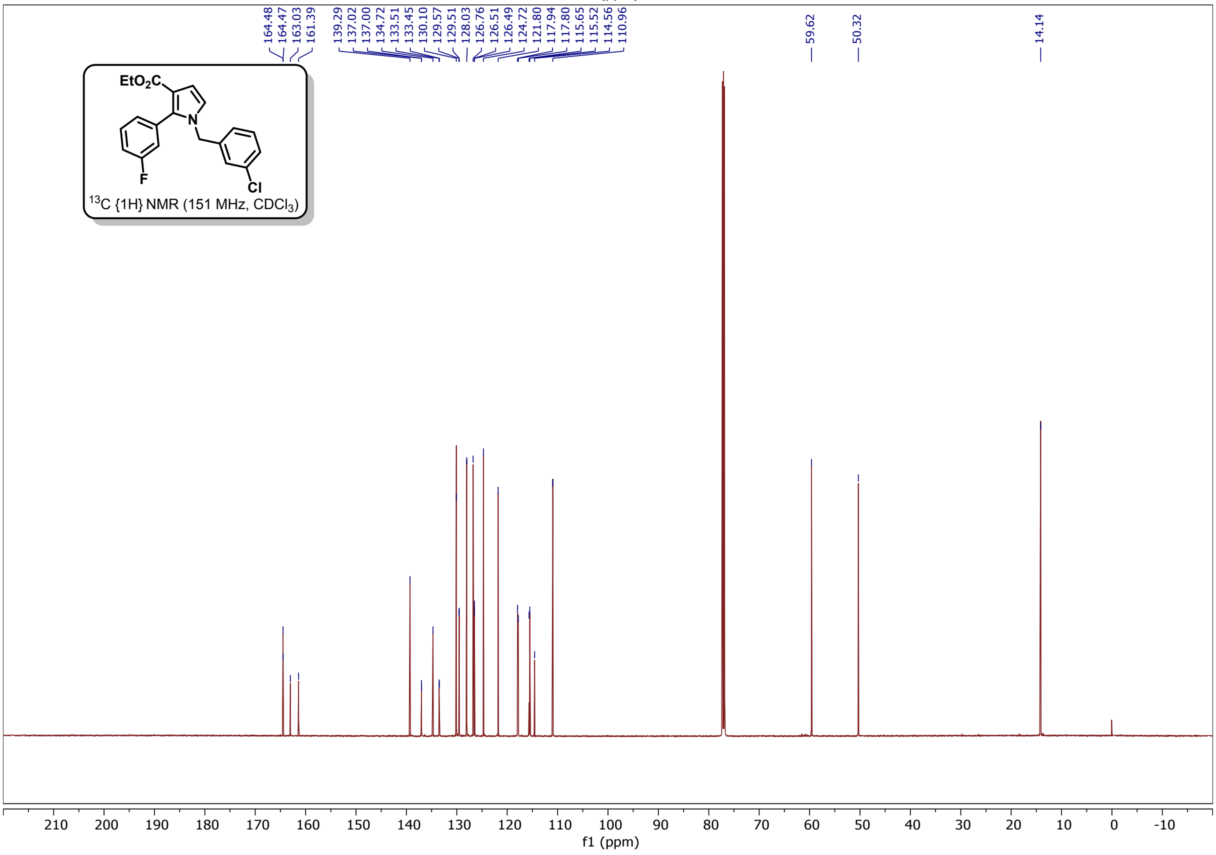

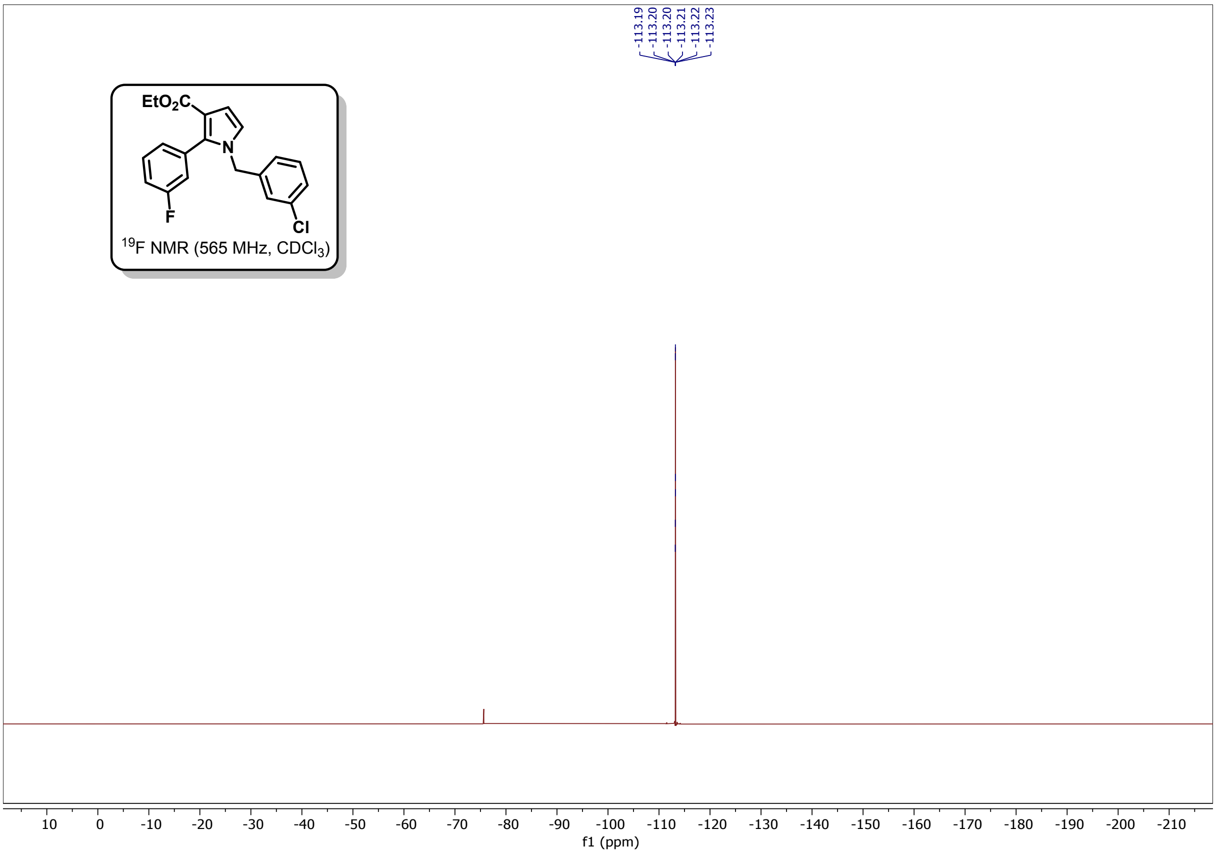
EtO2C
N
F
Cl
19F NMR (565 MHz, CDCl3)
-113.19
-113.20
-113.20
-113.21
-113.22
-113.23
10 0 -10 -20 -30 -40 -50 -60 -70 -80 -90 -100 -110 -120 -130 -140 -150 -160 -170 -180 -190 -200 -210
f1 (ppm)

Ethyl 1-(3-fluorobenzyl)-2-(3-fluorophenyl)-1H-pyrrole-3-carboxylate (4ag):

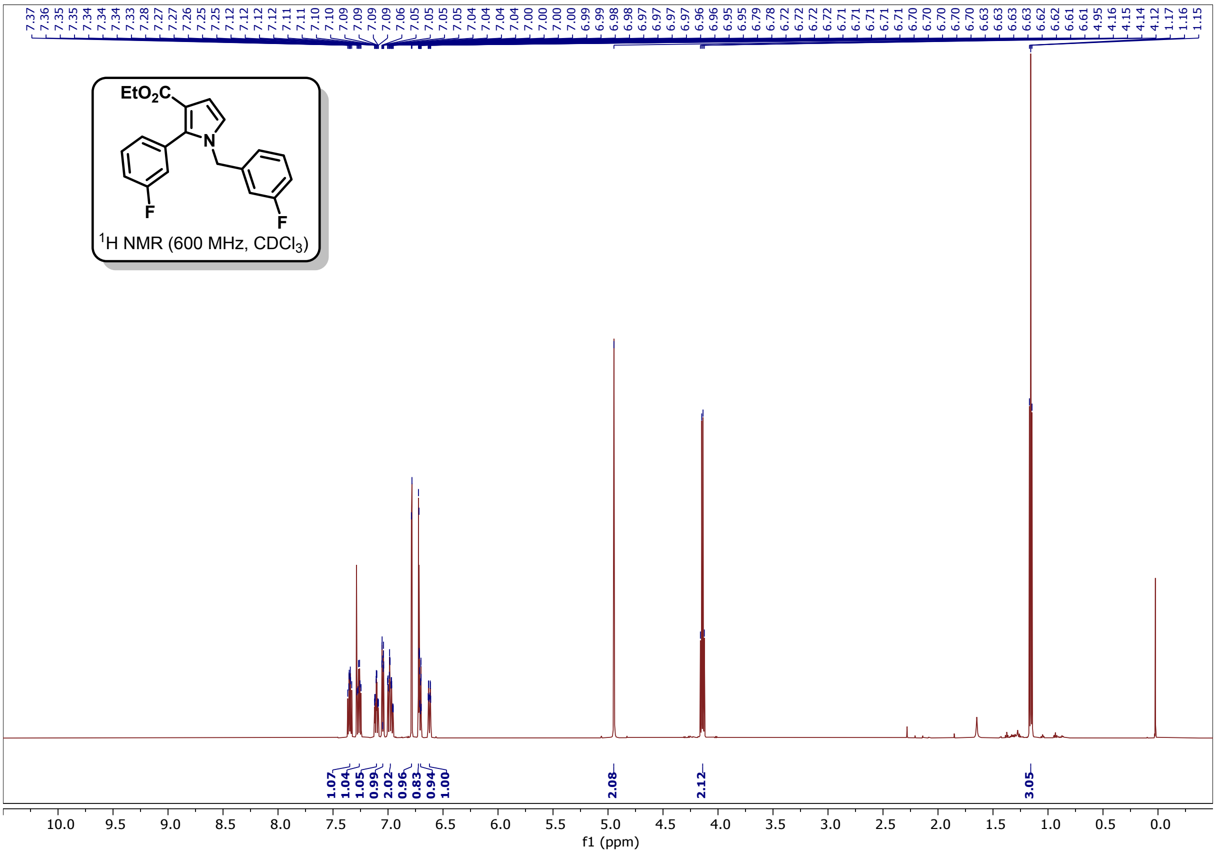

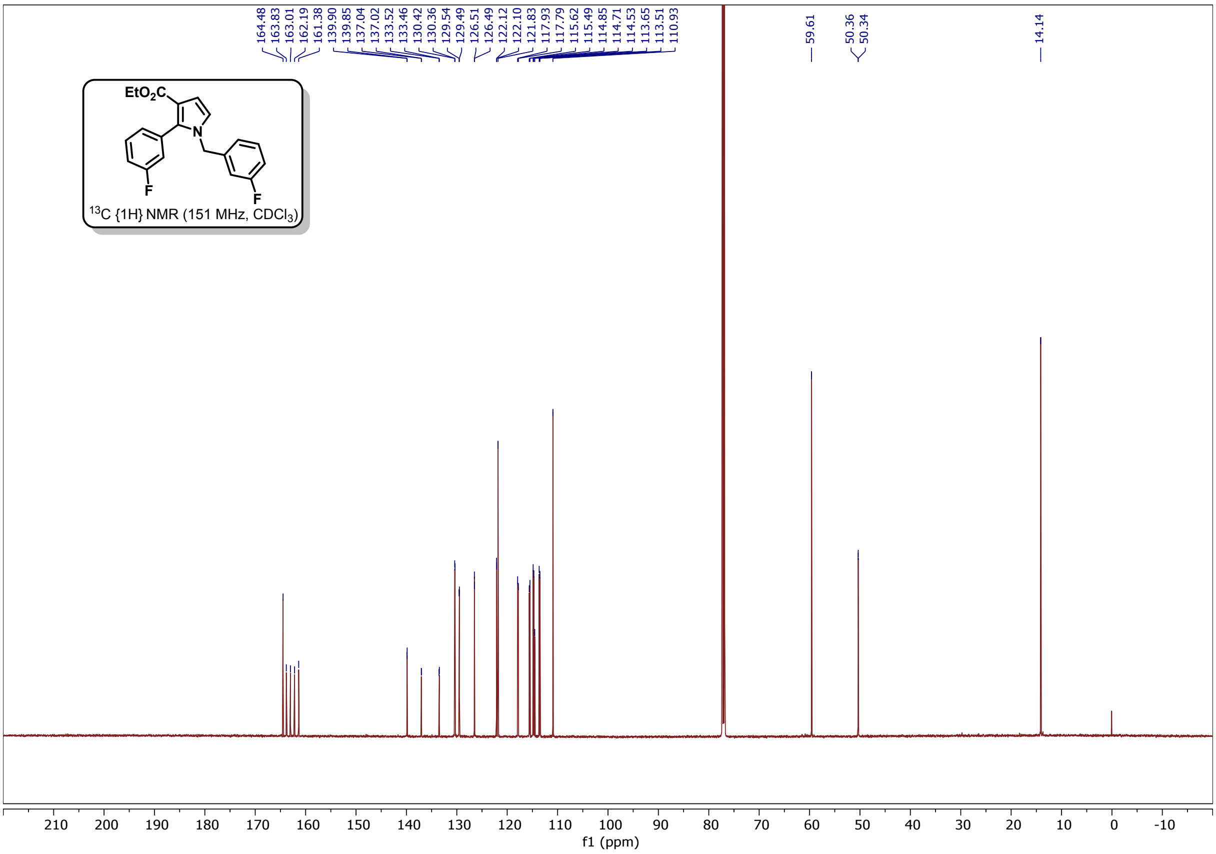

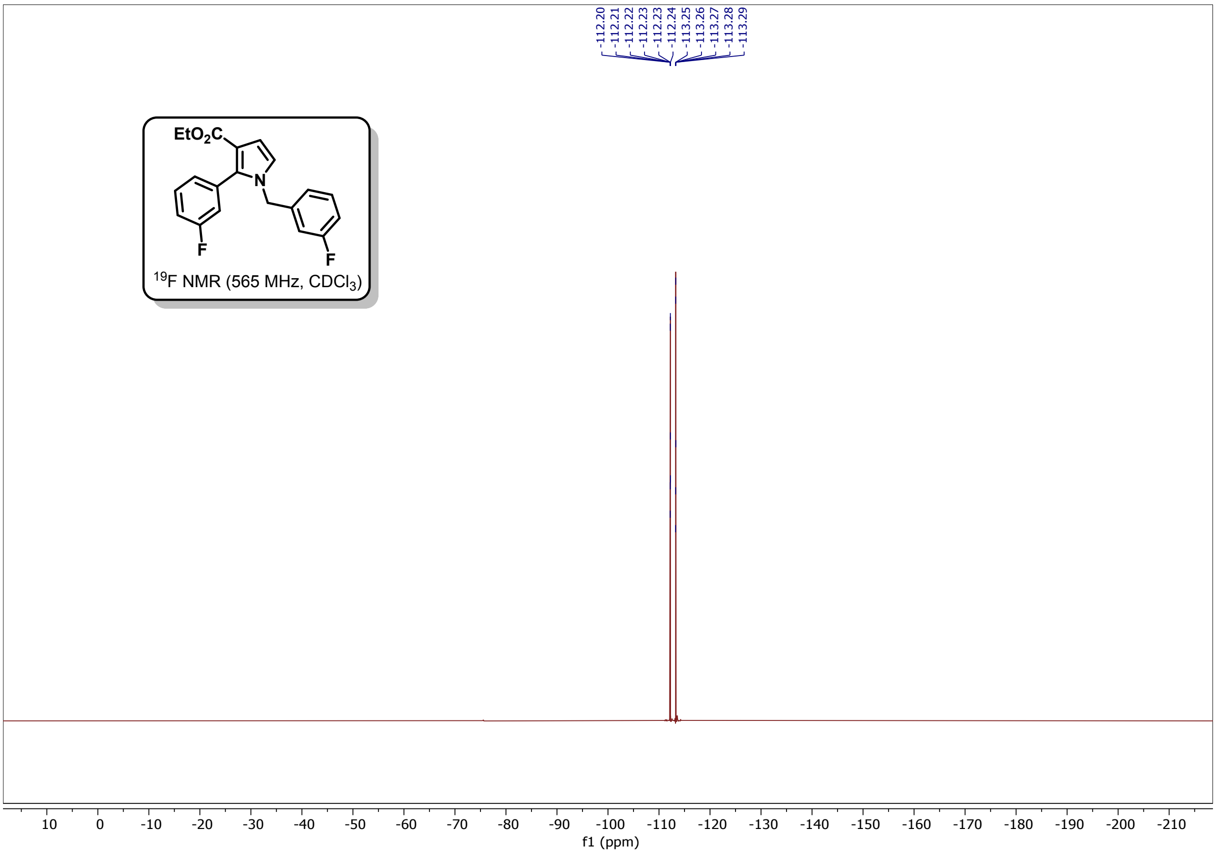

-112.20
-112.21
-112.22
-112.23
-112.23
-112.24
-113.25
-113.26
-113.27
-113.28
-113.29
EtO2C
N
F
F
19F NMR (565 MHz, CDCl3)
10 0 -10 -20 -30 -40 -50 -60 -70 -80 -90 -100 -110 -120 -130 -140 -150 -160 -170 -180 -190 -200 -210
f1 (ppm)

Ethyl 2-(3-fluorophenyl)-1-(3-(trifluoromethyl)benzyl)-1H-pyrrole-3-carboxylate (4ah):

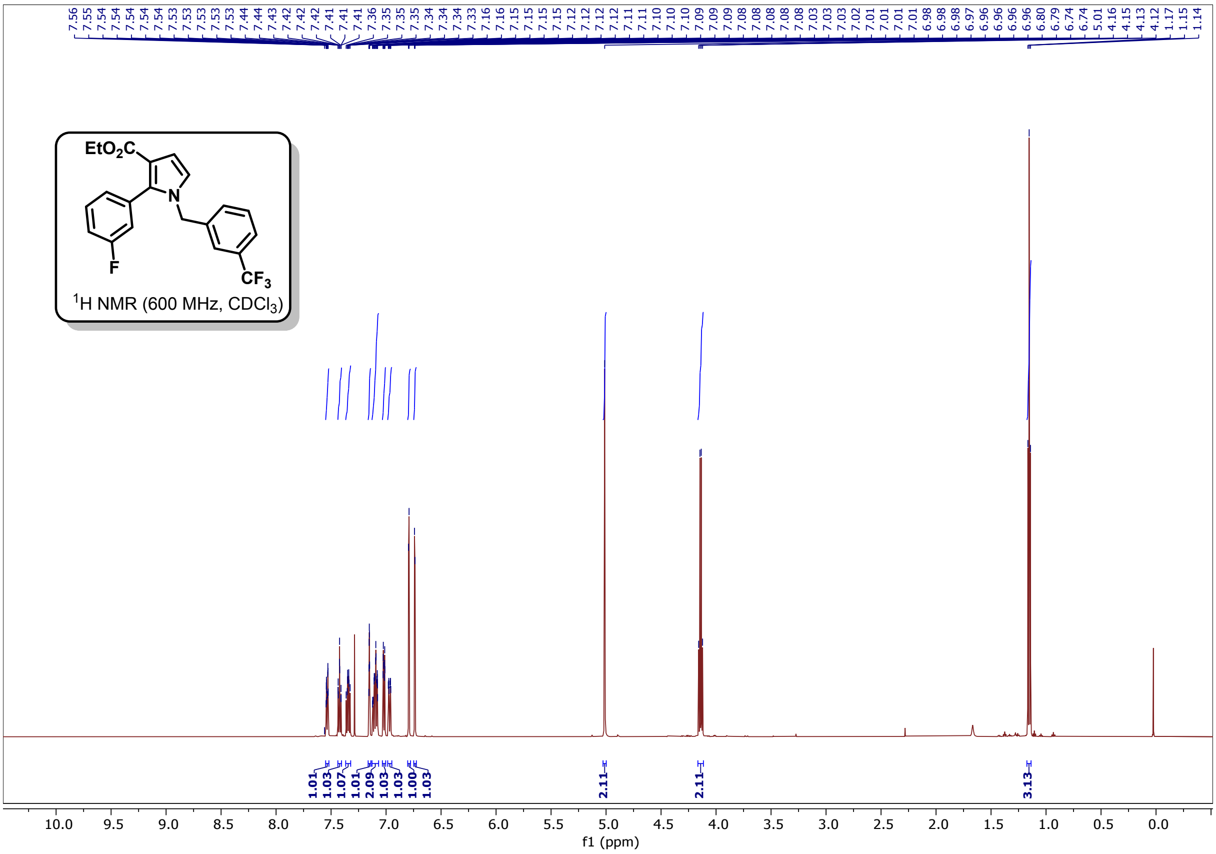

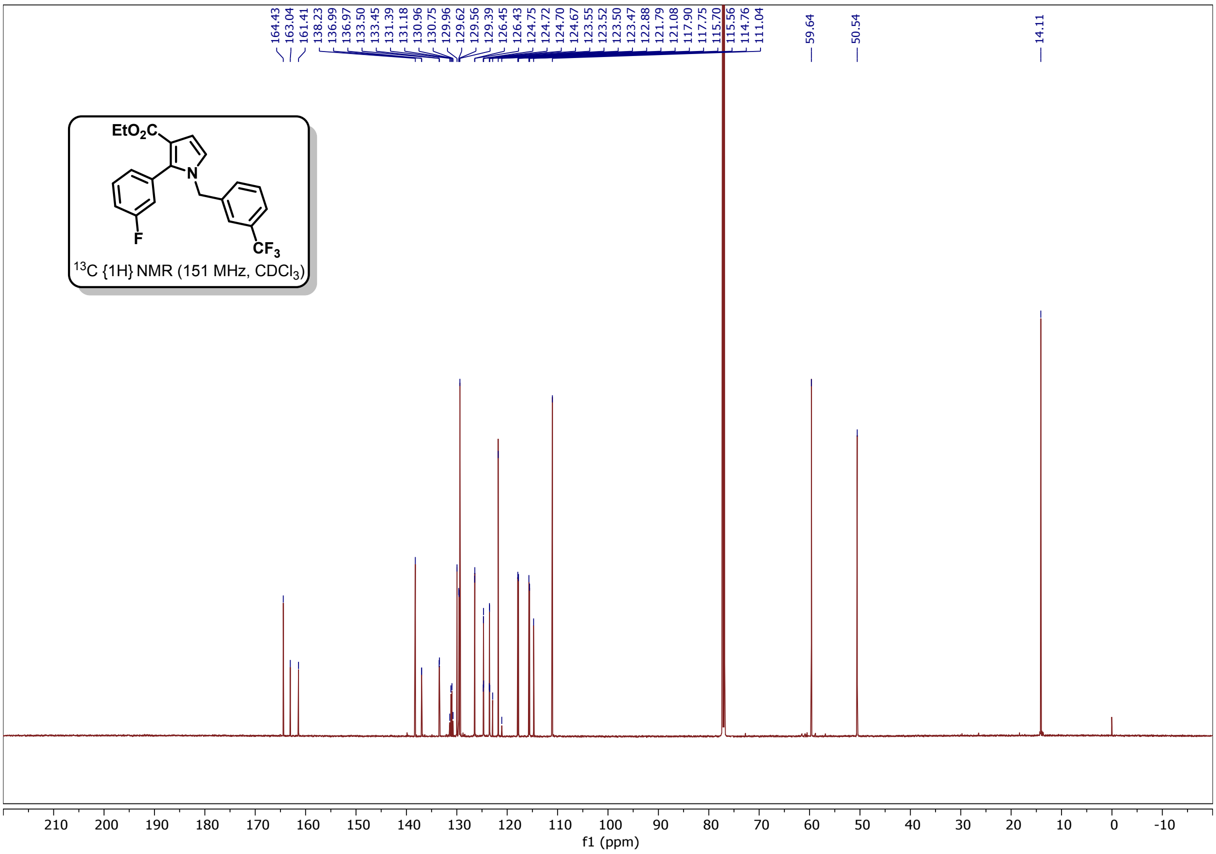

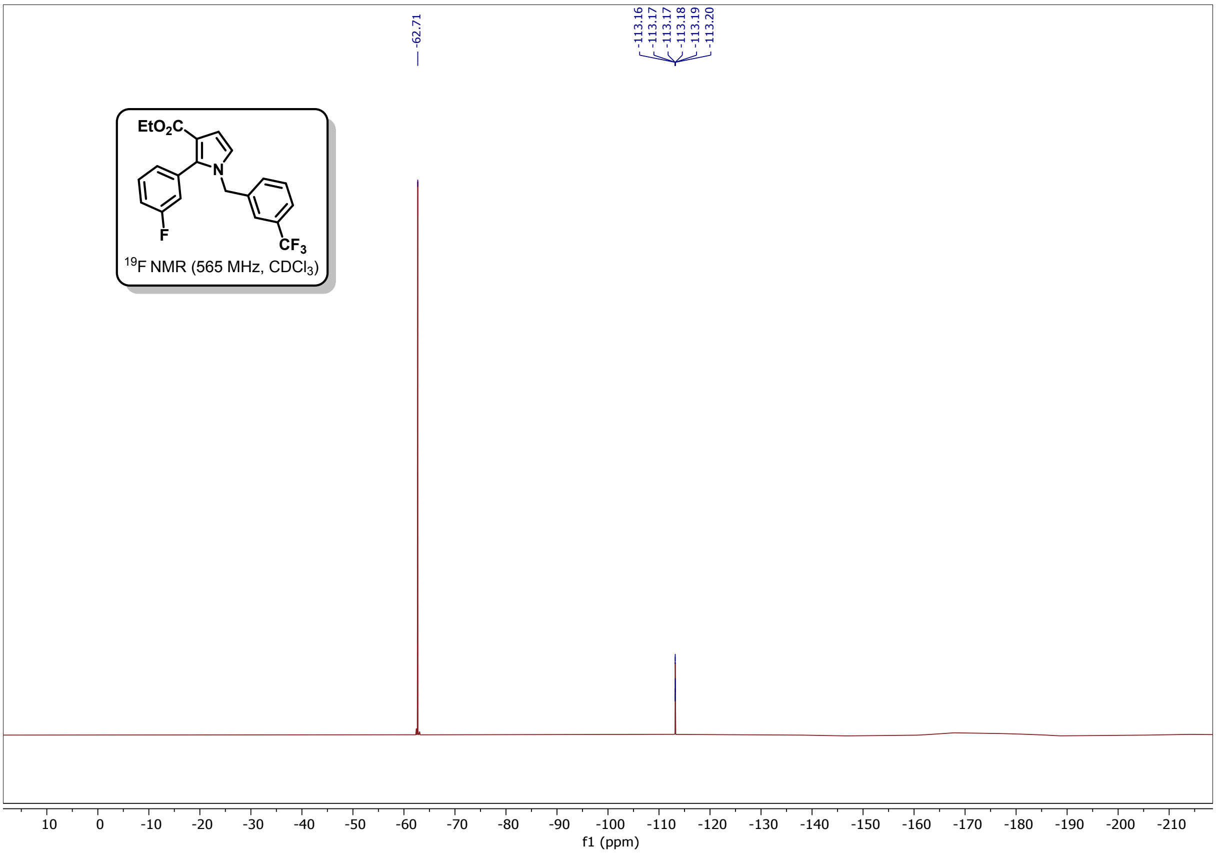

-62.71
-113.16
-113.17
-113.17
-113.18
-113.19
-113.20
EtO2C
N
F
CF3
19F NMR (565 MHz, CDCl3)
10 0 -10 -20 -30 -40 -50 -60 -70 -80 -90 -100 -110 -120 -130 -140 -150 -160 -170 -180 -190 -200 -210
f1 (ppm)

Ethyl 1-(4-(tert-butyl)benzyl)-2-(3-fluorophenyl)-1H-pyrrole-3-carboxylate (4ai):

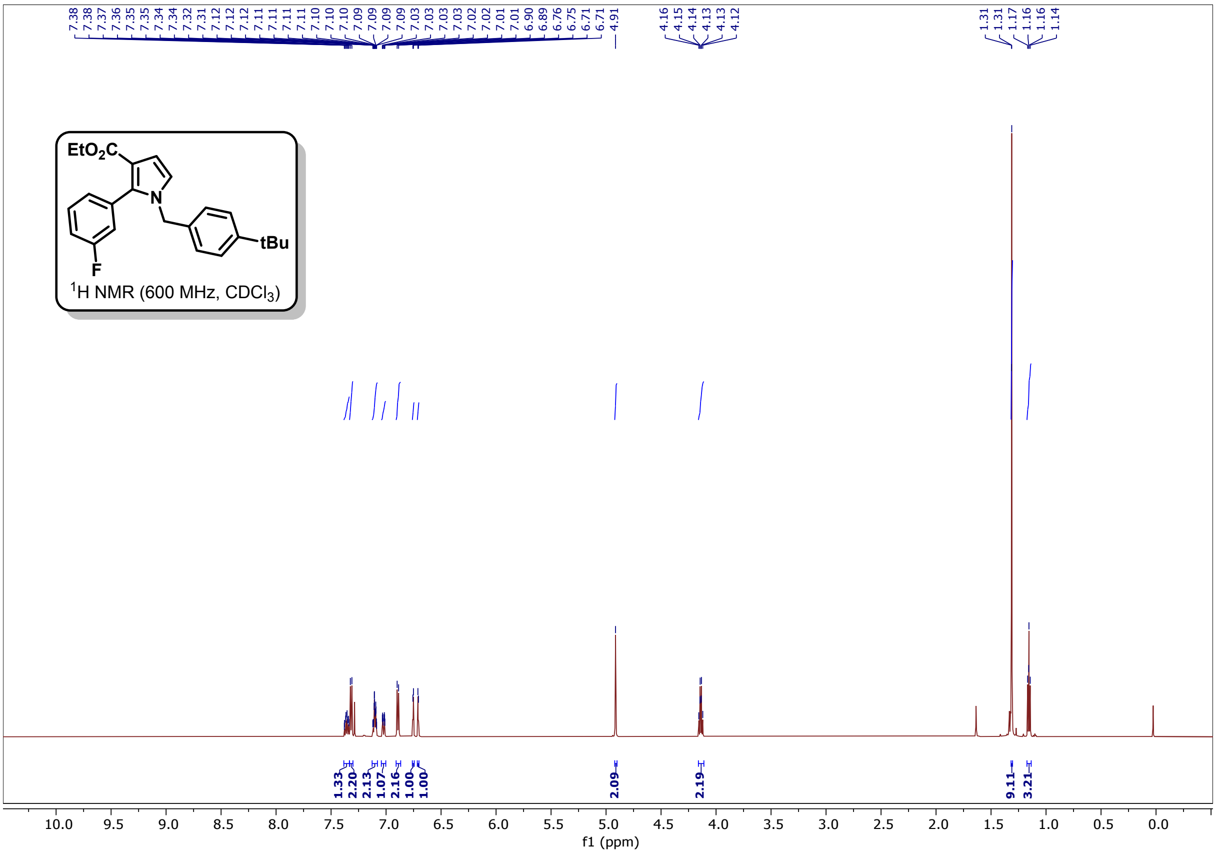

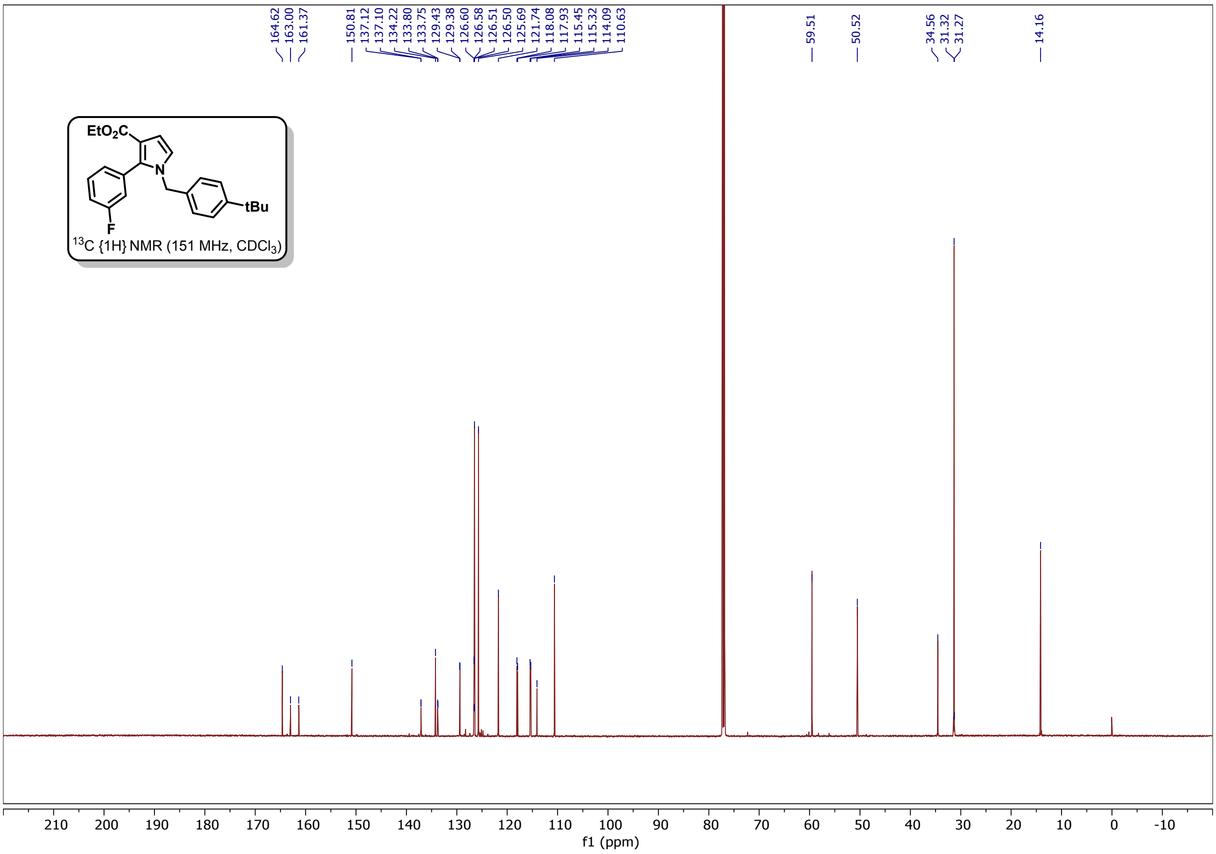

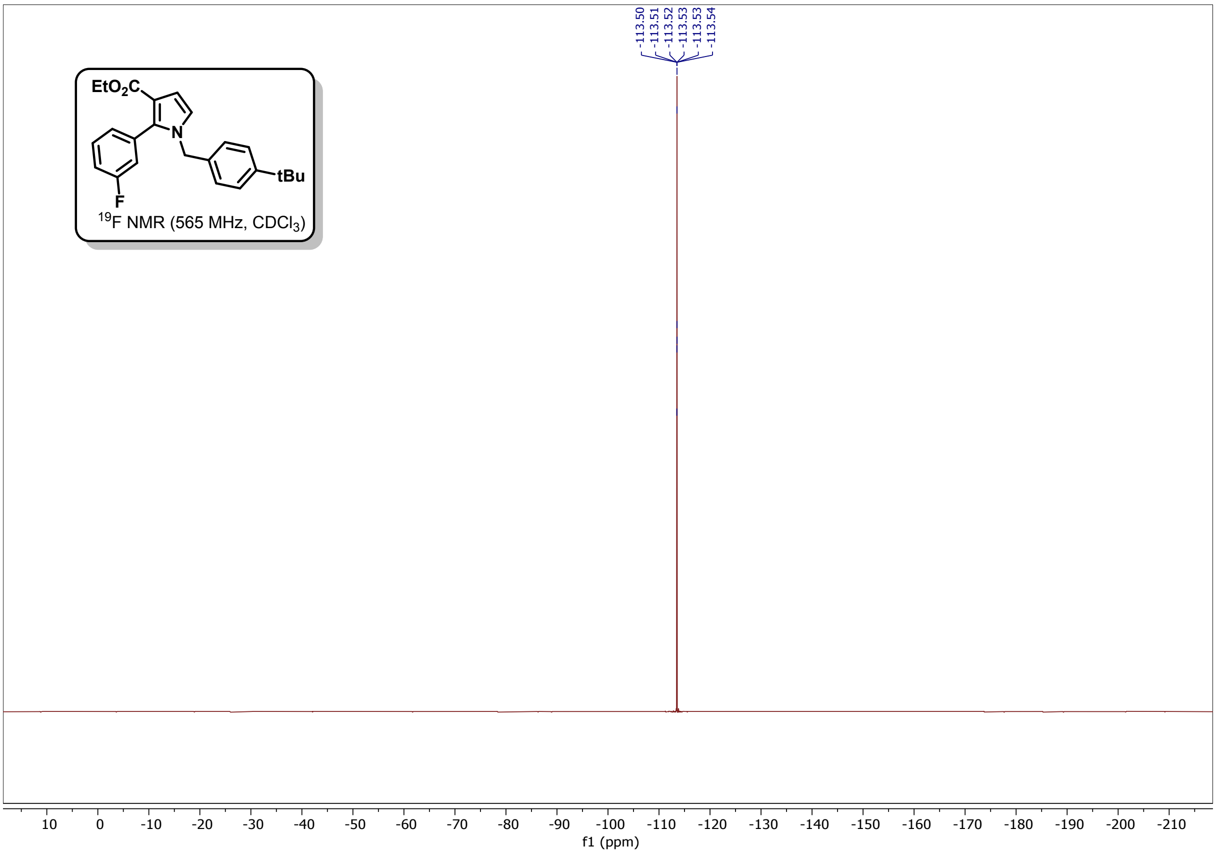

-113.50
-113.51
-113.52
-113.53
-113.53
-113.54
EtO2C
N
tBu
F
19F NMR (565 MHz, CDCl3)
10 0 -10 -20 -30 -40 -50 -60 -70 -80 -90 -100 -110 -120 -130 -140 -150 -160 -170 -180 -190 -200 -210
f1 (ppm)

Ethyl 1-butyl-2-(3-fluorophenyl)-1H-pyrrole-3-carboxylate (4aj):

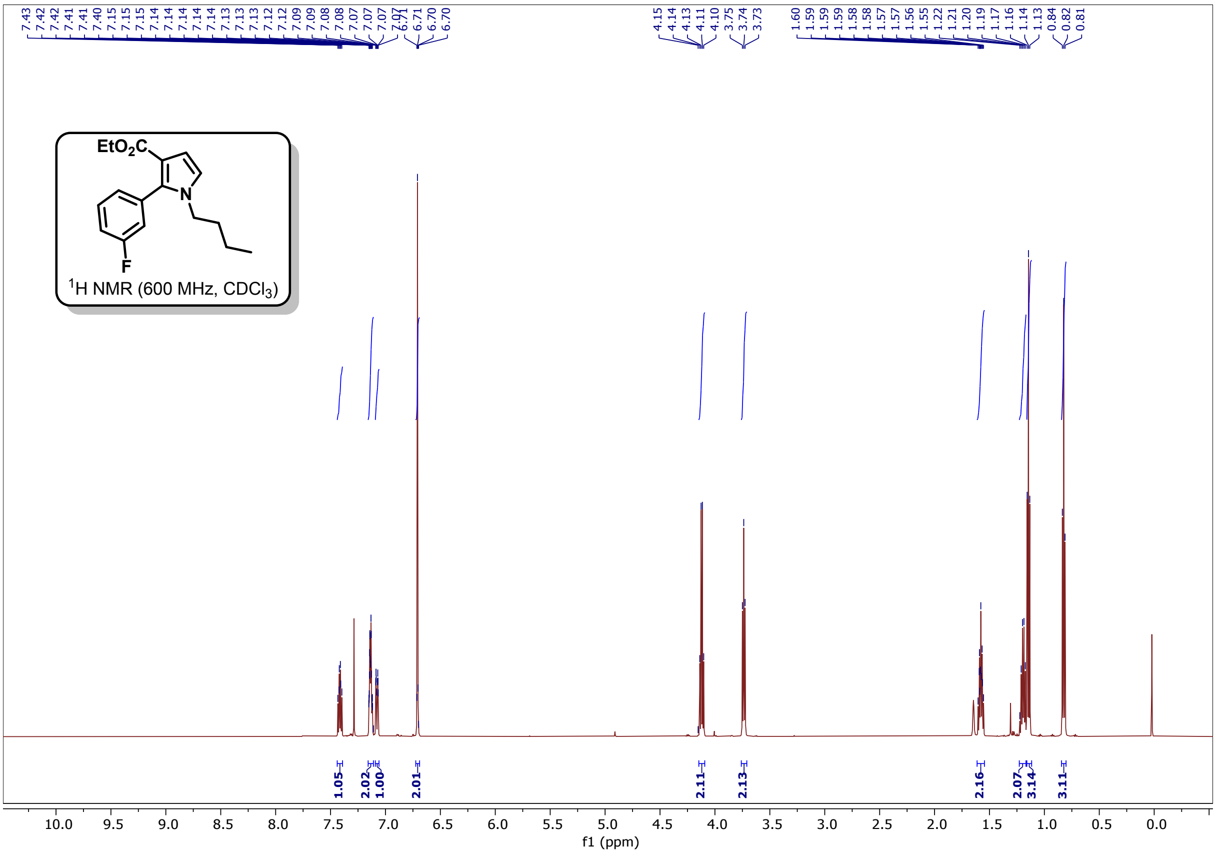

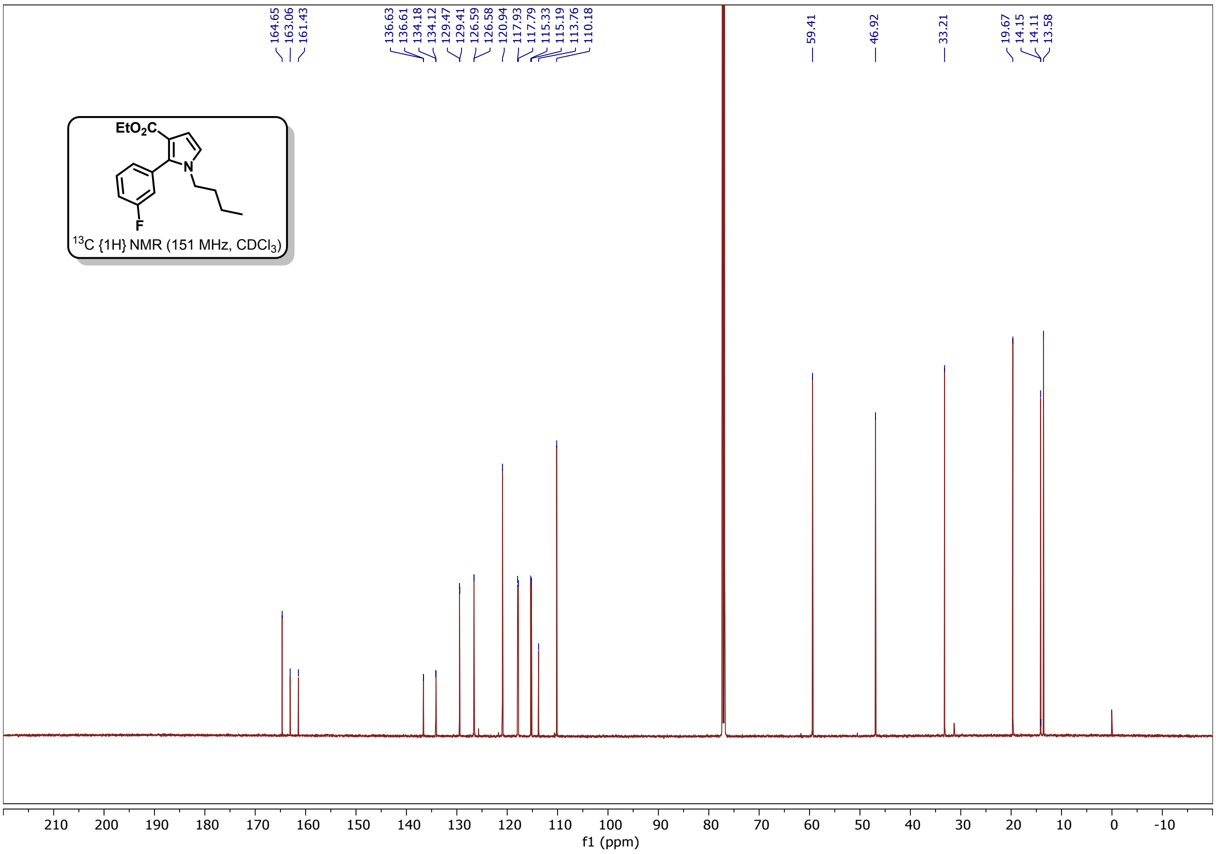

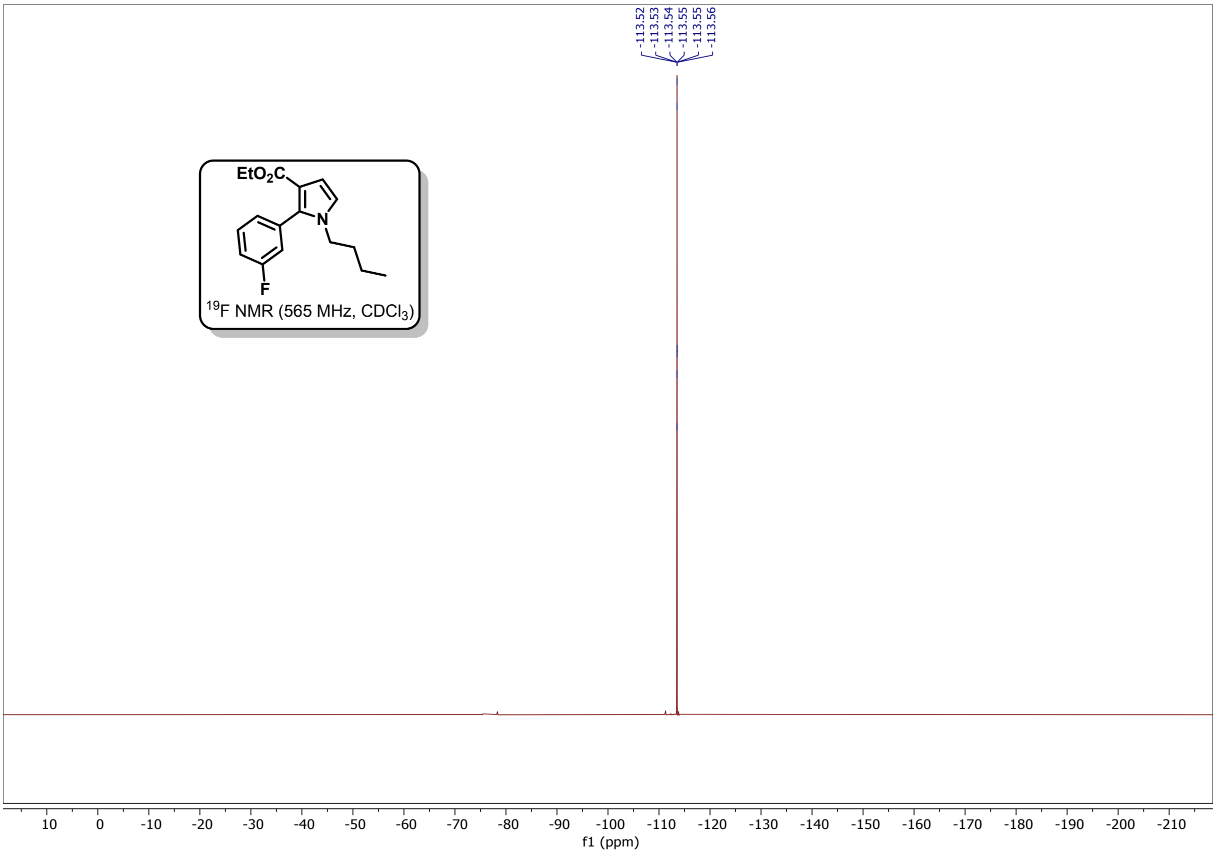
-113.52
-113.53
-113.54
-113.55
-113.55
-113.56
EtO2C
N
F
19F NMR (565 MHz, CDCl3)
10 0 -10 -20 -30 -40 -50 -60 -70 -80 -90 -100 -110 -120 -130 -140 -150 -160 -170 -180 -190 -200 -210
f1 (ppm)

Ethyl 1-(2,2-dimethoxyethyl)-2-(3-fluorophenyl)-1H-pyrrole-3-carboxylate (4ak):

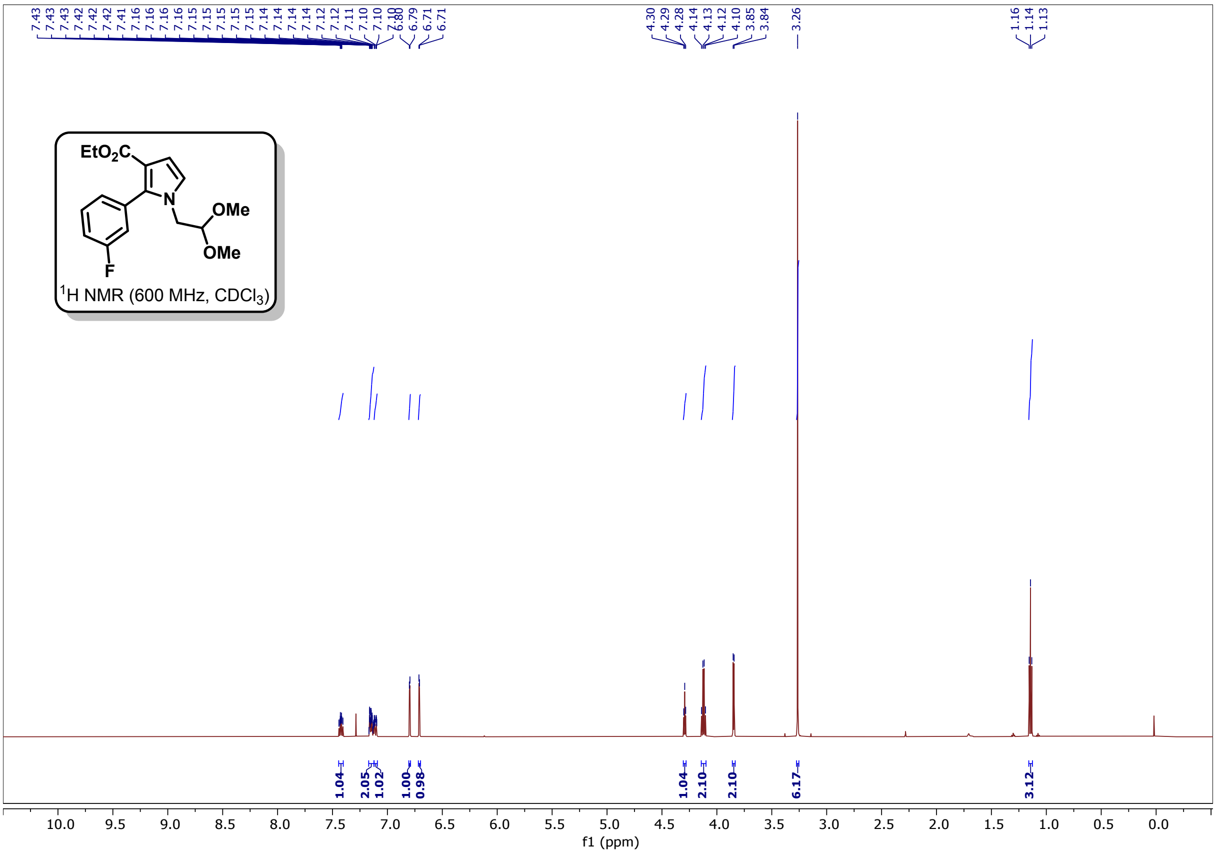

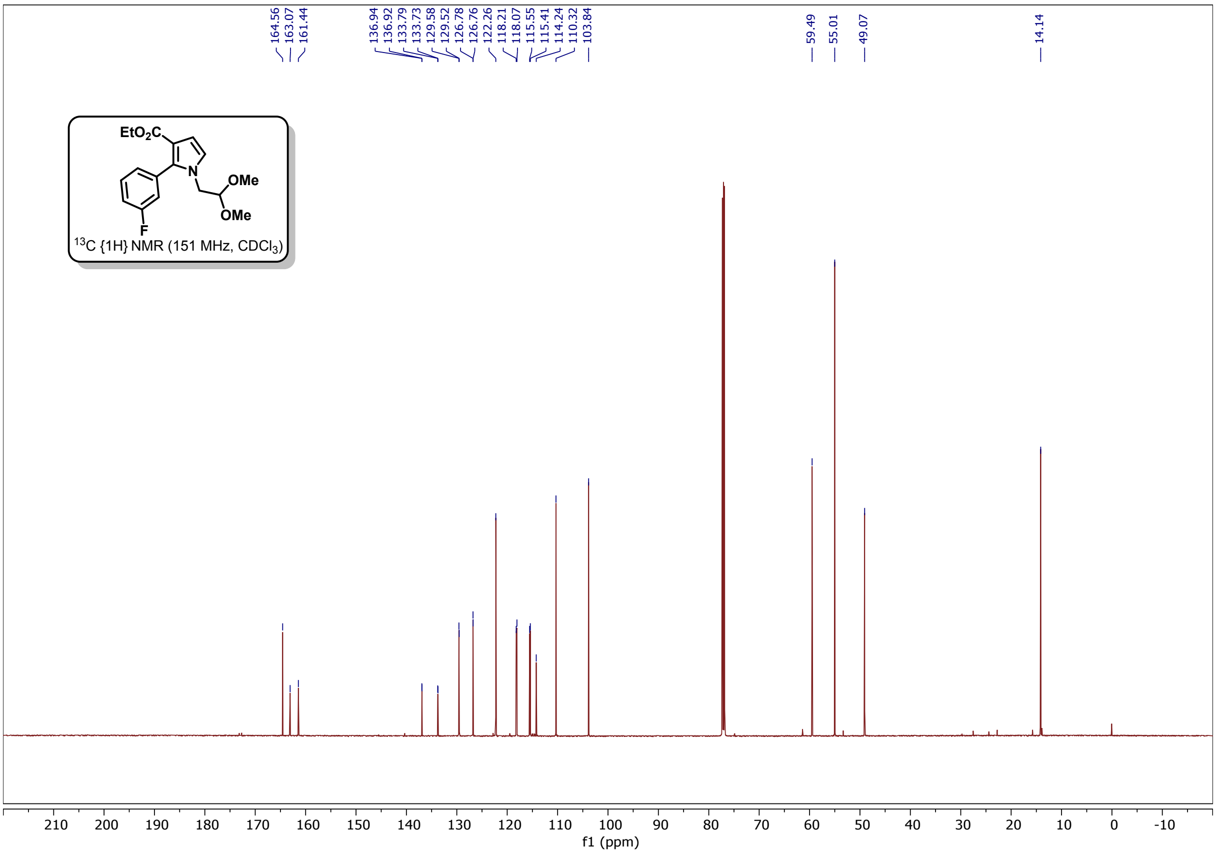

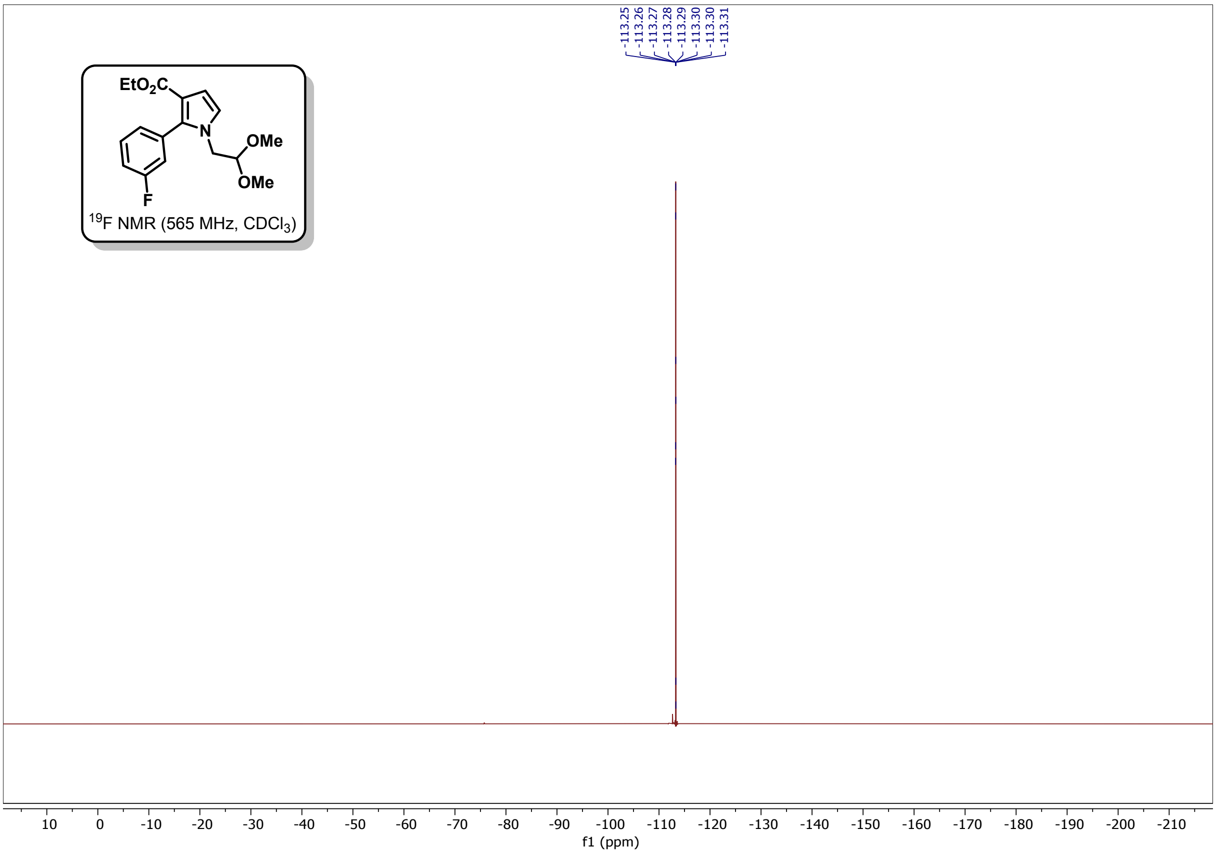

EtO2C
N
OMe
OMe
F
19F NMR (565 MHz, CDCl3)
-113.25
-113.26
-113.27
-113.28
-113.29
-113.30
-113.30
-113.31
10 0 -10 -20 -30 -40 -50 -60 -70 -80 -90 -100 -110 -120 -130 -140 -150 -160 -170 -180 -190 -200 -210
f1 (ppm)

Ethyl 2-(3-fluorophenyl)-1-(prop-2-yn-1-yl)-1H-pyrrole-3-carboxylate (4al):

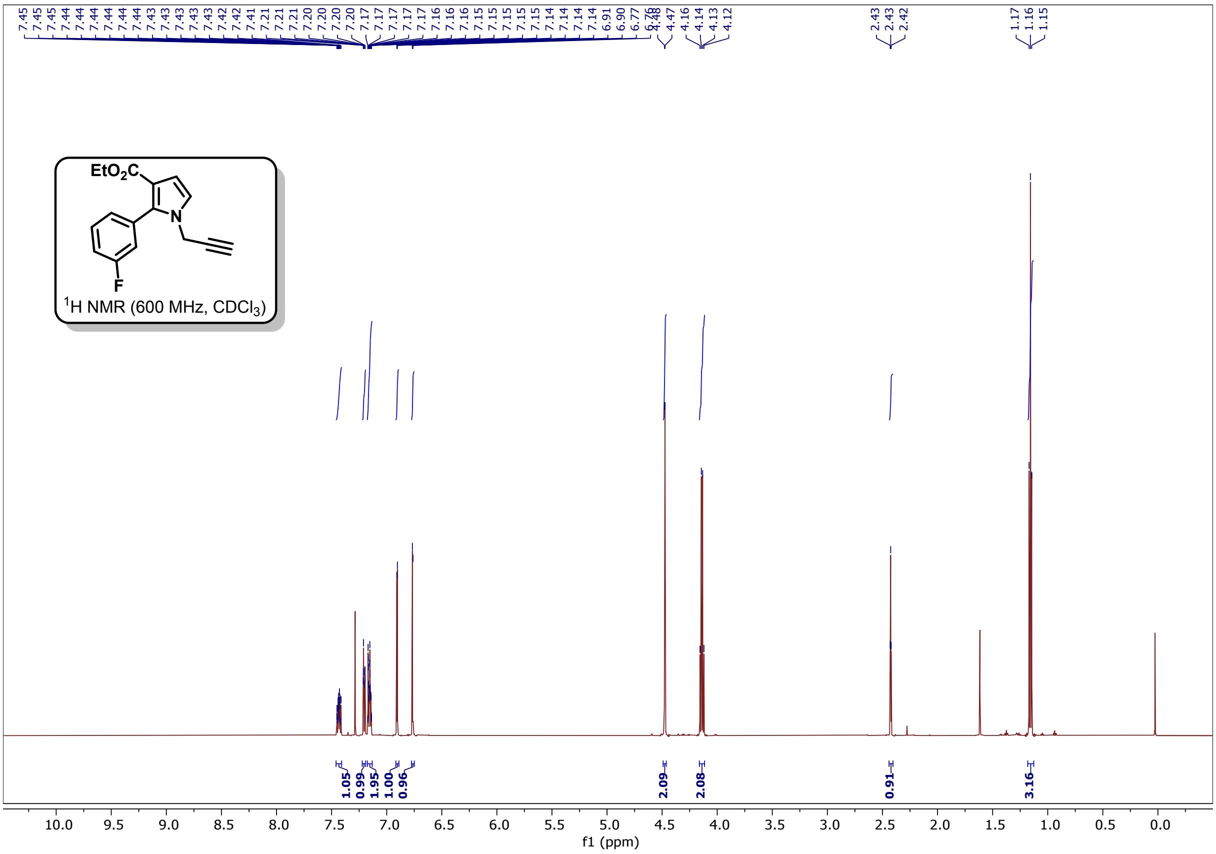

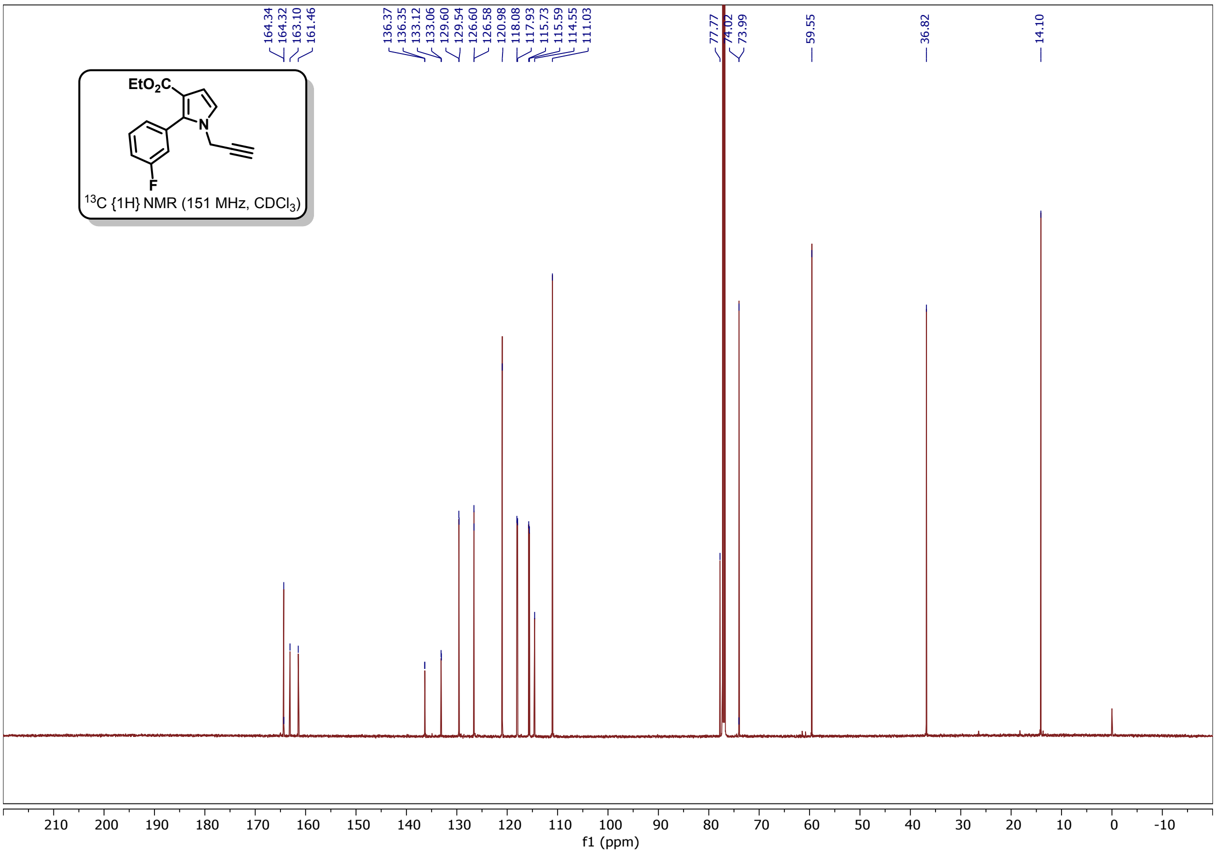

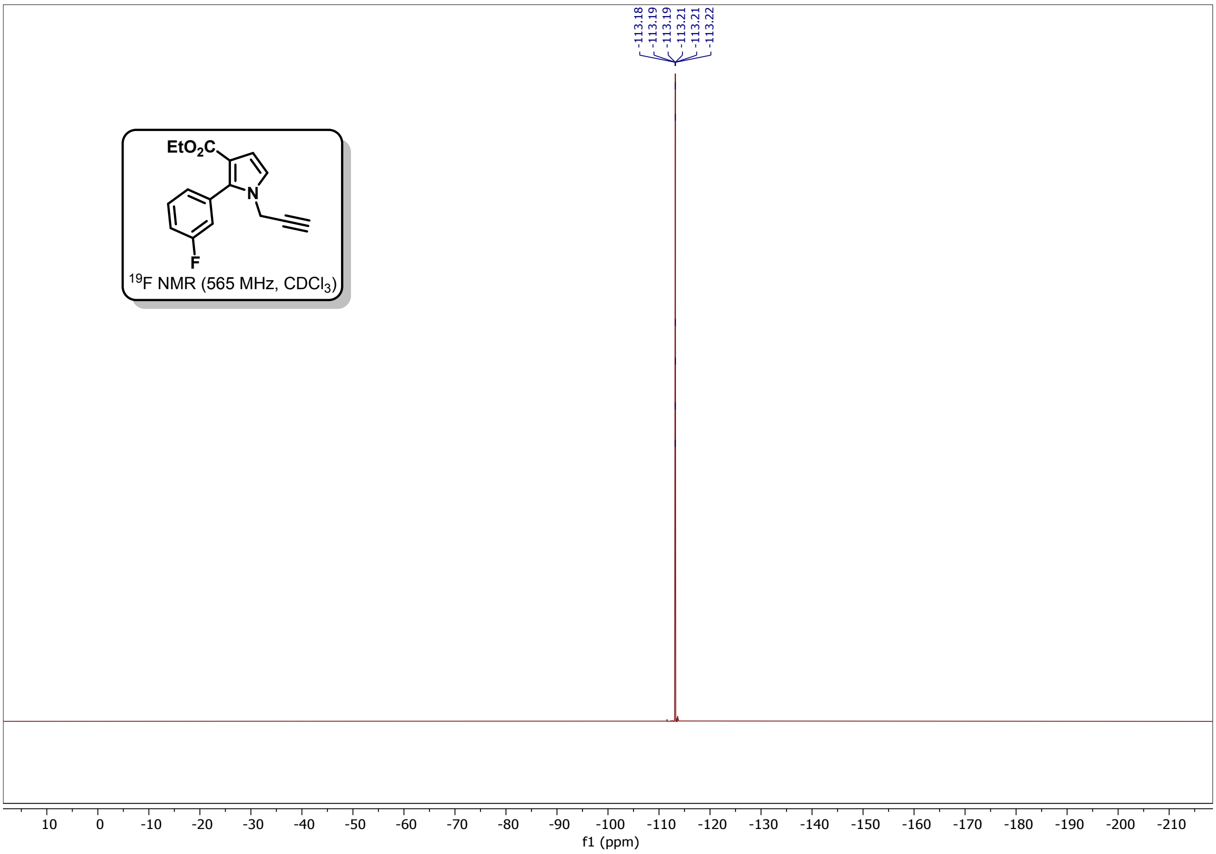

-113.18
-113.19
-113.19
-113.21
-113.21
-113.22
EtO2C
N
F
19F NMR (565 MHz, CDCl3)
10 0 -10 -20 -30 -40 -50 -60 -70 -80 -90 -100 -110 -120 -130 -140 -150 -160 -170 -180 -190 -200 -210
f1 (ppm)

Ethyl 1-allyl-2-(3-fluorophenyl)-1H-pyrrole-3-carboxylate (4am):

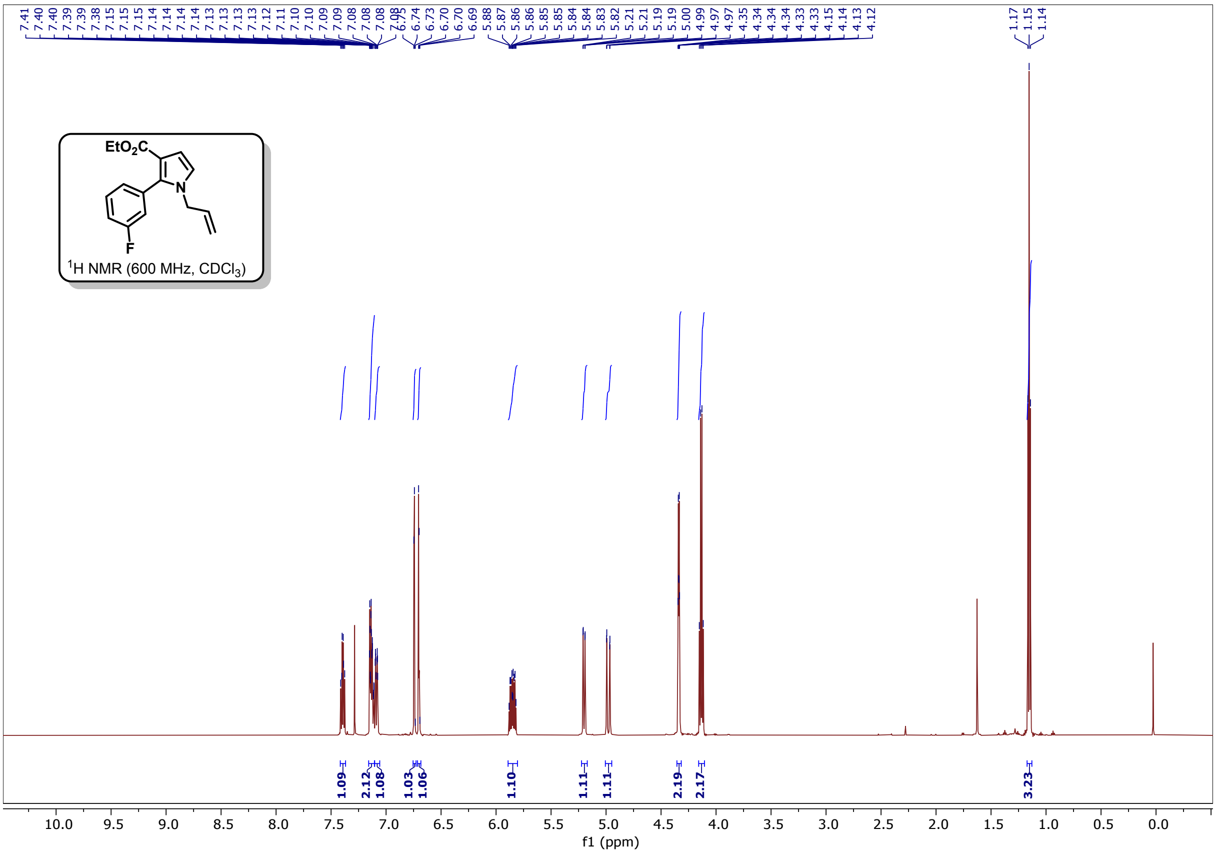

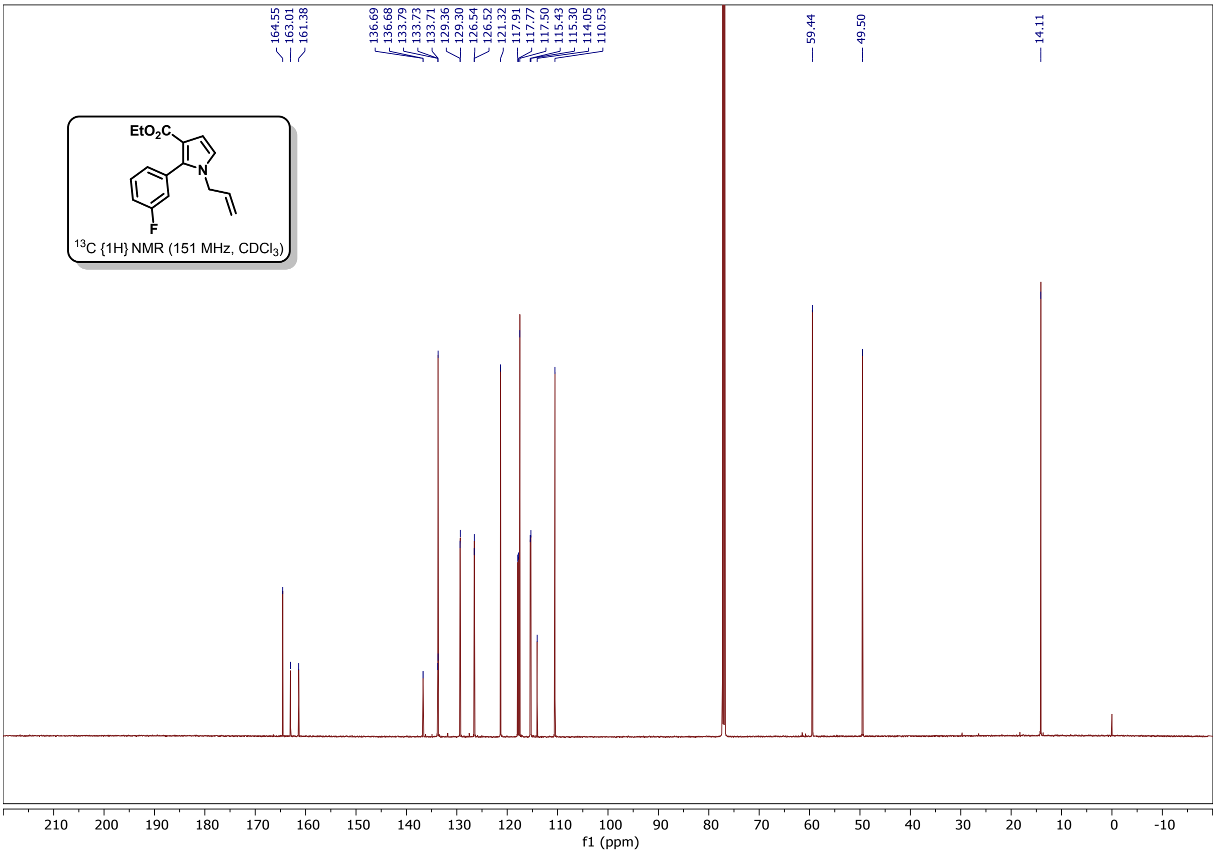

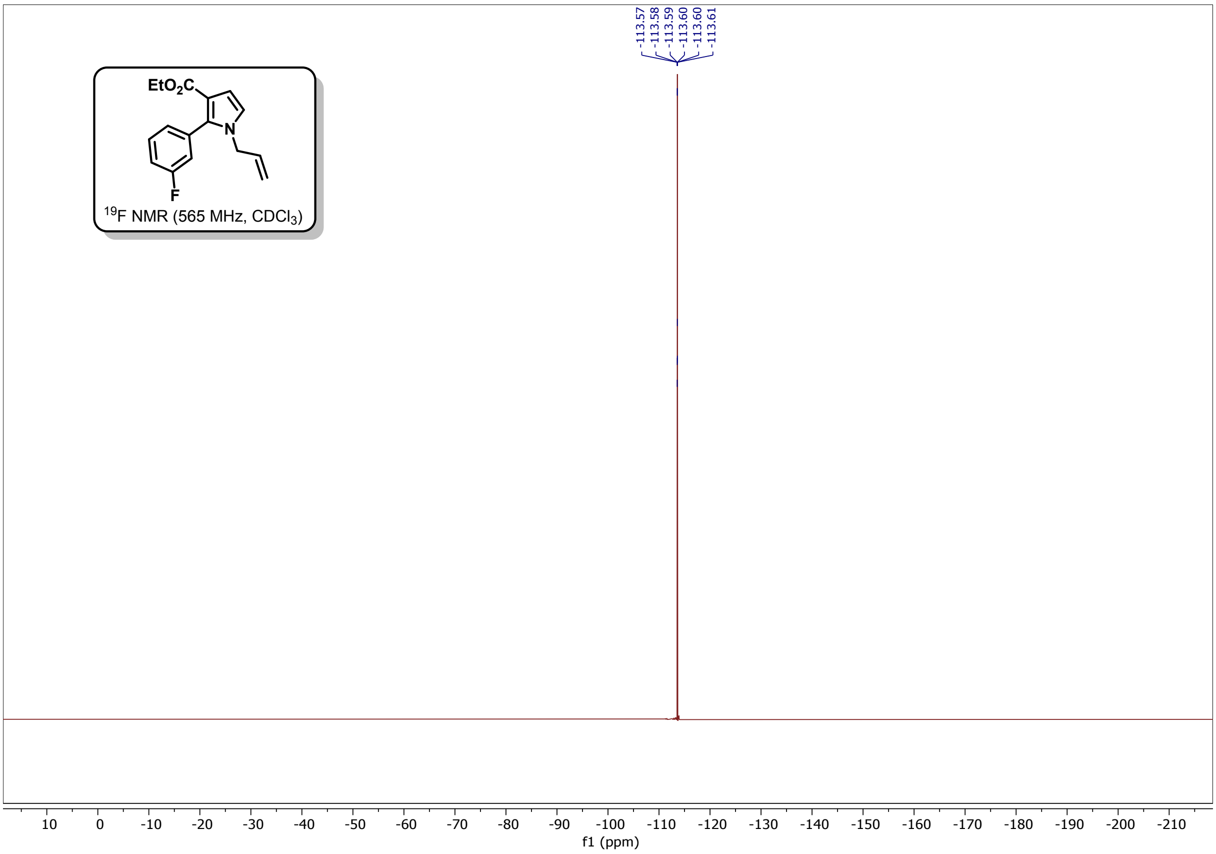

-113.57
-113.58
-113.59
-113.60
-113.60
-113.61
EtO2C
N
F
19F NMR (565 MHz, CDCl3)
10 0 -10 -20 -30 -40 -50 -60 -70 -80 -90 -100 -110 -120 -130 -140 -150 -160 -170 -180 -190 -200 -210
f1 (ppm)

Ethyl (R)-1-(1-(tert-butoxycarbonyl)pyrrolidin-3-yl)-2-(3-fluorophenyl)-1H-pyrrole-3-carboxylate (4an):

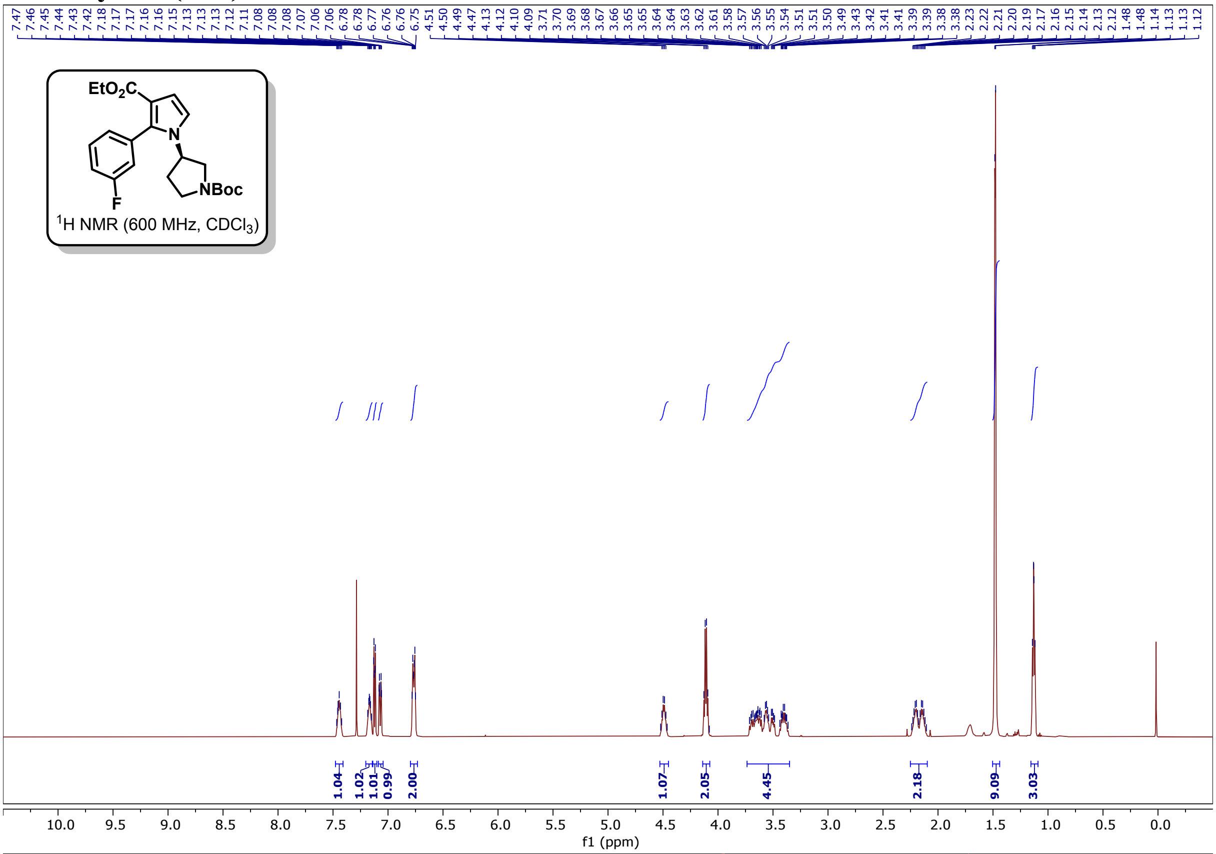

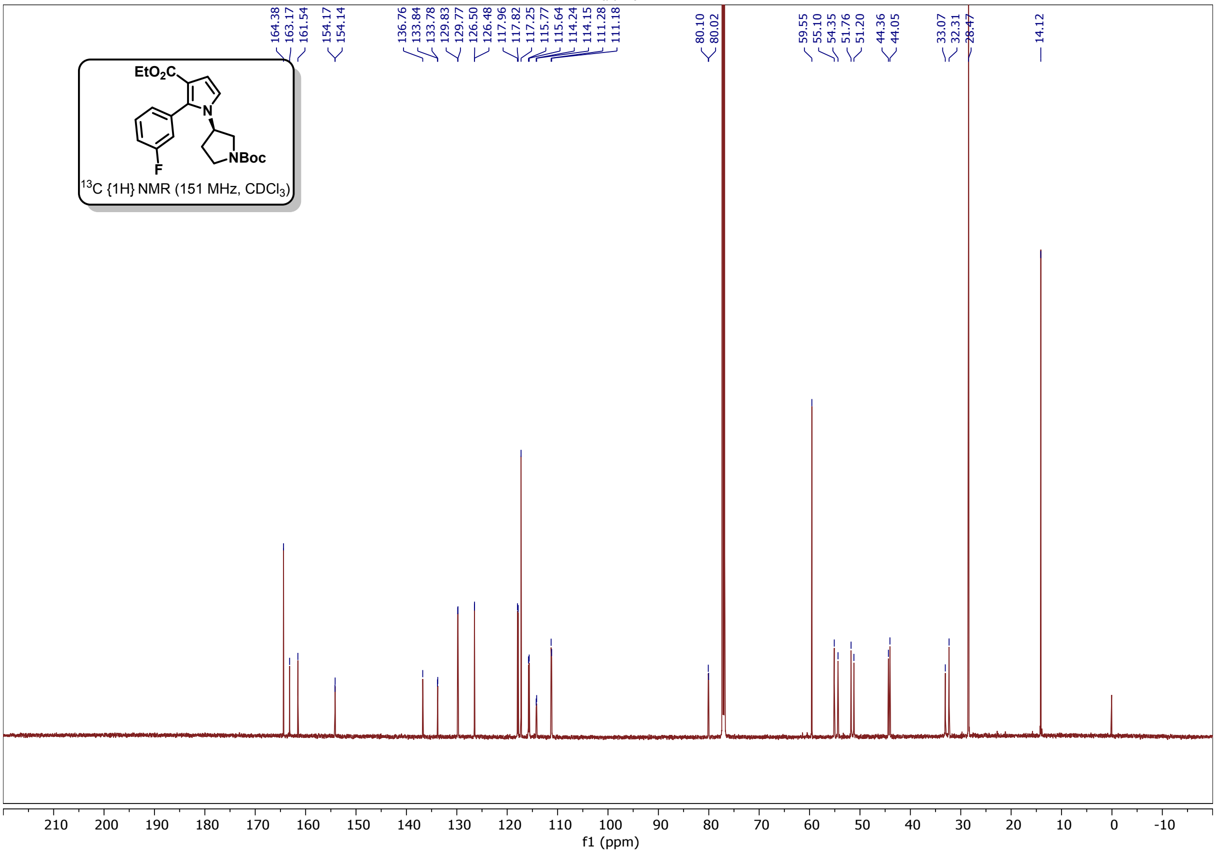

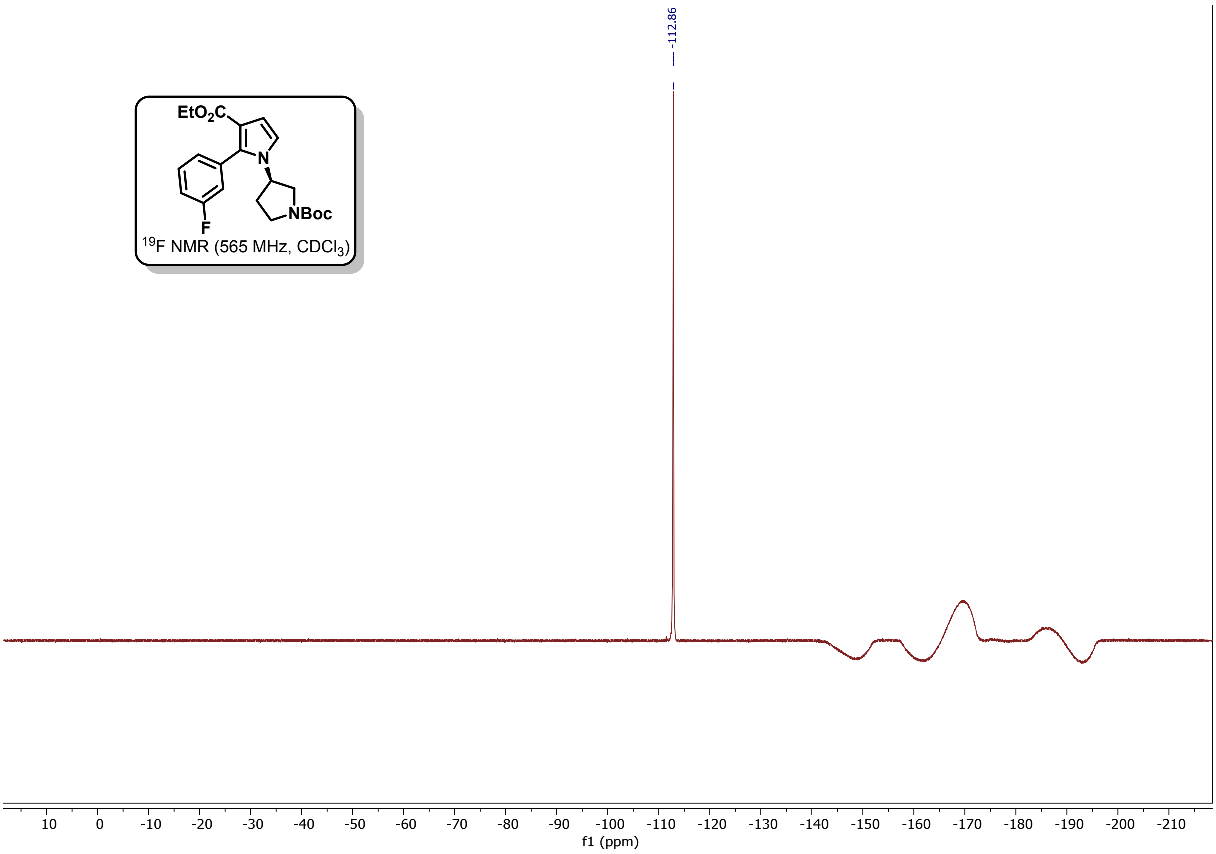
-112.86
EtO2C
F
NBoc
19F NMR (565 MHz, CDCl3)
10 0 -10 -20 -30 -40 -50 -60 -70 -80 -90 -100 -110 -120 -130 -140 -150 -160 -170 -180 -190 -200 -210
f1 (ppm)

Ethyl 1-(1-(4-bromophenyl)ethyl)-2-(3-fluorophenyl)-1H-pyrrole-3-carboxylate (4ao):

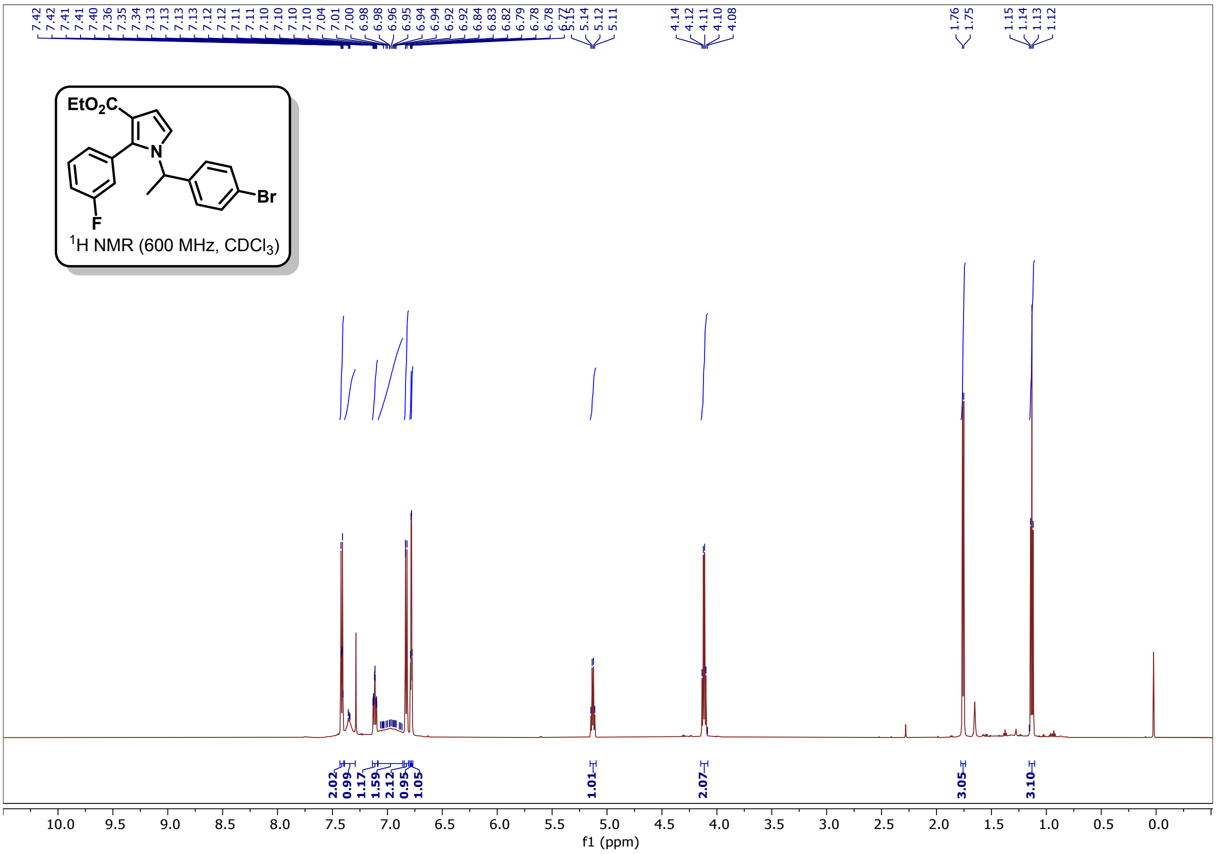

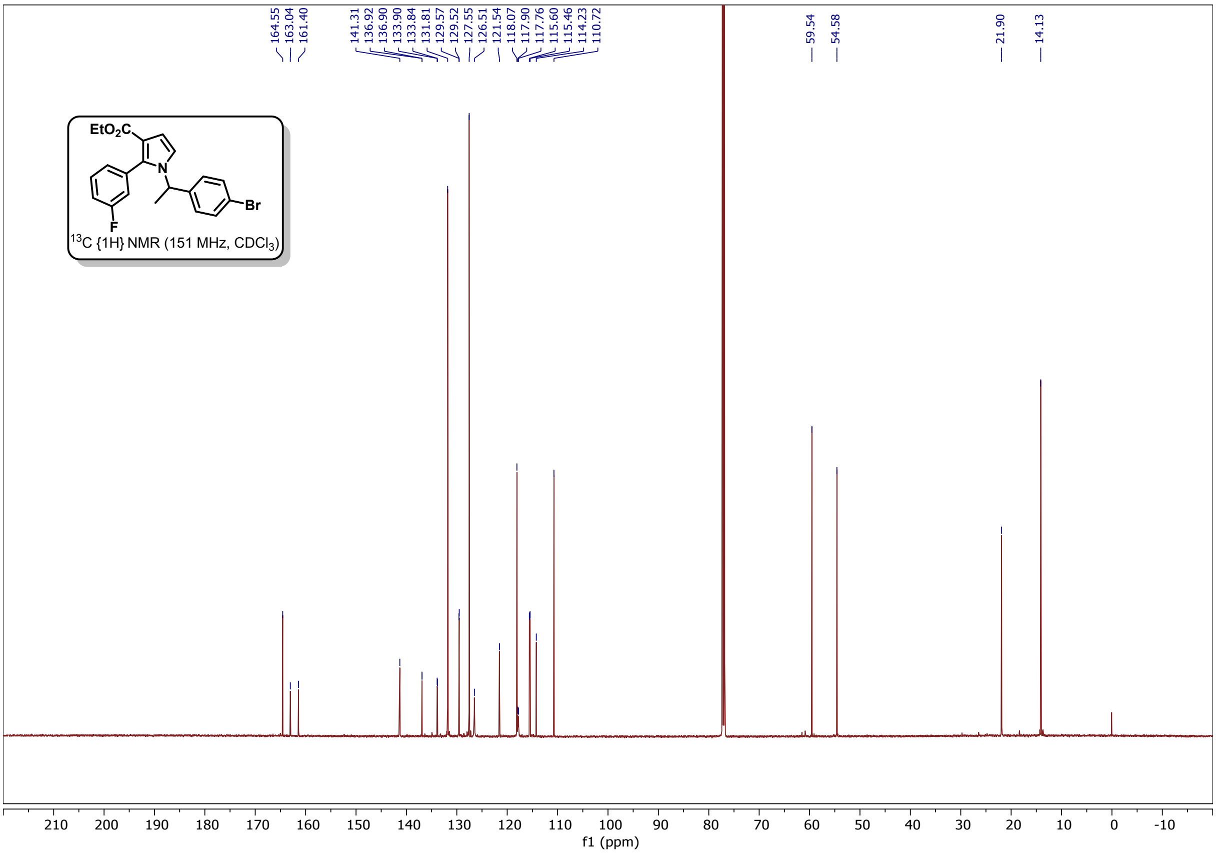

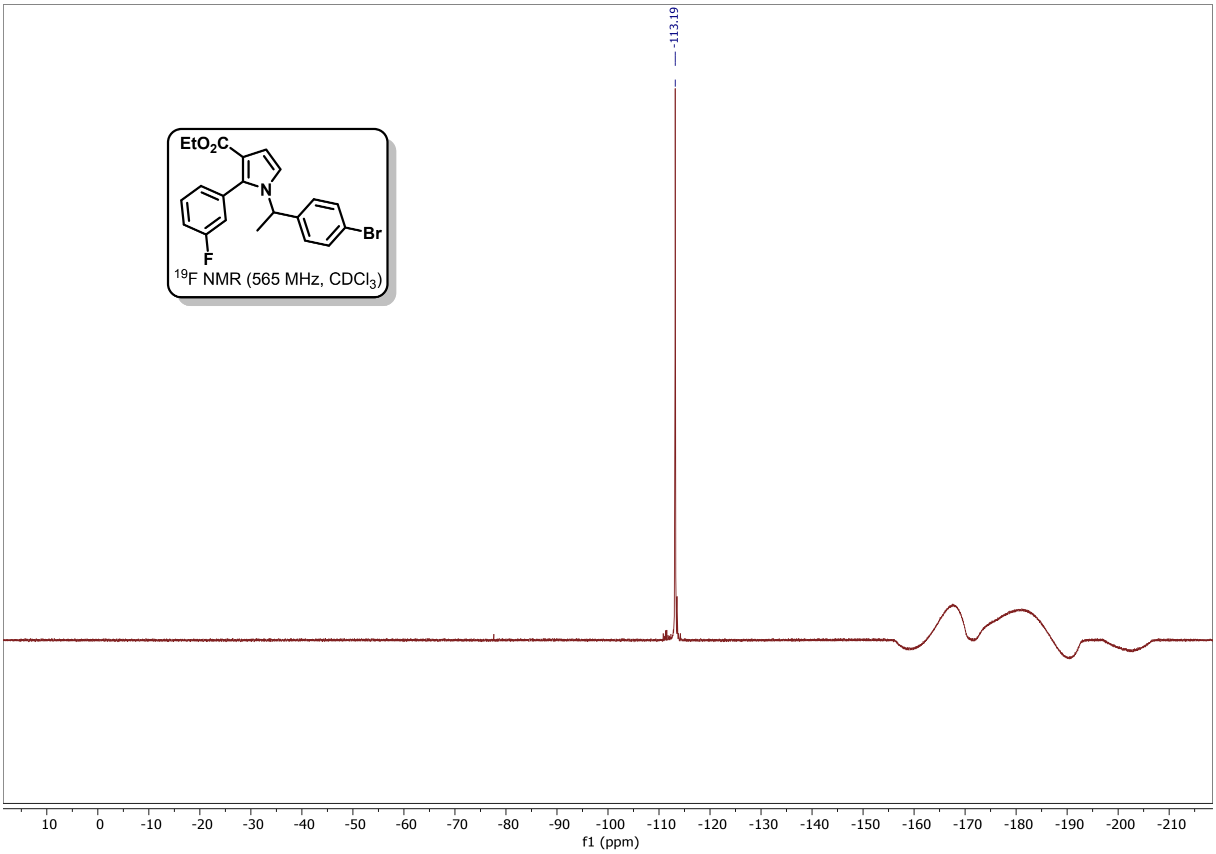

-113.19
EtO2C
N
Br
F
19F NMR (565 MHz, CDCl3)
10 0 -10 -20 -30 -40 -50 -60 -70 -80 -90 -100 -110 -120 -130 -140 -150 -160 -170 -180 -190 -200 -210
f1 (ppm)

Ethyl 2-(3-fluorophenyl)-1-phenyl-1H-pyrrole-3-carboxylate (4ap):

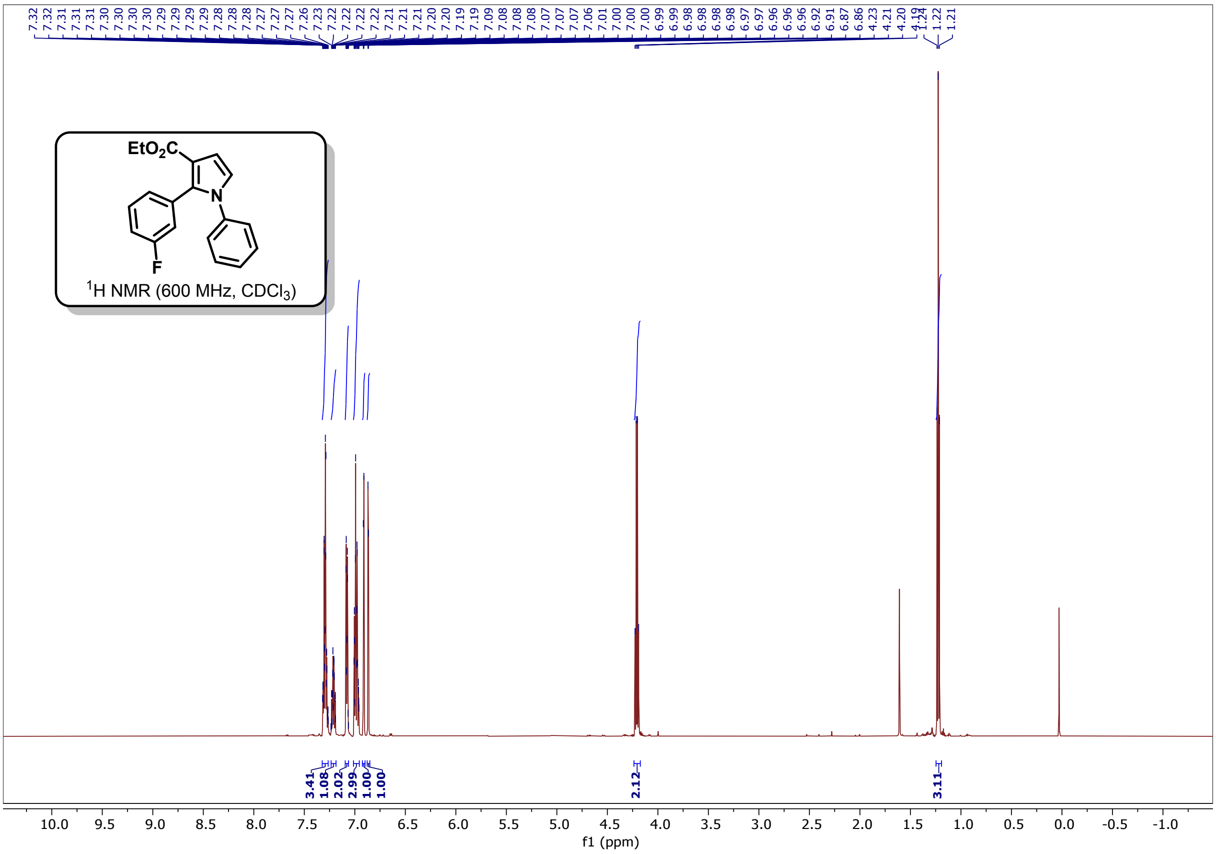

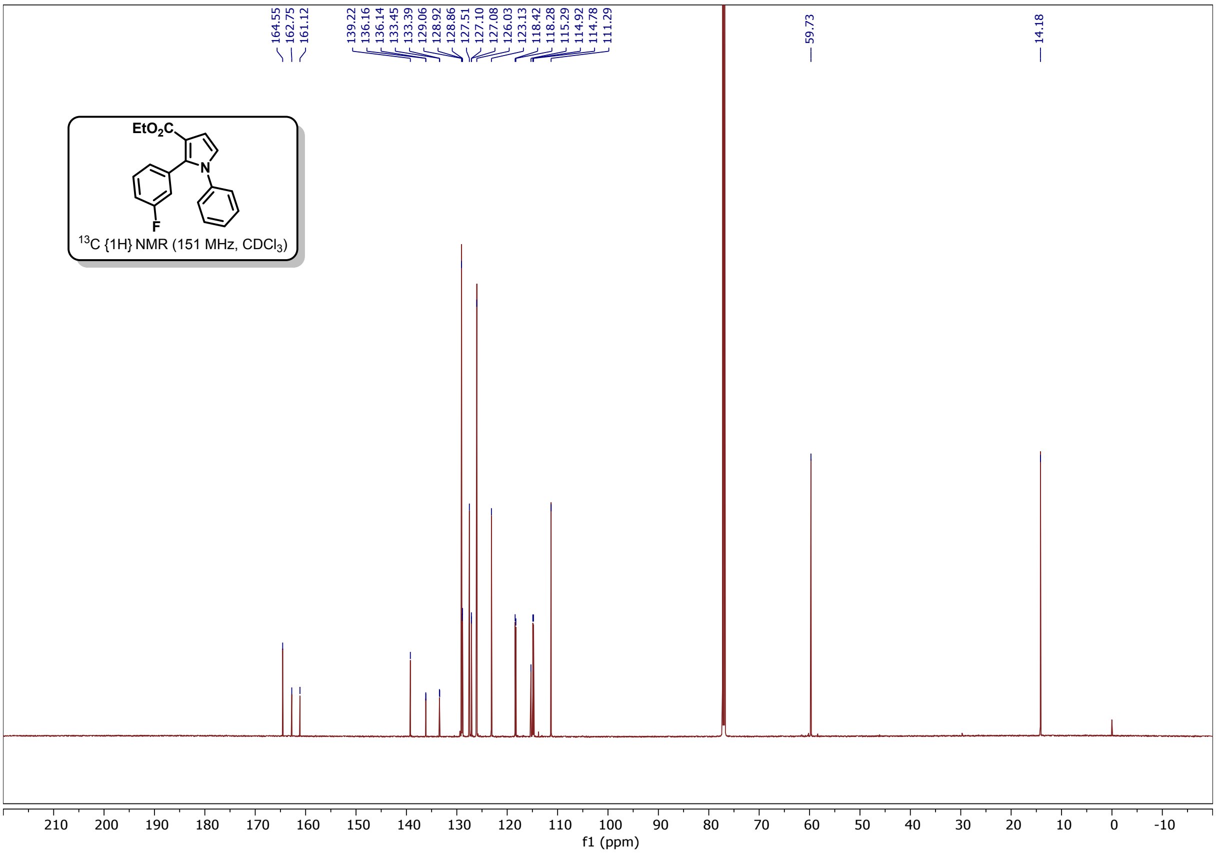

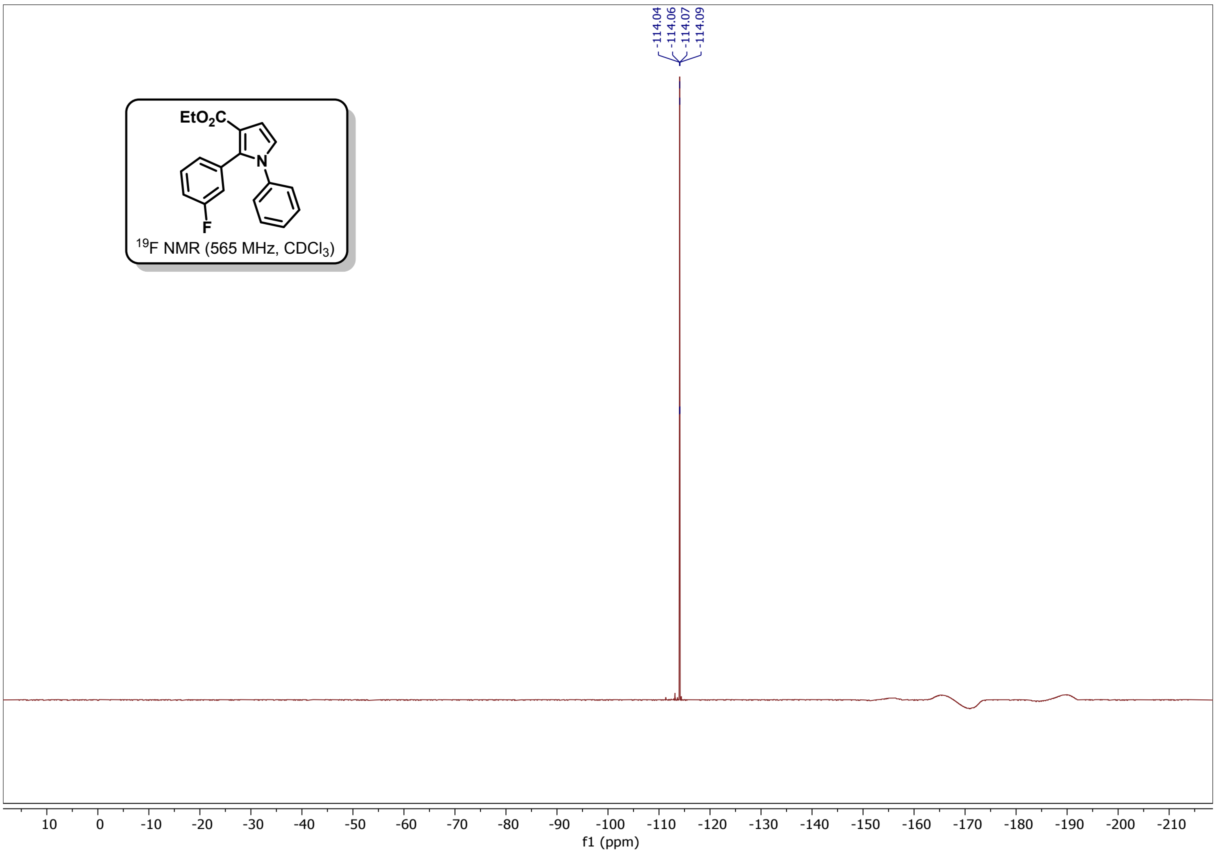

-114.04
-114.06
-114.07
-114.09
EtO2C
N
F
19F NMR (565 MHz, CDCl3)
10 0 -10 -20 -30 -40 -50 -60 -70 -80 -90 -100 -110 -120 -130 -140 -150 -160 -170 -180 -190 -200 -210
f1 (ppm)

Ethyl 2-(3-fluorophenyl)-1-(3-isopropylphenyl)-1H-pyrrole-3-carboxylate (4aq):

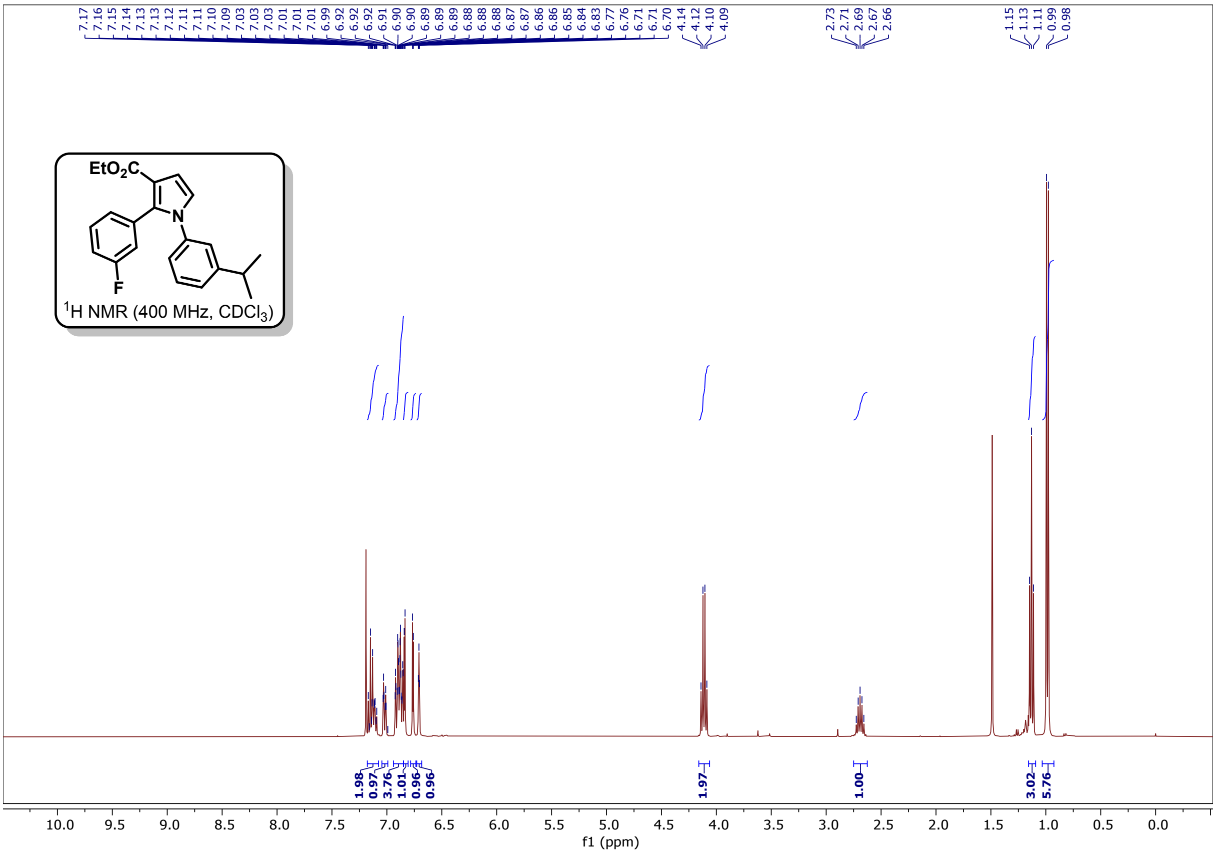

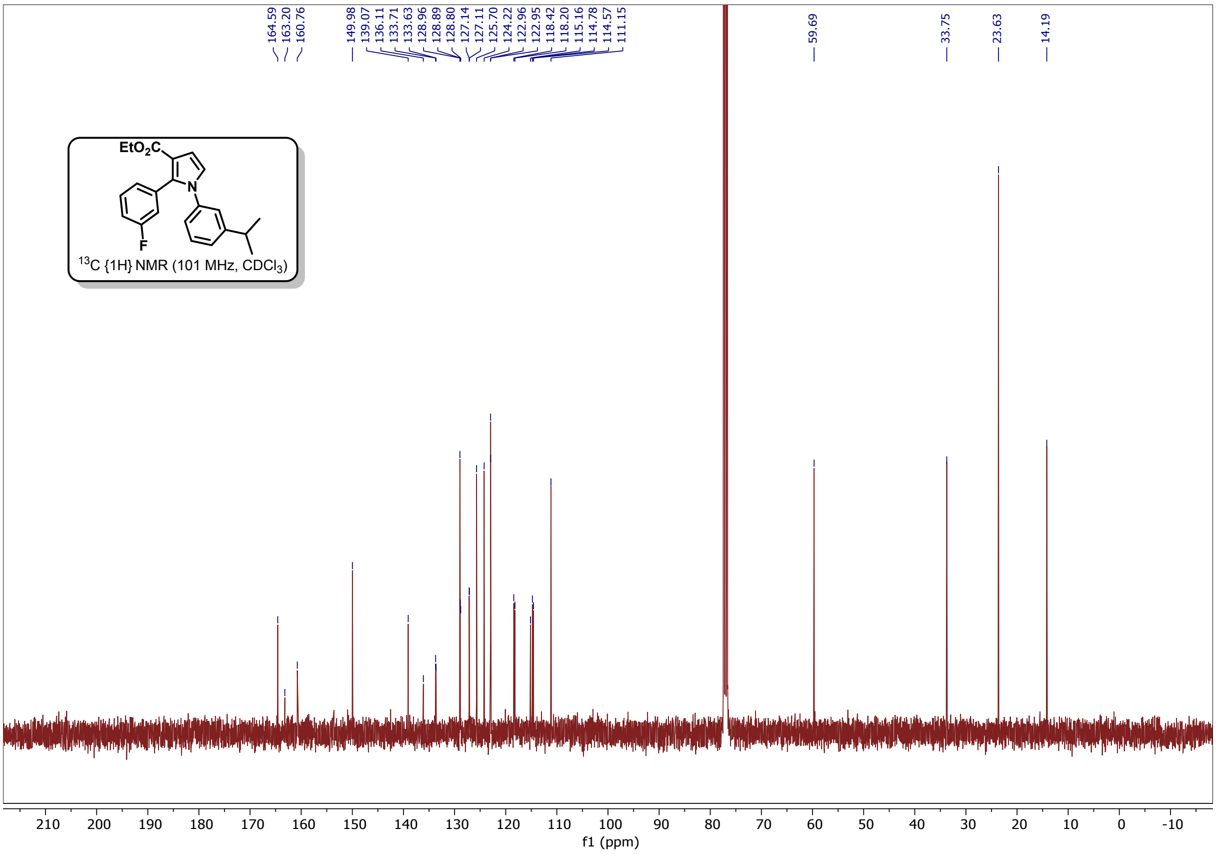

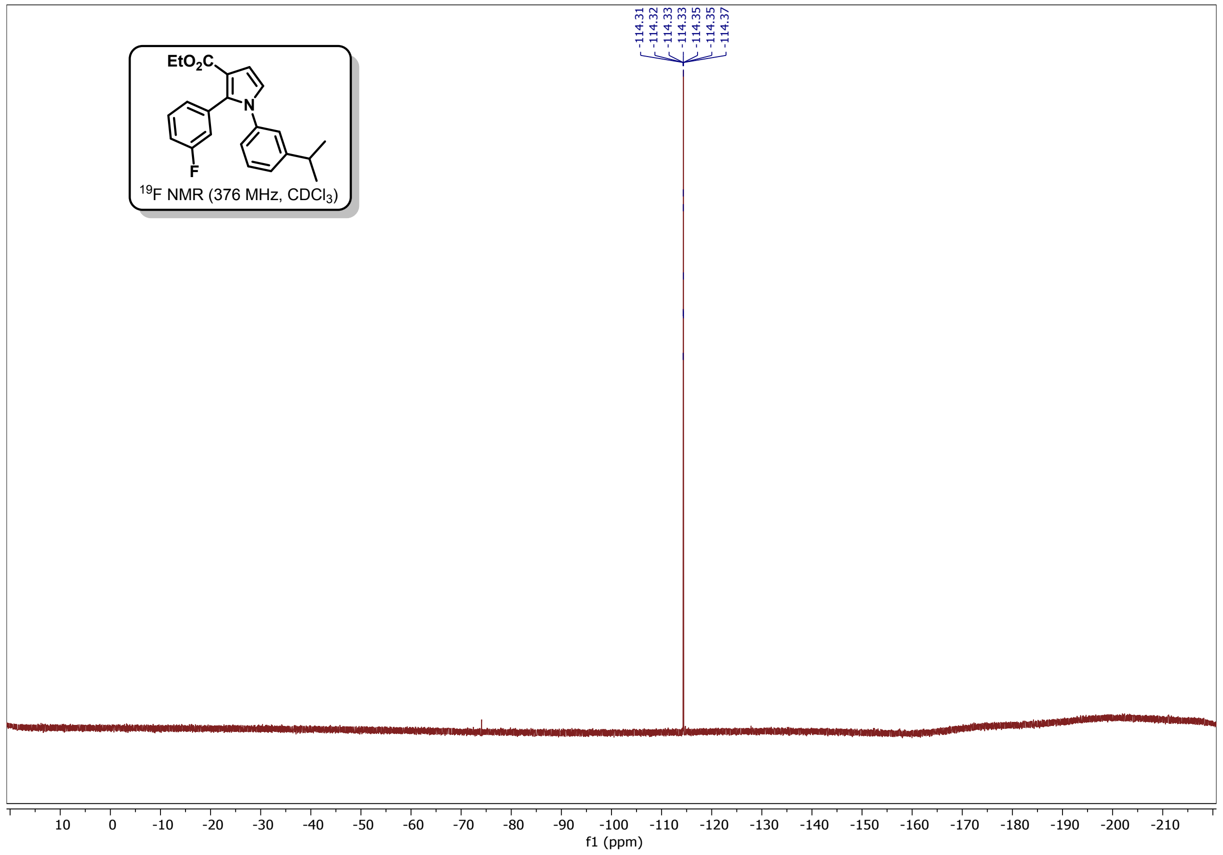

EtO2C
N
F
19F NMR (376 MHz, CDCl3)
-114.31
-114.32
-114.33
-114.33
-114.35
-114.35
-114.37
10 0 -10 -20 -30 -40 -50 -60 -70 -80 -90 -100 -110 -120 -130 -140 -150 -160 -170 -180 -190 -200 -210
f1 (ppm)

Ethyl 2-(3-fluorophenyl)-1-(5,6,7,8-tetrahydronaphthalen-1-yl)-1H-pyrrole-3-carboxylate (4ar):

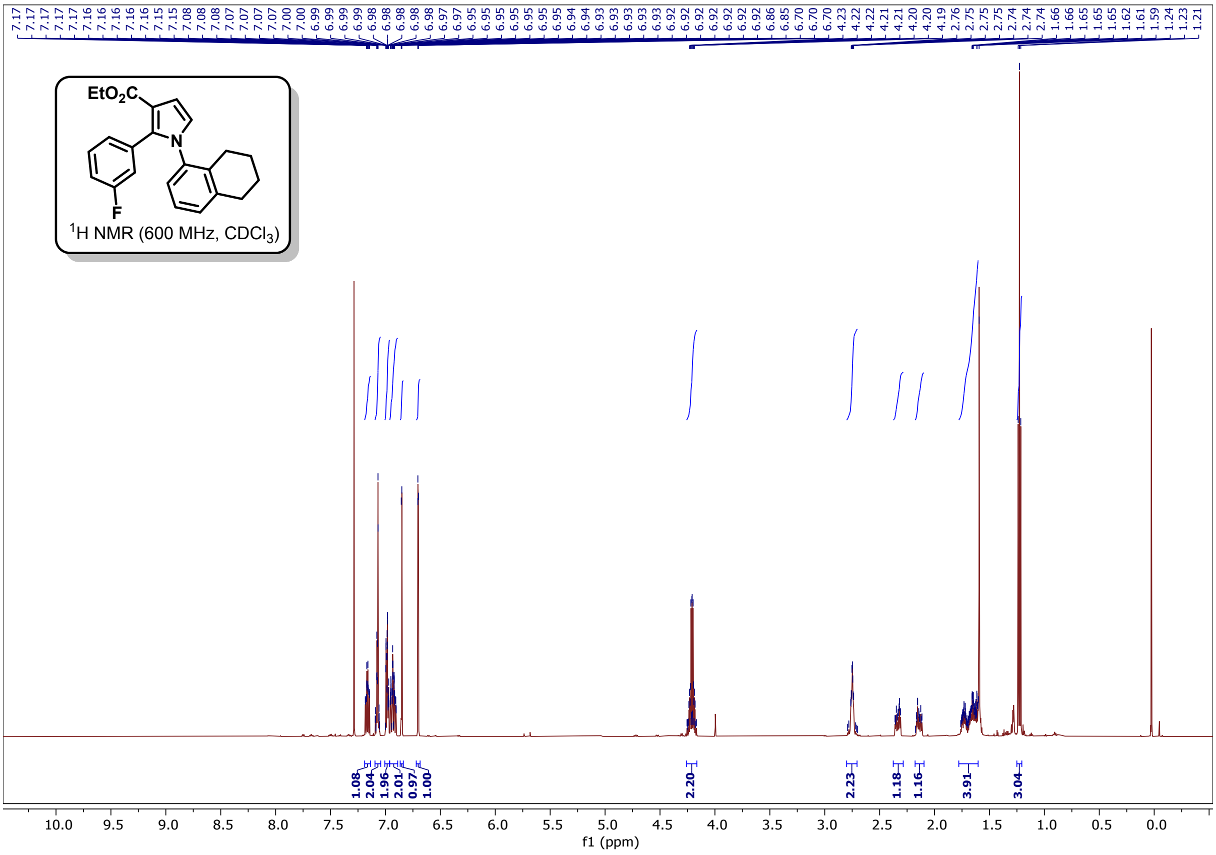

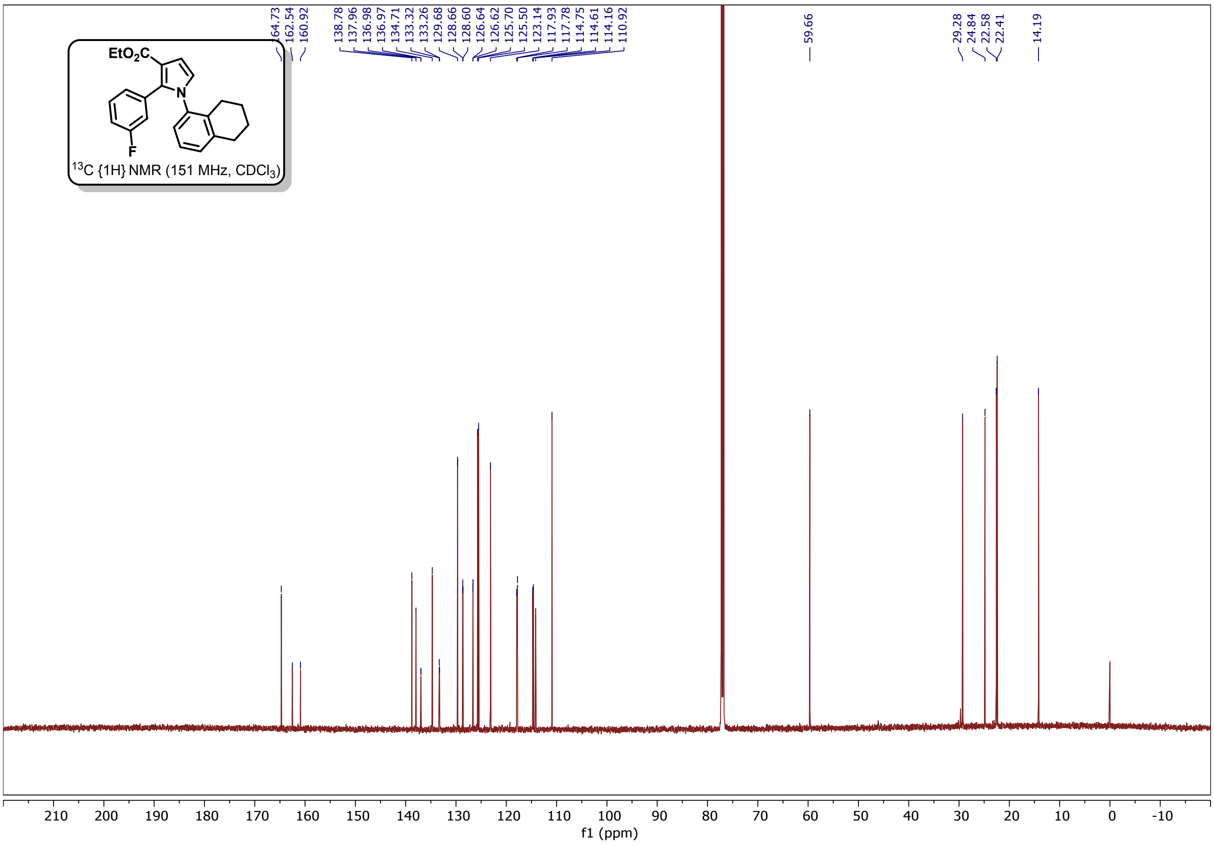

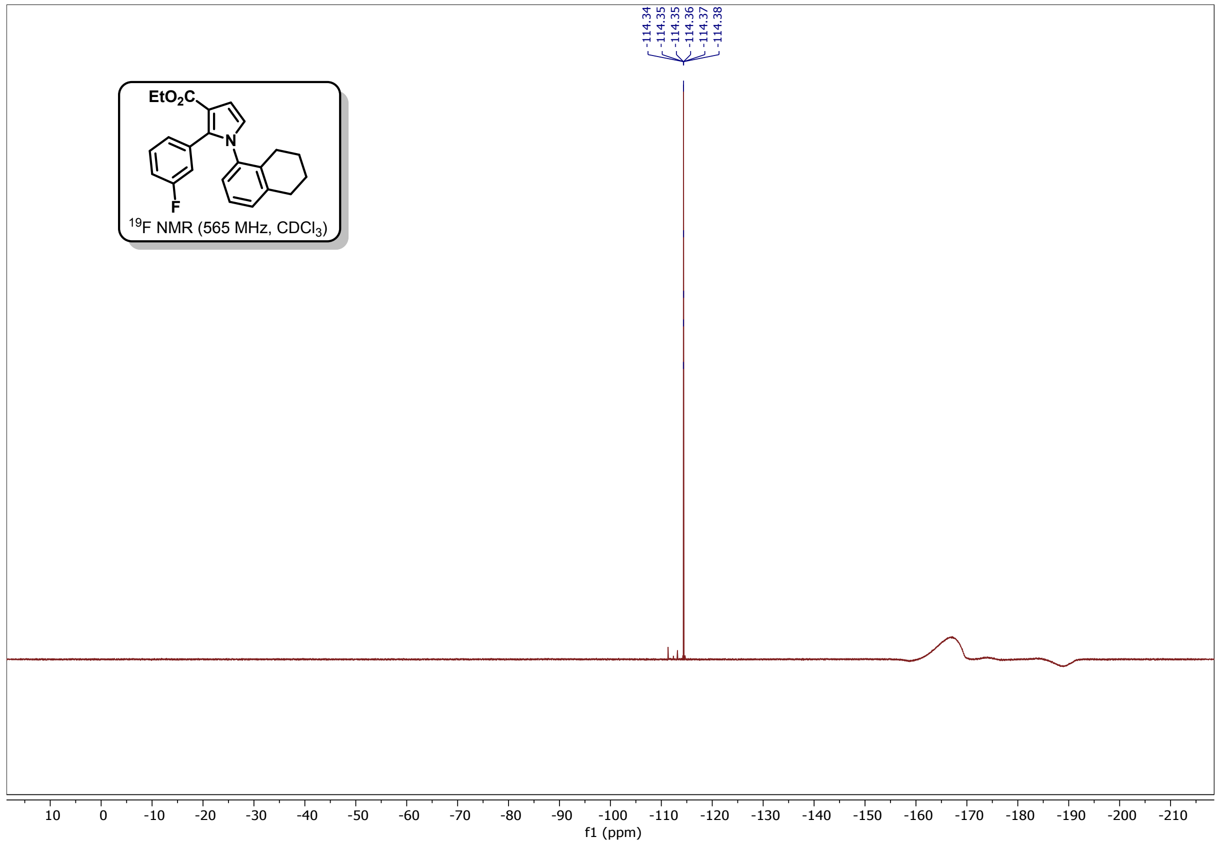

-114.34
-114.35
-114.35
-114.36
-114.37
-114.38
EtO2C
N
F
19F NMR (565 MHz, CDCl3)
10 0 -10 -20 -30 -40 -50 -60 -70 -80 -90 -100 -110 -120 -130 -140 -150 -160 -170 -180 -190 -200 -210
f1 (ppm)

Ethyl 1-phenethyl-2-phenyl-1H-pyrrole-3-carboxylate (4ba):

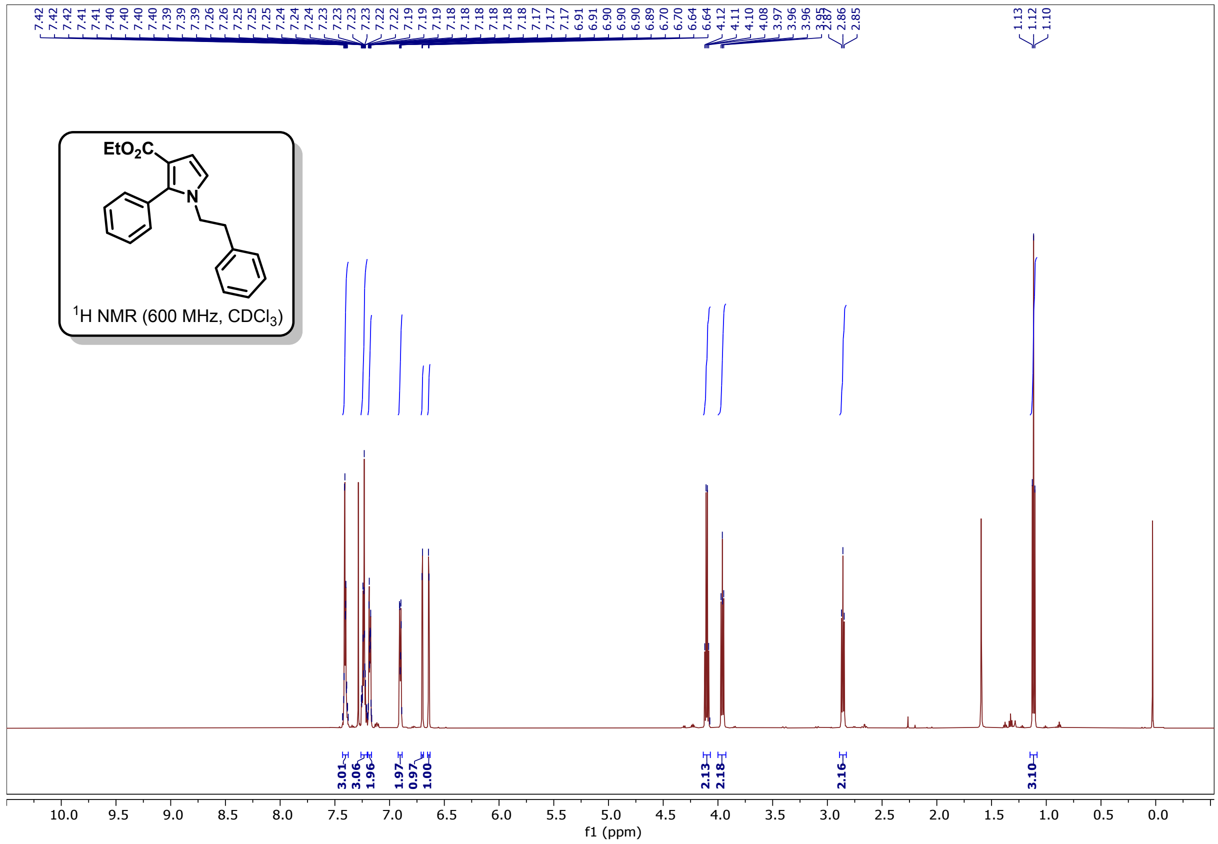

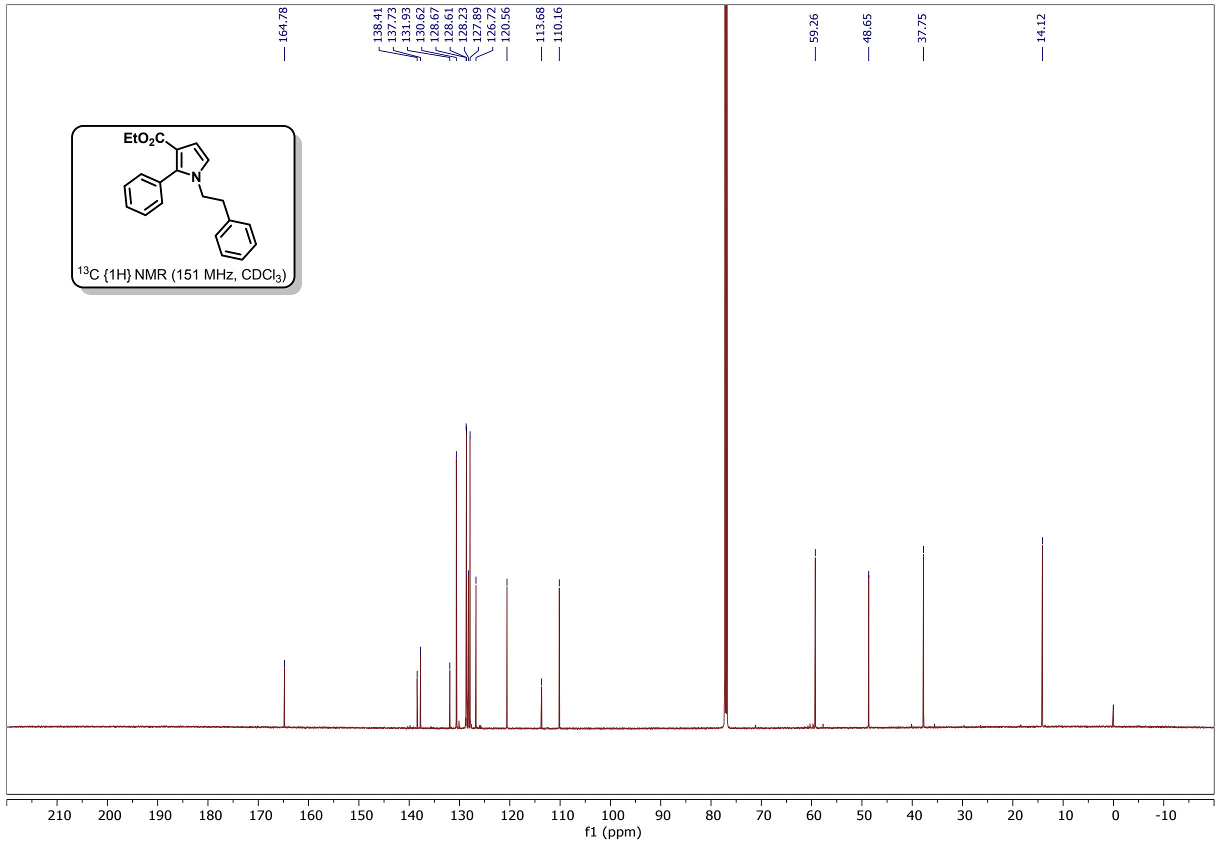

Ethyl 2-(4-nitrophenyl)-1-phenethyl-1H-pyrrole-3-carboxylate (4ca):

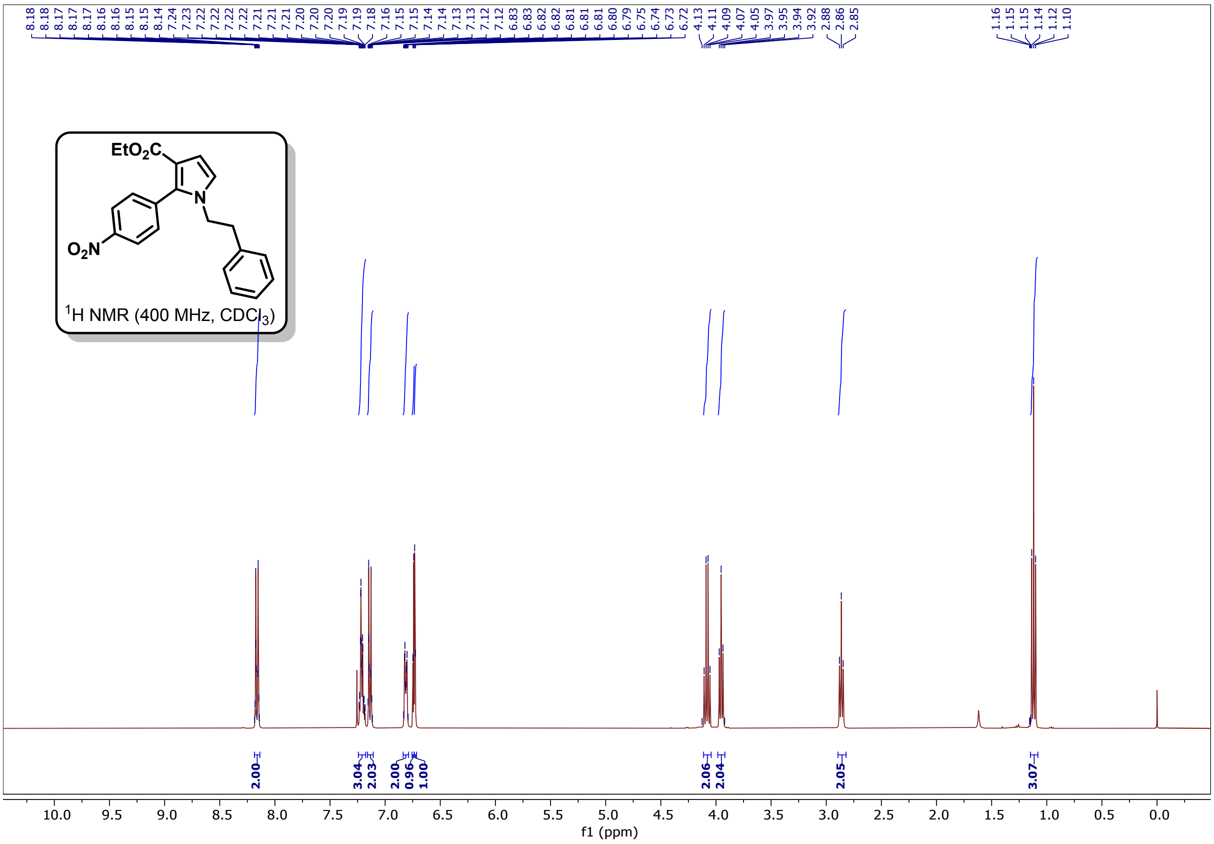

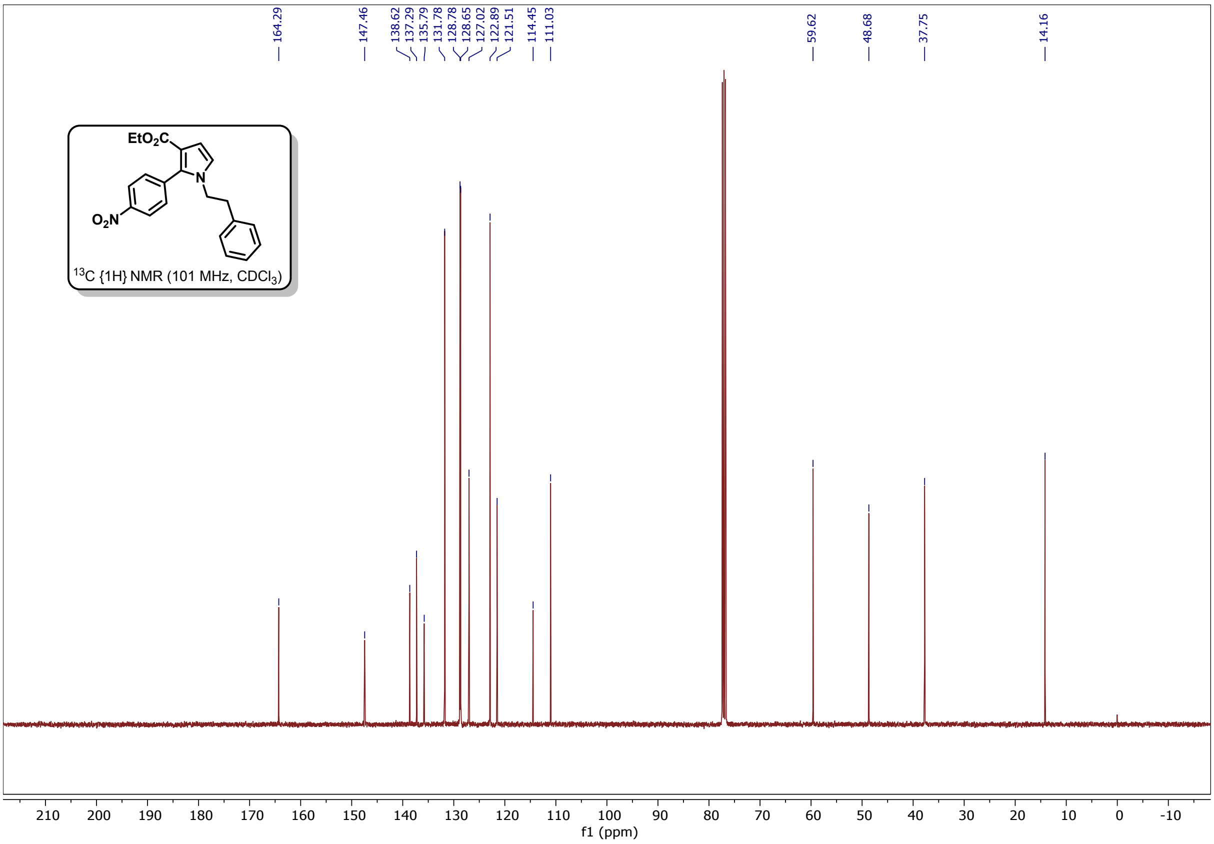

Ethyl 1-phenethyl-2-(4-(trifluoromethoxy)phenyl)-1H-pyrrole-3-carboxylate (4da):

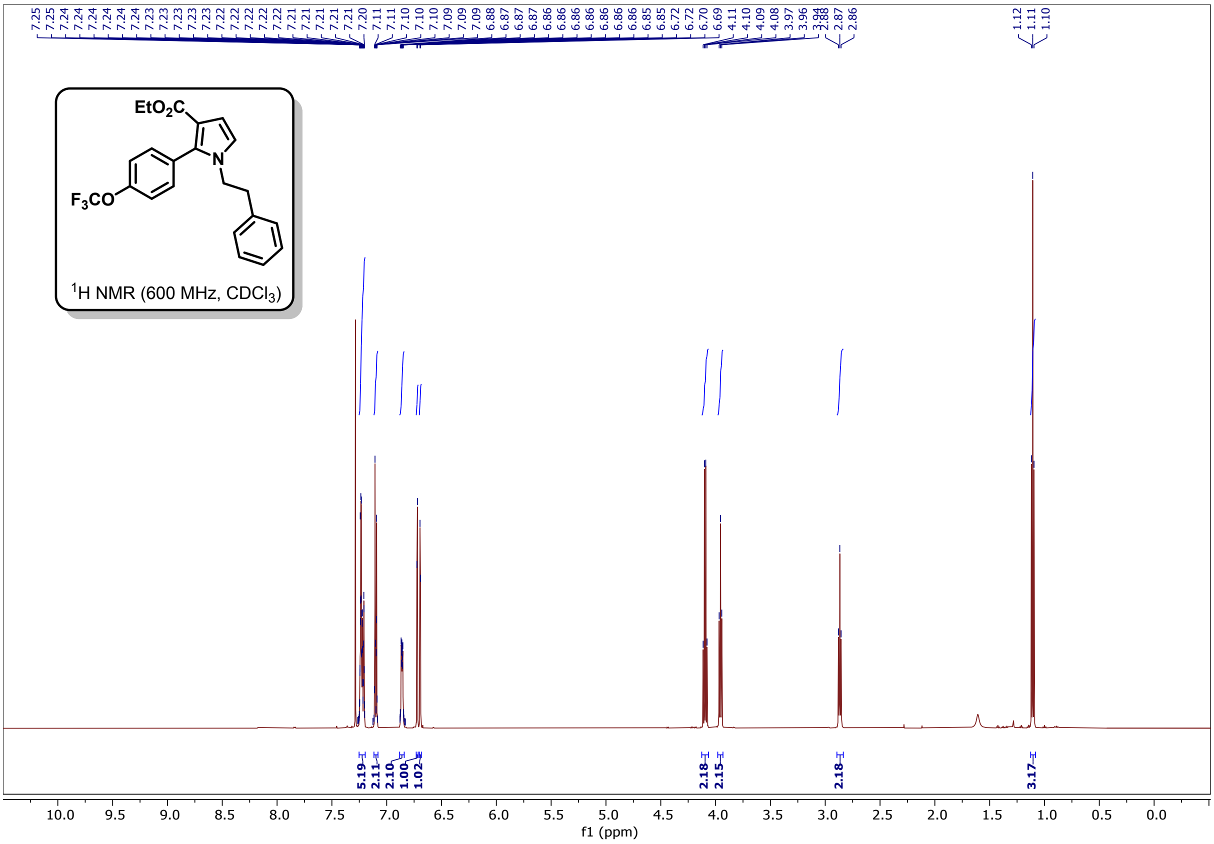

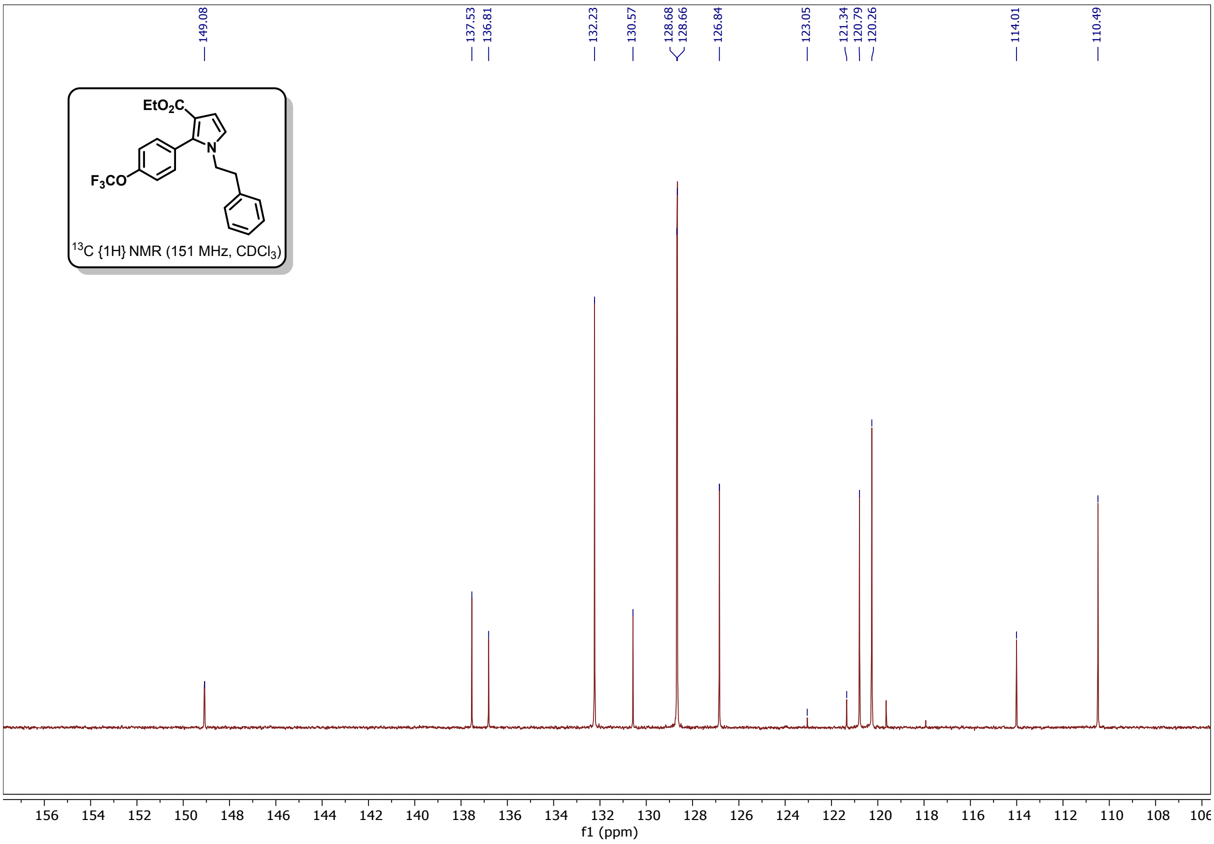

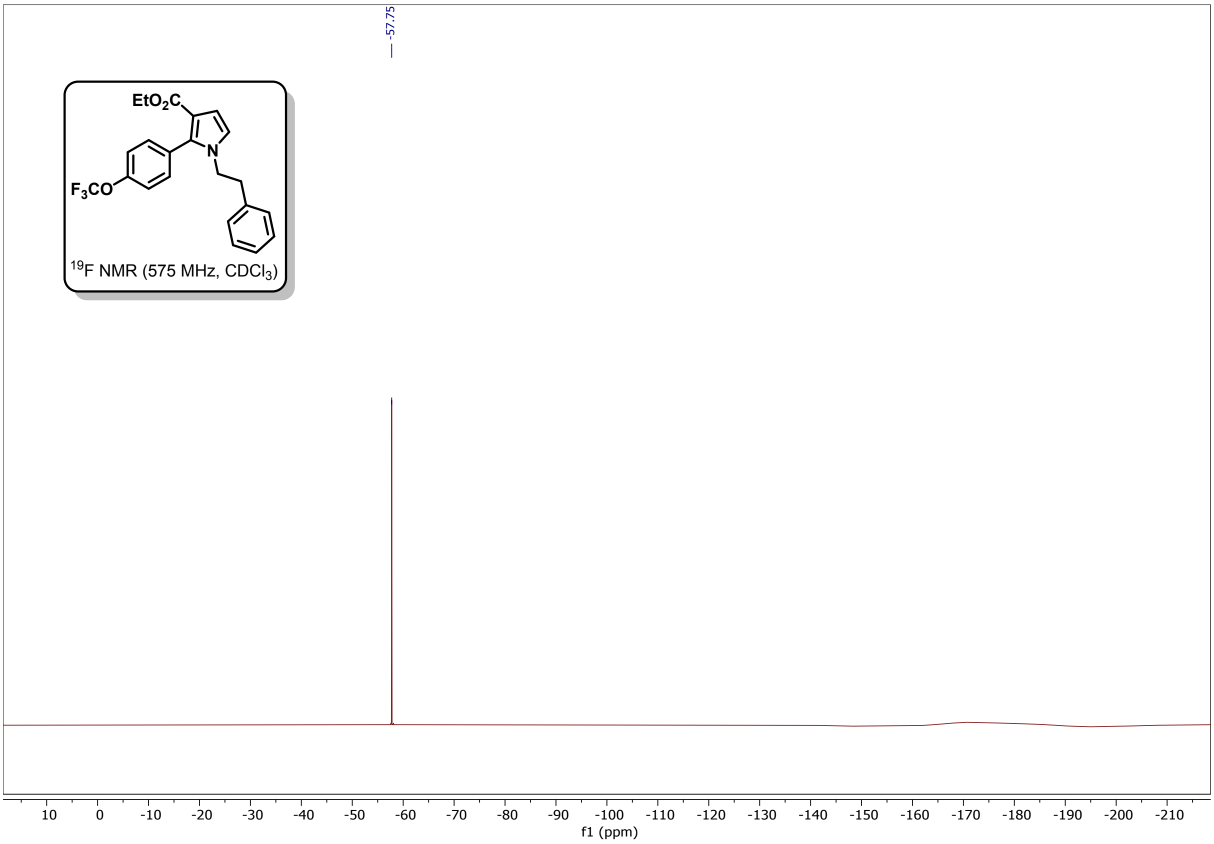

-57.75
EtO2C
F3CO
N
19F NMR (575 MHz, CDCl3)
10 0 -10 -20 -30 -40 -50 -60 -70 -80 -90 -100 -110 -120 -130 -140 -150 -160 -170 -180 -190 -200 -210
f1 (ppm)

Ethyl 2-(4-(N,N-dipropylsulfamoyl)phenyl)-1-phenethyl-1H-pyrrole-3-carboxylate (4ea):

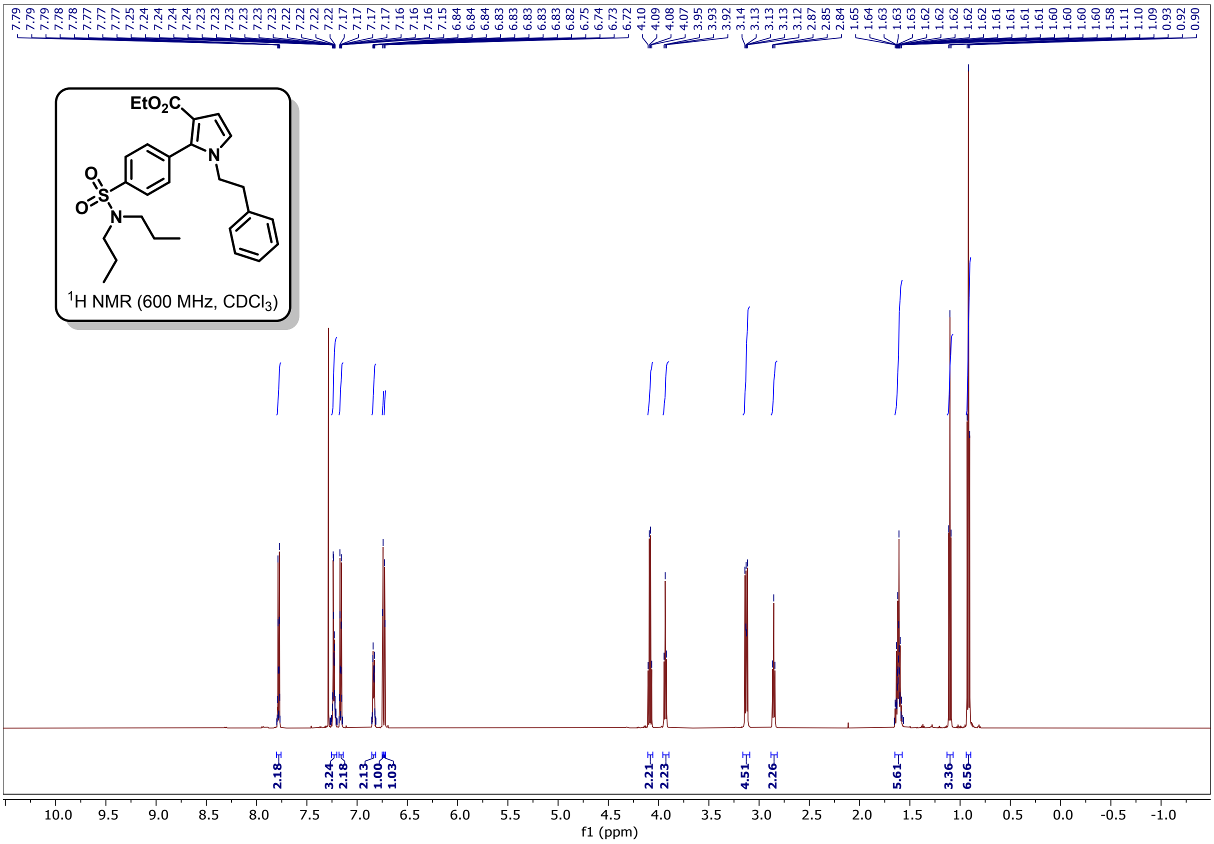

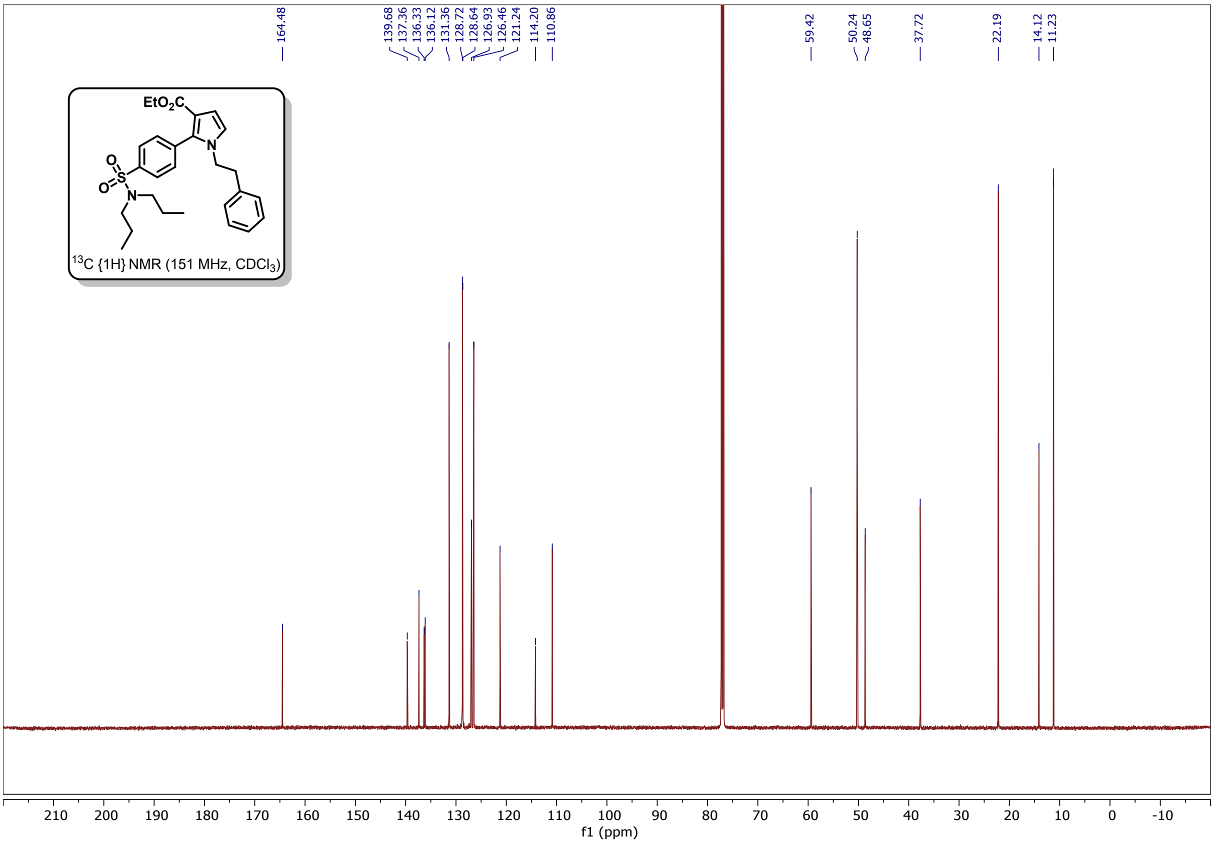

Ethyl 2-(naphthalen-1-yl)-1-phenethyl-1H-pyrrole-3-carboxylate (4fa):

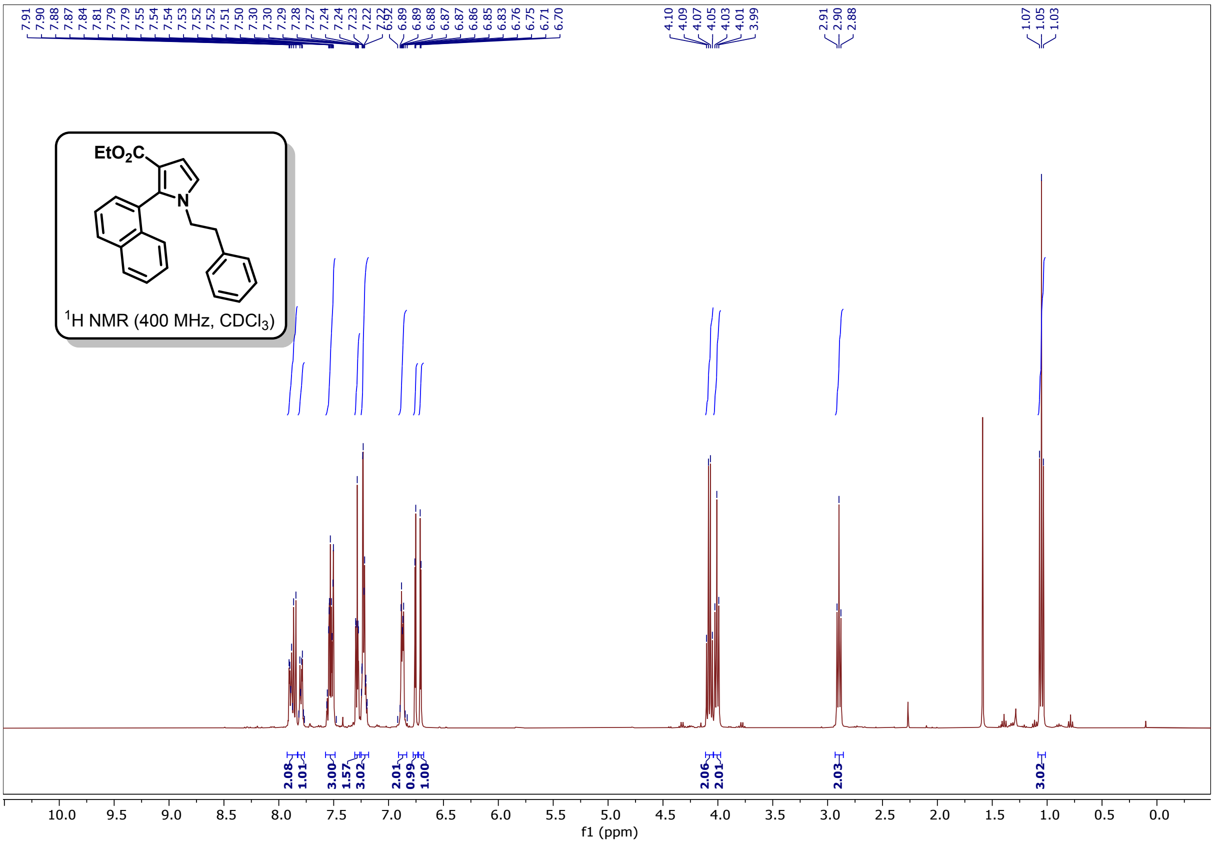

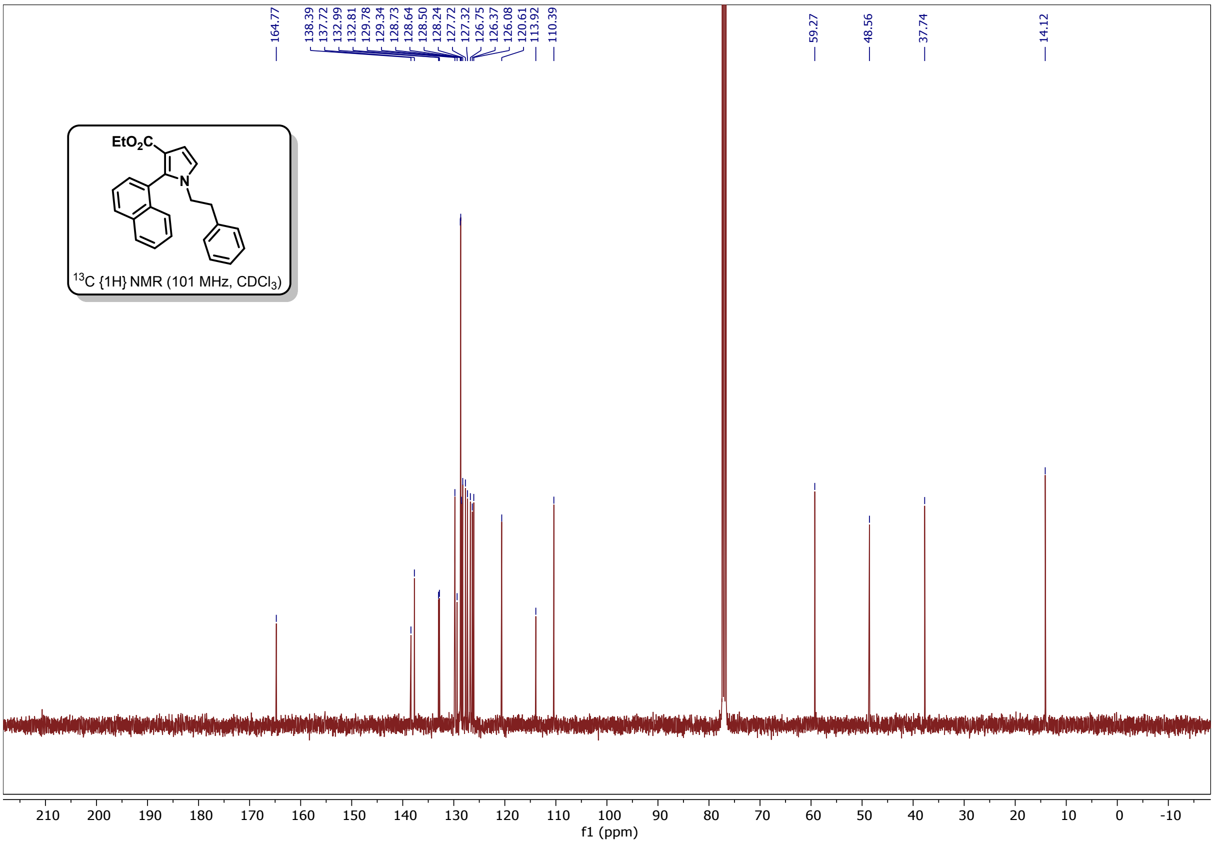

Methyl 1-phenethyl-2-(6-(trifluoromethyl)pyridin-3-yl)-1H-pyrrole-3-carboxylate (4ga):

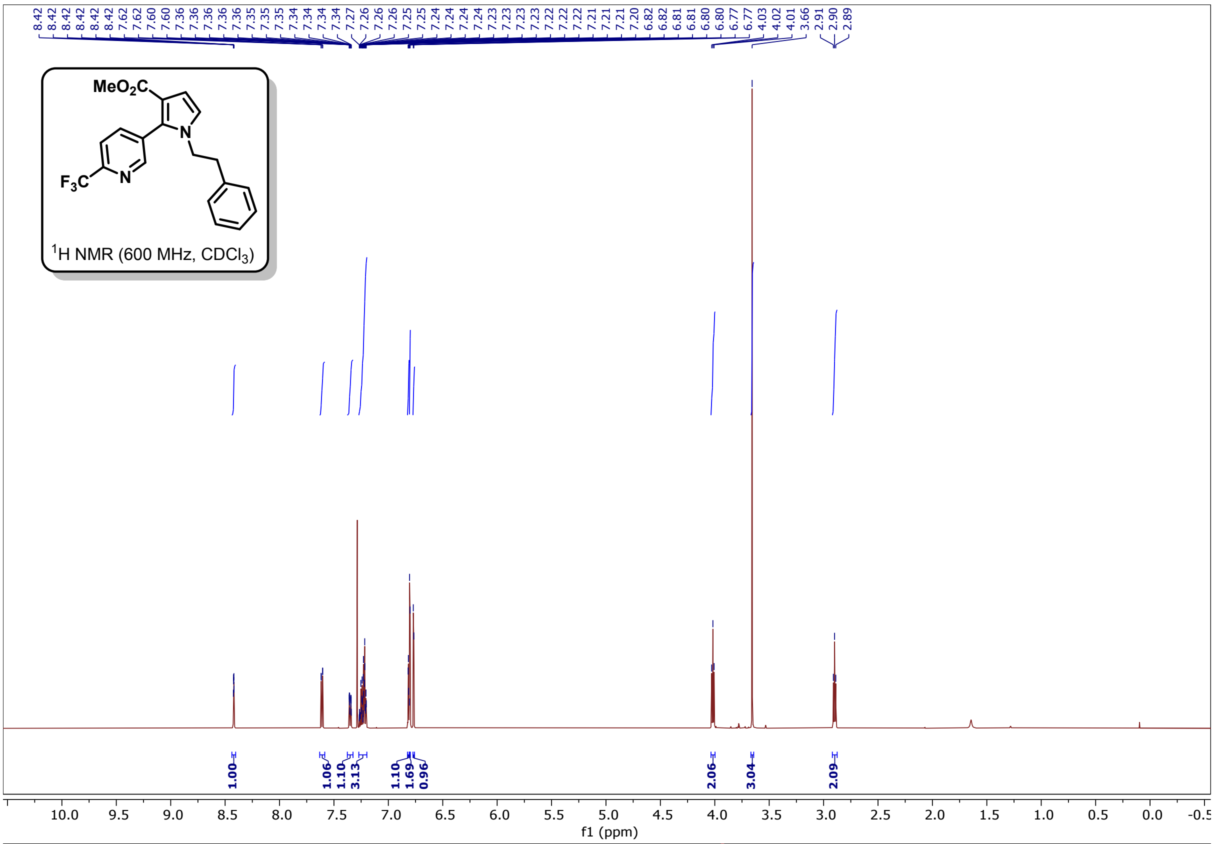

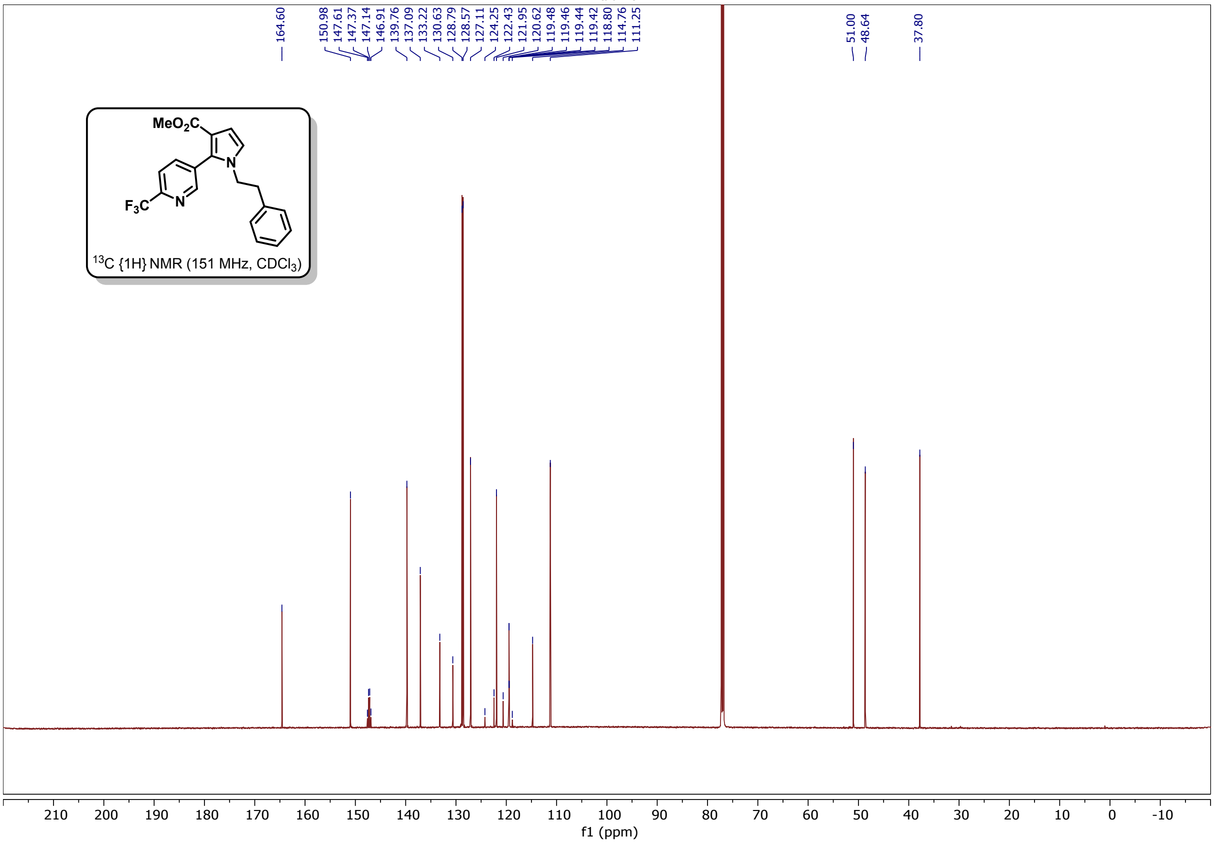

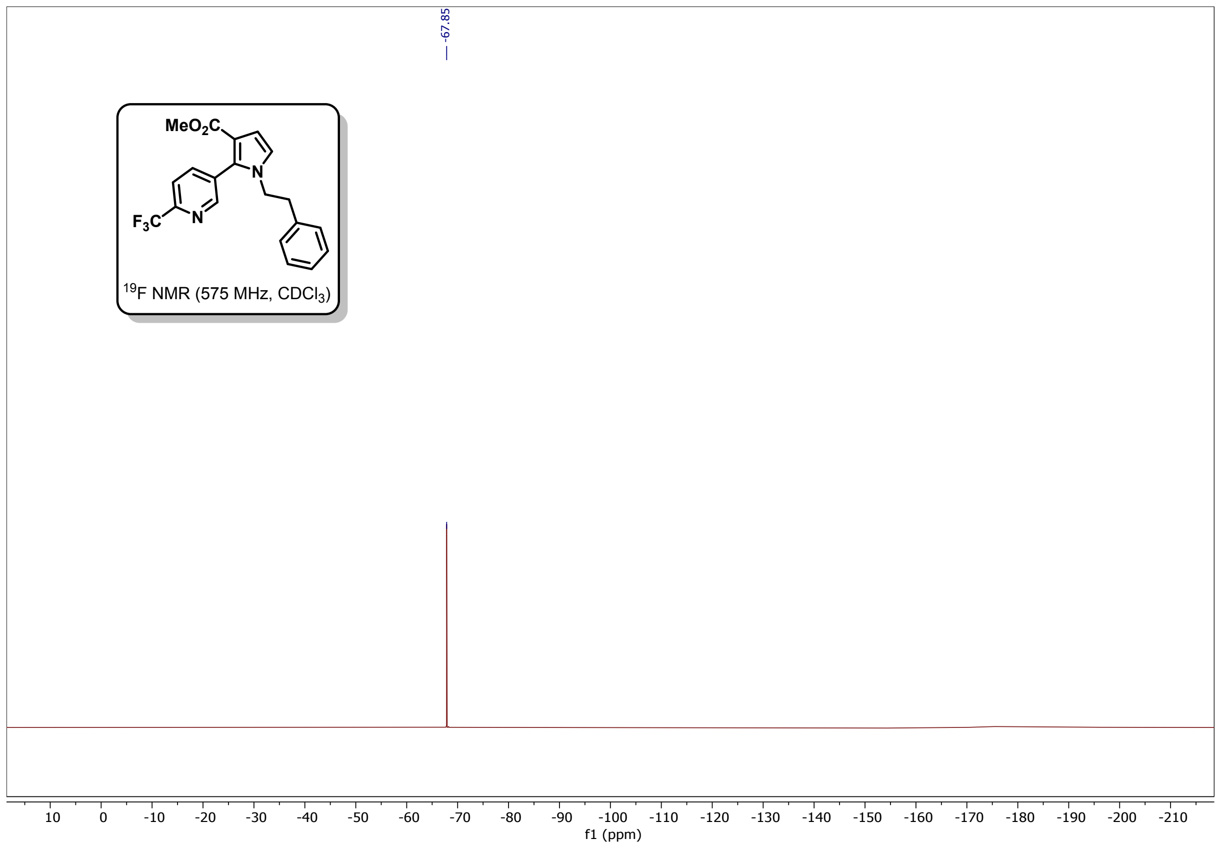
-67.85
MeO2C
N
F3C
N
19F NMR (575 MHz, CDCl3)
10
0
-10
-20
-30
-40
-50
-60
-70
-80
-90
-100
-110
-120
-130
-140
-150
-160
-170
-180
-190
-200
-210
f1 (ppm)

Methyl 1-phenethyl-2-(2-(trifluoromethyl)pyridin-4-yl)-1H-pyrrole-3-carboxylate (4ha):

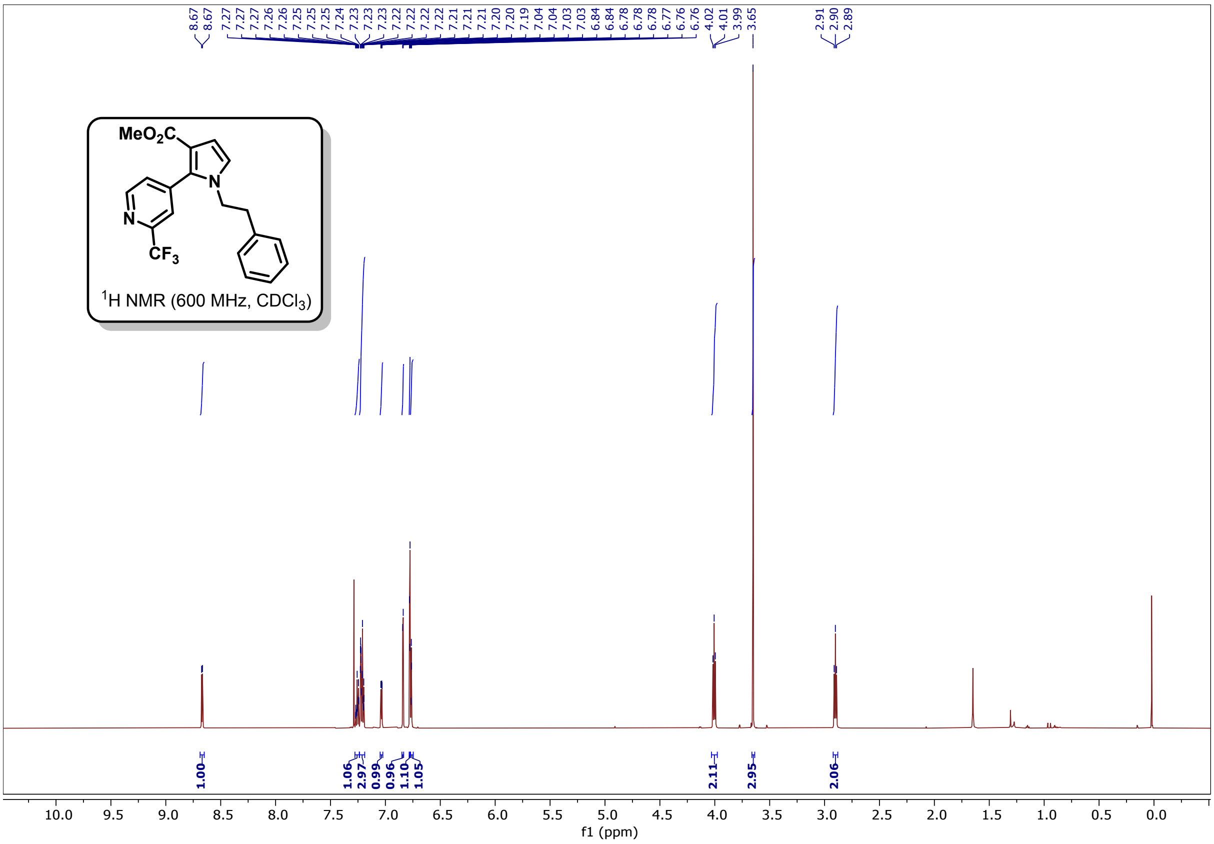

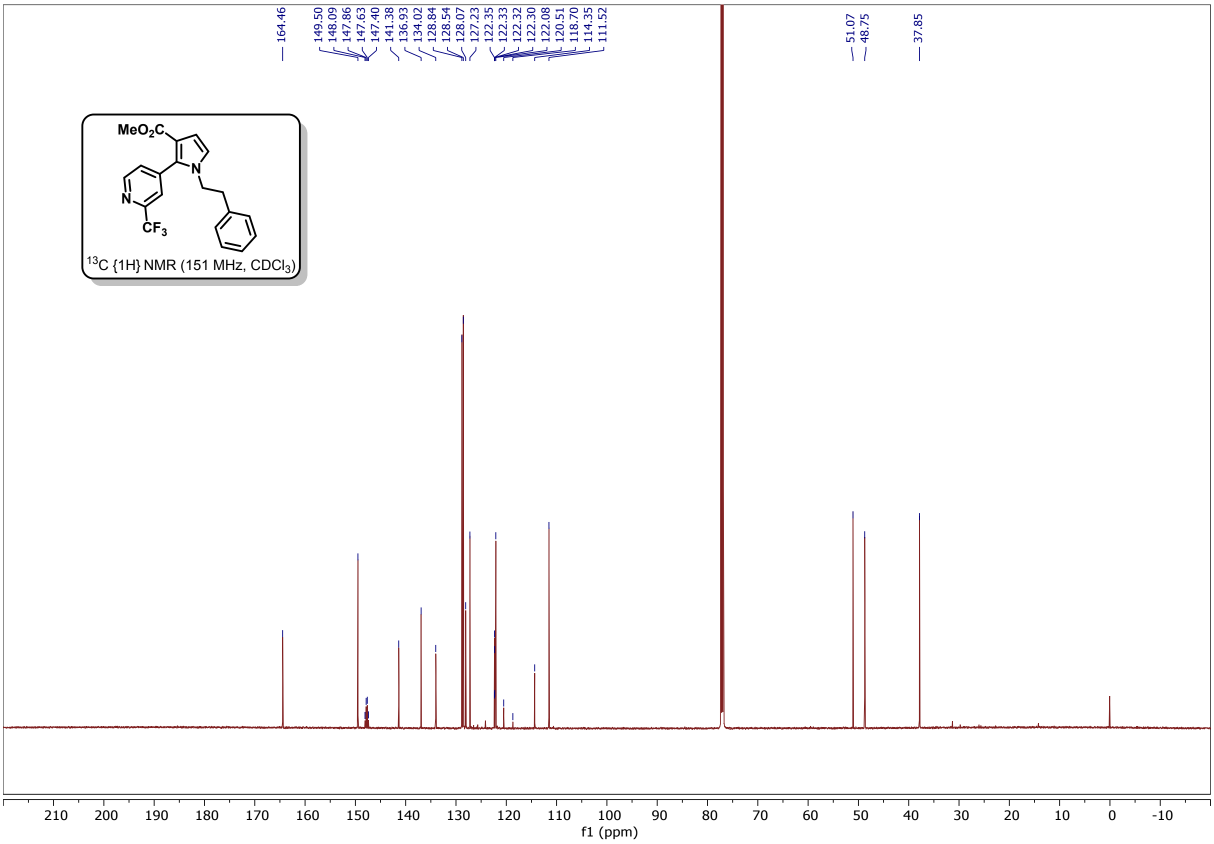

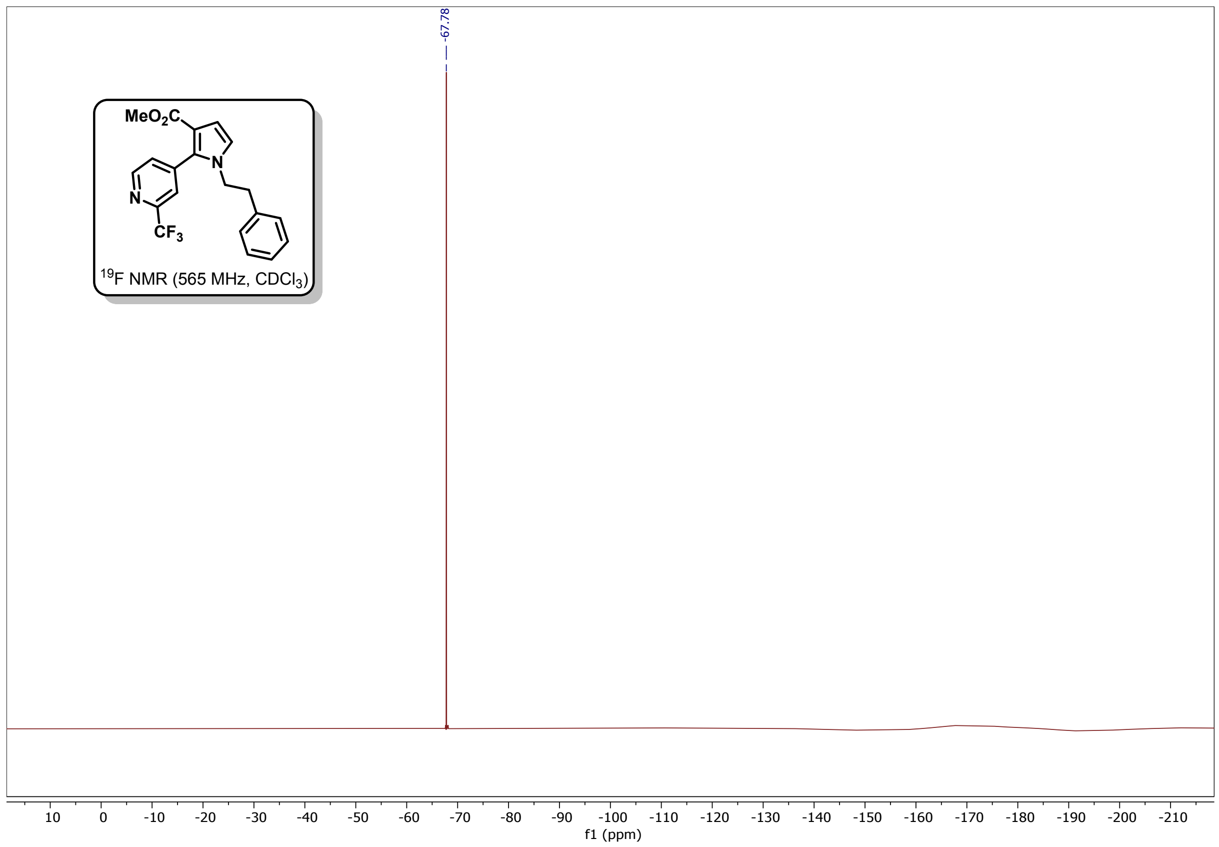
-67.78
MeO2C
N
N
CF3
19F NMR (565 MHz, CDCl3)
10 0 -10 -20 -30 -40 -50 -60 -70 -80 -90 -100 -110 -120 -130 -140 -150 -160 -170 -180 -190 -200 -210
f1 (ppm)

Ethyl 2-(6-chloropyridin-3-yl)-1-phenethyl-1H-pyrrole-3-carboxylate (4ia):

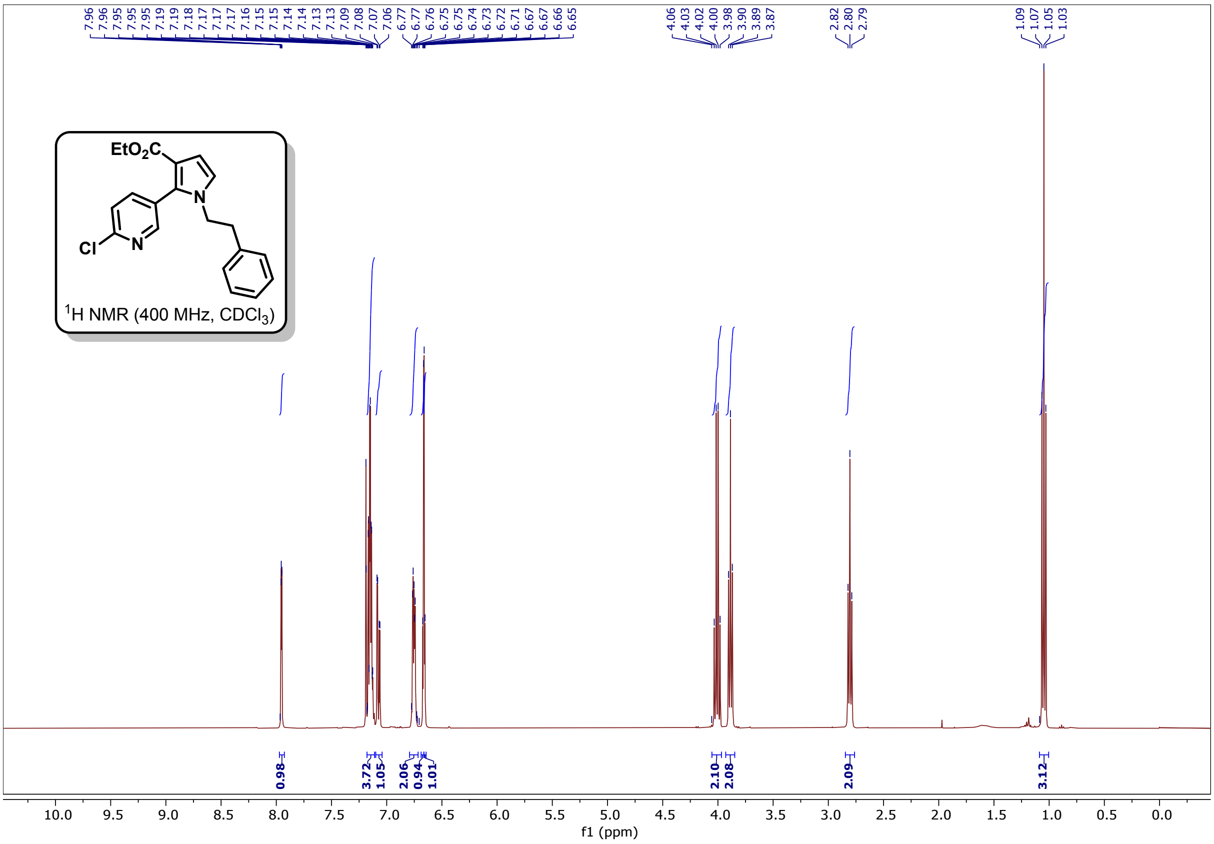

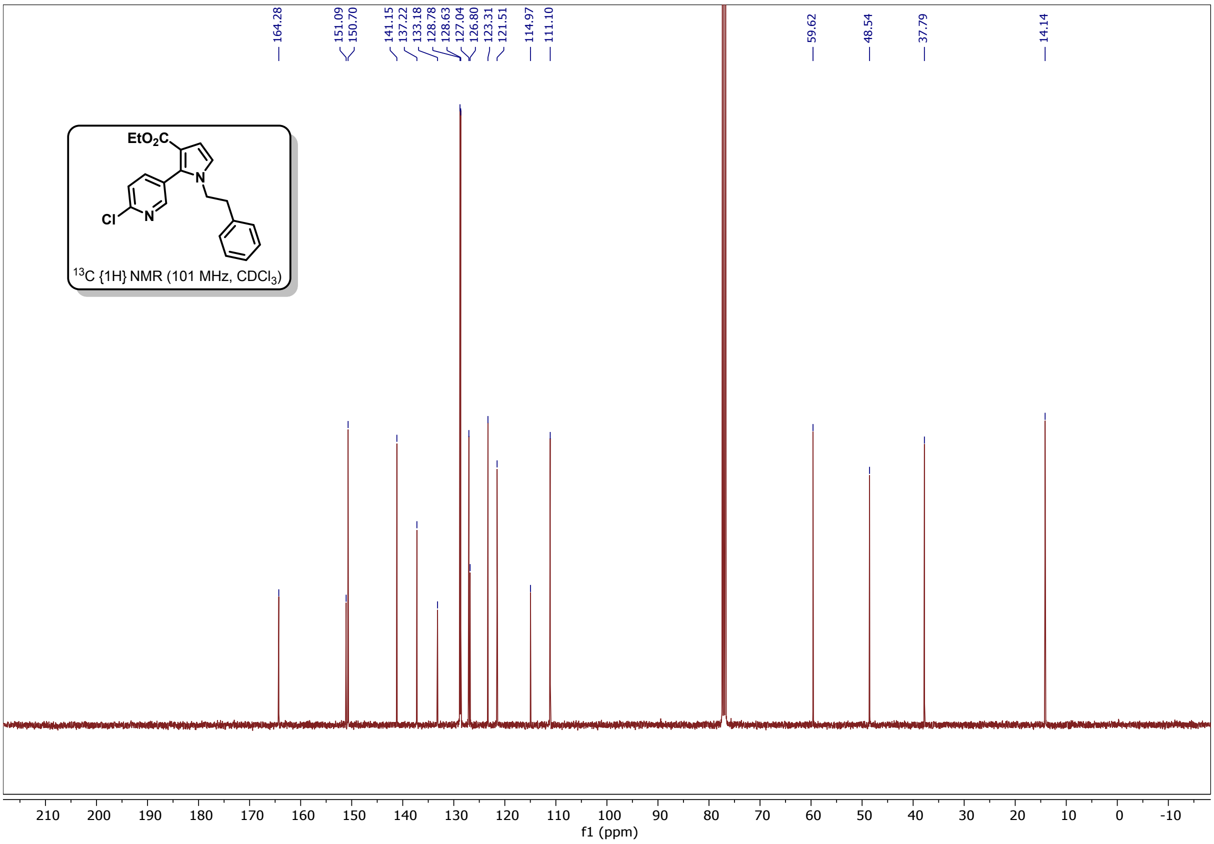

Ethyl 1-phenethyl-2-(quinoxalin-6-yl)-1H-pyrrole-3-carboxylate (4ja):

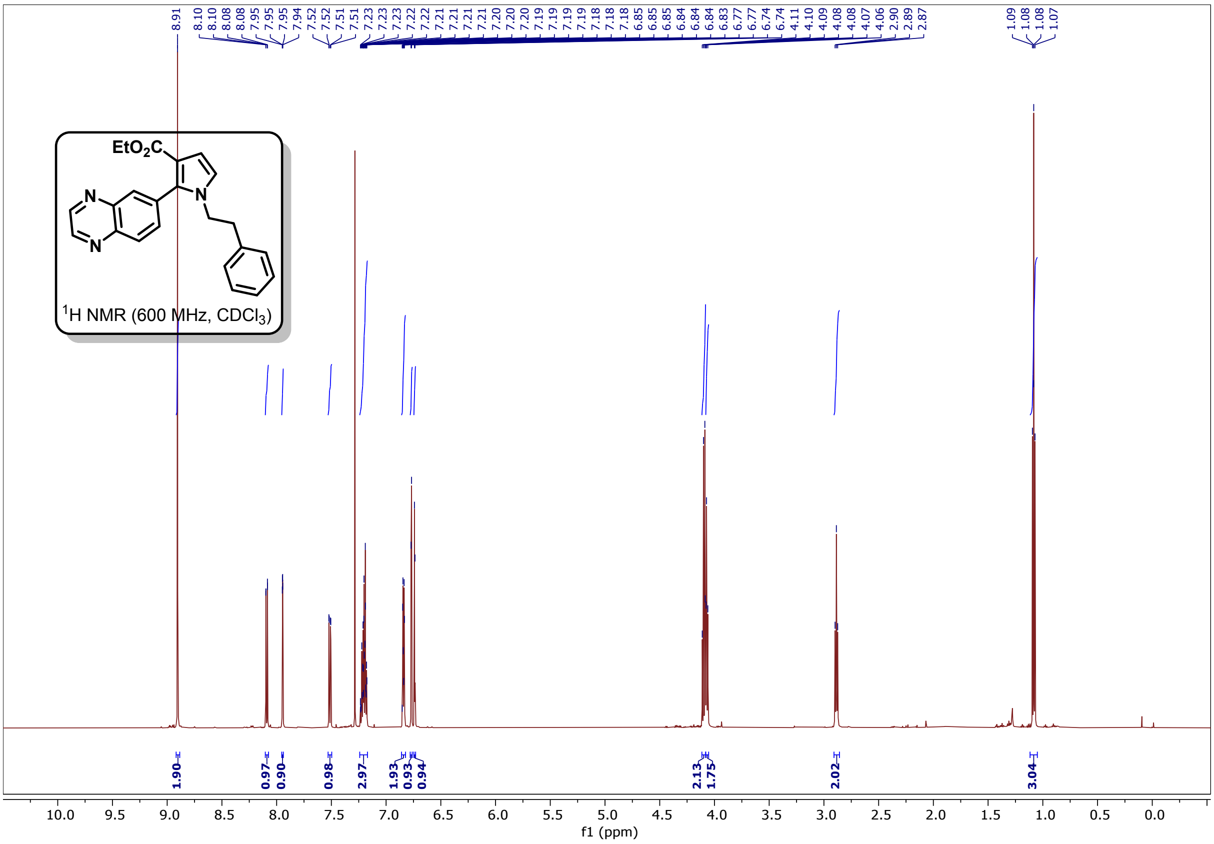

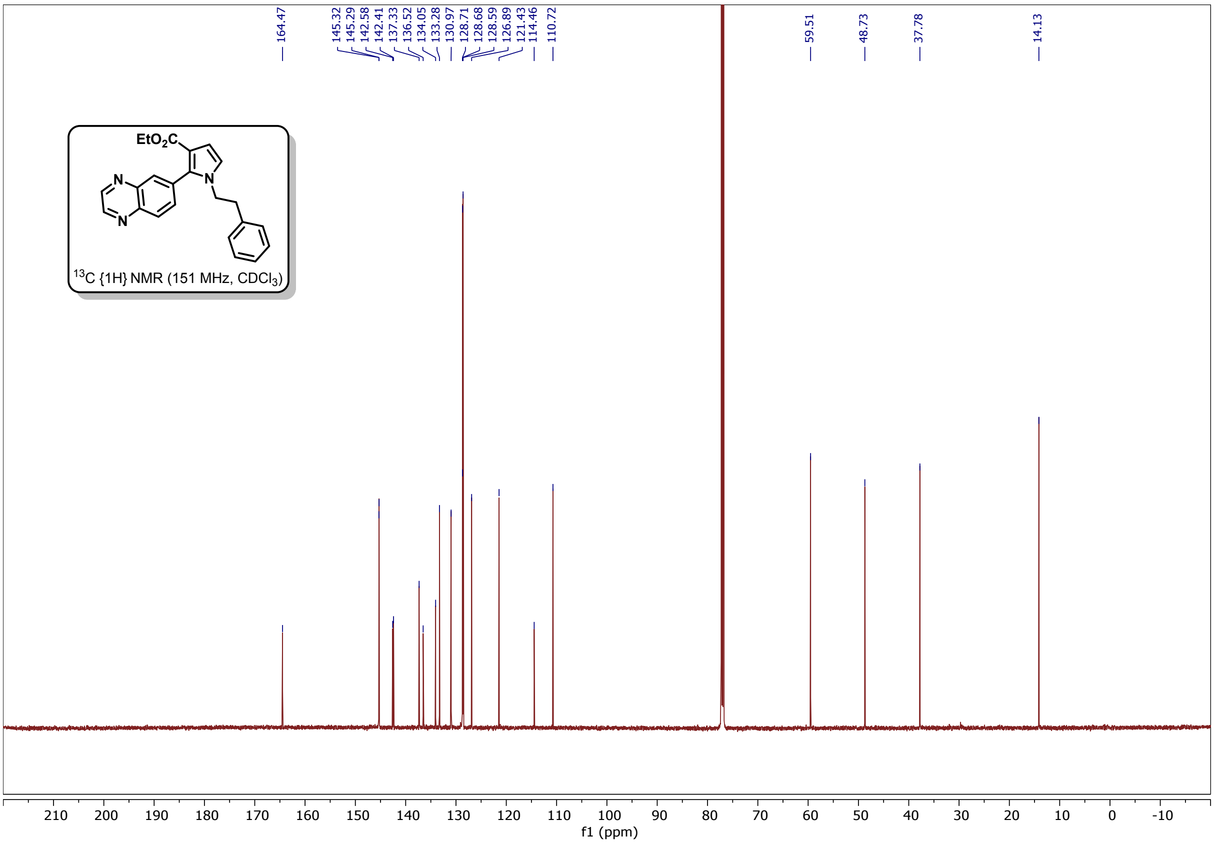

Ethyl 1-phenethyl-2-(thiophen-2-yl)-1H-pyrrole-3-carboxylate (4ka):

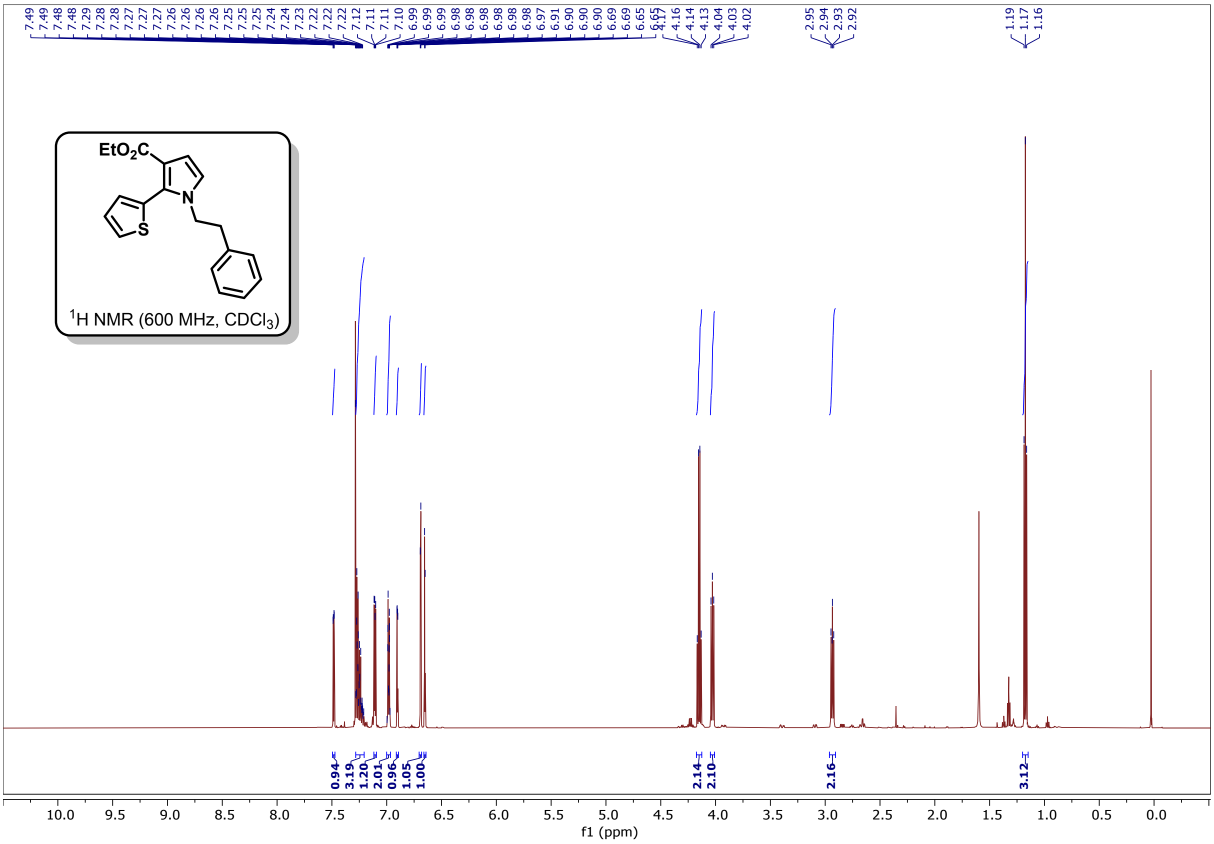

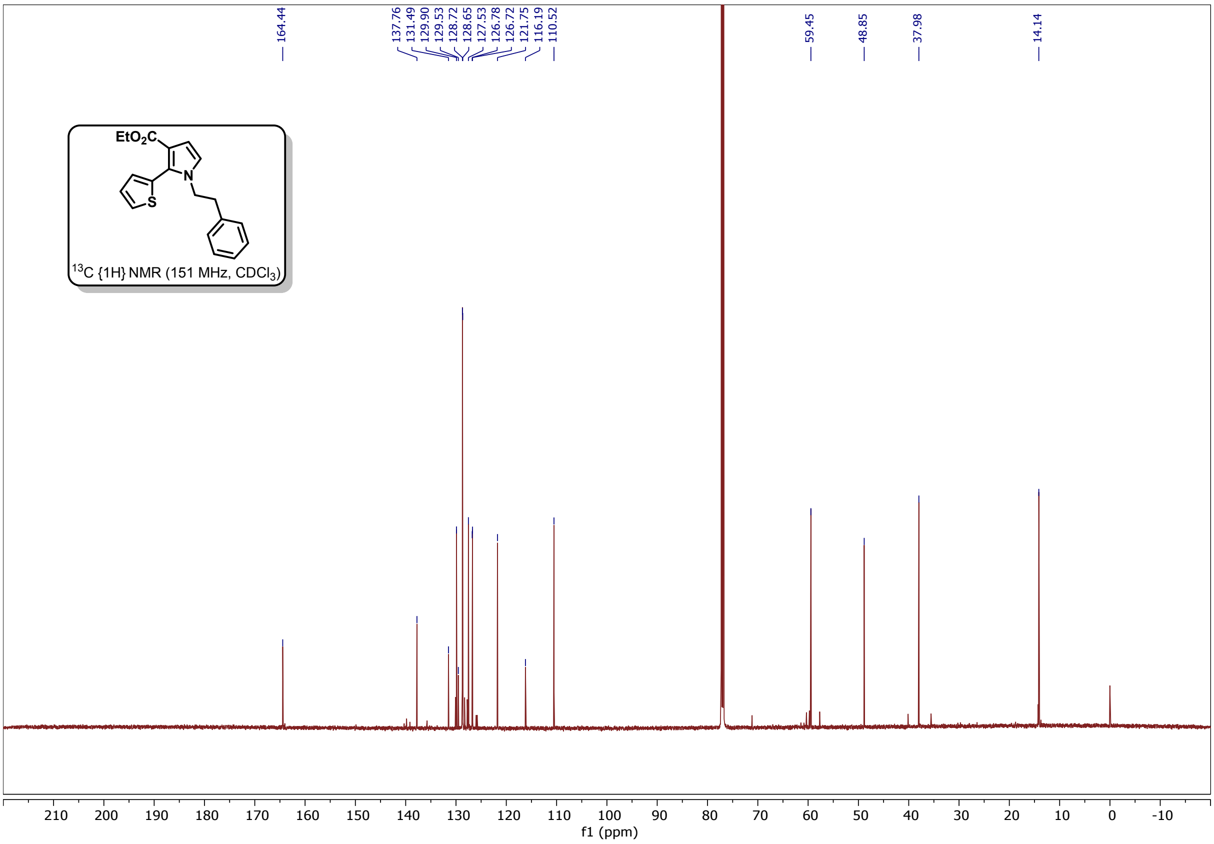

Ethyl 2-(5-chlorofuran-2-yl)-1-phenethyl-1H-pyrrole-3-carboxylate (4la):

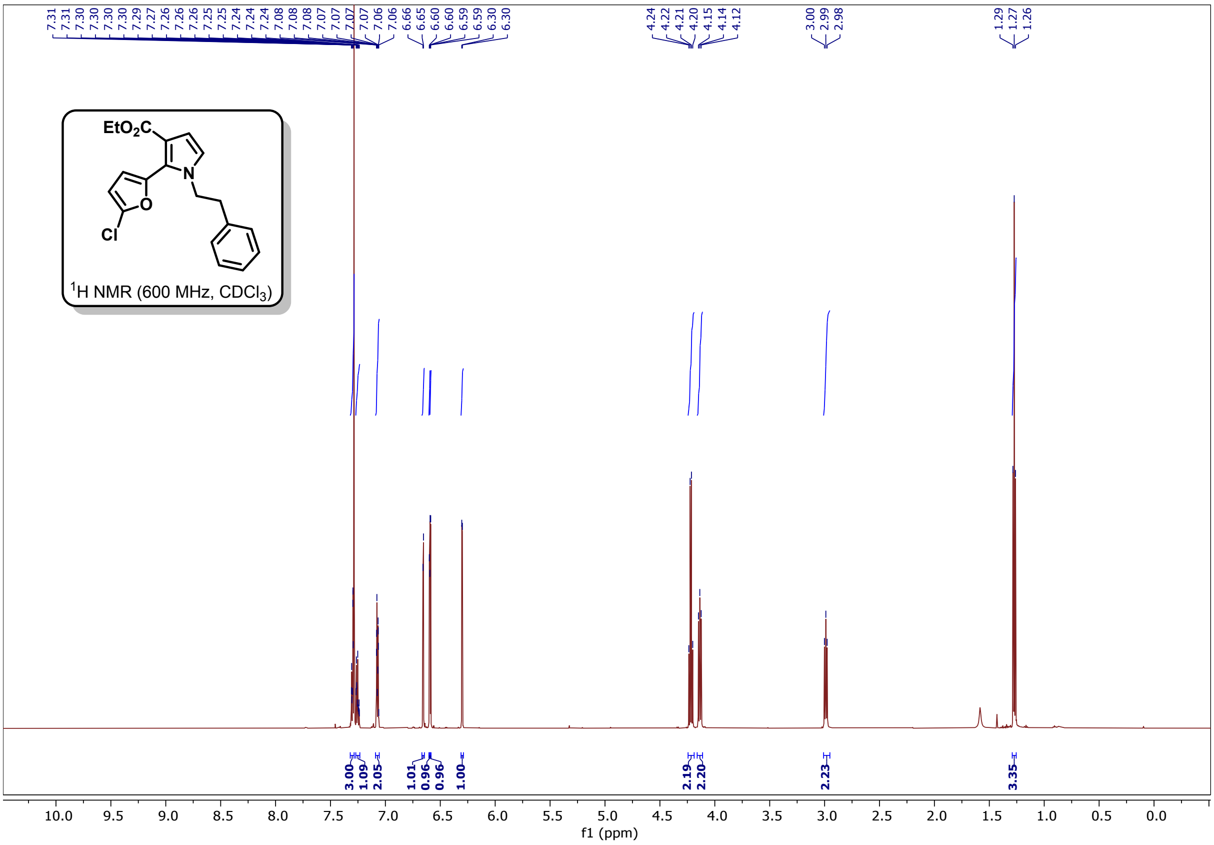

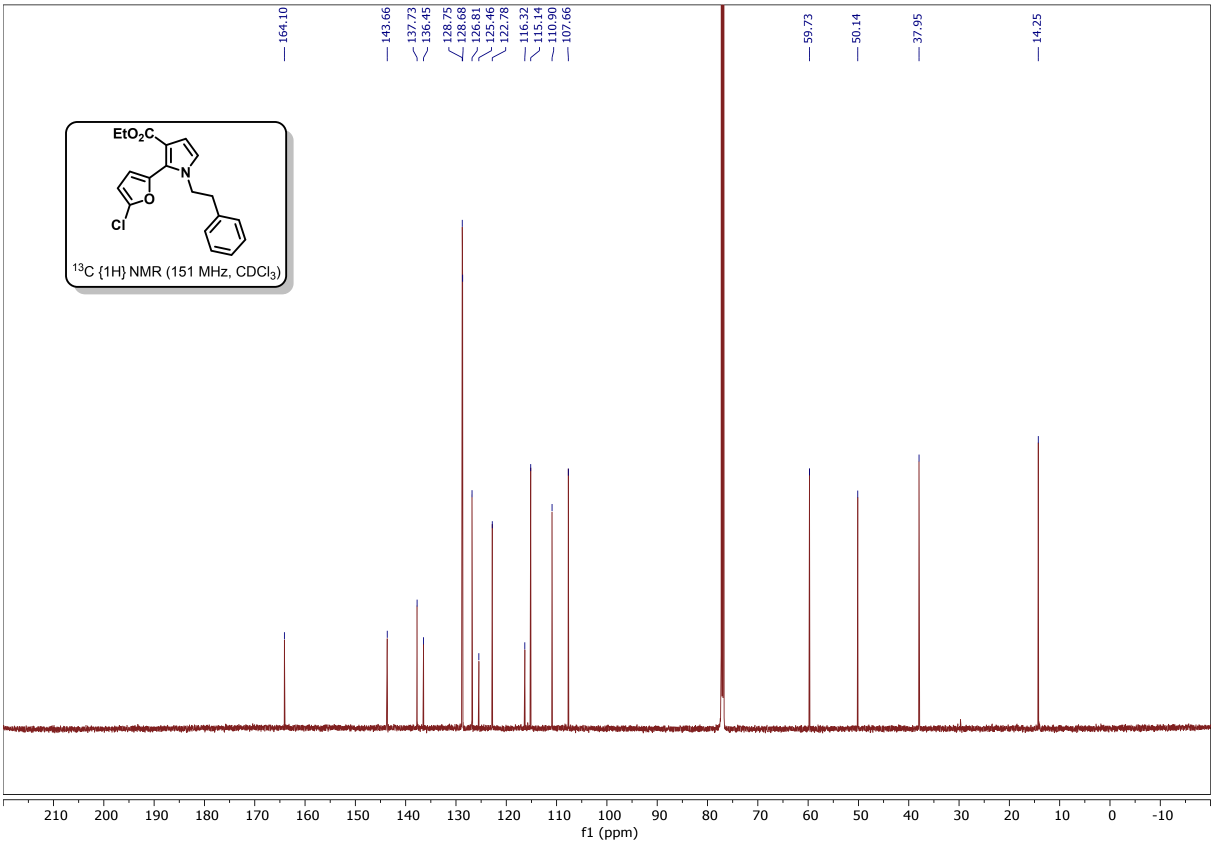

Ethyl 2-(oxazol-5-yl)-1-phenethyl-1H-pyrrole-3-carboxylate (4ma):

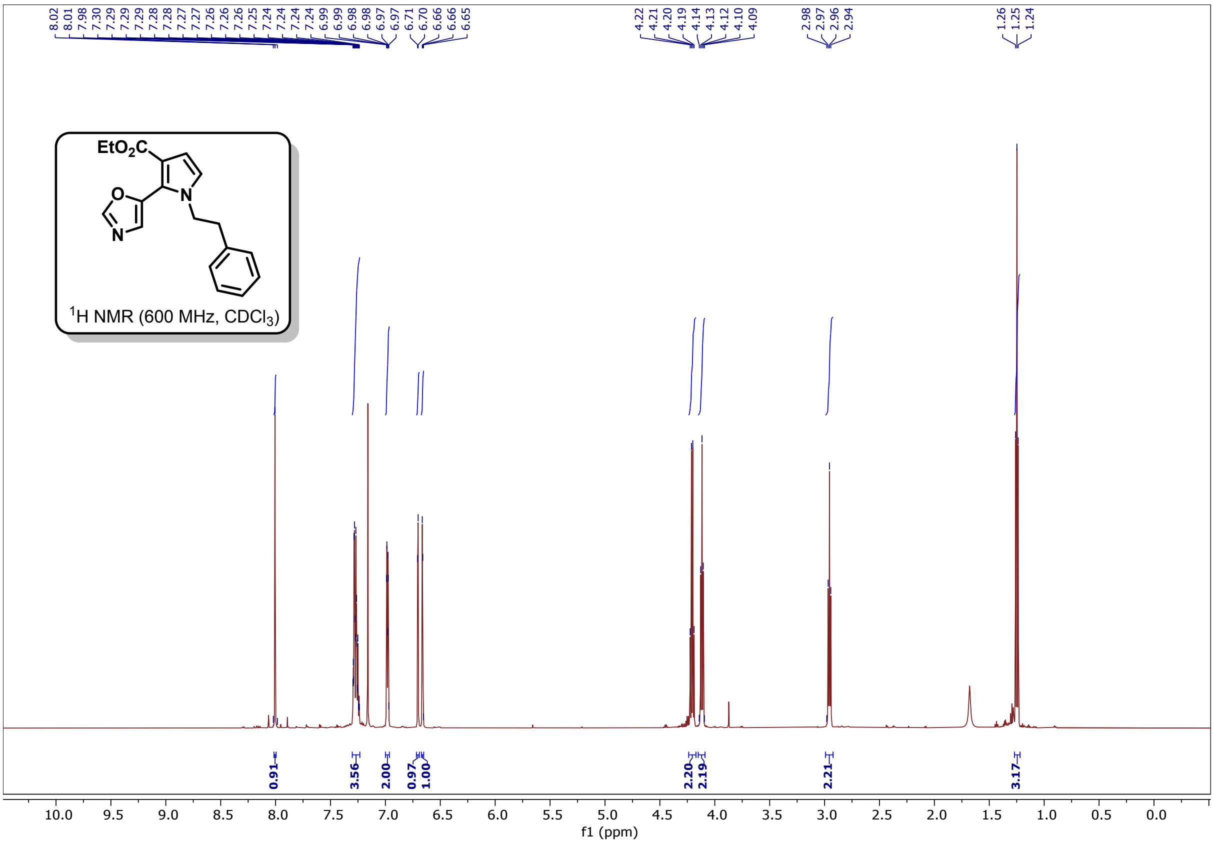

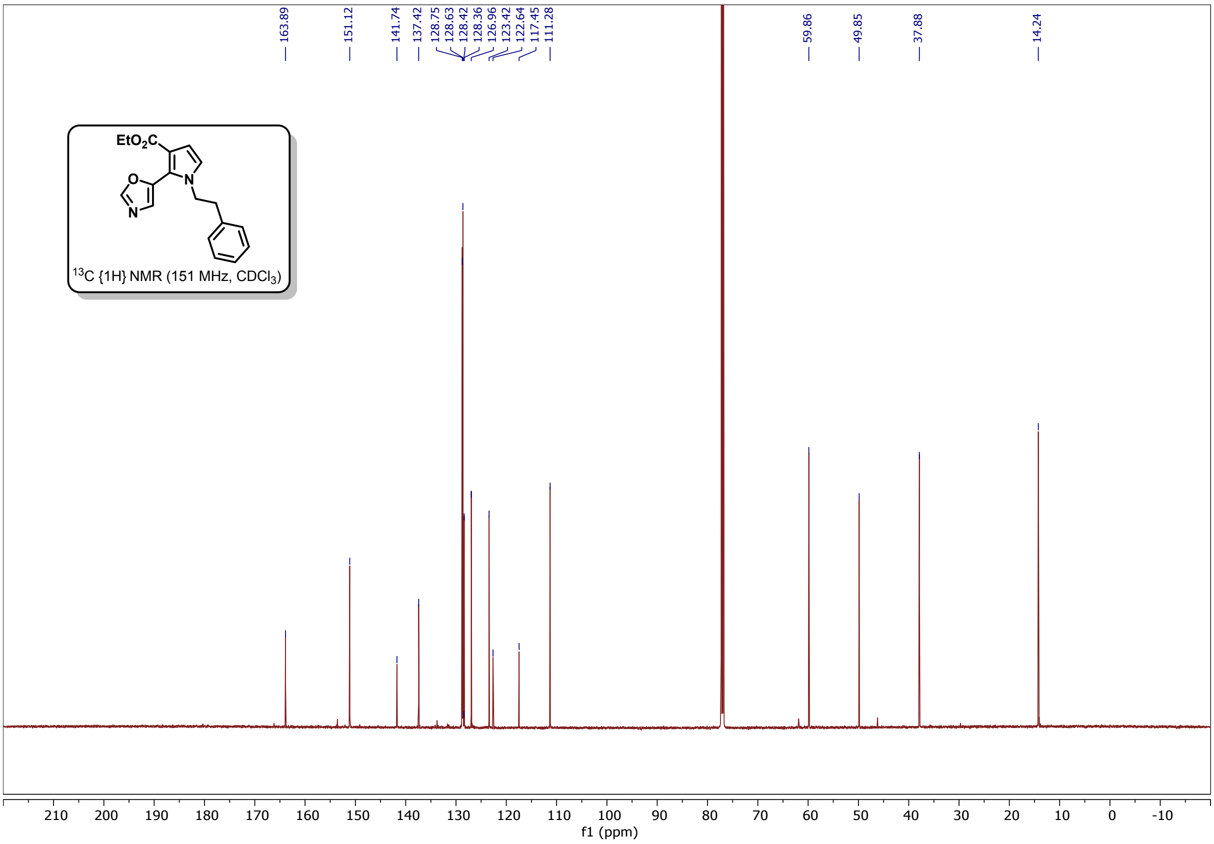

Ethyl 1-phenethyl-2-(thiazol-4-yl)-1H-pyrrole-3-carboxylate (4na):

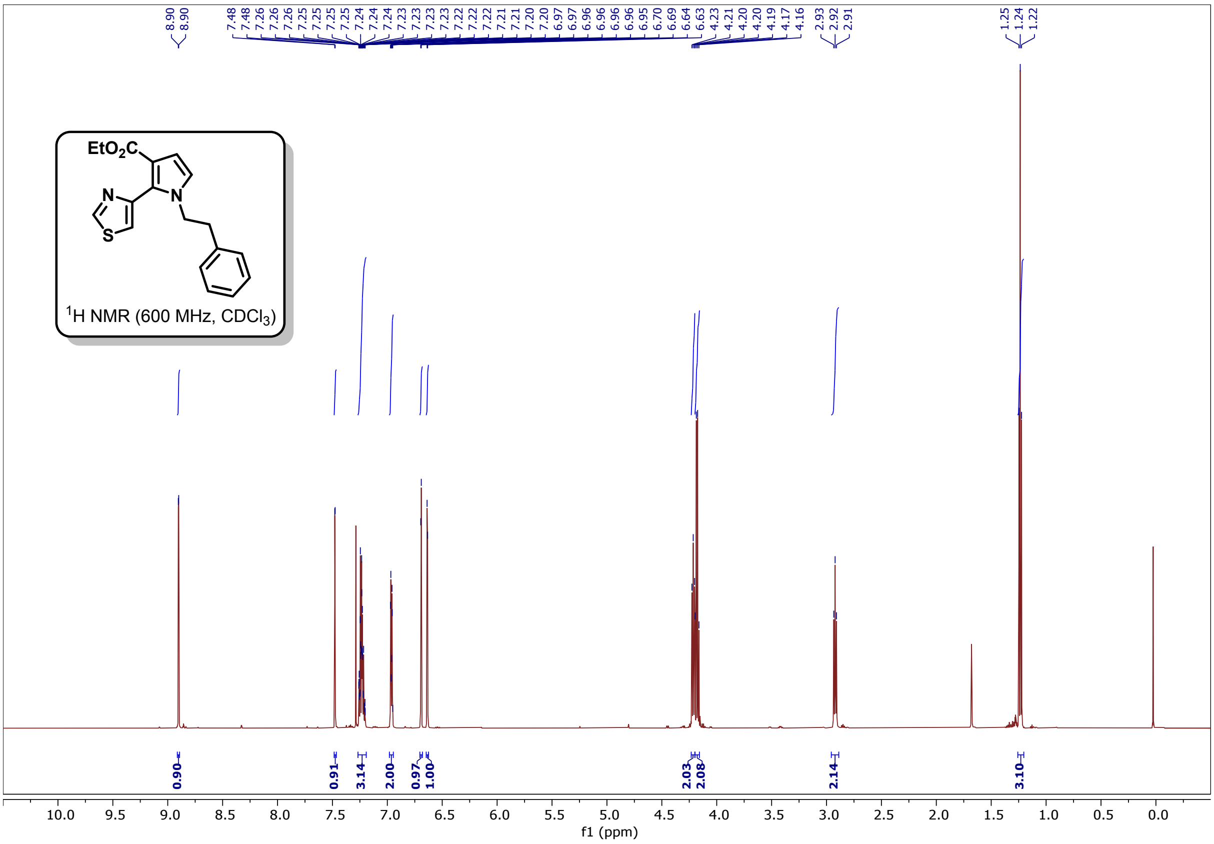

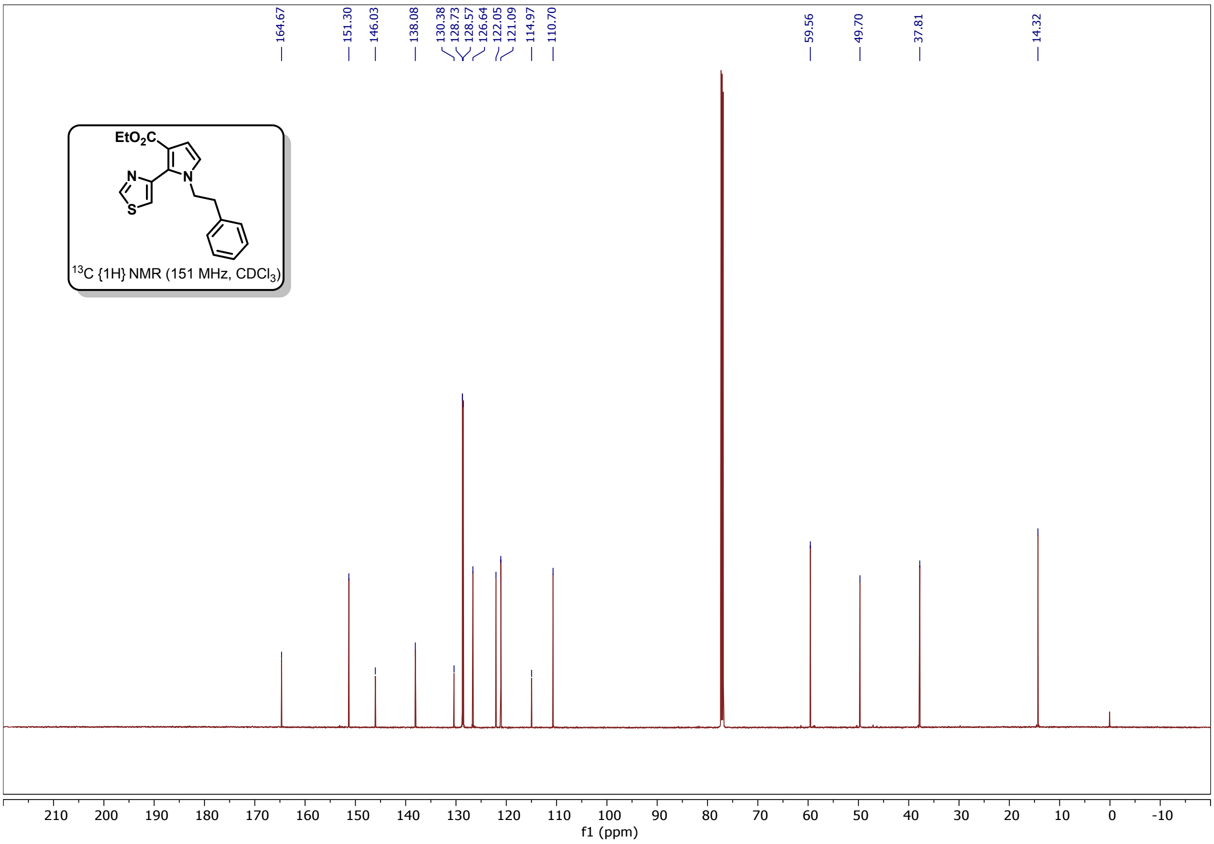

Ethyl 1-phenethyl-2-(1-(pyrimidin-2-yl)piperidin-4-yl)-1H-pyrrole-3-carboxylate (4oa):

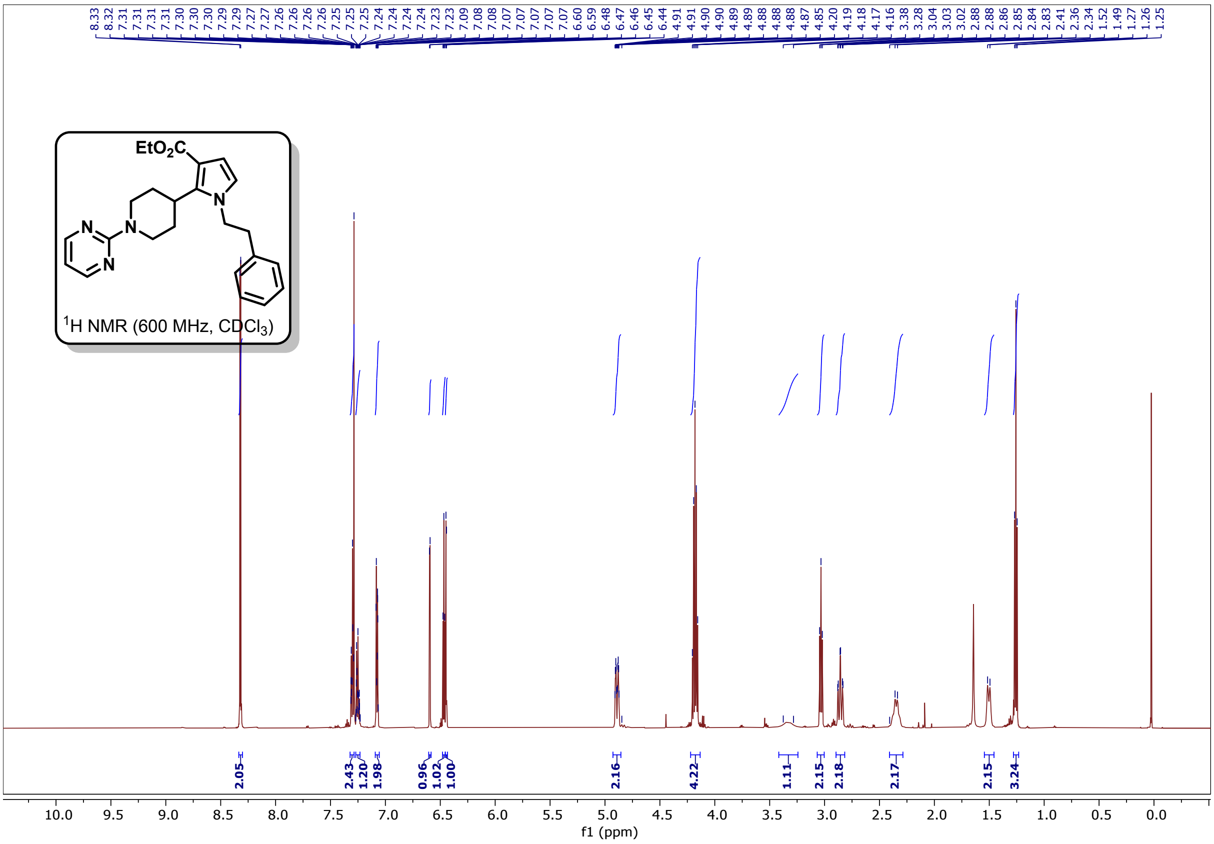

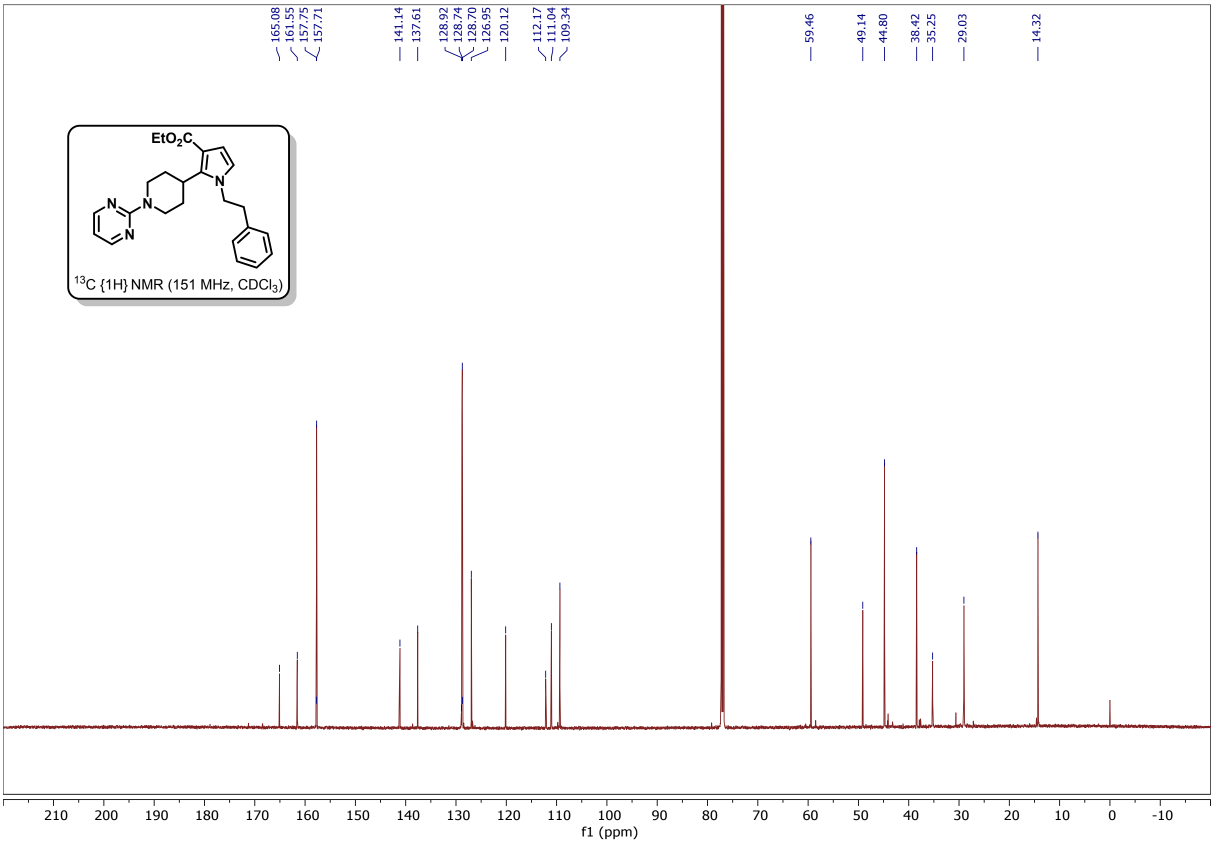

Methyl 2-(4,4-difluorocyclohexyl)-1-phenethyl-1H-pyrrole-3-carboxylate (4pa):

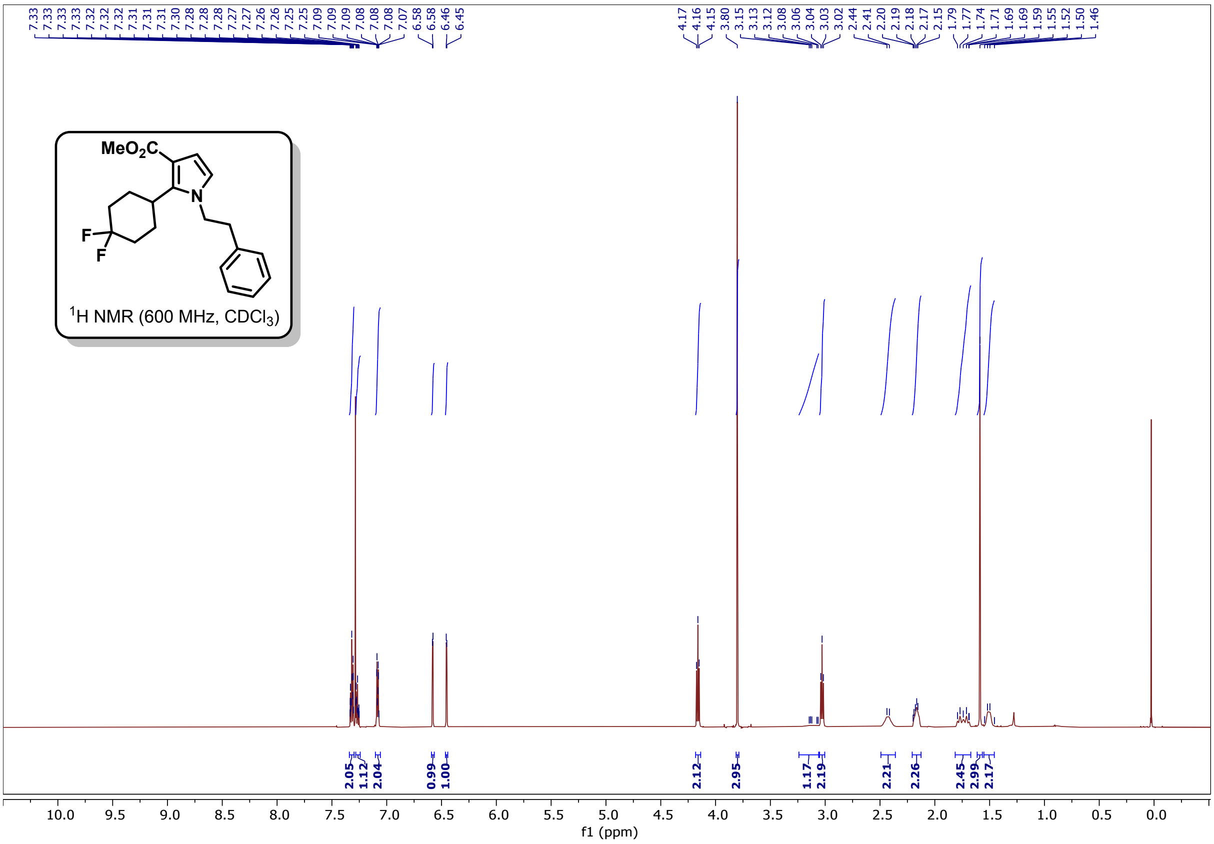

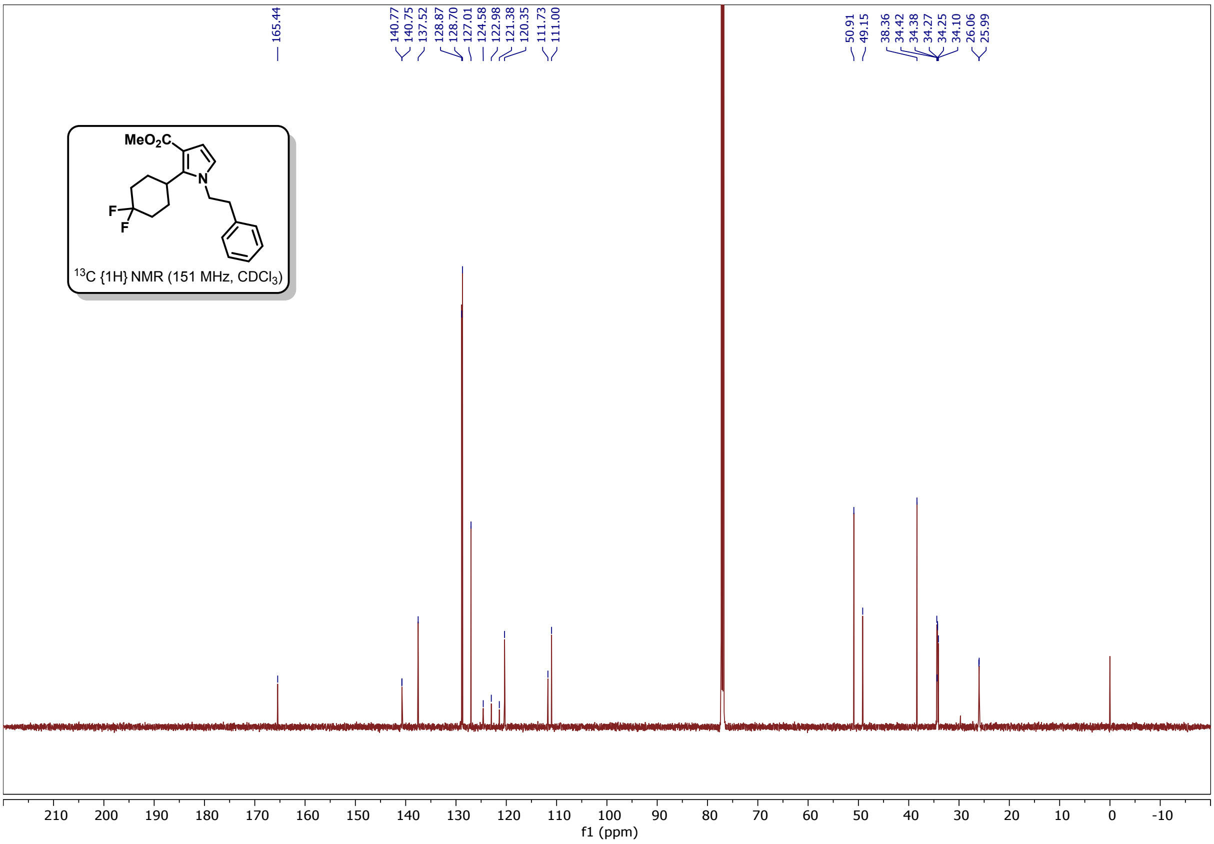

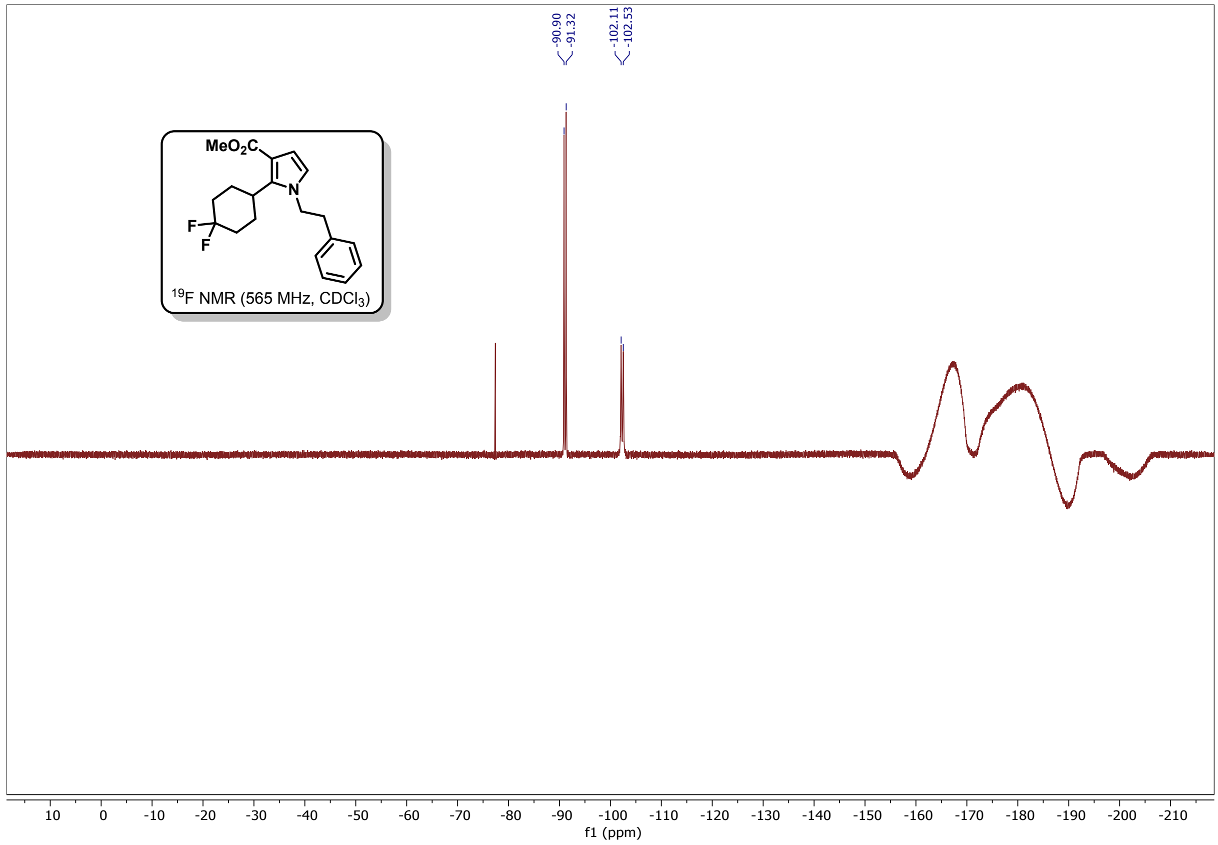

-90.90
-91.32
-102.11
-102.53
MeO2C
N
F
F
19F NMR (565 MHz, CDCl3)
10
0
-10
-20
-30
-40
-50
-60
-70
-80
-90
-100
-110
-120
-130
-140
-150
-160
-170
-180
-190
-200
-210
f1 (ppm)

Ethyl 2-(4,4-difluorocyclohexyl)-1-phenethyl-1H-pyrrole-3-carboxylate (4qa):

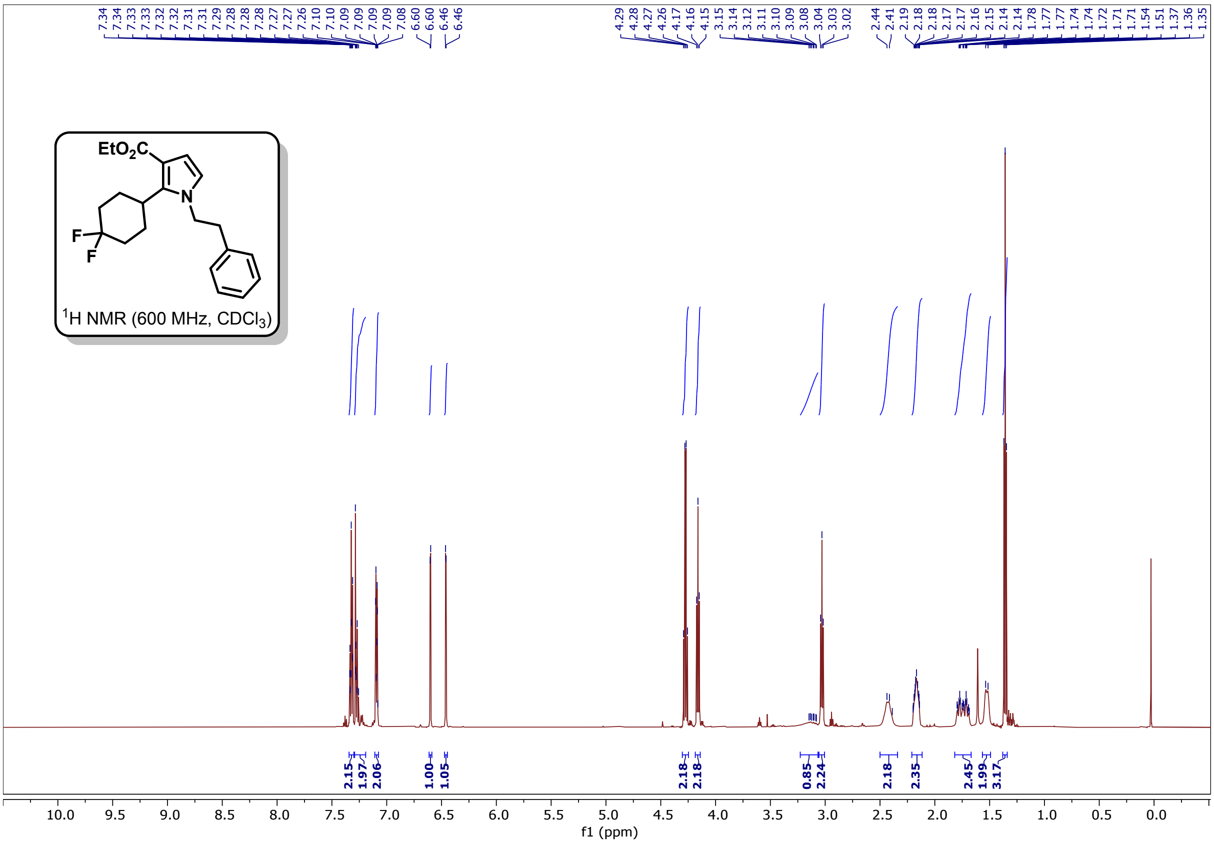

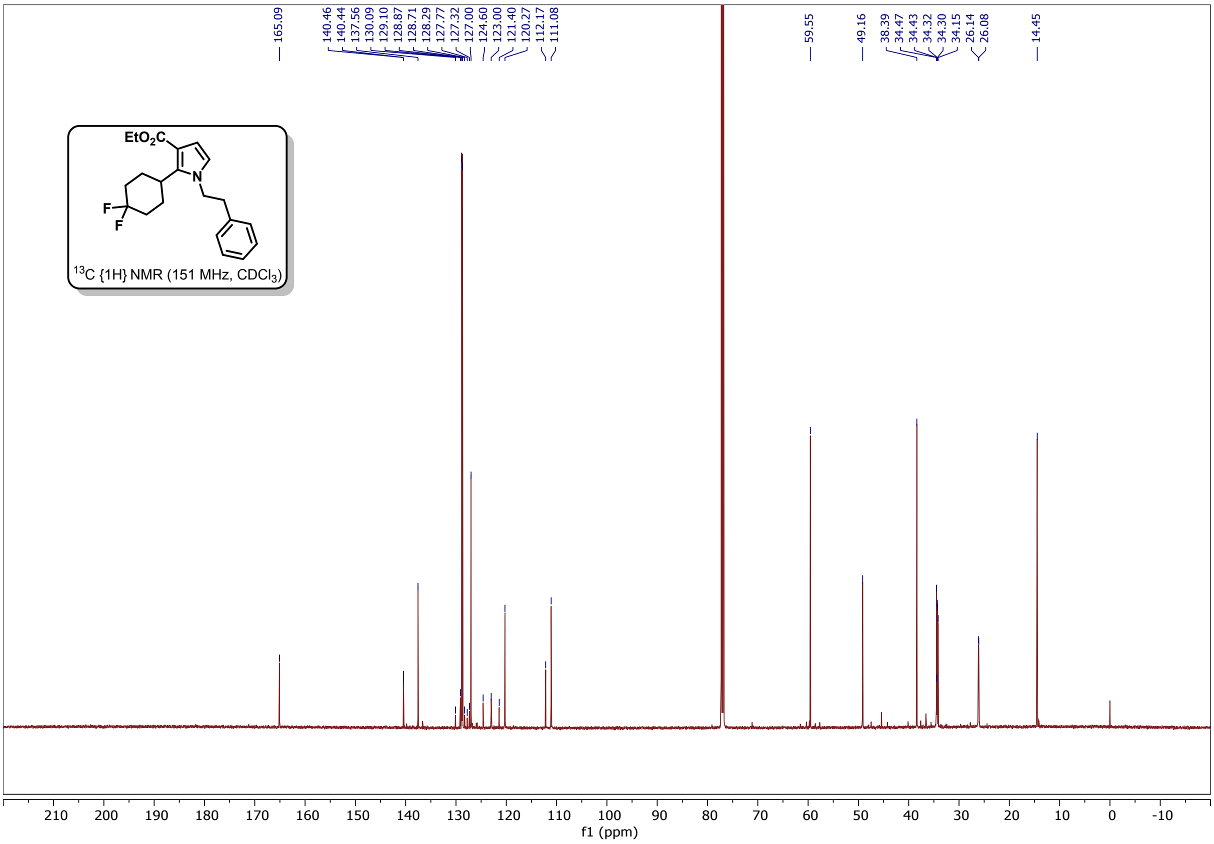

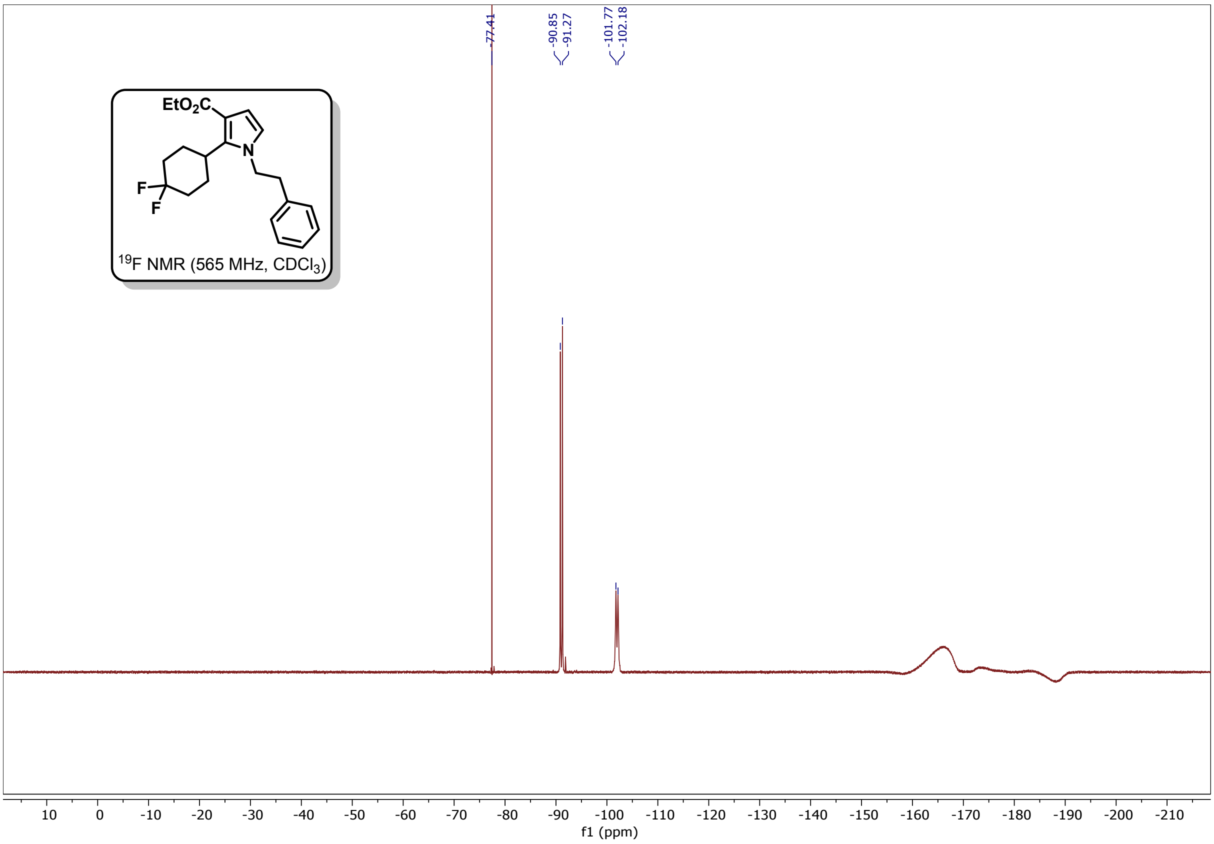

-77.41
-90.85
-91.27
-101.77
-102.18
EtO2C
N
F
F
19F NMR (565 MHz, CDCl3)
10
0
-10
-20
-30
-40
-50
-60
-70
-80
-90
-100
-110
-120
-130
-140
-150
-160
-170
-180
-190
-200
-210
f1 (ppm)

tert-butyl 4-(3-(ethoxycarbonyl)-1-phenethyl-1H-pyrrol-2-yl)piperidine-1-carboxylate (4ra):

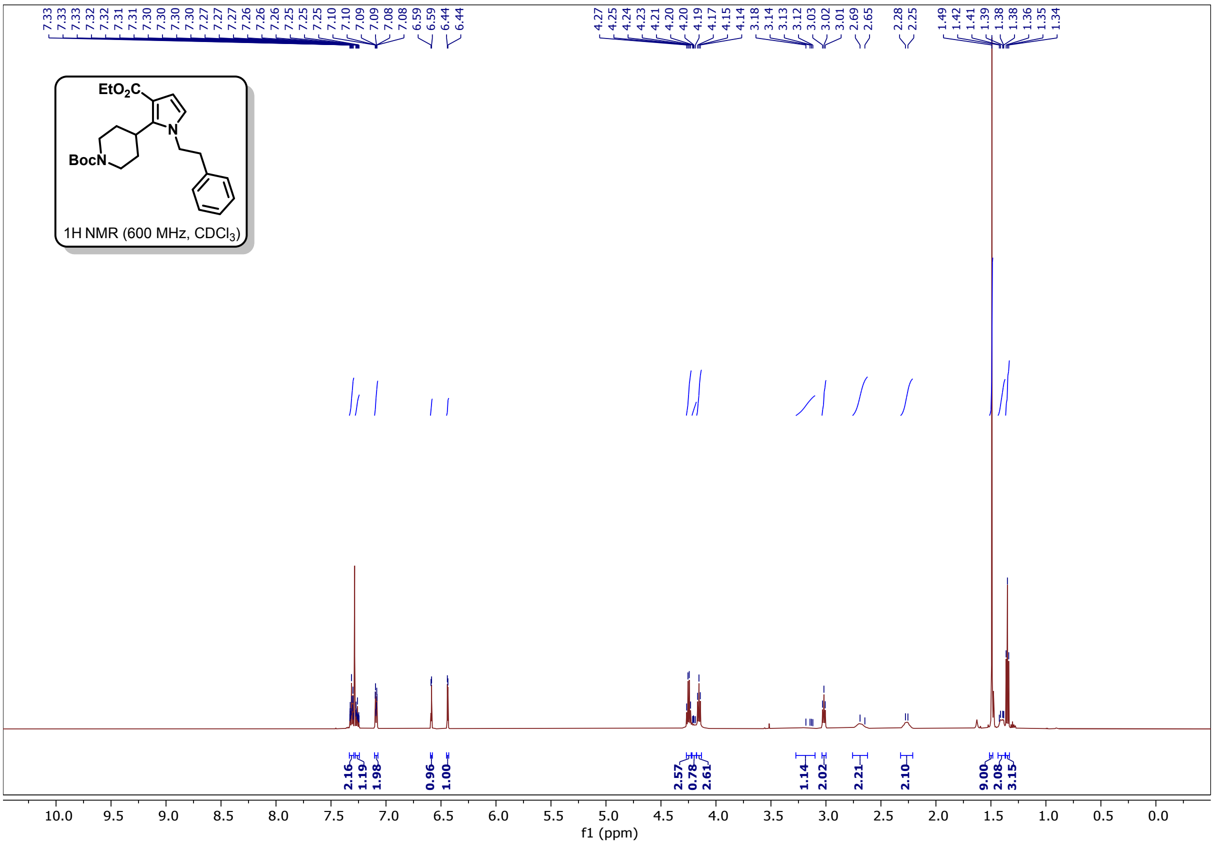

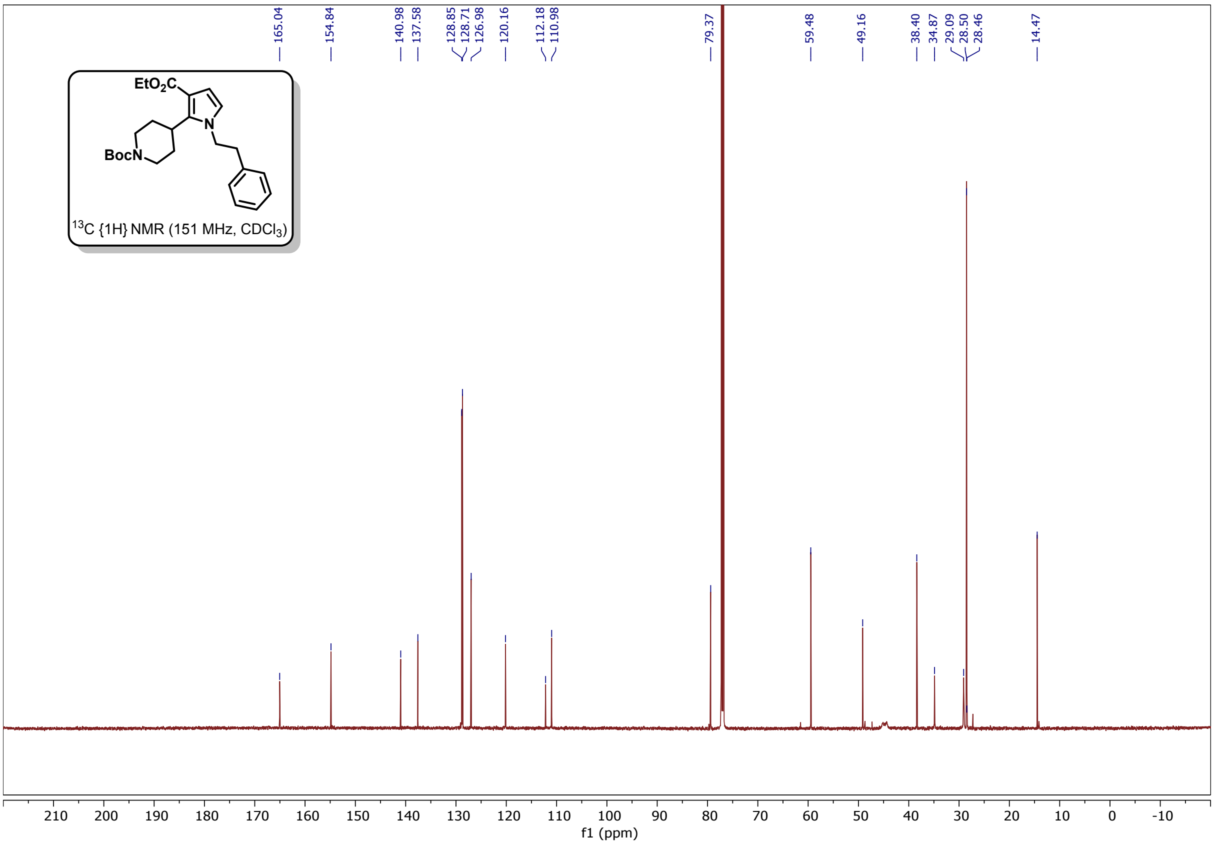

Ethyl (R)-2-(1-(tert-butoxycarbonyl)pyrrolidin-3-yl)-1-phenethyl-1H-pyrrole-3-carboxylate (4sa):

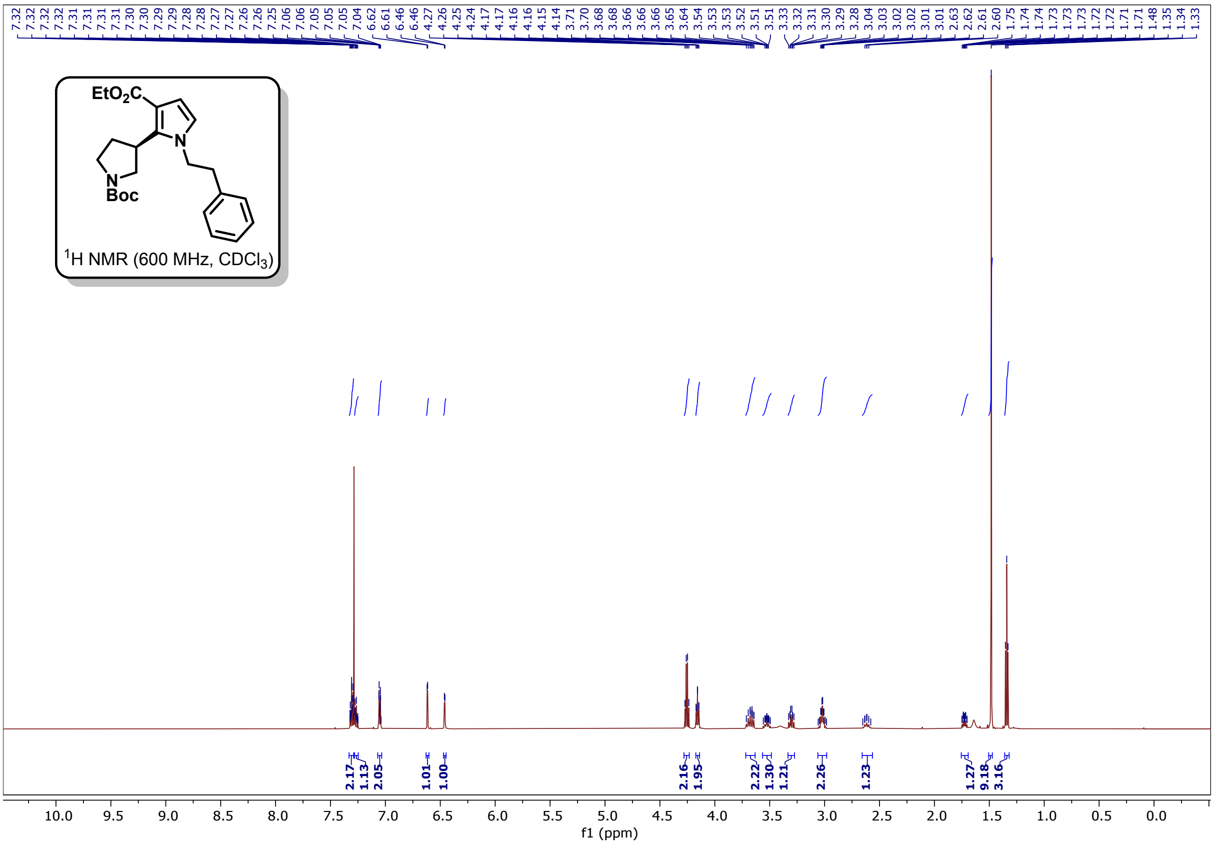

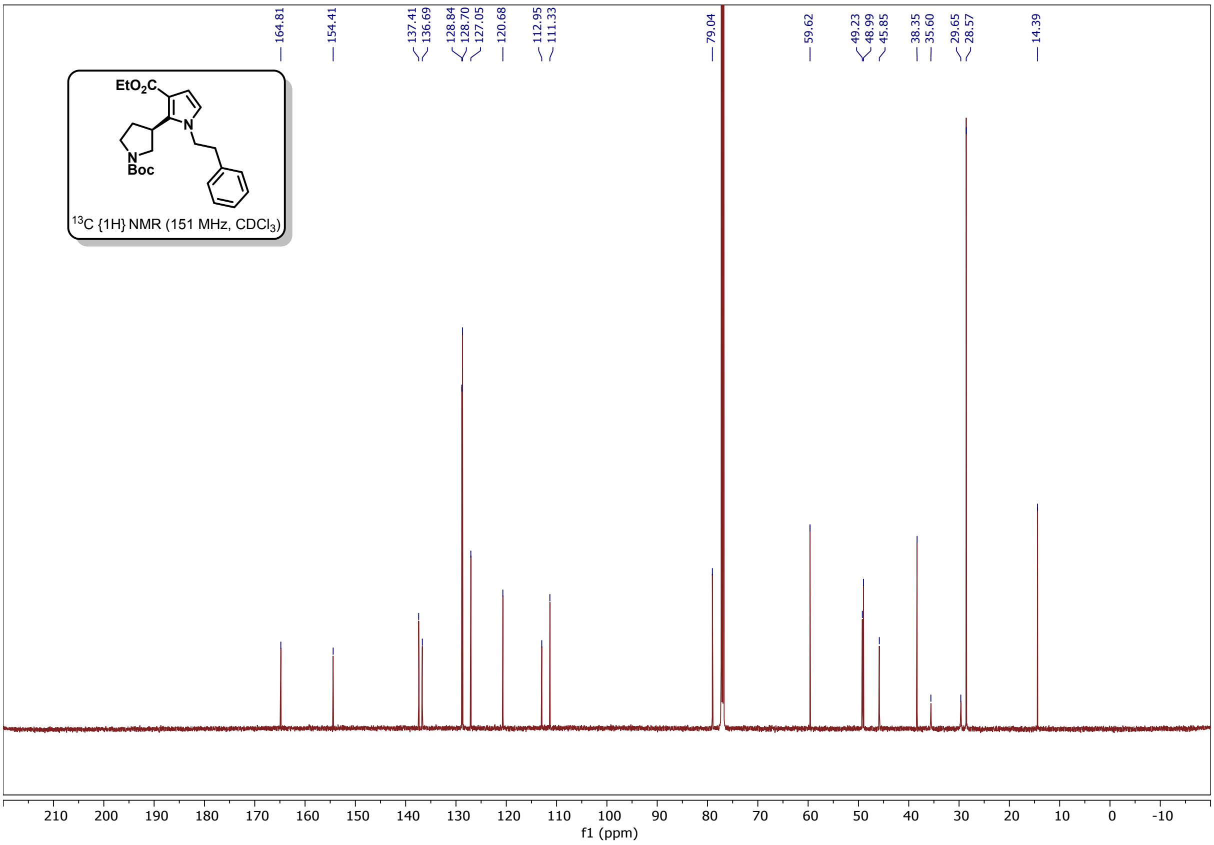

Ethyl 2-(1-(tert-butoxycarbonyl)azetidin-3-yl)-1-phenethyl-1H-pyrrole-3-carboxylate (4ta):

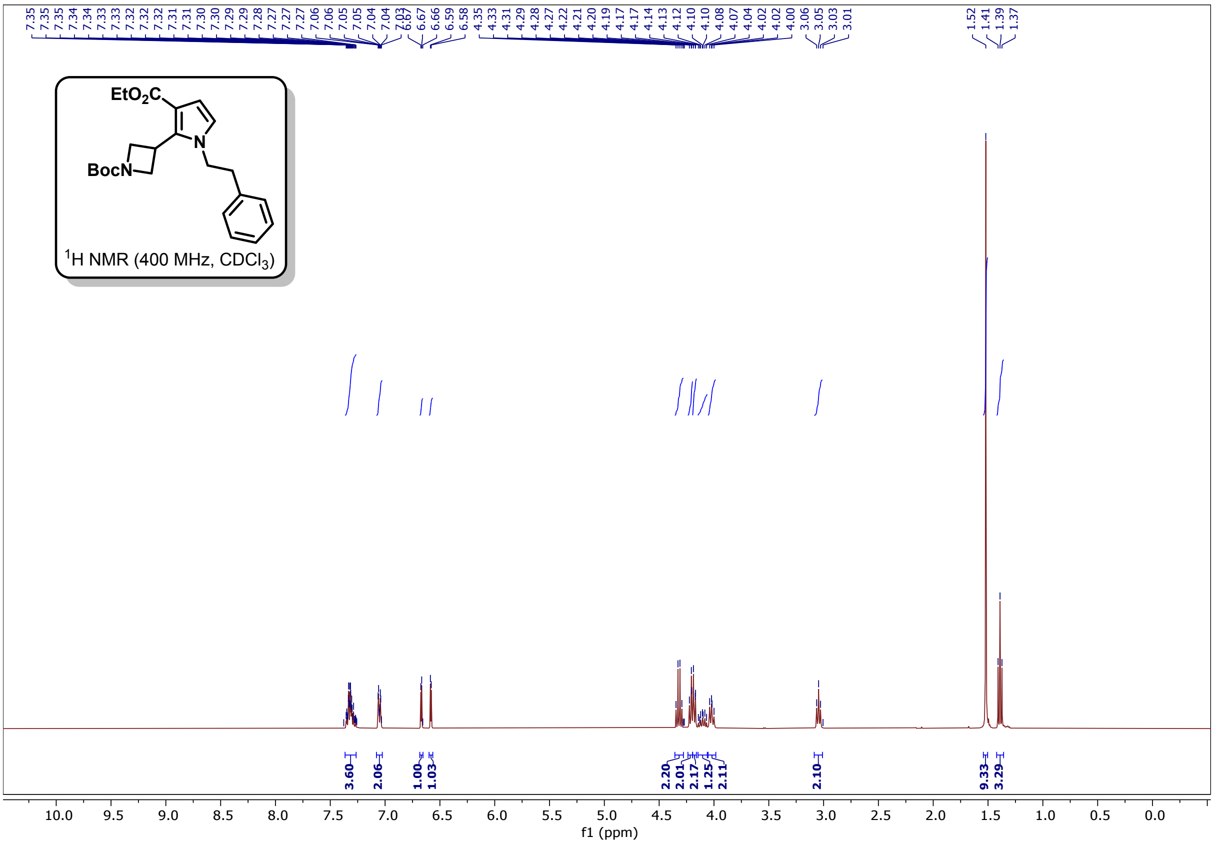

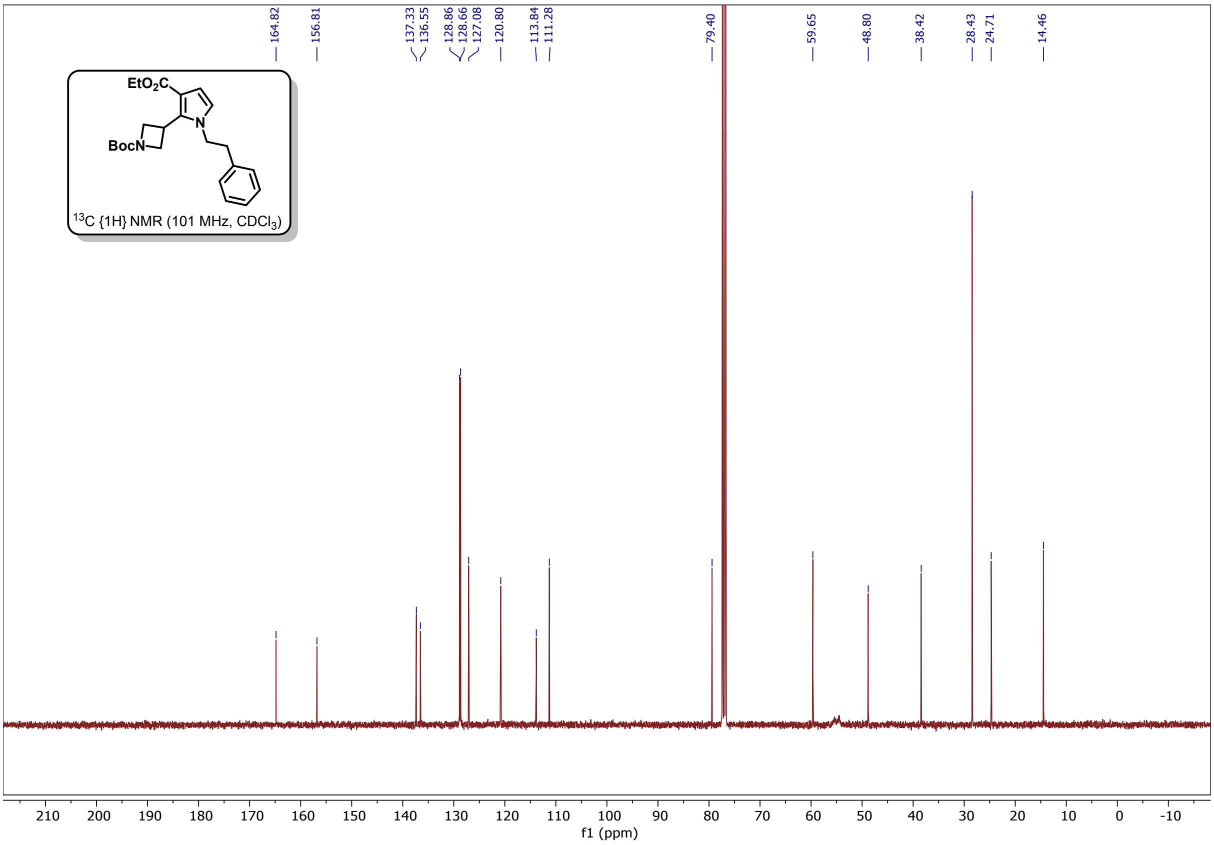

Ethyl 2-(3-methoxy-3-oxopropyl)-1-phenethyl-1H-pyrrole-3-carboxylate (4ua):

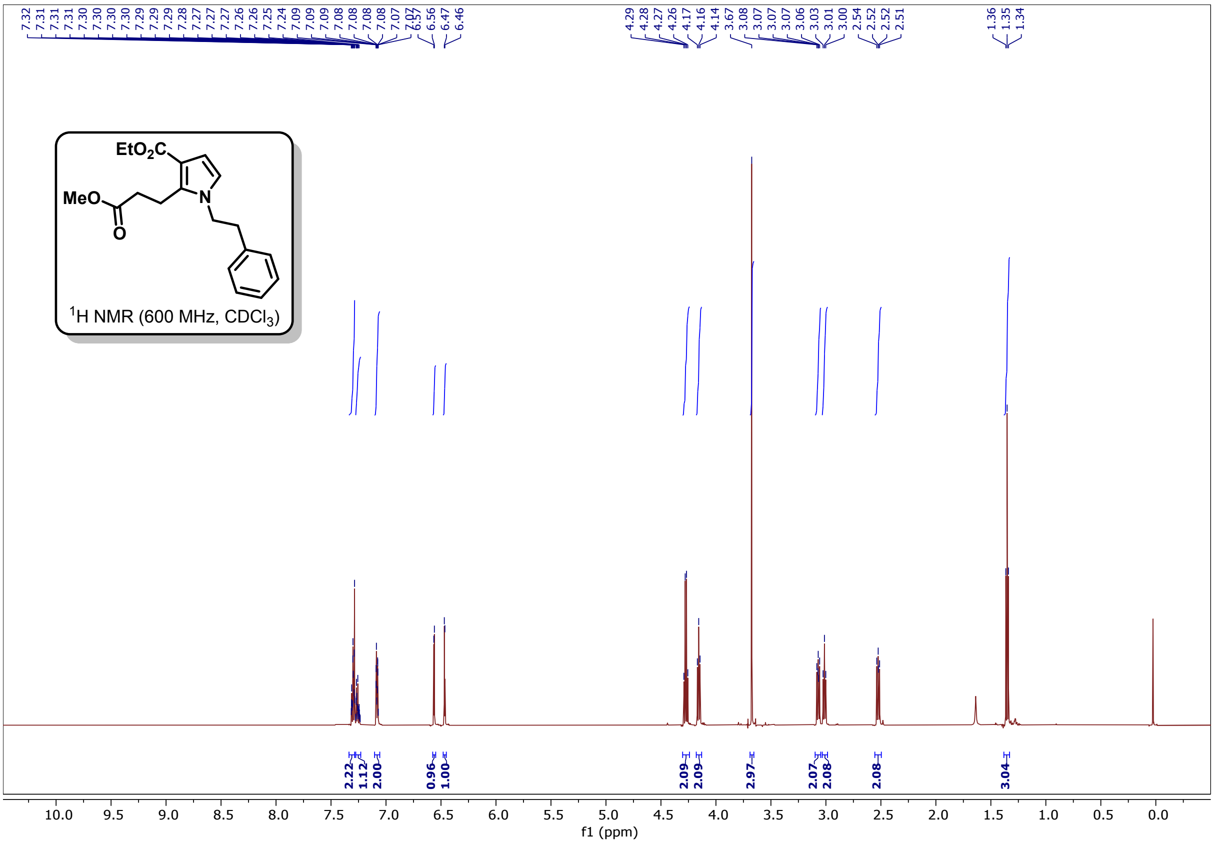

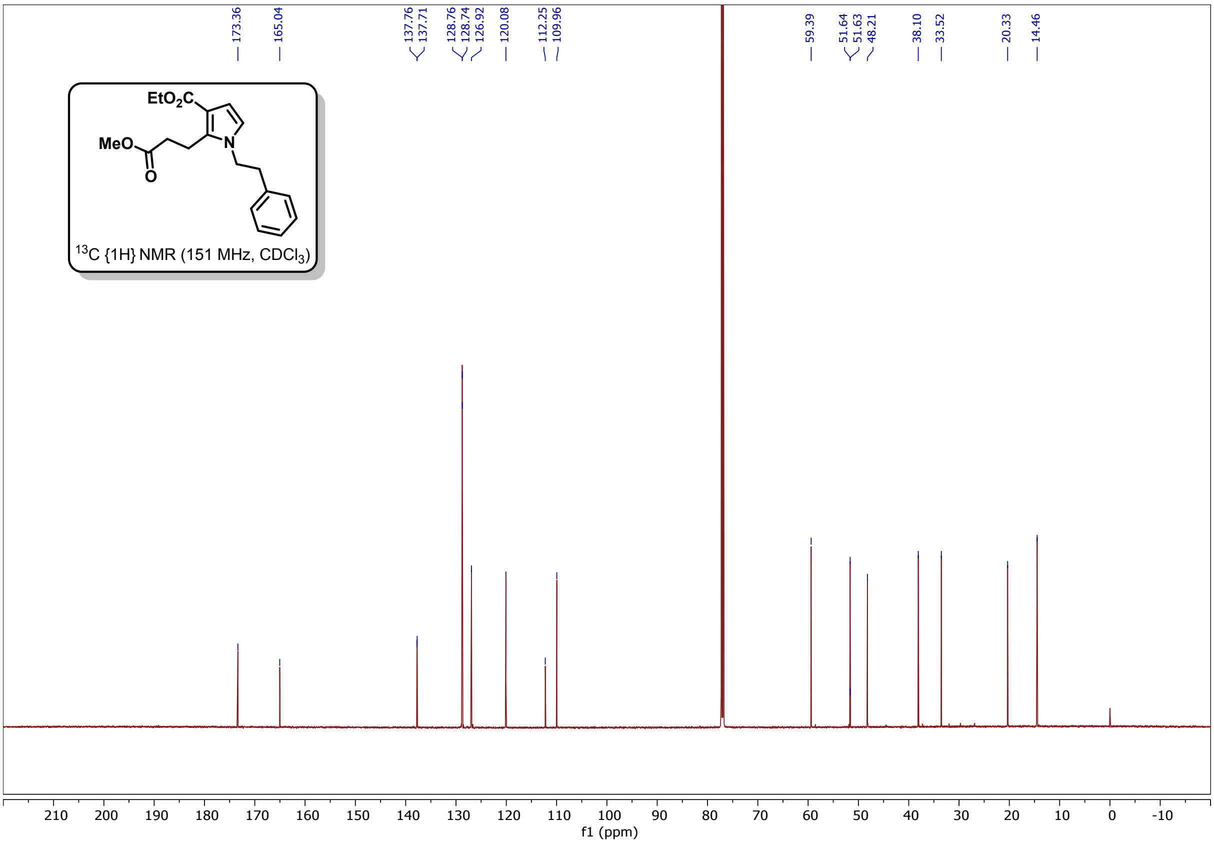

Ethyl 2-(2-fluorophenyl)-1-phenethyl-1H-pyrrole-3-carboxylate (4xa):

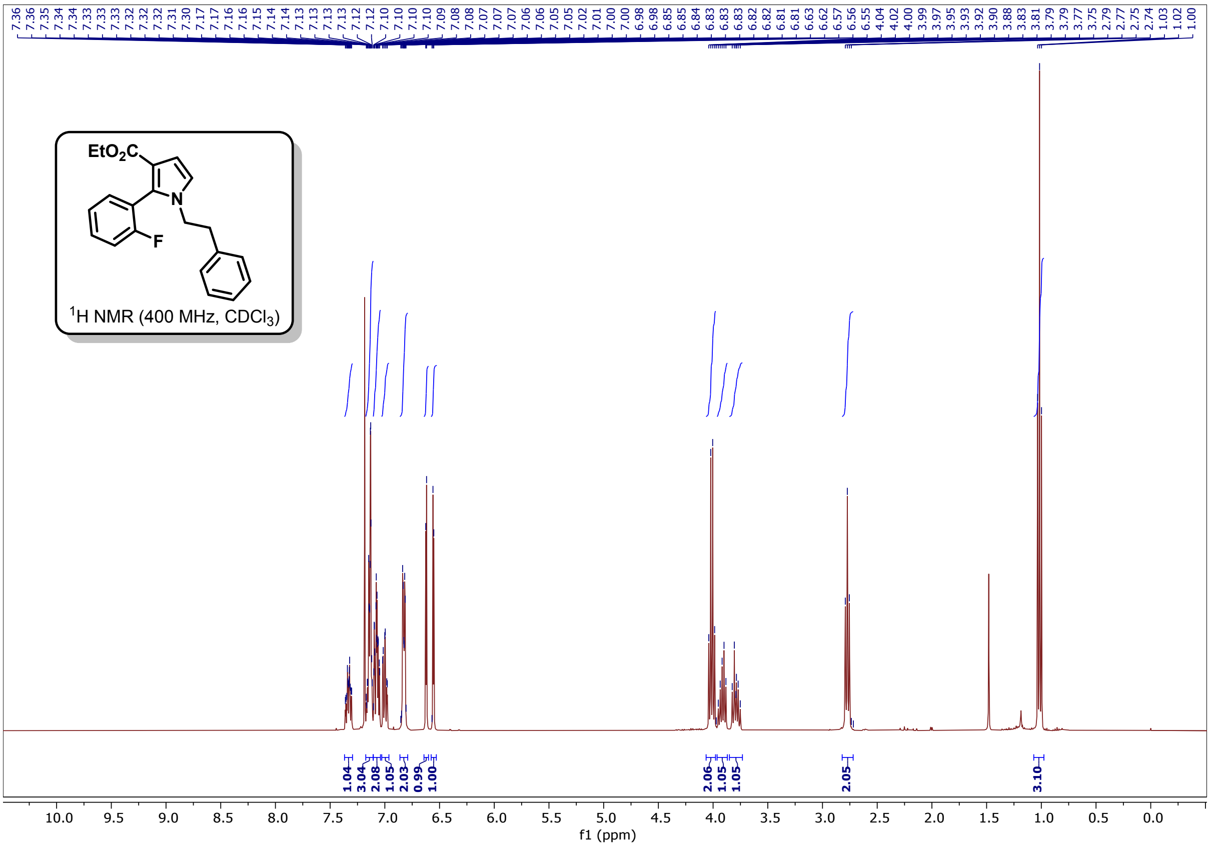

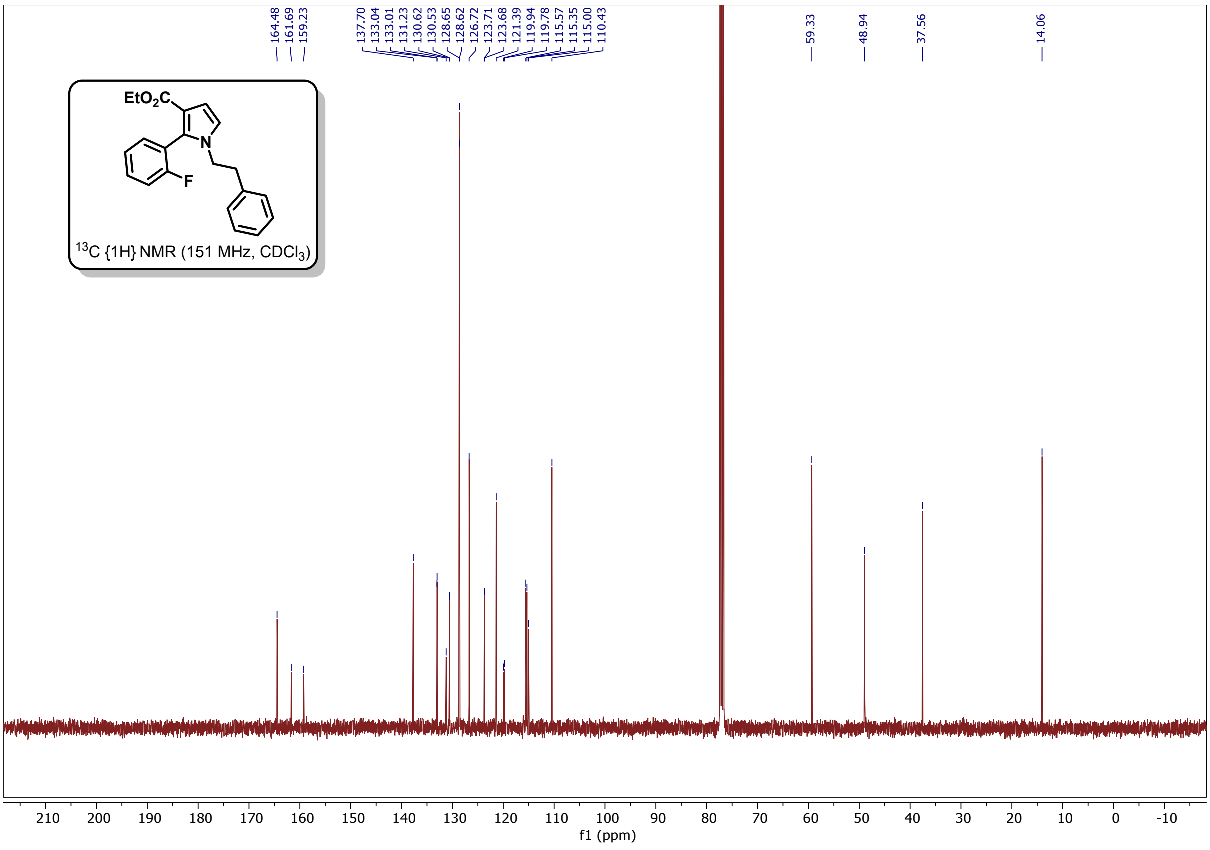

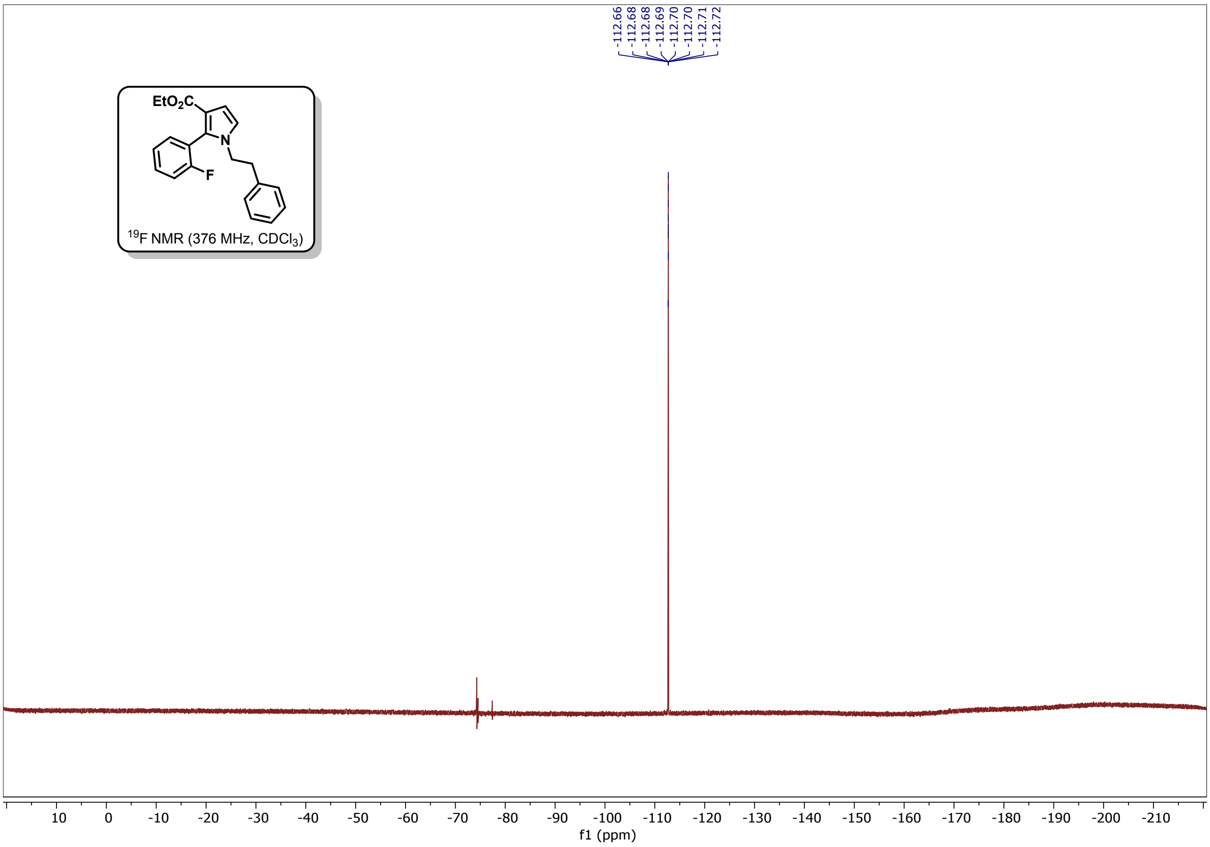
-112.66
-112.68
-112.68
-112.69
-112.70
-112.70
-112.71
-112.72
EtO2C
N
F
19F NMR (376 MHz, CDCl3)
10 0 -10 -20 -30 -40 -50 -60 -70 -80 -90 -100 -110 -120 -130 -140 -150 -160 -170 -180 -190 -200 -210
f1 (ppm)

2-(3-fluorophenyl)-1-phenethyl-1H-pyrrole-3-carboxylic acid (5aa):

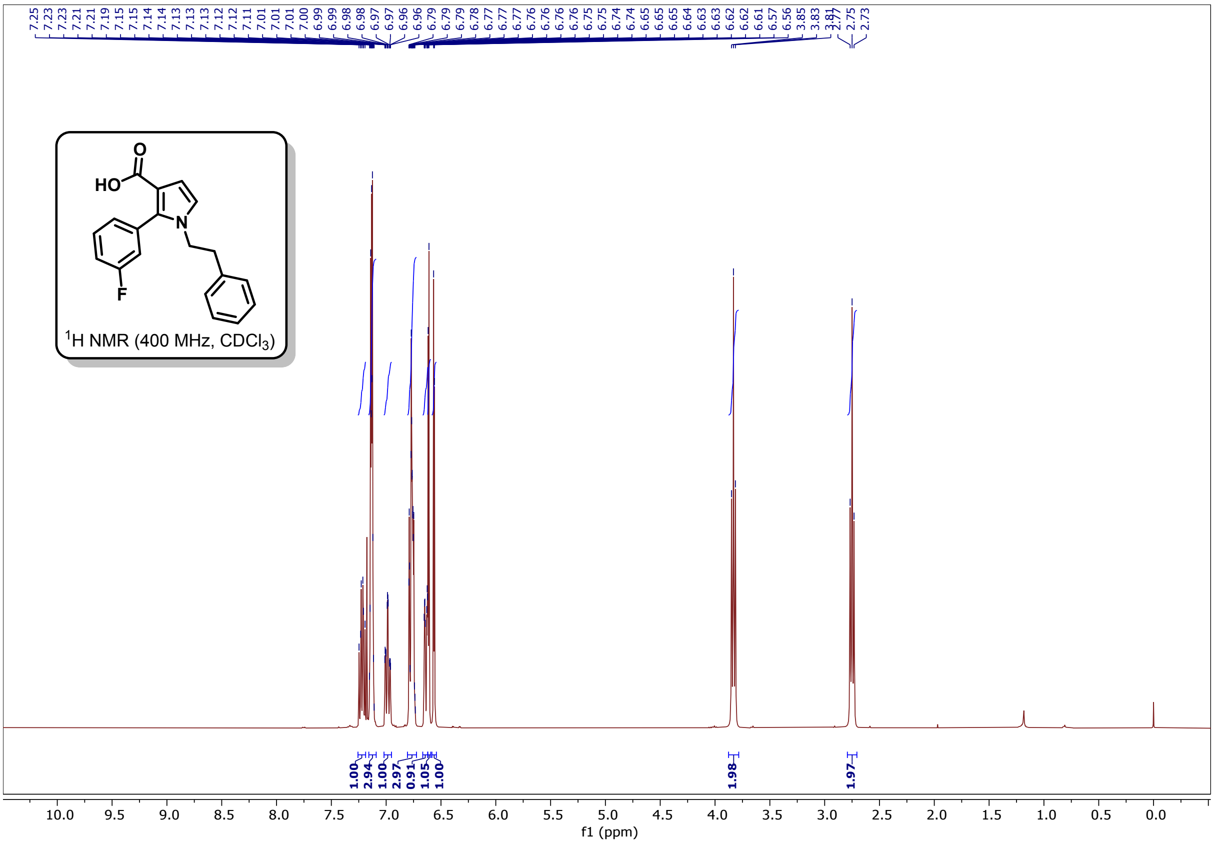

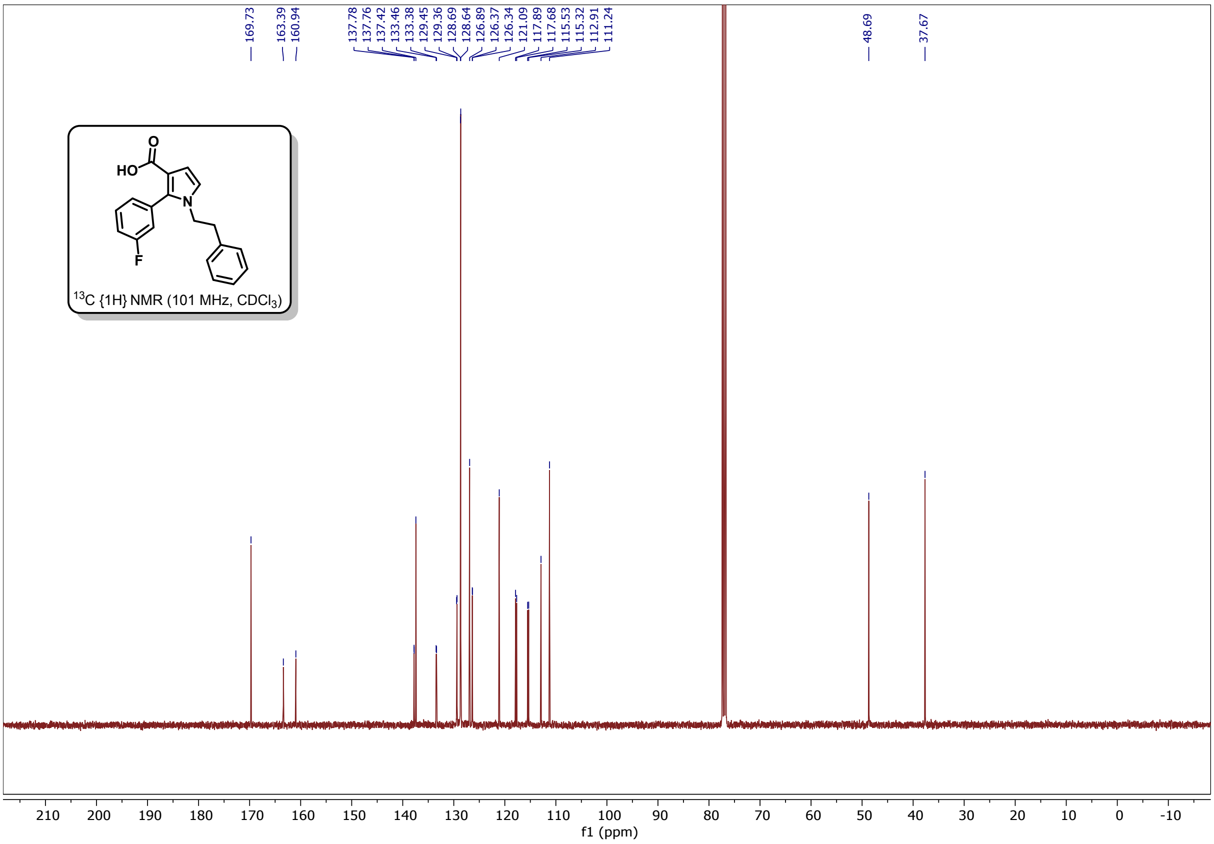

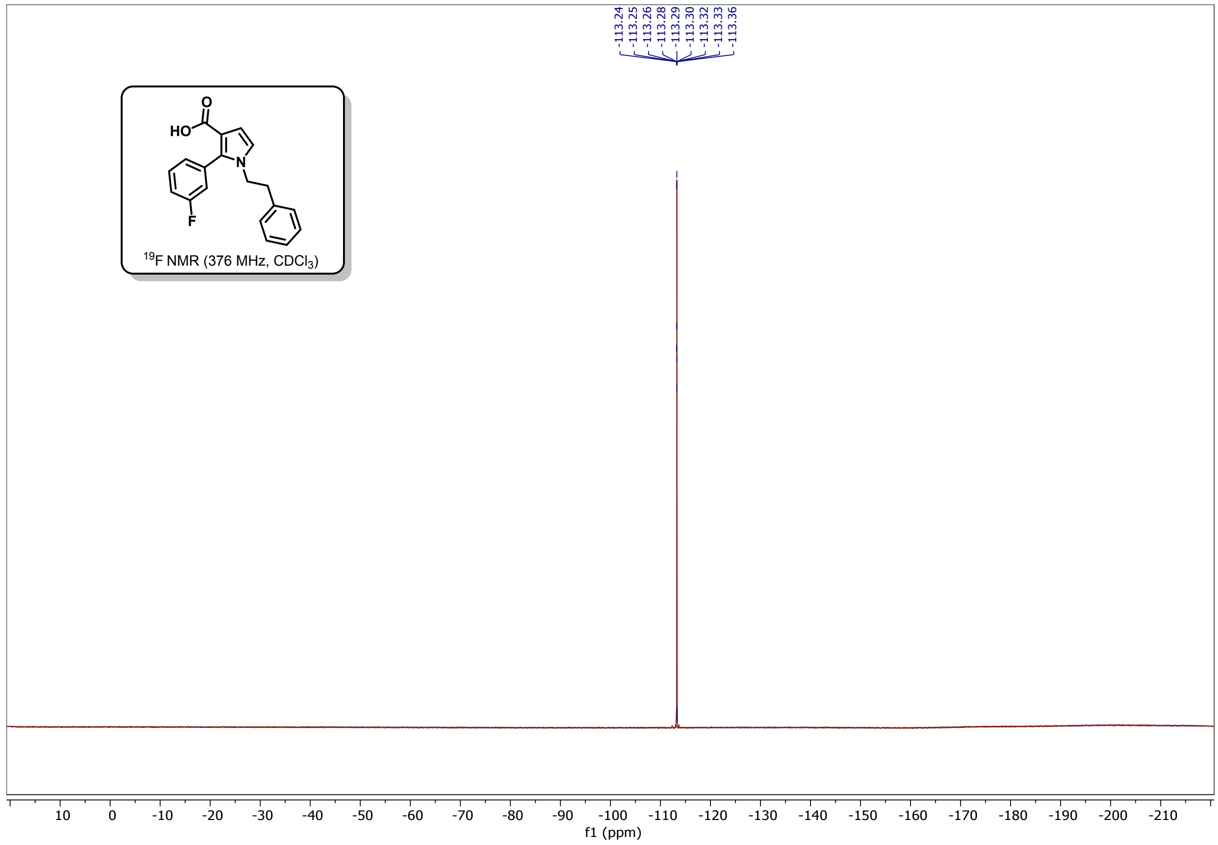

-113.24
-113.25
-113.26
-113.28
-113.29
-113.30
-113.32
-113.33
-113.36
HO
O
N
F
^{19}F NMR (376 MHz, CDCl$_3$)
10 0 -10 -20 -30 -40 -50 -60 -70 -80 -90 -100 -110 -120 -130 -140 -150 -160 -170 -180 -190 -200 -210
f1 (ppm)

N-allyl-2-(3-fluorophenyl)-1-phenethyl-1H-pyrrole-3-carboxamide (5aa'):

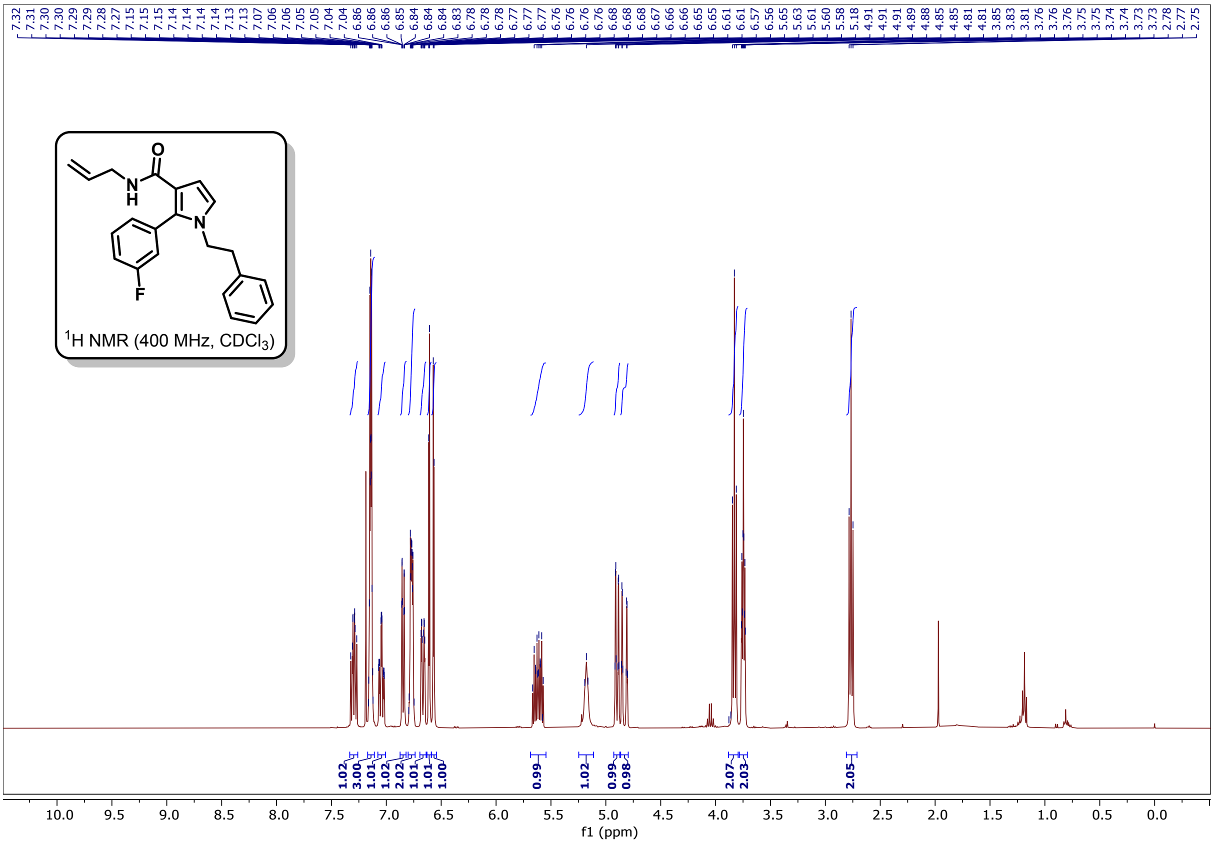

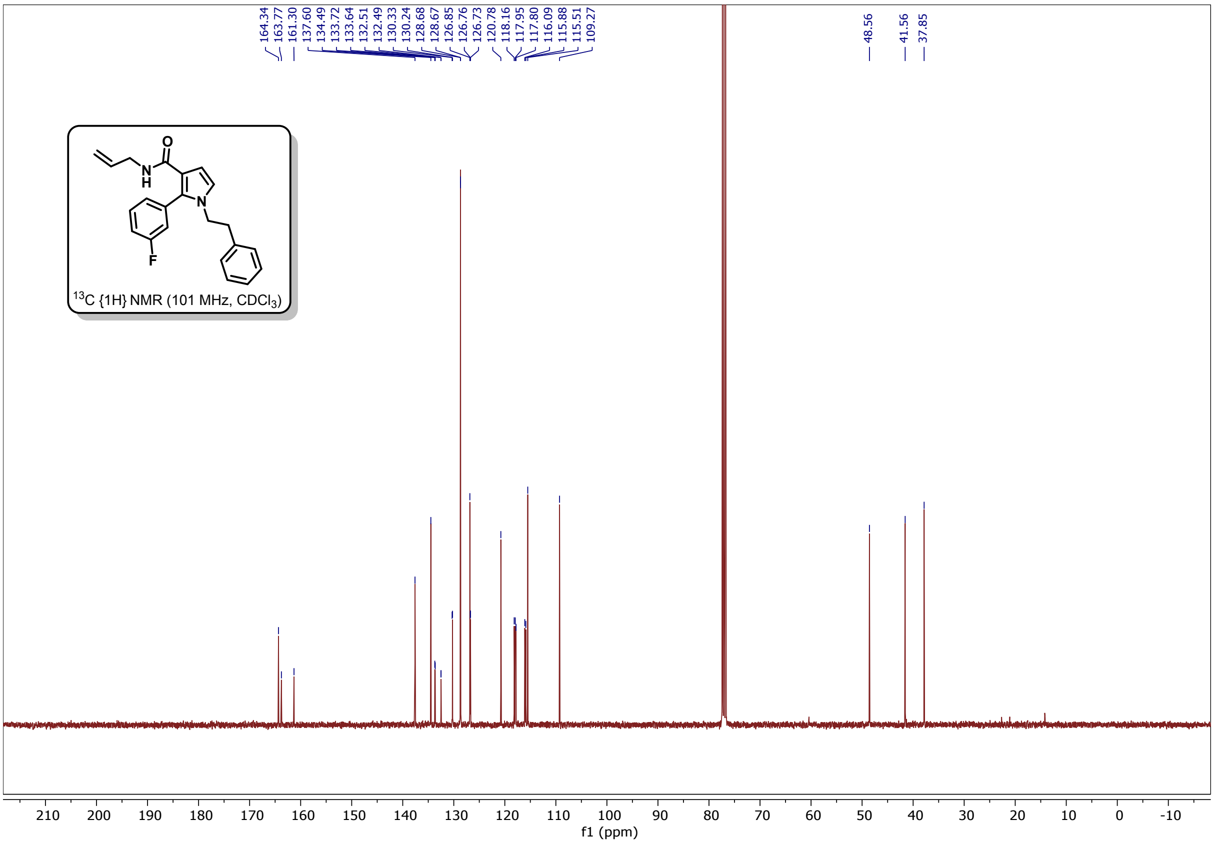

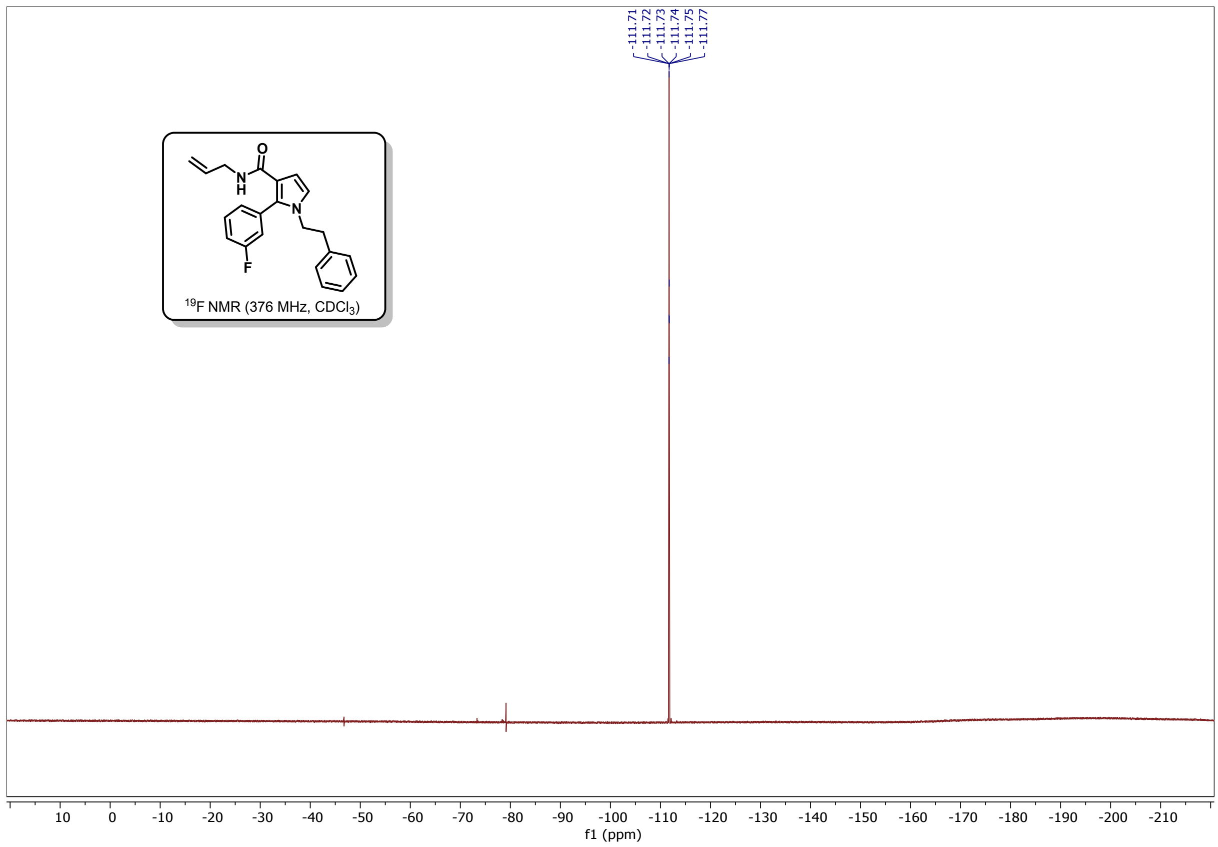
-111.71
-111.72
-111.73
-111.74
-111.75
-111.77
^{19}F NMR (376 MHz, CDCl$_3$)
10 0 -10 -20 -30 -40 -50 -60 -70 -80 -90 -100 -110 -120 -130 -140 -150 -160 -170 -180 -190 -200 -210
f1 (ppm)

REFERENCES

(1) D. Chen, L. Xu, B. Ren, Z. Wang and C. Liu, *Org. Lett.*, 2023, **25**, 4571–4575.
